# Gold(I)-Catalysed Direct Thioetherifications Using Allylic Alcohols: an Experimental and Computational Study

**DOI:** 10.1002/chem.201403293

**Published:** 2014-07-30

**Authors:** Lorena Herkert, Samantha L J Green, Graeme Barker, David G Johnson, Paul C Young, Stuart A Macgregor, Ai-Lan Lee

**Affiliations:** [a] Institute of Chemical Sciences, Heriot-Watt University Edinburgh, EH14 4AS (UK) E-mail: S.A.Macgregor@hw.ac.uk A.Lee@hw.ac.uk

**Keywords:** allylation, allylic alcohols, gold, mechanism, thiols

## Abstract

A gold(I)-catalysed direct thioetherification reaction between allylic alcohols and thiols is presented. The reaction is generally highly regioselective (S_N_2′). This dehydrative allylation procedure is very mild and atom economical, producing only water as the by-product and avoiding any unnecessary waste/steps associated with installing a leaving or activating group on the substrate. Computational studies are presented to gain insight into the mechanism of the reaction. Calculations indicate that the regioselectivity is under equilibrium control and is ultimately dictated by the thermodynamic stability of the products.

## Introduction

Thioether linkages are present in many pharmaceuticals, natural products and synthetic intermediates, and as such, thioethers are compounds of both academic and industrial importance.[1] Allylic thioethers also play an important role in bioorganic chemistry[2] and as building blocks for organic synthesis.[3] Despite this, reports using thiols as nucleophiles with late transition metals are nowhere near as numerous as with other nucleophiles, because sulfur is known to deactivate/poison late transition metal catalysts.[4] In the active field of homogenous gold-catalysis,[5] for example, successful reports of thiols as nucleophiles are relatively scarce.[6, 7] Indeed, we have recently shown that thiols in the presence of cationic gold(I) catalysts are in equilibrium with an inactive digold species [{Au(L)}_2_(μ-SR)]X, which can slow down (or in some cases shut down) catalytic reactions.[6g, 8]

One of the research efforts within our group has been to develop gold-catalysed regioselective methods toward allylic ethers.[9] Within this context, we recently developed a gold(I)-catalysed *direct* allylic etherification[10] of unactivated alcohols.[11] Unlike previous methods for S_N_2′ allylic etherifications,[12] neither the allylic alcohol electrophile[13] nor the alcohol nucleophile need to be activated (either to install a leaving group in the former or form an alkoxide in the latter), leading to mild reaction conditions which are tolerant of various functional groups. Despite the possible deactivation of gold catalysts by thiols, the potential to develop a mild, selective and atom economical *thio*etherification procedure led us to explore the title reaction.

**Scheme 1:**
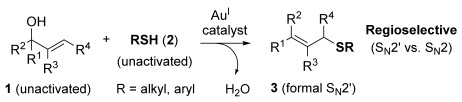
Direct allylic thioetherifications using gold(I)-catalysis.

We herein report the first gold-catalysed direct thioetherification method using unactivated allylic alcohols and unactivated thiols as substrates (Scheme 1). This dehydrative allylation procedure is therefore very atom economical, producing only water as the by-product and avoiding any unnecessary waste/steps associated with installing a leaving group on the allyl alcohol and activating additive/group on the thiol. Most traditional metal-catalysed S-allylation methods are not atom efficient, requiring a leaving group and/or thiol activation.[14] More recently, Ir- and Ru-catalysed dehydrative S-allylation procedures have been developed to overcome this issue, however, Ir- and Ru-catalysed methods all give the opposite (formal S_N_2) regioselectivity, via a presumed π-allyl intermediate.[15] By using gold(I)-catalysis and thereby accessing a different mechanistic route (vide infra), direct dehydrative S-allylation by S_N_2′ regioselectivity is now possible for the first time. We also present a computational study of the mechanism of this process, and through this account for the regioselectivities observed experimentally: this was found to be dictated by the thermodynamic stabilities of the products.

## Results and Discussion

Our investigations commenced with the optimisation of the reaction conditions using allylic alcohol **4** and thiophenol **2 a** (Table 1). We have previously ascertained that Echavarren’s catalyst **5**[16] is tolerant of deactivation by sulfur nucleophiles,[6g, 8] so **5** was adopted as our preferred catalyst in these studies. Since a thiol is more nucleophilic than an alcohol, the thiol nucleophile does not need to be in large excess (c.f. 5 equiv alcohol nucleophile for the related etherification reaction to avoid self-reaction of **1**),[11] so 1–1.1 equiv of the thiol nucleophile is sufficient for direct thioetherifications (Table 1). A slightly higher temperature of 40 versus 30 °C is clearly useful for higher conversions (entries 1 and 2) and 24 h appears to be enough for a reasonable conversion (entries 2–5). Next, chlorinated solvents DCE and chloroform pushed the reaction to higher conversions (90 and 92 % respectively, entries 6 and 7) but crucially also provided cleaner conversions, with no sign of the inseparable and unidentified side products which appeared in the toluene reactions. A further temperature screen using chloroform as solvent (entries 7–9) suggests that 35 °C is the ideal compromise between mild conditions and good conversions. Thus, the conditions in entry 8, Table 1 were adopted as the optimised general conditions.

**Table 1:** Selected optimisation and control reactions

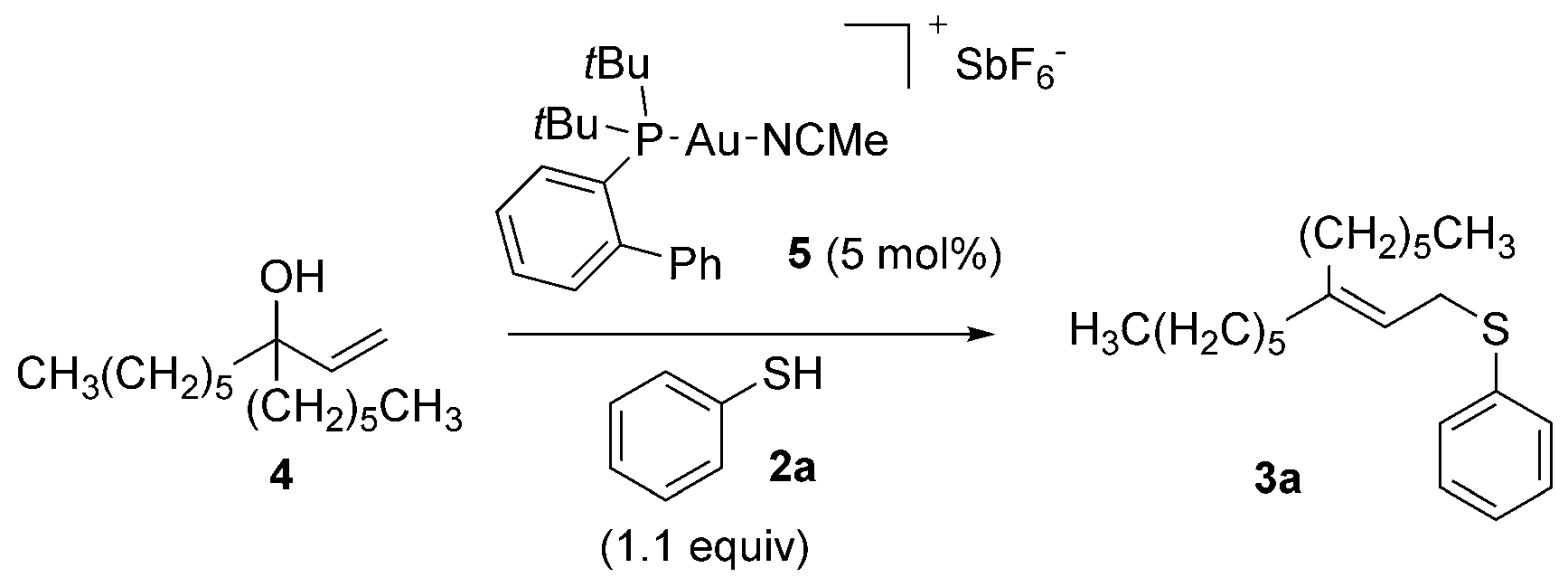				
Entry	*T* [°C]	Solvent^[a]^	*t* [h]	Conv. [%]^[b]^
1	30	toluene	72	46^[c]^
2	40	toluene	72	88^[c]^
3	40	toluene	8	67^[c]^
4	40	toluene	24	81^[c]^
5	40	toluene	48	82^[c]^
6	40	DCE	24	90
7	40	CHCl_3_	24	92
8	35	CHCl_3_	24	91
9	30	CHCl_3_	24	79
10^[d]^	40	toluene	24	0
11^[e]^	40	toluene	24	76
12^[f]^	40	toluene	24	9

[a] 0.4 m. [b] Determined by ^1^H NMR analysis of the crude mixture. [c] Unidentified side-products were observed in toluene. [d] No gold catalyst added. [e] 2,6-Di-*tert-*butylpyridine (5 mol %) added. [f] 4 Å molecular sieves added.

A few control reactions were also carried out in order to ascertain if gold is acting as a catalyst. In the absence of a gold catalyst, the reaction does not proceed at all (entry 10). When a sterically hindered mild base 2,6-di-*tert*-butylpyridine was added to the gold-catalysed reaction (to mop up any trace acid which may form in the reaction), the reaction still proceeds well (entry 11), suggesting that the reaction is gold- and not trace-acid catalysed. Addition of 4 Å molecular sieves was also investigated (to help remove any water formed during the reaction) but this appeared to have a detrimental effect on the conversion (entry 12) and was thus not investigated further.

With our optimised results in hand, a thiol nucleophile screen was carried out using allylic alcohols **4** as the model substrate (Table 2). A range of thiophenols **2 a**–**2 i** reacts smoothly to give the desired allylic thioether **3 a–3 i** (entries 1–9). Thiols with substitutuents at the *ortho*, *meta* and *para* position perform well (entries 2–6) as do those with electron-donating (entries 2–6 and 9) and electron-withdrawing substituents (entries 7 and 8). The bromo-substituent in **3 h** also provides a handle for further functionalisation. Next, a competing functional group (OH in 4-mercaptophenol **2 i**) was investigated, as phenols have been shown to form chromans or Friedel–Crafts allylation products under gold-catalysis with allylic alcohols.[11a, 17] Pleasingly, the reaction proceeds chemoselectively at the thiophenol site, although the yield is somewhat lower than with other thiophenols (49 %, entry 9). This could be due to the unprotected OH group slightly interrupting the H-bonded 6-membered ring transition state (vide infra, Scheme 3).

**Table 2:** Thiol nucleophile scope

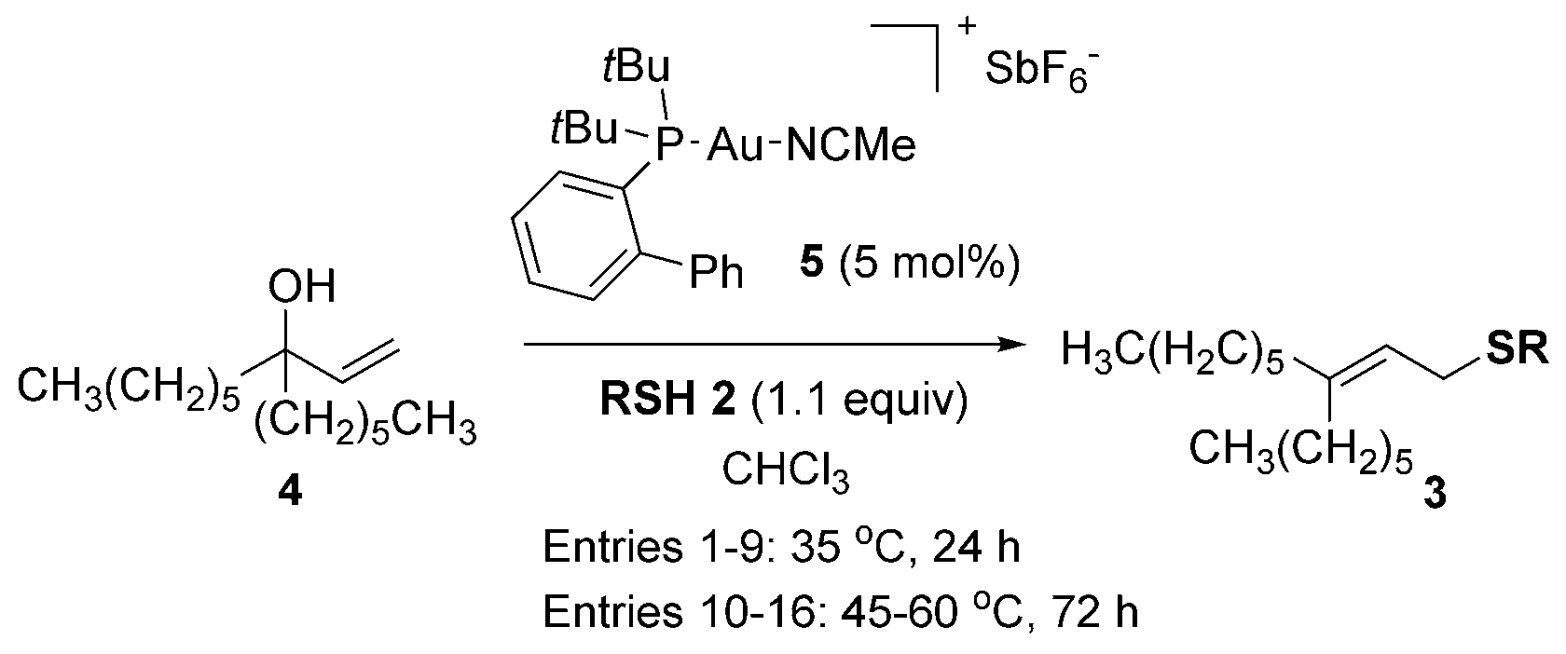				
Entry	*T* [°C]	Thiol2	Product	Yield [%]^[a]^
1	35	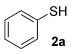	**3 a**	70
2	35	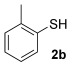	**3 b**	62
3	35	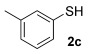	**3 c**	76
4	35	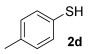	**3 d**	71
5	35	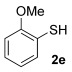	**3 e**	66
6	35	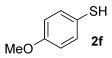	**3 f**	64
7	35	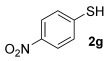	**3 g**	68
8	35	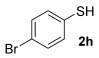	**3 h**	73
9	35	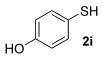	**3 i**	49
10	35	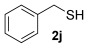	**7 j**	42
11^[b]^	50	BnSH **2 j**	**3 j**	56
12^[b]^	50	*n*BuSH **2 k**	**3 k**	47
13	60	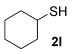	**3 l**	40
14^[b]^	60	CySH **2 l**	**3 l**	56
15^[b]^	50	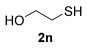	**3 n**	45
16^[b]^	45	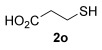	**3 o**	92^[c]^

[a] Isolated yield. Regioselectivity S_N_2′/S_N_2>20:1 unless otherwise stated. [b] Resin-bound scavenger Reaxa QuadraPure™ MPA added at the end of reaction. [c] Formal S_N_2′/S_N_2 ratio 18:1.

**Scheme 2:**
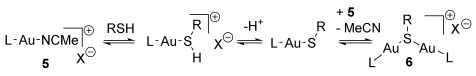
Postulated mechanism for formation of 6 from 5.

Next, we moved from thiophenols to alkyl thiols (entries 10–16). This change of S-nucleophile class initially caused several problems. Firstly, the reactions are a lot slower, despite alkyl thiols being more nucleophilic. We have recently ascribed this to the fact that the higher Lewis basicity of alkyl thiols (vs. thiophenols) will push the equilibrium towards the deactivated [{Au(L)}_2_(μ-SR)]SbF_6_ species **6**, resulting in a lower concentration of active catalyst in solution (Scheme 2).[8] Nevertheless, higher temperatures (50–60 °C) and longer reaction times (72 h) allow for successful conversions to the desired allylic thioether products.

**Figure 1:**
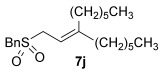
Oxidised product 7 j.

Having overcome the first reactivity issue, a second problem soon emerged, especially when primary alkyl thiols were employed as nucleophiles. Although the desired allylic thioether (e.g., **3 j**) is observed in the crude mixture, the product oxidises upon silica gel chromatography to give the sulfone **7 j** (Figure 1) instead (entry 10). Attempts to stop the oxidation by using an alumina column met with only moderate success (16 % **3 j**). Since control reactions show that leaving **3 j** in air, silica gel or alumina, respectively, does not cause any oxidation, we surmised that it must be a combination of the gold catalyst, silica and air during column chromatography which causes the oxidation. Indeed, removing the gold using a resin-bound scavenger Reaxa QuadraPure™ MPA[10f] prior to purification by column chromatography solves the oxidation problem and allows the allylic thioether **3 j** to be obtained successfully in 56 % yield (entry 11).

The secondary alkyl thiol **2 l** was less prone to oxidation (entry 13), implying that the oxidation is sensitive to steric hindrance. Nevertheless, the yield of **3 l** is still improved upon scavenging the gold catalyst prior to purification by column chromatography (56 vs. 40 %, entries 13 and 14). Finally, chemoselectivity was probed by using alkyl thiols with pendant functional groups (entries 15 and 16). The hydroxyl group in **2 n** is tolerated, although it produces a modest 45 % yield (entry 15). As with entry 9, a plausible cause for this lower yield with **2 n** is the disruption of the H bonding in the 6-membered transition state by the –OH pendant group (vide infra). In contrast, a carboxylic acid pendant group is tolerated very well (**2 o**, entry 16), providing **3 o** in 92 % yield. It should be noted, however, that while entries 1–15 all provide exclusively S_N_2′ regioselectivity, **3 o** is the only thiol investigated to show traces of the formal S_N_2 isomer (∼5 %). Nevertheless, the selectivity is still very good (18:1 S_N_2′/S_N_2).

Having established the sulfur nucleophile scope, we turned our attention to the allylic alcohol substrate scope (Table 3). Initially, substituent effects on tertiary allylic alcohols were investigated (entries 1–9). Increasing the steric bulk of the substituents (*n*-hexyl→cyclohexyl) is tolerated and in fact produces a good 86 % yield (entries 1 and 2). Where *E*/*Z* isomers are possible, the reaction is selective for the *E* isomer (entries 3–5), with the *E*/*Z* ratios increasing the better the steric differentiation between the two substituents (e.g., >20:1 for **3 q** but a lower 9:1 for **3 r**). An exocyclic tertiary allylic alcohol substrate **12** is also tolerated well (entry 6). Next, the effect of substitution around the alkene was investigated, thus, γ-substituted (entries 7 and 8) and β*-*substituted (entry 9) tertiary allylic alcohols were subjected to the reaction conditions. Pleasingly, both the γ-substituted and the β-substituted substrate reacted to give decent yields (50, 72 and 61 %, respectively), with **3 v** also providing >20:1 *E*/*Z* selectivity.

**Table 3:** Allylic alcohol scope

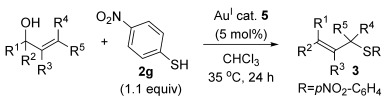				
Entry	Allylic alcohol	Product	Yield [%]^[a]^	Note^[b]^
1	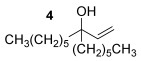	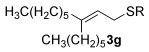	68	–
2	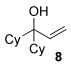	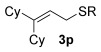	86	–
3	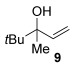	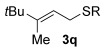	59	>20:1 *E*/*Z*
4	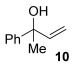	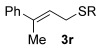	59	9:1 *E*/*Z*
5	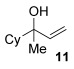	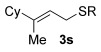	73	∼6:1 *E*/*Z*
6	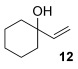	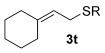	55	–
7	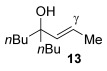	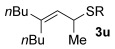	50	–
8	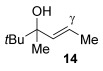	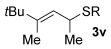	72	>20:1 *E*/*Z*
9	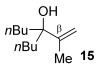	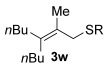	61	–
10^[c]^	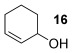	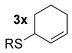	89	–
11^[c]^	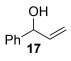	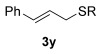	64	>20:1 *E*/*Z*
12^[c]^	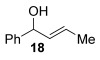	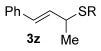	72	>20:1 *E*/*Z*
13^[c]^	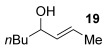	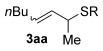	65	∼3:2 *E*/*Z*, 8:1 S_N_2′/S_N_2
14^[c]^	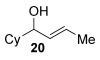	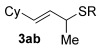	85	>20:1 *E*/*Z*, 2:1 S_N_2′/S_N_2
15^[c]^	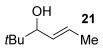	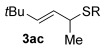	71	4:1 *E*/*Z*
16	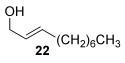	–	N.D.^[d]^	poor conv.
17	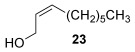	–	N.D.^[d]^	poor conv.
18	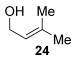	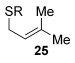	59	“S_N_2” regioselectivity
19	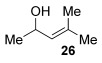	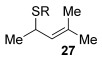	87	“S_N_2” regioselectivity

[a] Isolated yield. Regioselectivity S_N_2′/S_N_2>20:1 unless otherwise stated. [b] Determined by ^1^H NMR analysis. [c] 45 °C for 72 h. [d] Not determined. Poor conversions and complex mixture of products.

Next, secondary allylic alcohols were investigated as substrates (entries 10–15). The secondary allylic alcohols require a higher temperature (45 °C) and longer reaction time (72 h) to go to completion. Nevertheless, cyclic allylic alcohol **16** provides a very good 89 % yield of desired product **3 x** (entry 10). Acyclic allylic alcohol **17** as well as γ-substituted secondary alcohol **18** (both with a Ph substituent) also react smoothly (entries 11 and 12). Replacing the Ph substituent in **18** with alkyl substituents (**19**–**21**), however, provided some of the poorest selectivities of all the allylic alcohols screened (entries 13–15). Firstly, the *n*Bu substituted **19** provided a reasonable 8:1 formal S_N_2′/S_N_2 regioselectivity, but a very poor 3:2 *E*/*Z* selectivity (entry 13). Increasing the steric hindrance from *n*Bu to Cy (**20**) gives a good 85 % yield and drastically improves the *E*/*Z* selectivity (>20:1) but the regioselectivity drops (S_N_2′/S_N_2 2:1, entry 14). Finally, increasing the bulkiness of the substituent even further from Cy to *t*Bu (**21**) provides excellent (>20:1) regioselectivity again, with a decent 4:1 *E*/*Z* ratio (entry 15).

Reactions with primary allylic alcohols were significantly less straightforward (entries 16–18). Both the *trans* and *cis* primary allylic alcohols **22** and **23** were reluctant to undergo the reaction, providing mainly unreacted starting material and a complex mixture of other unidentified products (entries 16 and 17). In contrast, γ,γ-disubstituted primary allylic alcohol **24** reacts smoothly, but gives the opposite formal S_N_2 regioselectivity (**25** instead of **3**, entry 18). In order to ascertain if it is the γ,γ-substitution which causes a switch of regioselectivity, or if it is specific to the primary allylic alcohol **24**, the secondary γ,γ-substituted alcohol **26** was investigated next (entry 19). Indeed, the formal S_N_2 product **27** is formed exclusively in good 87 % yield. Therefore, it appears that although the direct thioetherification is in general S_N_2′ selective (Table 2 and entries 1–15, Table 3), γ,γ-substitution causes a complete switch in regioselectivity (entries 18 and 19, Table 3).

Having completed our studies on the substrate scope, we were keen to extend this methodology to asymmetric methods. Although the majority of the products formed by this method are achiral, some of the γ-substituted substrates may be amenable to chirality transfer. In theory, an enantioenriched chiral allylic alcohol with a γ-substituent (such as **18**, entry 12), which is easily accessible in high *ee* values by Sharpless kinetic resolution[18] or enzyme resolution,[19] should be able to transfer its chirality[20] to the allylic thioether product **3 z**, especially if a 6-membered ring H-bonded intermediate is involved. Disappointingly, our initial attempt at chirality transfer with enantioenriched secondary allylic alcohol (*S*)-**18** (91:1 e.r.)[21] produced only racemic product **3 z**. Suspecting that the phenyl substituent in **18** is to blame (the phenyl substituent may be stabilising a planar allylic cation intermediate which may be otherwise disfavoured), enantioenriched allylic alcohol (*R*)-**21** (94:6 e.r.)[19] and (*R*)-**20** (>99:1 e.r.) were investigated instead. Unfortunately, the corresponding products **3 ac** and **3 ab** were also racemic. These results are surprising as the allylic etherification counterpart (alcohol additions to allylic alcohols) is known to proceed by chirality transfer.[11b, 22] Therefore, although the lack of chirality transfer in the above γ-substituted cases is disappointing, it does provide valuable indication that the factors controlling allylic etherification and allylic thioetherification may be subtly different.

**Scheme 3:**
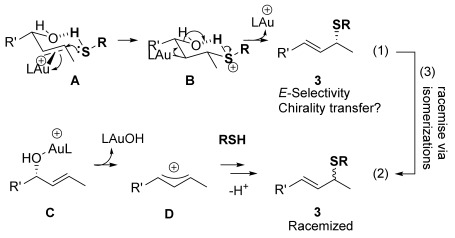
Plausible mechanisms. Calculations and control studies point toward 1 and 3 being the likely pathways.

If the direct allylic thioetherification proceeds in a similar fashion to the etherification procedure,[11a, b] then chirality transfer would be expected (Eq. (1), Scheme 3). Since gold(I) is an excellent π-Lewis acid,[5e] it is likely to activate the alkene functionality in the allylic alcohol towards attack by a thiol nucleophile (**A**, Scheme 3).[10g] Demetallation and elimination of water (possibly enabled by intramolecular H bonding, **B**), will then regenerate the catalyst and produce the desired allylic ether product **3**. Such a chair-like 6-membered ring transition state would be crucial for any chirality transfer (Eq. (1), Scheme 3).

A possible alternative mechanism to explain the racemisation is that the reaction proceeds via an allyl cation **D** (Eq. (2), Scheme 3). Gold(I) catalysts are also known to coordinate to alcohols,[23] in this case turning the hydroxyl group into a better leaving group (**C**). Attack at the less hindered position could occur on **D** to provide the product **3**. Active catalyst LAu^+^ is presumably regenerated by protonolysis of LAuOH.[24] Such a mechanism could explain why the reaction is easiest with tertiary allylic alcohols (e.g., **13**, **14**), slightly harder with secondary (**18**–**21**) and does not work with primary (**22** and **23**; at least when comparing mono-γ-substituted substrates), due to differences in the stabilisation of the cationic intermediate.

This alternative mechanism, however, is not totally consistent with all observations. For example, if the reaction indeed goes via an allylic cation **D**, the reaction with **19** might be expected to have poor regioselectivity (primary alkyl substituent at both ends of **D**), but a good 8:1 formal S_N_2′/S_N_2 selectivity is observed. Therefore, a third alternative might be that the reaction goes through Equation (1), but the product **3** then racemises (by isomerisation between formal S_N_2′ and formal S_N_2 type products, Eq. (3), Scheme 3), especially in cases where higher reaction temperatures and times are required to push the reaction to completion (e.g., entries 10–15, Table 3). Indeed, the mechanism could also change depending on the substituents present.

Density functional theory (DFT) calculations have been performed to probe these selectivity issues.[25] The calculations mainly employed a [(Me_3_P)Au(MeCN)]^+^ model catalyst, **5′**, and initially considered the reaction of PhSH with Me_2_C(OH)CH=CH_2_, **4′**, a simple model of allylic alcohol **4** with the *n*-hexyl groups replaced by methyls. Subsequent calculations with the full Echavarren catalyst, **5**, provided very similar energetics, details of which are given in the Supporting Information. We report free energies derived from gas-phase optimisations with the BP86 functional, corrected for chloroform solvent (PCM method) and dispersion effects (i.e., at the BP86-D3(CHCl_3_) level).

**Scheme 4:**
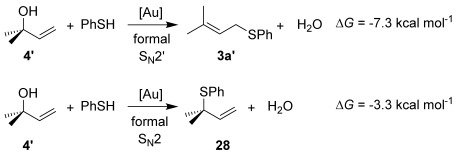
Thermodynamics of the alternative products derived from 4′: formal S_N_2′ product 3 a′ and formal S_N_2 product 28.

As a first step, we considered the thermodynamics of the alternative formal S_N_2′ and S_N_2 products that can be formed (along with water) through the reaction of **4′** with PhSH (Scheme 4). Both processes are exergonic, but the formal S_N_2′ product, **3 a′**, is computed to be 4 kcal mol^−1^ more stable than the alternative formal S_N_2 product **28**.

**Figure 2:**
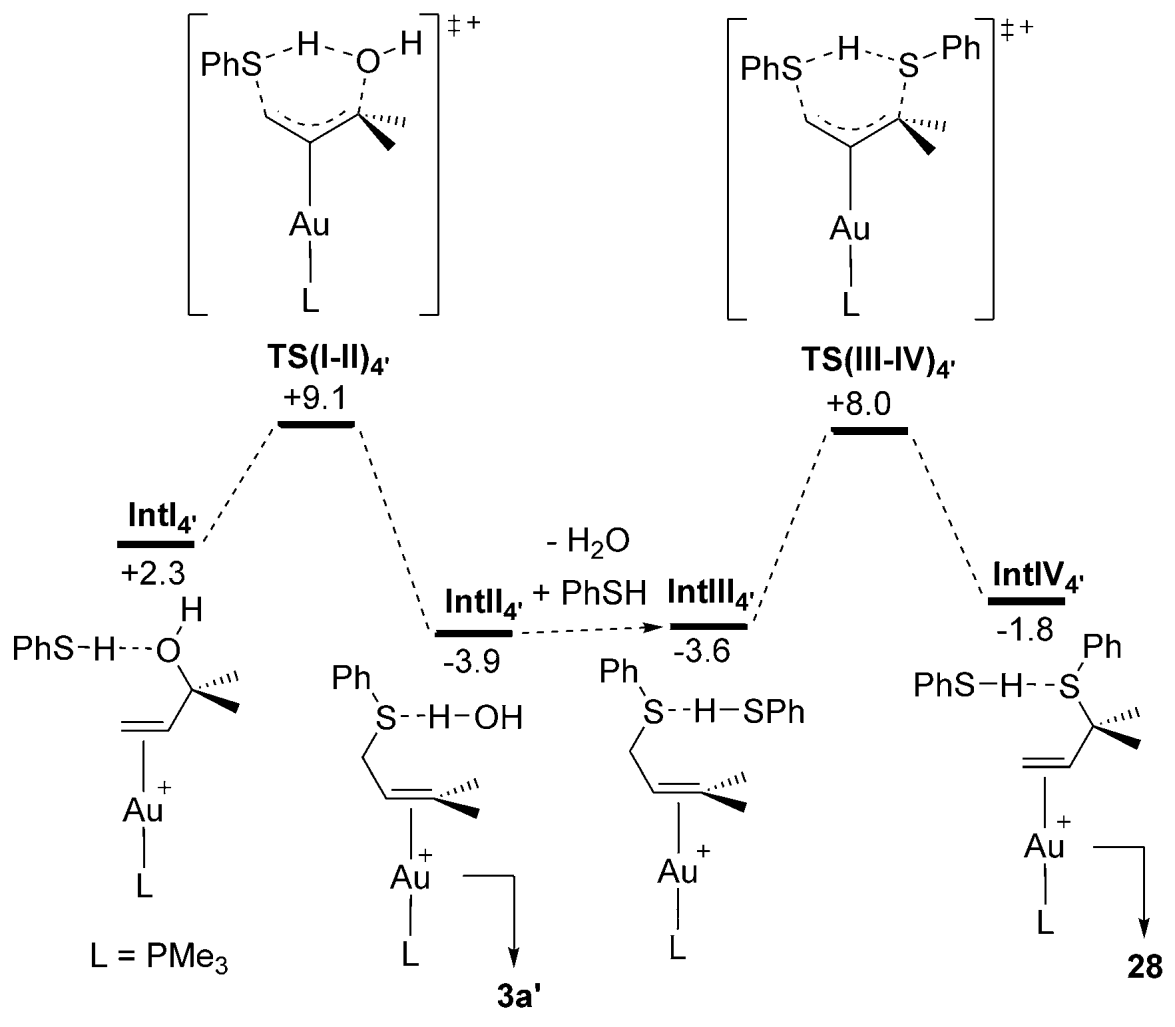
Computed free energy reaction profile (BP86-D3(CHCl_3_), kcal mol^−1^) for the reaction of 4′ with PhSH at model catalyst 5′ to form 3 a′ and 28.

**Figure 3:**
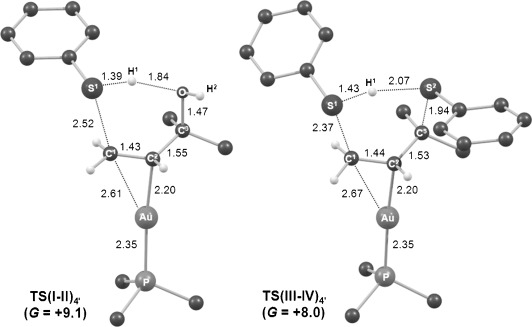
Computed geometries of S_N_2′ transition states TS(I-II)_4′_ and TS(III-IV)_4′_ with selected distances [Å] and free energies [kcal mol^−1^].

A computed mechanism for the formation of **3 a′** and **28** is summarised in Figure 2, where free energies are quoted relative to the model catalyst **5′** and the separated reactants (**4′** and PhSH). The profile starts from **IntI_4′_**, formed from **5′** by displacement of MeCN by a π-bound allylic alcohol which is itself H bonded to the external PhSH. The latter is located *anti* to the metal centre and so is set up for external attack of the thiol at the γ-position (C^1^, see Figure 3 for atom numbering). This S_N_2′ step proceeds with concerted proton transfer and loss of H_2_O to give **IntII_4′_**, where the allylic thioether product is now π-bound to gold and H_2_O is H bonded to the sulfur centre.[26] The process occurs via **TS(I-II)_4′_** with a modest barrier of only 6.8 kcal mol^−1^. Subsequent conformational searching revealed several additional forms of **IntI_4′_**, the most stable of which has *G*=−2.5 kcal mol^−1^. This species, **IntI_4′b_**, is related to **IntI_4′_** by rotation about the C^2^–C^3^ bond which then places the {HOLHSPh} moiety over the Au centre. The overall barrier for the S_N_2′ process, relative to this low energy precursor, is therefore 11.6 kcal mol^−1^ and so remains readily accessible (assuming facile interconversion of **IntI_4′b_** and **IntI_4′_**).[27, 28] From **IntII_4′_** the S_N_2′ product **3 a′** can then either be released, or alternatively a second S_N_2′ step with PhSH can lead to the net formal S_N_2 product **28**. Thus, displacement of H_2_O from **IntII_4′_** with PhSH gives **IntIII_4′_** (*G*=−3.2 kcal mol^−1^). Thiol attack via **TS(III-IV)_4′_** (*G*=+8.0 kcal mol^−1^) then generates **IntIV_4′_** (*G*= −1.8 kcal mol^−1^) in which the net S_N_2 product **28** is now π-bound to the gold centre.2

The computed structures of **TS(I-II)_4′_** and **TS(III-IV)_4′_** are given in Figure 3. For **TS(I-II)_4′_** the main geometric feature is a slipping of the alkene moiety induced by the approach of the thiol, with an elongation of the Au–C^1^ distance to 2.61 Å compared with 2.30 Å in **IntI_4′_**. At this stage there is relatively little elongation of either the S^1^–H^1^ (1.39 Å compared with 1.37 Å in **IntI_4′_**) or the C^3^–O bonds (1.47 Å compared with 1.46 Å in **IntI_4′_**) and in these terms the transition state exhibits an early geometry. In contrast **TS(III-IV)_4′_**, equating to nucleophilic attack of a second PhSH at C^3^, has a late geometry with the new C^3^–S^2^ bond nearly fully formed (1.94 Å compared with 1.92 Å in **IntIV_4′_**), extension of the C^1^LS^1^ distance to 2.37 Å and slippage of the gold away from the C^2^–C^3^ bond such that it begins to interact with C^1^ (AuLC^1^=2.67 Å).3

The low barriers and moderate exergonicities associated with the two S_N_2′ steps in Figure 2 suggest that both processes should be readily accessible and reversible at the temperatures used experimentally. The similar energies of the different π-bound intermediates also suggest that a mixture of such species will be formed in solution during the catalysis, although **IntII_4′_**, the immediate precursor to the observed S_N_2′ thioether product (here modeled by **3 a′**), is the most stable of these. This is also consistent with the reaction conditions, which require relatively long reaction times to achieve equilibrium, but without significant heating. The experimentally observed selectivity for the S_N_2′ product reflects therefore the greater thermodynamic stability of this species over the alternative formal S_N_2 product **28**, rather than any kinetic preference for the S_N_2′ product.

**Scheme 5:**
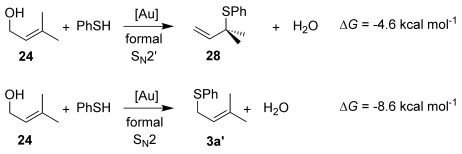
Thermodynamics of the alternative products derived from 24: formal S_N_2′ product 28 and formal S_N_2 product 3 a′.

This thermodynamic basis for regioselectivity appears quite general, for example a clear preference was also computed for the S_N_2′ thioetherification products formed between PhSH and substrates **9**, **10** and **11** (models of **3 q**, **3 r** and **3 s**, respectively, Table 3) over their formal S_N_2 alternatives. Experimentally, γ,γ-disubstituted substrates **24** and **26** were exceptions in forming S_N_2 products. Calculations showed that this is consistent with a change in the relative energies of the products with **3 a′** (here a model of **25**, the formal S_N_2 product derived from **24**), being 4.0 kcal mol^−1^ more stable than the alternative S_N_2′ product **28** (Scheme 5).

**Figure 4:**
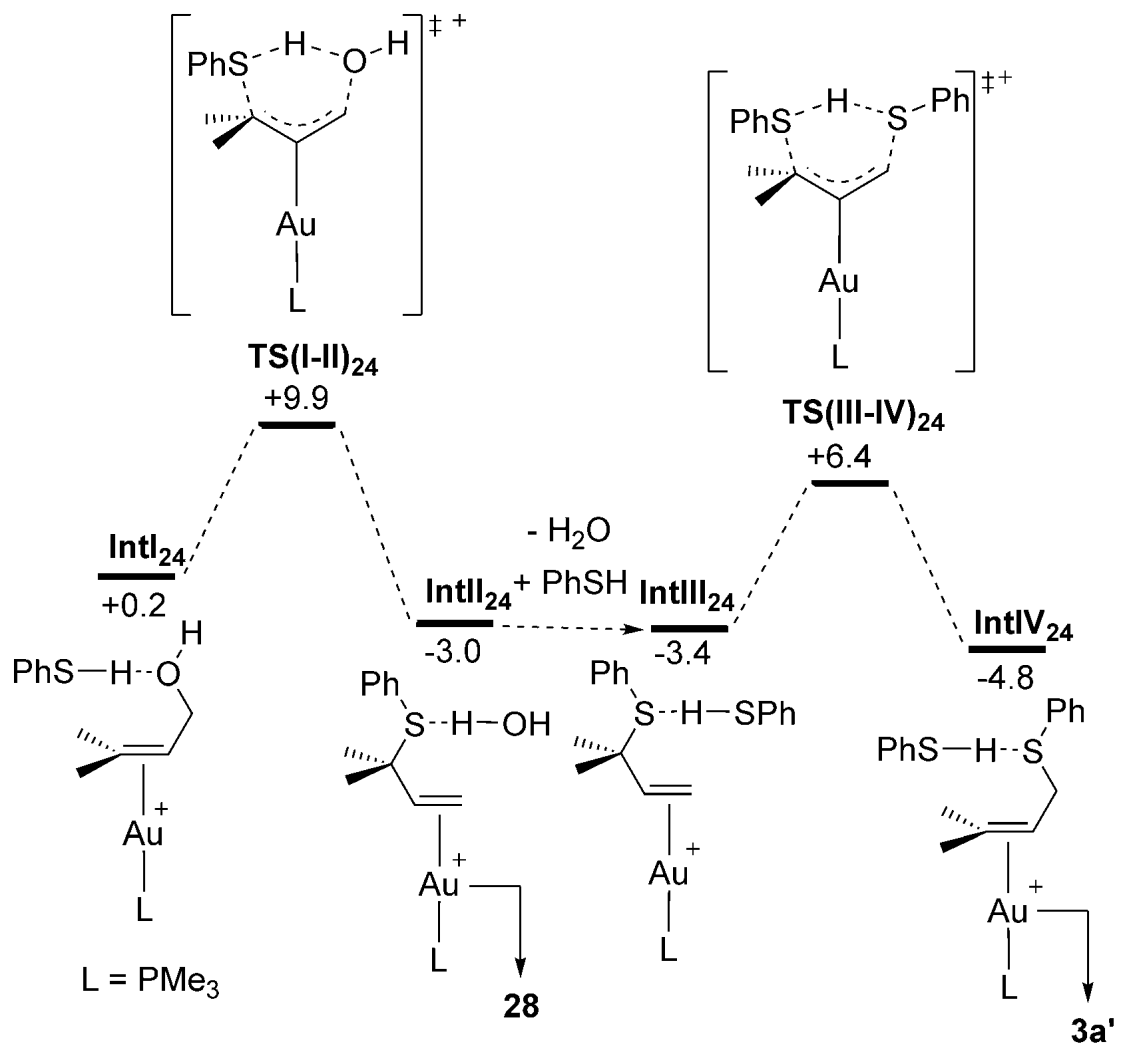
Computed free energy reaction profile (BP86-D3(CHCl_3_)) for the reaction of 24 with PhSH at model catalyst 5′ to form 28 and 3 a′.

**Figure 5:**
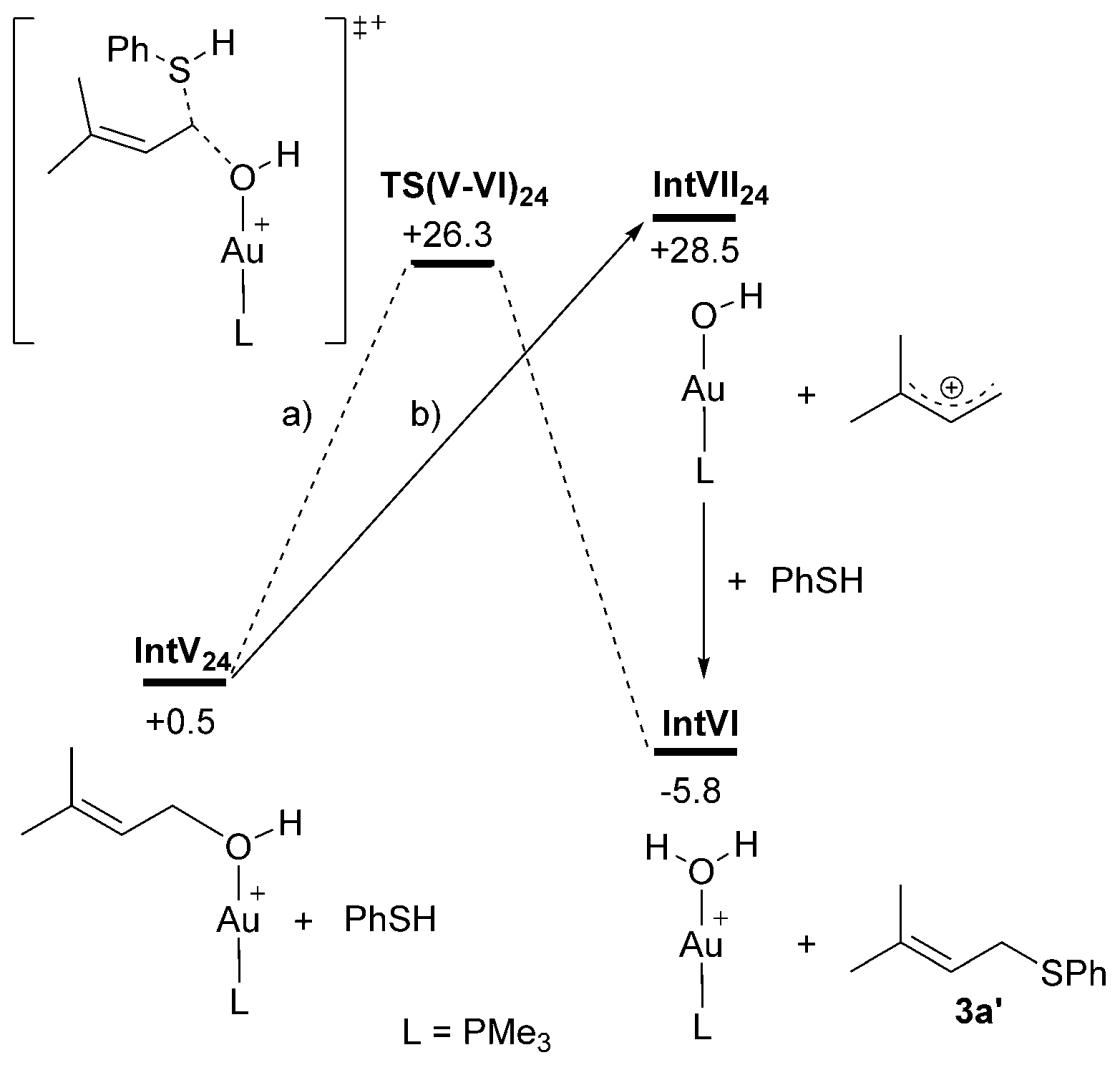
Alternative pathways for the formation of 3 a′ from O-bound precursor IntVI_24_ via: a) direct S_N_2 attack, and b) an allylic cation. Intermediate free energies at the BP86-D3(CHCl_3_) level are in kcal mol^−1^.

Due to the different regioselectivity in this case we also confirmed that the formal S_N_2 product, **3 a′**, is readily formed via two sequential S_N_2′ steps (Figure 4). Thus, nucleophilic attack of PhSH with **IntI_24_** proceeds through **TS(I-II)_24_** at +9.9 kcal mol^−1^ to give **IntII_24_** in which **28** is bound to the Au centre. A second S_N_2′ step with PhSH then occurs via **TS(III-IV)_24_** (*G*=+6.4 kcal mol^−1^) to give **IntIV_24_** at −4.8 kcal mol^−1^ from which **3 a′** can be released. The overall shape of the free energy surface in Figure 4 reiterates the facile reversibility of these processes, with the immediate precursor to the observed product (**IntIV_24_**) again corresponding to the most stable intermediate. In this case we also considered whether the formal S_N_2 product **3 a′** could be formed by a direct S_N_2 reaction of PhSH at an O-bound precursor, **IntV_24_** (Figure 5 a). **IntV_24_** has a relative free energy of +0.5 kcal mol^−1^ suggesting it is accessible in solution. However, a computed barrier via **TS(V-VI)_24_** of 26.3 kcal mol^−1^ indicates this process is disfavoured kinetically relative to the facile sequential S_N_2′ steps shown in Figure 4. Inclusion of an additional PhSH molecule in the calculation (to act as an initial proton acceptor in the transition state) did not result in any reduction in the barrier (see the Supporting Information). Similarly the formation of an allylic cation (**C**→**D** Scheme 3 and Figure 5 b) can be discounted as this process is associated with a free energy change of +28.5 kcal mol^−1^. We also tested the formation of the analogous allylic cation from substrate **17** and found that, while this is somewhat more accessible, it does remain strongly endergonic, by +23.9 kcal mol^−1^. Allylic cation formation therefore cannot account for the lack of chirality transfer observed with enantioenriched substrate **17**, despite the presence of the potentially stabilising phenyl substituent.4, 5

**Scheme 6:**
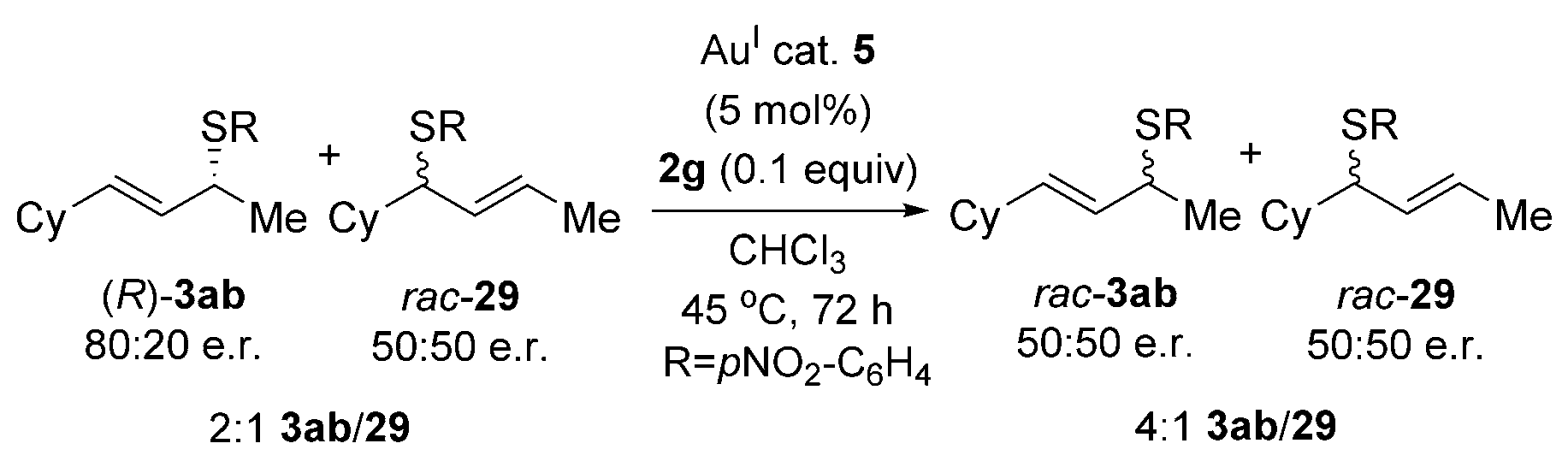
Racemisation and isomerisation study of products under the gold(I)-catalysed thioetherification reaction conditions.

The computational studies point towards accessible isomerisations between the two possible allylic thioether products (formal S_N_2′ or S_N_2 regioselectivities) and that any regioselectivity is ultimately dictated by the thermodynamic stability of the products. With this in mind, we postulated that the apparent failure of enantioenriched (*S*)-**18**, (*R*)-**21** and (*R*)-**20** to transfer chirality to its respective products is due to the subsequent racemisation of any enantioenriched product that might be formed (via path 3 in Scheme 3). To gain experimental proof for these hypotheses, a racemisation/isomerisation experiment was carried out (Scheme 6). A mixture of **3 ab** (which would be the formal S_N_2′ product in Entry 14, Table 3) and **29** (which would be the formal S_N_2 product in entry 14, Table 3) was prepared using an alternative three-step procedure by Gais;[29] this produced an inseparable mixture of (*R*)-**3 ab** in 80:20 e.r. and *rac*-**29** in a 2:1 ratio. This 2:1 mixture was then subjected to the gold(I) reaction conditions (Scheme 6). Indeed, not only is the enantioenriched (*R*)-**3 ab** completely racemised but the ratio of **3 ab**/**29** also increases from 2:1 to 4:1, suggesting equilibration towards the more stable isomer.[30] Therefore, path 1 followed by path 3 is likely the reason for the observed racemised products, rather than path 2 (Scheme 3).

## Conclusion

In conclusion, a mild, regioselective (formal S_N_2′) and stereoselective (*E*) gold(I)-catalysed direct thioetherification of allylic alcohols has been developed. The reaction has a wide substrate scope including tertiary, secondary and certain primary allylic alcohols, and tolerates a wide range of substituents on the thiol nucleophile. Unlike the related etherification reaction employing alcohol nucleophiles, the thiol nucleophile does not need to be in excess; an approximately 1:1 ratio of substrates is sufficient with no observed self-reaction of the allylic alcohol substrate. Furthermore, the S_N_2′ regioselectivity is generally excellent. The exception to this is when the γ-position of the allylic alcohol is fully substituted. When alkyl thiols are used as nucleophiles, the resulting products are prone to oxidation to the sulfone during silica gel chromatography, but this is successfully avoided by employing a gold-scavenging resin Reaxa QuadraPure™ MPA prior to purification. Computational studies on selected substrates suggest that both the formal S_N_2′ and S_N_2 products can be readily and reversibly formed under the reaction conditions. The observed regioselectivities are therefore dictated by the thermodynamic stabilities of the products. The observation of any formal S_N_2 products will be the result of two sequential S_N_2′ steps, this route being kinetically favoured over a direct S_N_2 reaction. The lack of chirality transfer in enantioenriched substrates can also be accounted for by the racemisation of products.

## Experimental Section

### General procedure

Thiol **2** (1.1 equiv) and catalyst **5** (5 mol %) were added to a solution of allylic alcohol (1 equiv) in chloroform (0.4 m). The resulting mixture was allowed to stir at 35–60 °C for 24–72 h as required. The mixture was then filtered through a plug of silica using diethyl ether as eluent. The filtrate was concentrated under reduced pressure and the crude material was purified by flash column chromatography. Full experimental procedures, characterisation for all new compounds and copies of ^1^H and ^13^C NMR spectra are provided in the Supporting Information.

## Supporting Information

As a service to our authors and readers, this journal provides supporting information supplied by the authors. Such materials are peer reviewed and may be re-organized for online delivery, but are not copy-edited or typeset. Technical support issues arising from supporting information (other than missing files) should be addressed to the authors.

miscellaneous_information
