# Supplementary material for: Gold(I)-Catalysed Direct Thioetherifications Using Allylic Alcohols: an Experimental and Computational Study
Source: Chemistry. 2014 Jul 30;20(36):11540–8. doi: 10.1002/chem.201403293 (PMC4517163; doi:10.1002/chem.201403293)
Supplement: Supplementary file 1 — miscellaneous_information [file chem0020-11540-sd1.pdf]

CHEMISTRY
A European Journal

Supporting Information

© Copyright Wiley-VCH Verlag GmbH & Co. KGaA, 69451 Weinheim, 2014

Gold(I)-Catalysed Direct Thioetherifications Using Allylic Alcohols: an Experimental and Computational Study

Lorena Herkert, Samantha L. J. Green, Graeme Barker, David G. Johnson, Paul C. Young, Stuart A. Macgregor,* and Ai-Lan Lee*[a]

chem_201403293_sm_miscellaneous_information.pdf

Table of contents

Experimental Supporting Information: Pages 3-72

1. General Experimental Section pg 3

2. Representative Optimisation Studies pg 4

3. Experimental Procedures pg 7

4. ^{1}H NMR and ^{13}C NMR Spectra of Synthesised Compounds pg 37

5. References for Experimental Section pg 72

Computational Supporting Information: Pages 73 -188

1. Computational Details and References pg 73

2. Reactions of Substrate **4'** pg 75

3. Reactions of Substrate **24** pg 103

4. Reactions of Substrate **4'** with PhSH at $[(Johnphos)Au(NCMe)]^{+}$, **5**. pg 127

5. Reactions of **9**, **10** and **11** with PhSH pg 149

6. Reactions of Substrate **17** pg 162

1) General Experimental Section

^1H NMR spectra was recorded on Bruker AV 300 and AV 400 spectrometers at 300 and 400 MHz respectively and referenced to residual solvent. ^{13}C NMR spectra were recorded using the same spectrometers at 75 and 100 MHz respectively. Chemical shift data are quoted in parts per million (ppm) and are referenced to tetramethylsilane (TMS) or to residual solvent peaks ($CDCl_3$ at δ_H 7.26). *J* values are given in Hz and s, d, dd, dt, t, q and m abbreviations correspond to singlet, doublet, doublet of doublet, doublet of triplet, triplet, quartet and multiplet. Mass spectra were obtained at the EPSRC National Mass Spectrometry Service Centre in Swansea and APCI represents atmospheric pressure chemical ionisation. Infrared spectra were obtained on Perkin-Elmer Spectrum 100 FT-IR Universal ATR Sampling Accessory, deposited neat to a diamond/ZnSe plate. Flash column chromatography was carried out using Matrix silica gel 60 from Fisher Chemicals and TLC was performed using Merck silica gel 60 F254 pre-coated sheets and visualised by UV (254 nm) or stained by the use of aqueous acidic $KMnO_4$ or aqueous acidic ammonium molybdate as appropriate. Petrol ether refers to petroleum ether (40-60 °C). Chemicals were purchased from Sigma-Aldrich, Acros, Fisher and Apollo chemical companies and used without further purification. Tetrahydrofuran was dried by distillation from sodium – benzophenone under nitrogen or using an MBRAUN SPS-800 solvent purification system. High performance liquid chromatography (HPLC) was done on Agilent Technologies 1120 Compact LC.

Gold catalyst **5** was purchased from Sigma-Aldrich and used without further purification. All thiophenols and thiols were purchased from Sigma-Aldrich, Fisher or Apollo and used without further purification. Scavenger QuadraPure(TM) MPA (100-400 mum particle size, extent of labeling: 1.5 mmol/g loading, 1% cross-linked with divinylbenzene) was purchased from Sigma-Aldrich.

The gold(I)-catalysed reactions were carried out in screw cap 1 dram vials unless otherwise indicated. No special precautions to exclude air or moisture were taken unless otherwise indicated.

2) Representative Optimization Studies

Below are representative screens that were carried out in order to optimise the reaction conditions.

General procedure for optimization studies

The catalyst **5** was added to a solution of allylic alcohol **4** and thiol **2a** in solvent at the required temperature, the vial was sealed and the resulting mixture was stirred for the required time. The solution was filtered through a plug of silica with diethyl ether followed by concentration under reduced pressure. The crude mixture was analysed by ^{1}H NMR to give the following results:

Temperature Screen:

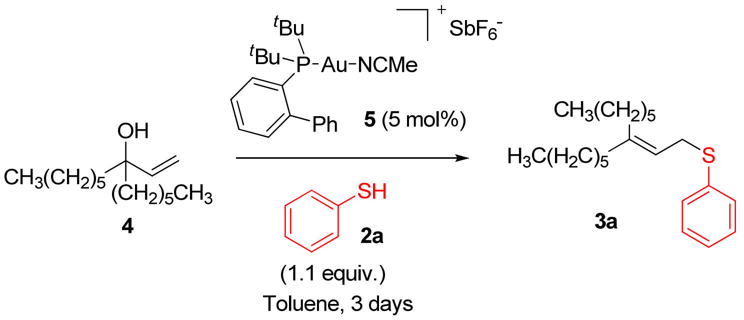

Entry	Temperature	Conversion to 3a
1	30 °C	46%
2	40 °C	88%

Reaction Time Screen:

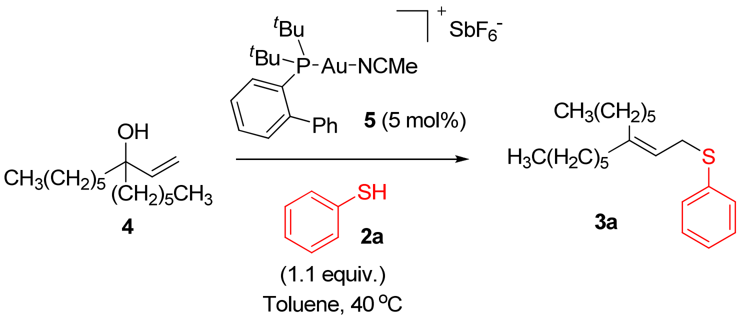

Entry	Time	Conversion to 3a
1	8 hours	67%
2	16 hours	81%
3	2 days	82%
4	3 days	88%

Concentration Screen:

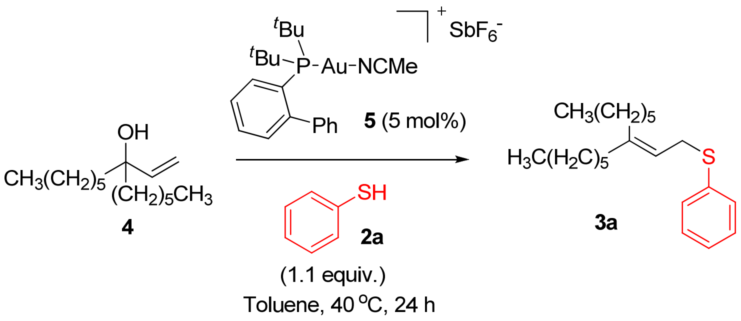

Entry	Concentration (mol L^{-1})	Conversion to 3a	Conversion to unidentified side products
1	0.621	72%	24%
2	0.386	72%	15%
3	0.199	74%	16%
4	0.0996	78%	11%
5	0.0498	65%	24%

Solvent Screen:

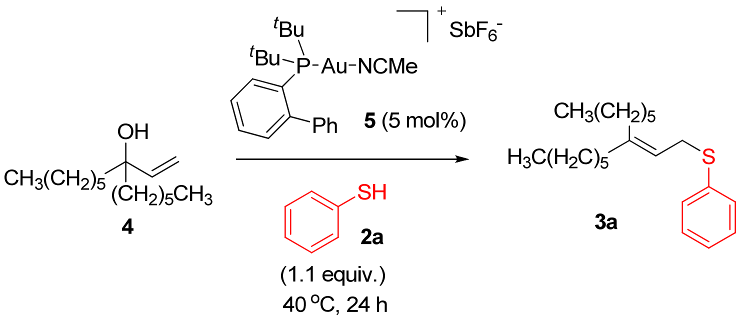

Entry	Solvent	Conversion to 3a
1	Toluene	72%
2	$CHCl_3$	92%
3	1,2-dichloroethane	90%

$CHCl_3$ was therefore used as the optimal solvent, as the solvent had changed another temperature screen was carried out.

Temperature Screen:

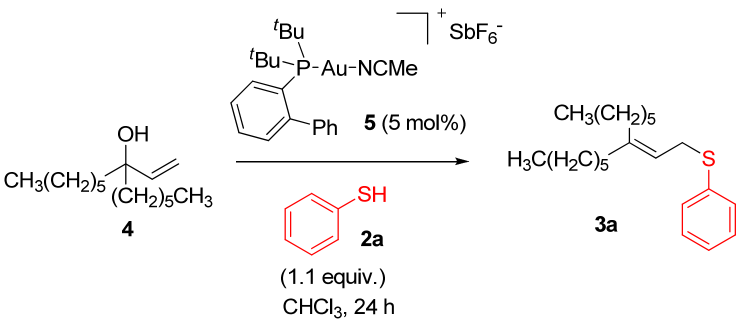

Entry	Temperature	Conversion to 3a
1	30 °C	79%
2	35 °C	91%
3	40 °C	92%

35 °C was therefore deemed sufficient for good conversion.

3) Experimental procedures

Synthesis of starting materials

Allylic alcohol **16** and **24** were purchased from Sigma-Aldrich. All other allylic alcohol substrates were prepared following known literature procedures. Allylic alcohols **4**,[1] **8**,[1] **9**,[2] **10**,[2] **11**,[3] **12**,[4] **17**[5] were obtained following known literature procedure using vinyl Grignard addition to ketones/aldehydes.[3, 6] **13-15**, **19**,[7] **20**,[8] **21**[9] were prepared by ^{n}BuLi, ^{t}BuLi or CyLi addition to the corresponding enone or enal as appropriate.[10] **18[9]** was prepared by PhMgBr addition to the crotonaldehyde. **22**[11] and **23**[12] were prepared by partial reduction of the corresponding alkynes. **26**[13] was prepared from reduction of the corresponding enone.[1, 13]

Characterization for **13-15** is given below.

4-Butyl-oct-2-ene-4-ol (13)

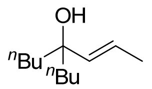

13

To a solution of ethyl-*trans*-2-butenoate (1.09 mL, 8.76 mmol) in diethyl ether (16 mL) *n*-BuLi (14.6 mL, 23.4 mmol) was added dropwise at 0 °C over 30 minutes. After stirring at 0 °C for 3 h the reaction mixture was quenched with saturated NH_4Cl (35 mL) and allowed to warm to room temperature. The layers were separated and the water layer was extracted with diethyl ether (3 × 50 mL). The combined organic layers were washed with brine (2 × 50 mL) and dried over Na_2SO_4. The solvent was removed under reduced pressure. The crude was purified through flash column chromatography in hexane:diethyl ether (5:1) to yield the titled compound **13** as a colourless oil (1.02 g, 3.50 mmol, 40 %). ν_{max} / cm^{-1} 3453 (br), 2956, 2929, 2861, 1467, 1456, 1377, 1253, 1141, 1027, 999, 970, 903, 799, 730. ^{1}H NMR (300 MHz, $CDCl_3$) δ 5.51 (dq, *J* = 15.5, 6.3 Hz, 1H, $CH_3C\underline{H}$=), 5.35 (dq, *J* = 15.5, 1.4 Hz, 1H, CH_3CH=$C\underline{H}$), 1.62 (dd, *J* = 6.3, 1.4 Hz, 3H, $C\underline{H}_3CH$=), 1.57 – 1.12 (m, 12H, $C\underline{H}_2$), 0.81 (t, *J* = 6.8 Hz, 6H, $C\underline{H}_3$). ^{13}C NMR (75 MHz, $CDCl_3$) δ 137.3 (CH), 122.7 (CH), 74.8 (C), 40.8 (CH_2), 25.8 (CH_2), 23.3 (CH_2), 17.7 (CH_3), 14.1 (CH_3). Found (ASAP): $[M]^+$ 183.741. $C_{12}H_{23}O_1$ requires 183.1743.

(*E*)-2,2,3-Trimethylhex-4-en-3-ol (14)

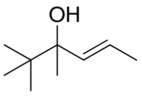

14

A solution of ${}^tBuLi$ (8.13 mL of a 1.9 M solution in hexanes, 15.45 mmol) was added dropwise to a stirred solution of (*E*)-3-penten-2-one (1 g, 1.16 mL, 11.89 mmol) in THF (10 mL) at 0 °C under Ar. The resulting solution was stirred at 0 °C for 30 min then warmed to rt. Then, a saturated solution of NH_4Cl (aq) (15 mL) was added, and the two layers were separated. The aqueous layer was extracted with Et_2O (3 × 20 mL). The combined organic layers were dried ($MgSO_4$) and evaporated under reduced pressure to give the crude product. Purification by flash column chromatography on silica with 9:1 petrol-Et_2O as eluent gave trimethylhexenol **14** (143 mg, 8%) as a colourless oil. ν_{max}/cm^{-1} 3456 (br), 2957, 2926, 2871, 1463, 1375, 1215, 755 cm^{-1}; ${}^1H$ NMR (300 MHz, $CDCl_3$) δ_H 5.69 (1H, dq, *J* = 15.6, 0.8 Hz, =CHC), 5.60 (1H, dq, *J* = 15.6, 5.9 Hz, MeC$\underline{H}$), 1.70 (3H, dd, *J* = 5.9, 0.8 Hz, =CH$\underline{Me}$), 1.34 (1H, br s, OH), 1.21 (3H, s, Me), 0.91 (9H, s, CMe_3); ${}^{13}C$ NMR (75 MHz, $CDCl_3$) δ_C 136.2 (HC), 123.2 (HC), 77.0 (C), 37.5 (C), 25.5 (CH_3), 23.6 (CH_3), 17.9 (CH_3); Found (APCI): $[M - H]^+$ 141.1271, $C_9H_{17}O$ requires 141.1274.

2-Methyl-3-butyl-hept-1-en-3-ol (15)

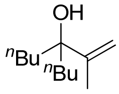

15

To a solution of methylmetacrylate (1.1 mL, 9.98 mmol) in dry diethyl ether (20 mL) *n*-BuLi (15 mL, 23.97 mmol) was added dropwise at 0 °C over 0.5 h. After stirring at 0 °C for a further 18 h the reaction mixture was quenched with saturated NH_4Cl (aq.) (20 mL). The layers were separated and the aqueous layer was extracted with diethyl ether (3 × 50 mL). The combined organic layers were washed with brine and dried over Na_2SO_4. The solution was concentrated under reduced pressure and purified with flash column chromatography (hexane: diethyl ether 10:1) to yield the titled compound **15** as acolourless oil (132.3 mg, 0.72 mmol, 7 %). ν_{max} / cm^{-1} 3484 (br), 2956, 2936, 2861, 1643, 1457, 1378, 1257, 1143, 1044, 989, 897, 793, 731. ${}^1H$ NMR (300 MHz, $CDCl_3$) δ 4.89 (s, 1H, =C$\underline{H}$H), 4.82 (s, 1H, =CH$\underline{H}$), 1.62 (s, 3H, C$\underline{H}_3$C=CH_2), 1.53 – 1.01 (m, 12H, CH_2), 0.83 (t, *J* = 6.9 Hz, 6H, CH_3). ${}^{13}C$ NMR (75 MHz, $CDCl_3$) δ 148.6 (C), 110.4 (CH_2), 77.7 (C), 39.2 (CH_2), 25.3 (CH_2), 23.1 (CH_2), 19.7 (CH_3), 14.1 (CH_3). Found (ASAP): [M]+ 184.1821. $C_{12}H_{24}O_1$ requires 184.1822.

General procedure A for 3a-3i (thiophenol nucleophiles):

Thiophenol **2** (1.1 equiv.) and **5** (5 mol%) were added to a solution of allylic alcohol **4** (1 equiv.) in chloroform (0.386 M) in a 1 dram vial. The reaction mixture was allowed to stir at 35 °C for 24 hours. The mixture was then filtered through a plug of silica with diethyl ether. The filtrate was concentrated under reduced pressure. Purification of the crude material was carried out by flash column chromatography.

3-Hexanon-2-en-1-yl phenyl sulfide (3a):

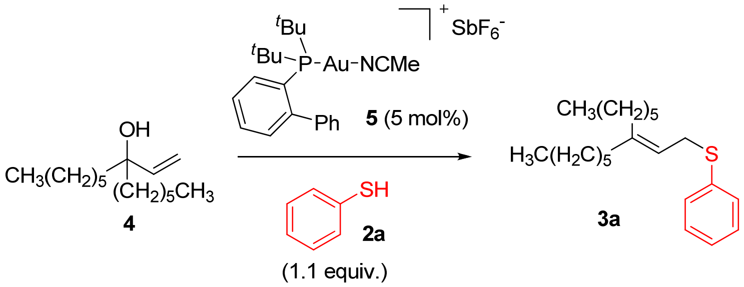

General procedure A. Purification of the crude product by column chromatography (pure petrol ether) yielded the title compound **3a** (17.3 mg, 0.054 mmol, 70%) as a pale yellow liquid.

ν_{max}/cm^{-1} 3060, 2960, 2853, 1580, 1439, 1024, 688; δ_H (300 MHz, $CDCl_3$) 7.46 – 7.04 (5H, m, Ar-H), 5.20 (1H, t, J = 7.7 Hz, C=CH), 3.49 (2H, d, J = 7.7 Hz, SCH_2), 1.95 – 1.84 (4H, m, $C=CCH_2$), 1.32 – 1.10 (16H, m, Alkyl-H), 0.86 – 0.76 (6H, m, Alkyl-CH_3); δ_c (75 MHz, $CDCl_3$) 144.8 (C), 137.1 (C), 129.8 (CH), 128.8 (CH), 126.1 (CH), 119.1 (CH), 36.9 (CH_2), 32.2 (CH_2), 31.9 (CH_2), 30.2 (CH_2), 29.6 (CH_2), 29.2 (CH_2), 28.6 (CH_2), 28.1 (CH_2), 22.8 ($2CH_2$), 14.3 ($2CH_3$); Found (APCI): $[M + H]^+$ 319.2454, $C_{21}H_{35}S$ requires 319.2454.

3-Hexanon-2-en-1-yl 2-methylphenyl sulfide (3b):

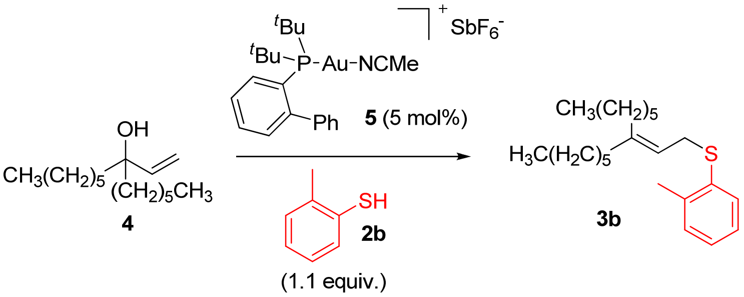

General procedure A. Purification of the crude product by column chromatography (pure petrol ether) yielded the title compound **3b** (15.2 mg, 0.046 mmol, 62%) as a colourless liquid.

ν_{max}/cm^{-1} 2955, 2925-2855, 1589, 1466, 1065, 740; δ_H (300 MHz, $CDCl_3$) 7.20 – 7.04 (4H, m, Ar-H), 5.22 (1H, t, *J* = 7.7 Hz, C=CH), 3.45 (2H, d, *J* = 7.7 Hz, SCH_2), 2.29 (3H, s, Aryl-CH_3), 1.98-1.85 (4H, m, CH_2=CH(CH_2Alkyl)), 1.33-1.10 (16H, m, Alkyl-H), 0.81 (6H, t, *J* = 6.6, Alkyl-CH_3); δ_c (75 MHz, $CDCl_3$) 145.1 (C), 137.7 (C), 136.6 (C), 130.1 (CH), 128.7 (CH), 126.4 (CH), 125.8 (CH), 118.8 (CH), 36.9 (CH_2), 31.9 (CH_2), 31.3 (CH_2), 30.3 (CH_2), 29.9 (CH_2), 29.6 (CH_2), 29.3 (CH_2), 29.1 (CH_2), 28.6 (CH_2), 28.1 (CH_2), 22.8 (CH_2), 20.5 (CH_3), 14.3 ($2CH_3$); Found (APCI): $[M + H]^+$ 333.2608, $C_{22}H_{37}S$ requires 333.2610.

3-Hexanon-2-en-1-yl 3-methylphenyl sulfide (3c):

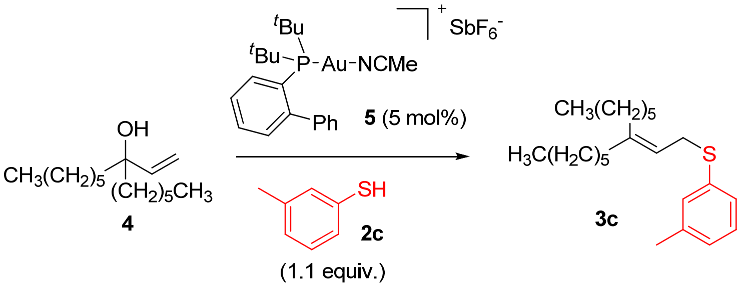

General procedure A. Purification of the crude product by column chromatography (pure petrol ether) yielded the title compound **3c** (17.2 mg, 0.052 mmol, 76%) as a colourless liquid.

ν_{max}/cm^{-1} 2955, 2925, 2855, 1592, 1466, 1081, 770, 688; δ_H (300 MHz, $CDCl_3$) 7.10 – 6.87 (4H, m, Ar-H), 5.20 (1H, t, *J* = 7.6 Hz, C=CH), 3.4 (2H, d, *J* = 7.6 Hz, SCH_2), 2.24 (3H, s, Aryl-CH_3), 1.91 (4H, t, *J* = 7.2 Hz, CH_2=CH(CH_2Alkyl), 1.43 – 1.23 (16H, m, Alkyl-H), 0.81 (6H, t, *J* = 6.7, Alkyl-CH_3); δ_c (75 MHz, $CDCl_3$) 144.7 (C), 138.6 (C), 136.7 (C), 130.4 (CH), 128.7 (CH), 126.9 (CH), 126.7 (CH), 119.2 (CH), 36.9 (CH_2), 32.1 (CH_2), 31.9 ($2CH_2$), 30.2 (CH_2), 29.6 (CH_2), 29.2 (CH_2), 28.6 (CH_2), 28.2 (CH_2), 22.8 ($2CH_2$), 21.5 (CH_3), 14.3 ($2CH_3$); Found (APCI): $[M + H]^+$ 333.2608, $C_{22}H_{37}S$ requires 333.2610.

3-Hexanon-2-en-1-yl 4-methylphenyl sulfide (3d):

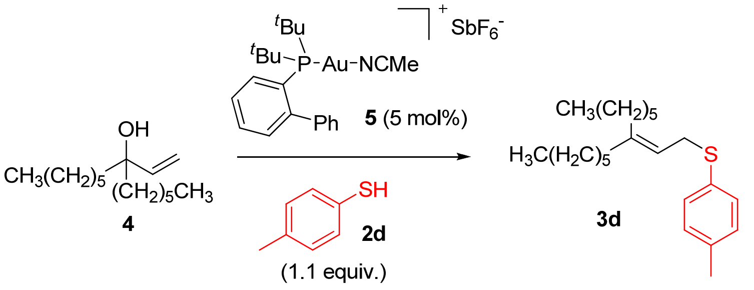

General procedure A. Purification of the crude product by column chromatography (pure petrol ether) yielded the title compound **3d** (19.4 mg, 0.058 mmol, 71%) as a pale yellow liquid.

ν_{max}/cm^{-1} 2955, 2926, 2856, 1491, 1092, 802; δ_H (300 MHz, CDCl$_3$) 7.29 (2H, d, J = 7.8 Hz, Ar-H), 7.11 (2H, d, J = 7.8 Hz, Ar-H), 5.19 (1H, t, J = 7.7 Hz, C=CH), 3.44 (2H, d, J = 7.7 Hz, SCH$_2$), 2.24 (3H, s, Aryl-CH$_3$), 1.94-1.82 (4H, m, CH$_2$=CH(CH$_2$Alkyl), 1.32-1.08 (16H, m, Alkyl-H), 0.81 (6H, t, J = 6.7, Alkyl-CH$_3$); δ_c (75 MHz, CDCl$_3$) 144.5 (C), 136.3 (C), 133.2 (C), 130.73 (CH), 129.9 (CH), 129.6 (CH), 128.7 (CH), 119.4 (CH), 36.9 (CH$_2$), 32.9 (CH$_2$), 31.9 (2CH$_2$), 30.2 (CH$_2$), 29.6 (CH$_2$), 29.2 (CH$_2$), 28.6 (CH$_2$), 28.2 (CH$_2$), 22.8 (2CH$_2$), 21.2 (CH$_3$), 14.3 (2CH$_3$); Found (APCI): [M]$^+$ 332.2530, C$_{22}$H$_{36}$S requires 332.2532.

1-[(3-Hexanon-2-en-1-yl)sulfanyl]-2-methoxybenzene (3e):

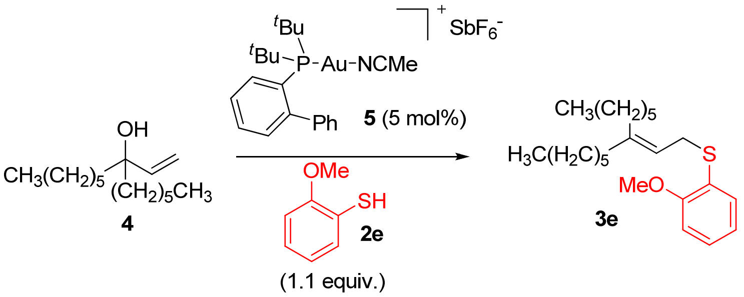

General procedure A. Purification of the crude product by column chromatography (pure petrol ether to 100:1 petrol ether-diethyl ether) yielded the title compound **3e** (15.7 mg, 0.045 mmol, 66%) as a colourless liquid.

ν_{max}/cm^{-1} 2955, 2925, 2855, 1577, 1463, 1243, 1044, 743; δ_H (300 MHz, CDCl$_3$) 7.21 (1H, td, J = 7.9, 1.6 Hz, Ar-H), 7.11 (1H, td, J = 7.9, 1.6 Hz, Ar-H), 6.83 (1H, td, J = 7.5, 1.6 Hz, Ar-H), 6.76 (1H, dd, J = 9.7, 5.7 Hz, Ar-H), 5.20 (1H, t, J = 7.7 Hz, C=CH), 3.82 (3H, s, OCH$_3$), 3.47 (2H, d, J = 7.7 Hz, SCH$_2$), 1.96-1.85 (4H, m, CH$_2$=CH(CH$_2$Alkyl), 1.31-1.11 (16H, m, Alkyl-H), 0.85-0.77 (6H, m, Alkyl-CH$_3$); δ_c (75 MHz, CDCl$_3$) 157.6 (C), 144.9 (C), 130.3 (CH), 127.2 (CH), 125.2 (C), 121.0 (CH), 119.1 (CH), 110.4 (CH), 55.9 (CH$_3$), 36.9 (CH$_2$), 31.9 (2CH$_2$), 30.4 (CH$_2$), 30.2 (CH$_2$), 29.7 (CH$_2$), 29.2 (CH$_2$), 28.7 (CH$_2$), 28.8 (CH$_2$), 22.81 (CH$_2$), 22.77 (CH$_2$), 14.3 (2CH$_3$); Found (APCI): [M + H]$^+$ 349.2558, C$_{22}$H$_{37}$OS requires 349.2560.

1-[(3-Hexanon-2-en-1-yl)sulfanyl]-4-methoxybenzene (3f):

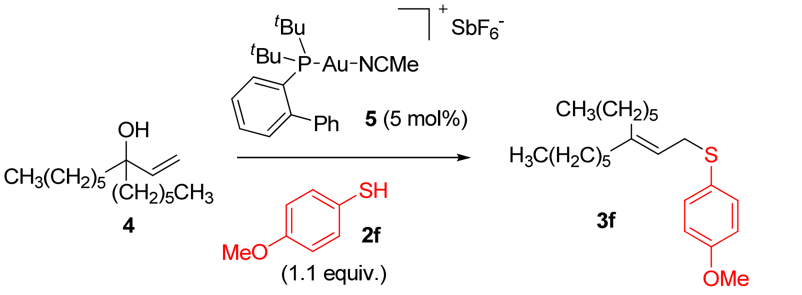

General procedure A. Purification of the crude product by column chromatography (pure petrol ether to 100:1 petrol ether-diethyl ether) yielded the title compound **3f** (15.3 mg, 0.044 mmol, 64%) as a colourless liquid.

ν_{max}/cm^{-1} 2955, 2927, 2856, 1592, 1493, 1245, 1036, 825; δ_H (300 MHz, $CDCl_3$) 7.28 (2H, d, J = 8.8 Hz, Aryl-H), 6.75 (2H, d, J = 8.8 Hz, Aryl-H), 5.17 (1H, t, J = 7.8 Hz, C=CH), 3.72 (3H, s, OCH_3), 3.38 (2H, d, J = 7.8 Hz, SCH_2), 1.92 – 1.84 (2H, m, $C{=}CCH_2$), 1.84 – 1.75 (2H, m, $C{=}CCH_2$), 1.32 – 1.08 (16H, m, Alkyl-H), 0.87 – 0.75 (6H, m, Alkyl-CH_3); δ_c (100 MHz, $CDCl_3$) 159.1 (C), 144.3 (C), 134.1 (CH), 126.8 (C), 119.8 (CH), 114.5 (CH), 55.5 (CH_3), 36.9 (CH_2), 34.3 (CH_2), 31.94 (CH_2), 31.91(CH_2), 30.1 (CH_2), 29.6 (CH_2), 29.2 (CH_2), 28.6 (CH_2), 28.2 (CH_2), 22.8 ($2CH_2$), 14.27 (CH_3), 14.26 (CH_3); Found (APCI): $[M + H]^+$ 349.2551, $C_{22}H_{37}OS$ requires 349.2560.

3-Hexanon-2-en-1-yl 4-nitrophenyl sulfide (3g):

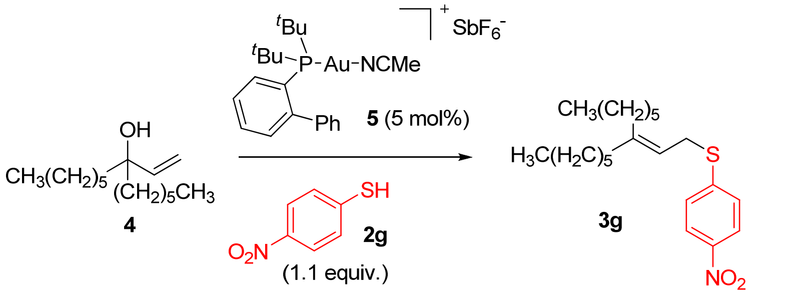

General procedure A. Purification of the crude product by column chromatography (pure petrol ether to 100:1 petrol ether-diethyl ether) yielded the title compound **3g** (18.3 mg, 0.050 mmol, 68%) as a yellow liquid.

ν_{max}/cm^{-1} 2956, 2928, 2857, 1579, 1514, 1337, 853; δ_H (300 MHz, $CDCl_3$) 8.04 (2H, d, J = 9.0 Hz Ar-H), 7.25 (2H, d, J = 9.0 Hz, Ar-H), 5.20 (1H, t, J = 7.5 Hz, C=CH), 3.60 (2H, d, J = 7.5 Hz, SCH_2), 2.07 – 1.90 (4H, m, CH_2=CH(CH_2Alkyl)), 1.38-1.12 (16H, m, Alkyl-H), 0.90-0.72 (6H, m, Alkyl-CH_3); δ_c (75 MHz, $CDCl_3$) 148.4 (C), 146.7 (C), 145.1 (C), 126.5 (CH), 124.0 (CH), 117.3 (CH), 36.9 (CH_2), 31.90 (CH_2), 31.86 (CH_2), 30.5 (CH_2), 30.4 (CH_2), 29.6 (CH_2), 29.1 (CH_2), 28.6 (CH_2), 28.0 (CH_2), 22.8 ($2CH_2$), 14.2 ($2CH_3$); Found (APCI): $[M + H]^+$ 364.2301, $C_{21}H_{34}O_2NS$ requires 364.2305.

1-Bromo-4-[(3-hexanon-2-en-1-yl)sulfanyl]benzene (3h):

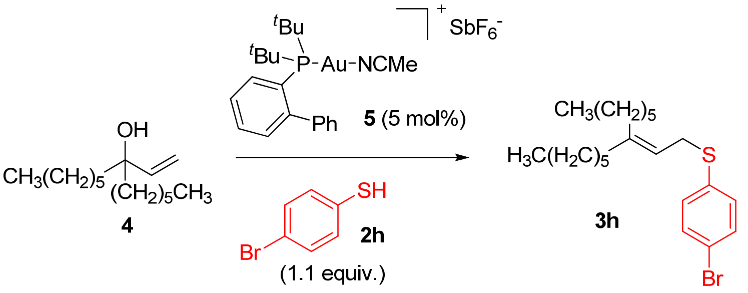

General procedure A. Purification of the crude product by column chromatography (pure petrol ether) yielded the title compound **3h** (20.2 mg, 0.051 mmol, 73%) as a colourless liquid.

ν_{max}/cm^{-1} 2955, 2925, 2855, 1472, 1069, 1008, 740; δ_H (300 MHz, $CDCl_3$) 7.31 (2H, d, *J* = 8.5 Hz, Ar-H), 7.12 (2H, d, *J* = 8.5 Hz, Ar-H), 5.17 (1H, t, *J* = 7.7 Hz, C=CH), 3.46 (2H, d, *J* = 7.7 Hz, SCH_2), 1.84-1.86 (4H, m, CH_2=CH(CH_2Alkyl), 1.30-1.13 (16H, m, Alkyl-H), 0.81 (6H, t, *J* = 5.4, Alkyl-CH_3); δ_c (75 MHz, $CDCl_3$) 145.2 (C), 136.3 (C), 131.9 (CH), 131.4 (CH), 120.0 (C), 118.8 (CH), 36.9 (CH_2), 32.3 (CH_2), 31.9 ($2CH_2$), 30.3 (CH_2), 29.6 (CH_2), 29.2 (CH_2), 28.6 (CH_2), 28.1 (CH_2), 22.8 ($2CH_2$), 14.3 ($2CH_3$); Found (APCI): $[M + H]^+$ 397.1555, $C_{21}H_{34}BrS$ requires 397.1559.

4-[(3-Hexanon-2-en-1-yl)sulfanyl]phenol (3i):

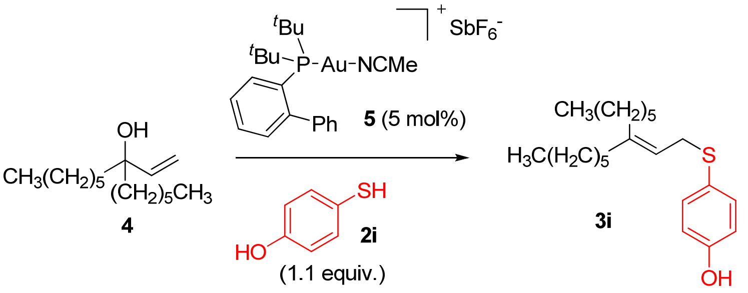

General procedure A. Purification of the crude product by column chromatography (pure hexane to 5:1 hexane-diethyl ether) yielded the title compound **3i** (11.8 mg, 0.035 mmol, 49%) as a pale yellow liquid.

ν_{max}/cm^{-1} 3363, 2955, 2926, 2856, 1493, 1215, 828; δ_H (300 MHz, $CDCl_3$) 7.28 – 7.15 (2H, d, *J* = 8.6 Hz, Ar-H), 6.73 -6.62 (2H, d, *J* = 8.6 Hz, Ar-H), 5.16 (1H, t, *J* = 7.8 Hz, C=CH), 4.59 (1H, s, OH), 3.37 (2H, d, *J* = 7.8 Hz, SCH_2), 1.93-1.74 (4H, m, CH_2=CH(CH_2Alkyl), 1.31-1.09 (16H, m, Alkyl-H), 0.88-0.74 (6H, m, Alkyl-CH_3); δ_c (75 MHz, $CDCl_3$) 155.0 (C), 144.4 (C), 134.3 (CH), 127.1 (C), 119.7 (CH), 115.9 (CH), 36.9 (CH_2), 34.3 (CH_2), 31.93 (CH_2), 31.90 (CH_2), 30.1 (CH_2), 29.6 (CH_2), 29.2 (CH_2), 28.6 (CH_2), 28.2 (CH_2), 22.8 ($2CH_2$), 14.3 ($2CH_3$); Found (APCI): $[M + H]^+$ 335.2403, $C_{21}H_{35}OS$ requires 335.2398.

Benzyl 3-hexanon-2-en-1-yl sulfone (7j):

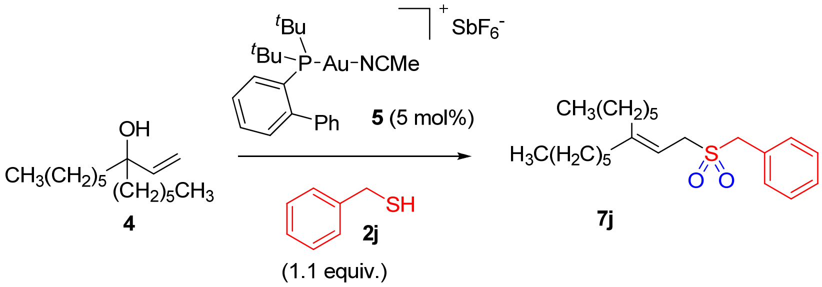

A solution of benzyl mercaptan **2j** (8.9 μL, 0.0688 mmol) and catalyst **5** (2.6 mg, 0.0033 mmol) in $CHCl_3$ (0.16 mL), was added to a solution of 3-hexyl-1-nonen-3-ol **4** (15.5 mg, 0.068 mmol) at 50 °C and stirred for 3 days. The solution was then filtered through a silica plug with diethyl ether and concentrated under reduced pressure. Purification of the crude product by column chromatography (pure petrol ether to 5:1 petrol ether:diethyl ether) yielded the title compound **7j** (10.5 mg, 0.029 mmol, 42%) as a colourless liquid.

ν_{max}/cm^{-1} 2927, 2857, 1655, 1456, 1313, 1117, 698; δ_H (300 MHz, $CDCl_3$) 7.32 (5H, m, Ar-H), 5.21 (1H, t, J = 7.6 Hz, C=CH), 4.10 (2H, s, SCH_2Ar), 3.53 (2H, d, J = 7.6 Hz, SCH_2CH), 2.06- 1.85 (4H, m, CH_2=CH(CH_2Alkyl)), 1.30-1.09 (16H, m, Alkyl-H), 0.89-0.73 (6H, m, Alkyl-CH_3); δ_c (75 MHz, $CDCl_3$) 150.9 (C), 130.8 (CH), 129.17 (CH), 129.10 (CH), 128.0 (C), 109.7 (CH), 58.2 (CH_2), 51.6 (CH_2), 37.2 (CH_2), 31.9 (CH_2), 31.8 (CH_2), 30.9 (CH_2), 29.5 (CH_2), 29.2 (CH_2), 28.3 (CH_2), 22.0 (CH_2), 22.79 (CH_2), 22.74 (CH_2), 14.2 ($2CH_3$); Found (APCI): $[M + H]^+$ 365.2507, $C_{22}H_{37}O_2S$ requires 365.2509.

General procedure B for 3j-3o (alkyl thiols as nucleophiles):

4-Nitrothiophenol (1.1 equiv.) and **5** (5 mol%) were added to a solution of allylic alcohol (1 equiv.) in chloroform (0.386 M) in a 1 dram vial. The vial was capped and the solution allowed to stir for 72 h (for temperatures see below), after which it was allowed to cool down to room temperature. Scavenger Reaxa QuadraPure^{TM} MPA (0.5 equiv.) was added and the mixture was stirred gently for 3 h. The reaction mixture was then filtered through a plug of cotton wool with diethyl ether. The filtrate was concentrated under reduced pressure. Purification of the crude material was carried out by flash column chromatography.

Benzyl 3-hexanon-2-en-1-yl sulfide (3j):

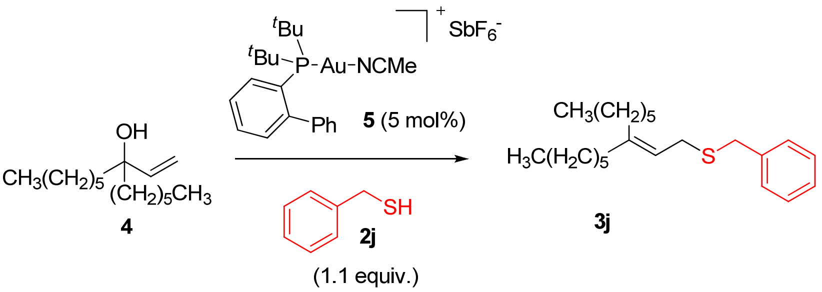

The reaction was carried out at 50 °C following general procedure B. The product was purified using flash column chromatography using neat hexane. Product **3j** was obtained as colourless liquid (12.3 mg, 37.0 µmol, 56%).

ν_{max}/cm^{-1} 2955, 2925, 2855, 1603, 1494, 1454, 1378, 1232, 1071, 767, 717, 697; δ_H (300 MHz, $CDCl_3$) 7.34-7.19 (5 H, m, Ar-H), 5.21 (1 H, t, J = 7.6 Hz, C=CH), 3.69 (2 H, s, SCH_2Ph), 3.07 (2 H, d, J = 7.6 Hz, C=CHCH$_2$), 2.09-1.83 (4 H, m, HC=CCH$_2$), 1.46-1.11 (16 H, m, alkyl CH$_2$), 0.99-0.71 (6 H, m, CH$_3$); δ_C (75 MHz, $CDCl_3$) 144.2 (C), 138.8 (C), 129.0 (CH), 128.6 (CH), 127.0 (CH), 120.0 (CH), 37.0 (CH_2), 35.9 (CH_2), 31.9 (CH_2), 31.9 (CH_2), 30.3 (CH_2), 29.6 (CH_2), 29.3 (CH_2), 29.1 (CH_2), 28.7 (CH_2), 28.3 (CH_2), 22.81 (CH_2), 22.79 (CH_2), 14.3 (2x CH_3); Found (APCI): $[M+H]^+$ 333.2607, $C_{22}H_{37}S$ requires 333.2610.

Butyl(3-hexylnon-2-en-1-yl)sulfide (3k)

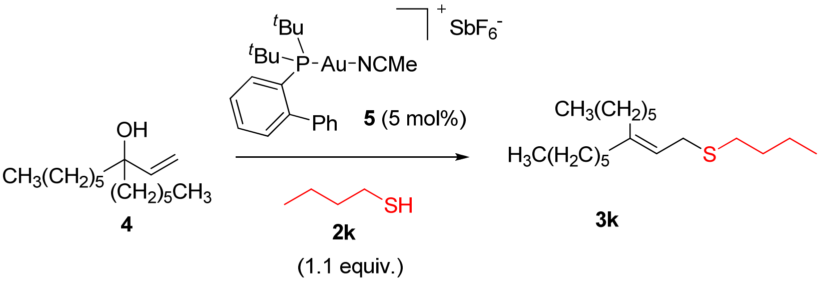

The reaction was carried out at 60 °C following general procedure B. The product was purified using flash column chromatography using neat hexane. Product **3k** was obtained as colourless liquid (9.40 mg, 31.5 µmol, 47 %).

ν_{max}/cm^{-1} 2956, 2925, 2856, 1465, 1378, 1221, 724; δ_H (300 MHz, $CDCl_3$) 5.20 (1 H, t, J = 7.7 Hz, C=C$\underline{H}$), 3.15 (2 H, d, J = 7.7 Hz, C=CHC$\underline{H}_2$), 2.46 (2 H, m, SC$\underline{H}_2$-alkyl), 2.09-1.92 (4 H, m, HC=CC$\underline{H}_2$), 1.63-1.18 (20 H, m, alkyl C$\underline{H}_2$), 0.97-0.81 (9 H, m, C$\underline{H}_3$); δ_C (75 MHz, $CDCl_3$) 143.5 (C), 120.7 (CH), 36.9 (CH_2), 32.1 (CH_2), 31.9 (2x CH_2), 31.1 (CH_2), 30.2 (CH_2), 29.6 (CH_2), 29.4 (CH_2), 29.3 (CH_2), 28.8 (CH_2), 28.3 (CH_2), 22.8 (2x CH_2), 22.3 (CH_2), 14.3 (2x CH_3), 13.9 (CH_3); Found (APCI): $[M+H]^+$ 299.2761, $C_{19}H_{39}S$ requires 299.2767.

Cyclohexyl(3-hexylnon-2-en-1-yl)sulfide (3l)

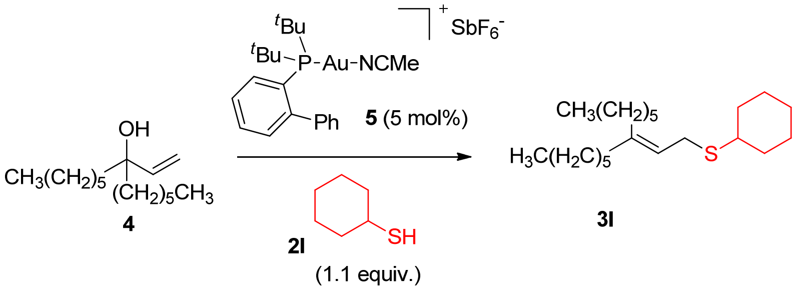

The reaction was carried out at 60 °C following general procedure B. The product was purified using flash column chromatography using neat hexane. Product **3l** was obtained as colourless liquid (12.0 mg, 36.4 µmol, 56%).

ν_{max}/cm^{-1} 2925, 2853, 1448, 1378, 1262, 1199, 998, 886, 724; δ_H (300 MHz, $CDCl_3$) 5.21 (1 H, t, J = 7.7 Hz, C=C$\underline{H}$), 3.17 (2 H, d, J = 7.7 Hz, C=CHC$\underline{H}_2$), 2.76-2.50 (1 H, m, SC$\underline{H}$), 2.12-1.86 (4 H, m, HC=CC$\underline{H}_2$), 1.85-1.10 (26 H, m, alkyl C$\underline{H}_2$), 0.75-0.95 (6 H, m, C$\underline{H}_3$); δ_C (75 MHz, $CDCl_3$) 143.1 (C), 121.0 (CH), 43.0 (CH), 36.9 (CH_2), 33.9 (CH_2), 31.9 (2x CH_2), 31.8 (CH_2), 30.1 (CH_2), 29.7 (CH_2), 29.2 (CH_2), 28.7 (CH_2), 28.2 (CH_2), 27.9 (CH_2), 26.4 (CH_2), 26.1 (CH_2), 22.8 (2x CH_2), 14.3 (2x CH_3); Found (APCI): $[M+H]^+$ 325.2919, $C_{21}H_{41}S$ requires 325.2923.

2-((3-Hexylnon-2-en-1-yl)thio)ethanol (3n)

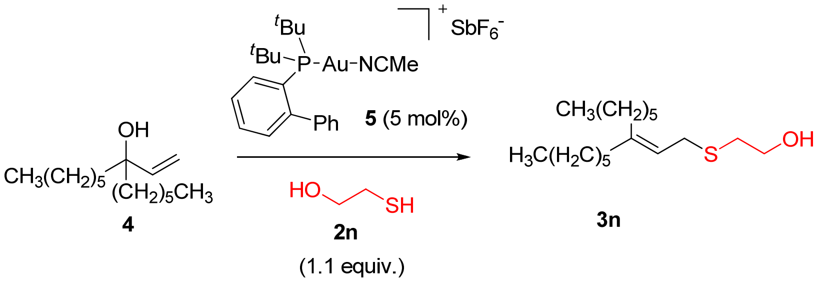

The reaction was carried out at 50 °C following general procedure B. The product was purified using flash column chromatography using a gradient eluent system of neat hexane to 5:1 hexane:diethyl ether. Product **3n** was obtained as yellow liquid (8.50 mg, 29.7 µmol, 45%).

ν_{max}/cm^{-1} 3366, 2955, 2924, 2856, 1655, 1465, 1378, 1221, 1045, 1013, 724; δ_H (300 MHz, $CDCl_3$) 5.20 (1 H, t, J = 7.7 Hz, C=C$\underline{H}$), 3.70 (2 H, t, J = 5.8 Hz, C$\underline{H}_2$OH), 3.16 (2 H, d, J = 7.7 Hz, C=CHC$\underline{H}_2$), 2.69 (2 H, t, J = 5.8 Hz, SC$\underline{H}_2$$CH_2$), 2.13 (1 H, s, O$\underline{H}$), 2.06-1.92 (4 H, m, HC=CC$\underline{H}_2$), 1.46-1.13 (16 H, m, alkyl C$\underline{H}_2$), 0.98-0.75 (6 H, m, C$\underline{H}_3$); δ_C (75 MHz, $CDCl_3$) 144.4 (C), 120.0 (CH), 60.4 (CH_2), 36.9 (CH_2), 34.6 (CH_2), 31.9 (2x CH_2), 30.3 (CH_2), 29.6 (CH_2), 29.3 (CH_2), 28.8 (CH_2), 28.7 (CH_2), 28.2 (CH_2), 22.8 (2x CH_2), 14.3 (2x CH_3); Found (APCI): $[M+H]^+$ 287.2404, $C_{17}H_{35}OS$ requires 287.2403.

3-(3-Hexylnon-2-enylthio)propanoic acid (3o)

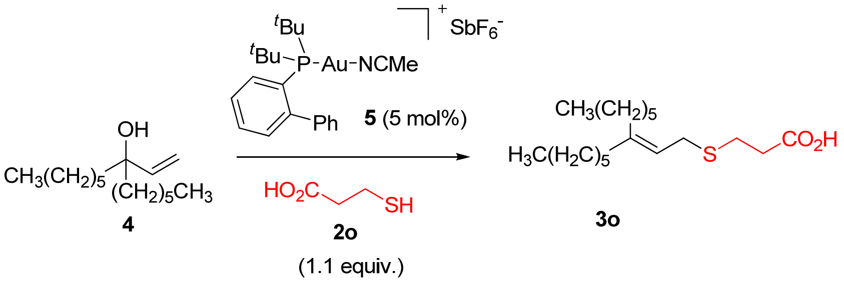

The reaction was carried out at 45 °C following general procedure B. The product was purified using flash column chromatography using a gradient eluent system of neat hexane to 1:1 hexane:diethyl ether. Product **3o** was obtained as colourless liquid (18.5 mg, 58.8 µmol, 89%).

ν_{max}/cm^{-1} 3035, 2955, 2925, 2856, 1709, 1430, 1264, 1197, 927, 724; δ_H (300 MHz, $CDCl_3$) 5.20 (1 H, t, J = 7.7 Hz, C=C$\underline{H}$), 3.19 (2 H, d, J = 7.7 Hz, C=CHC$\underline{H}_2$), 2.80-2.59 (4 H, m, SC$\underline{H}_2$C$\underline{H}_2$), 2.10-1.90 (4 H, m, HC=CC$\underline{H}_2$), 1.48-1.14 (16 H, m, alkyl C$\underline{H}_2$), 0.95-0.76 (6 H, m, C$\underline{H}_3$); δ_C (75 MHz, $CDCl_3$) 177.7 (C), 144.4 (C), 120.0 (CH), 36.9 (CH_2), 34.8 (CH_2), 31.9 (2x CH_2), 30.2 (CH_2), 29.6 (CH_2), 29.5 (CH_2), 29.3 (CH_2), 28.7 (CH_2), 28.2 (CH_2), 25.9 (CH_2), 22.8 (2x CH_2), 14.3 (2x CH_3); Found (APCI): $[M+H]^+$ 315.2356, $C_{18}H_{35}O_2S$ requires 315.2352.

General procedure C for 3p-3w:

4-Nitrothiophenol **2g** (1.1 equiv.) and **5** (5 mol%) were added to a solution of allylic alcohol substrate (1 equiv.) in chloroform (0.386 M) in a 1 dram vial. The vial was capped and the solution allowed to stir at 35 °C for 24 h. The mixture was then filtered through a plug of silica with diethyl ether. The filtrate was concentrated under reduced pressure. Purification of the crude material was carried out by flash column chromatography.

(3,3-Dicyclohexylallyl)(4-nitrophenyl)sulfane (3p)

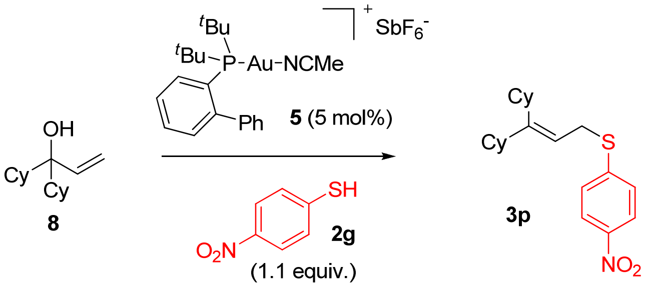

General procedure C. Purified using flash column chromatography using a gradient eluent system of neat hexane to 50:1 hexane:diethyl ether. Product **3p** was obtained as yellow solid (20.8 mg, 57.9 μmol, 86%).

M.p. 102-104 °C; ν_{max}/cm^{-1} 2926, 2851, 1659, 1573, 1503, 1447, 1334, 1092, 906, 851, 740; δ_H (300 MHz, $CDCl_3$) 8.11 (2 H, d, *J* = 9.0 Hz, Ar-H), 7.33 (2 H, d, *J* = 9.0 Hz, Ar-H), 5.24 (1 H, t, *J* = 7.6 Hz, C=CH), 3.72 (2H, d, *J* = 7.6 Hz, C=CHCH_2), 2.43 (1 H, m, cyclohexyl CH), 1.92 (1 H, m, cyclohexyl CH'), 1.83-0.93 (20 H, m, cyclohexyl CH_2); δ_C (75 MHz, $CDCl_3$) 156.8 (C), 148.5 (C), 145.1 (C), 126.8 (CH), 123.9 (CH), 115.4 (CH), 41.2 (CH), 40.5 (CH), 35.0 (CH_2), 31.0 (CH_2), 30.3 (CH_2), 27.2 (CH_2), 26.6 (CH_2), 26.3 (CH_2), 26.2 (CH_2); Found (APCI): $[M+H]^+$ 360.1984, $C_{21}H_{30}NO_2S$ requires 360.1992.

(E)-(4-Nitrophenyl)(3,4,4-trimethylpent-2-enyl)sulfane (3q)

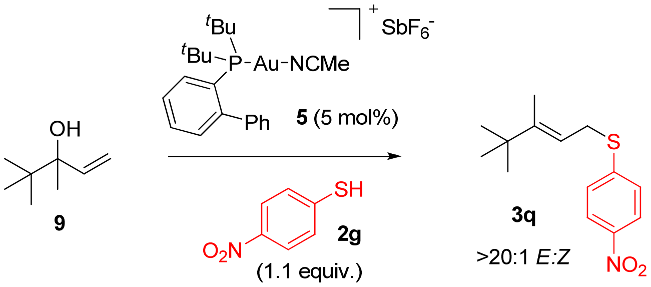

General procedure C. Purified using flash column chromatography; using a gradient eluent system of neat hexane to 20:1 hexane:diethyl ether. Product obtained as yellow liquid (18.3 mg, 69.0 µmol, 59%). ν_{max}/cm^{-1} 2963, 2870, 1646, 1594, 1577, 1509, 1478, 1333, 1090, 852, 836, 741; δ_H (300 MHz, $CDCl_3$) 8.11 (2 H, d, *J* = 9.0 Hz, Ar-H), 7.31 (2 H, d, *J* = 9.0 Hz, Ar-H), 5.36 (1 H, t, *J* = 7.4 Hz, C=CH), 3.67 (2 H, d, *J* = 7.4 Hz, C=CHCH_2), 1.73 (3 H, s, C=CCH_3), 1.04 (9 H, s, C=CC(CH_3)$_3$); δ_C (75 MHz, $CDCl_3$) 150.0 (C), 148.4 (C), 145.1 (C), 126.7 (CH), 123.9 (CH), 114.6 (CH), 36.7 (C), 31.0 (CH_2), 29.0 (CH_3), 13.2 (CH_3); Found (APCI): [M+H]$^+$ 266.1207, $C_{14}H_{20}NO_2S$ requires 266.1209.

(E)-(4-Nitrophenyl)(3-phenylbut-2-enyl)sulfane (3r)

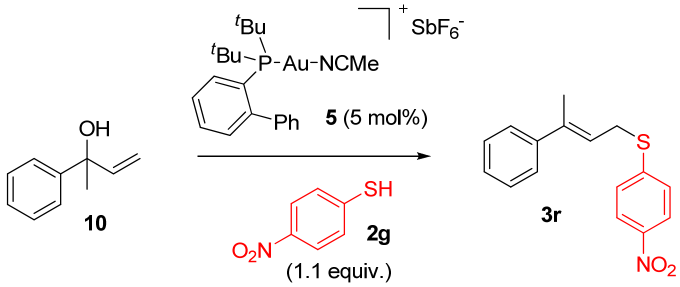

General procedure C. ^{1}H NMR analysis of crude reaction mixture showed that *E*:*Z* ratio was 9:1. Purified using flash column chromatography; using a gradient eluent system of neat petroleum ether to 50:1 petroleum ether:diethyl ether. Product **3r** (*E* isomer only) was obtained as a yellow oil (17.0 mg, 59.6 µmol, 59%).

ν_{max}/cm^{-1} 2919, 1682, 1594, 1577, 1509, 1478, 1334, 1089, 852, 837, 740; δ_H (300 MHz, $CDCl_3$) 8.13 (2 H, d, *J* = 9.0 Hz, SAr-H), 7.37 (2 H, d, *J* = 9.0 Hz, SAr-H), 7.42-7.23 (5 H, m, Ar-H), 5.89 (1 H, t, *J* = 7.6 Hz, C=CH), 3.86 (2 H, d, *J* = 7.6 Hz, C=CHCH_2), 2.14 (3 H, s, CCH_3); δ_C (75 MHz, $CDCl_3$) 147.7 (C), 145.3 (C), 142.6 (C), 140.4 (C), 128.5 (CH), 127.7 (CH), 126.9 (CH), 125.9 (CH), 124.1 (CH), 120.9 (CH), 31.2 (CH_2), 16.3 (CH_3); Found (APCI): [M+H]$^+$ 286.0891, $C_{16}H_{16}NO_2S$ requires 286.0896.

(E)-(3-Cyclohexylbut-2-enyl)(4-nitrophenyl)sulfane (3s)

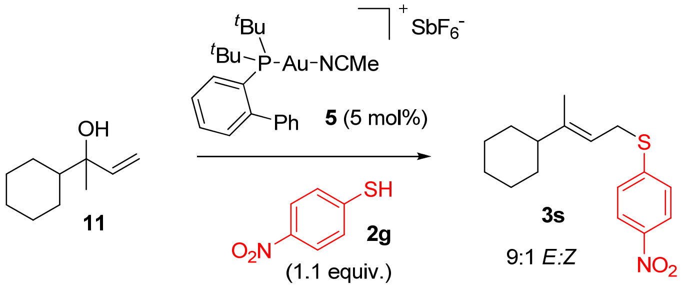

General procedure C. ^{1}H NMR analysis of crude reaction mixture showed that *E*:*Z* ratio was approximately 6:1. Purified using flash column chromatography; using a gradient eluent system of neat hexane to 50:1 hexane:diethyl ether. Product **3s** was obtained as yellow liquid (20.7 mg, 71.0 µmol, 73%) as a 6:1 mixture of *E* and *Z* isomers. Characterisation of the major *E* isomer is given below.

ν_{max}/cm^{-1} 2924, 2851, 1655, 1593, 1577, 1508, 1332, 1090, 852, 836, 741; δ_H (300 MHz, $CDCl_3$) 8.10 (2 H, d, *J* = 9.0 Hz, Ar-H), 7.31 (2 H, d, *J* = 9.0 Hz, Ar-H), 5.33-5.19 (1 H, m, *J* = 7.4 Hz, C=CH), 3.72-3.62 (2 H, m, *J* = 7.4 Hz, C=CHCH_2), 1.95-1.02 (11 H, cyclohexyl CH, cyclohexyl CH_2), 1.69 (3 H, s, CH_3); δ_C (75 MHz, $CDCl_3$) 148.4 (C), 147.2 (C), 145.1 (C), 126.6 (CH), 124.0 (CH), 115.8 (CH), 47.4 (CH), 31.8 (CH_2), 31.2 (CH_2), 30.6 (CH_2), 29.8 (CH_2), 26.7 (CH_2), 26.4 (CH_2), 14.9 (CH_3); Found (APCI): $[M+H]^+$ 292.1364, $C_{16}H_{22}NO_2S$ requires 292.1366.

(2-Cyclohexylideneethyl)(4-nitrophenyl)sulfane (3t)

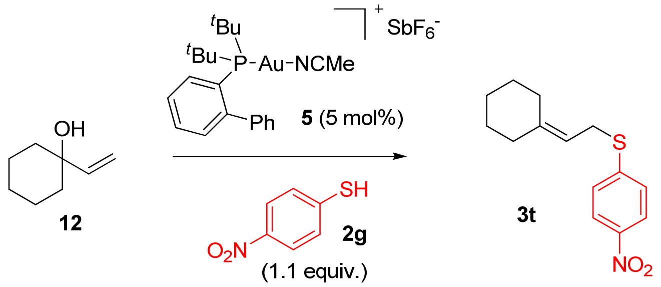

General procedure C. Purified using flash column chromatography; using a gradient eluent system of neat hexane to 50:1 hexane:diethyl ether. Product **3t** was obtained as a yellow oil (17.3 mg, 65.7 µmol, 55%). ν_{max}/cm^{-1} 2927, 2853, 1661, 1593, 1577, 1508, 1332, 1090, 852, 835, 740; δ_H (300 MHz, $CDCl_3$) 8.10 (2 H, d, *J* = 9.0 Hz, Ar-H), 7.32 (2 H, d, *J* = 9.0 Hz, Ar-H), 5.24 (1 H, t, *J* = 7.6 Hz, C=CH), 3.67 (2 H, d, *J* = 7.6 Hz, C=CHCH_2S), 2.26-2.02 (4 H, m, C=CCH_2CH_2), 1.61-1.47 (6 H, m, CH_2); δ_C (75 MHz, $CDCl_3$) 148.3 (C), 146.2 (C), 145.1 (C), 126.6 (CH), 123.9 (CH), 114.3 (CH), 37.1 (CH_2), 29.9 (CH_2), 29.1 (CH_2), 28.5 (CH_2), 27.9 (CH_2), 26.7 (CH_2); Found (APCI): $[M+H]^+$ 264.1051, $C_{14}H_{18}NO_2S$ requires 264.1053.

(4-Butyloct-3-en-2-yl)(4-nitrophenyl)sulfane (3u)

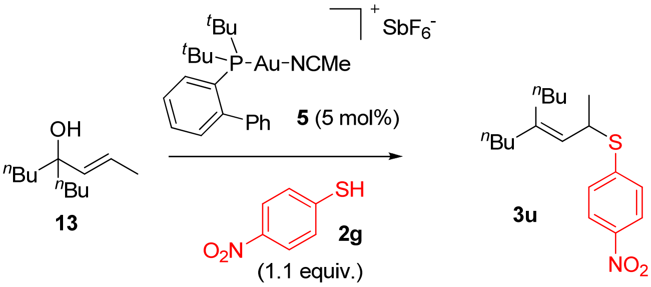

General procedure C. Purified using flash column chromatography; using a gradient eluent system of neat hexane to 50:1 hexane:diethyl ether. Product **3u** was obtained as yellow liquid (13.0 mg, 40.4 μmol, 50%).

ν_{max}/cm^{-1} 2956, 2929, 2860, 1653, 1577, 1512, 1335, 1091, 852, 742; δ_H (300 MHz, $CDCl_3$) 8.09 (2 H, d, J = 9.0 Hz, Ar-H), 7.38 (2 H, d, J = 9.0 Hz, Ar-H), 5.09 (1 H, d, J = 9.8 Hz, C=CH), 4.26 (1 H, dq, J = 9.8, 6.7 Hz, C=CHCH), 2.21-1.86 (4 H, m, HC=CH_2), 1.40 (3 H, d, J = 6.7 Hz, $SCHCH_3$), 1.36-1.08 (8 H, m, alkyl CH_2), 0.95-0.78 (6 H, m, alkyl CH_3); δ_C (75 MHz, $CDCl_3$) 147.0 (C), 145.6 (C), 143.3 (C), 129.4 (CH), 126.0 (CH), 123.8 (CH), 40.6 (CH), 36.3 (CH_2), 30.8 (CH_2), 30.4 (CH_2), 30.3 (CH_2), 23.1 (CH_2), 22.5 (CH_2), 21.9 (CH_3), 14.2 (CH_3), 14.1 (CH_3); Found (APCI): $[M+H]^+$ 322.1831, $C_{18}H_{28}NO_2S$ requires 322.1835.

(E)-(4-Nitrophenyl)(4,5,5-trimethylhex-3-en-2-yl)sulfane (3v)

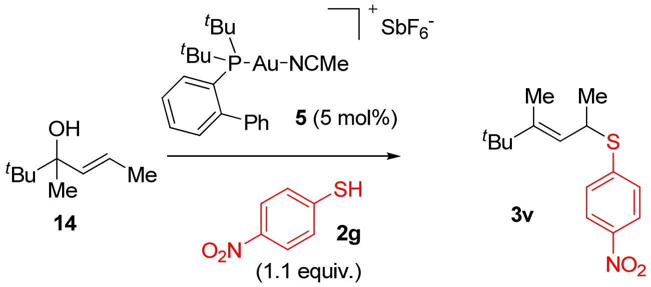

General procedure C. Purified using flash column chromatography using eluent system 19:1 hexane-Et_2O. Product **3v** was obtained a colourless oil (21 mg, 75 μmol, 72%).

ν_{max}/cm^{-1} 2961, 2924, 2868, 1594, 1577, 1513, 1477, 1361, 1260, 1110, 1084, 1010, 852 cm^{-1}; ^{1}H NMR (300 MHz, $CDCl_3$) δ_H 8.09 (2H, d, J = 9.0 Hz, ArH), 7.36 (2H, d, J = 9.0 Hz, ArH), 5.16 (1H, dq, J = 9.4, 1.3 Hz, C=CH), 4.23 (1H, dq, J = 9.4, 6.7 Hz, SCH), 1.66 (3H, d, J = 1.3 Hz, =CCH_3), 1.41 (3H, d, J = 6.7 Hz, $SCHCH_3$), 0.99 (9H, s, CMe_3); ^{13}C NMR (75 MHz, $CDCl_3$) δ_C 147.1 (C), 146.7 (C), 145.6 (C), 129.4 (CH), 123.7 (CH), 123.3 (CH), 41.1 (CH), 36.4 (C), 29.0 (CH_3), 21.4 (CH_3), 13.3 (CH_3); Found (APCI): $[M+H]^+$ 280.1369, $C_{15}H_{22}NO_2S$ requires 280.1366.

(3-Butyl-2-methylhept-2-enyl)(4-nitrophenyl)sulfane (3w)

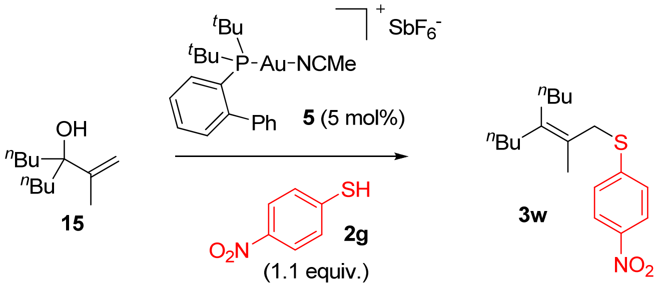

General procedure C. Purified using flash column chromatography; using a gradient eluent system of neat hexane to 50:1 hexane:diethyl ether. Product **3w** was obtained as yellow liquid (16.0 mg, 49.8 μmol, 61%).

ν_{max}/cm^{-1} 2956, 2929, 2860, 1578, 1511, 1334, 1091, 852, 837, 742; δ_H (300 MHz, $CDCl_3$) 8.11 (2 H, d, J = 9.0 Hz, Ar-H), 7.33 (2 H, d, J = 9.0 Hz, Ar-H), 3.69 (2 H, s, SCH_2), 2.09-1.97 (4 H, m, $C{=}CCH_2$ alkyl), 1.79 (3 H, s, $C{=}CCH_3$), 1.42-1.21 (8 H, m, alkyl CH_2), 0.96-0.84 (6 H, m, alkyl CH_3); δ_C (75 MHz, $CDCl_3$) 149.2 (C), 145.1 (C), 141.1 (C), 126.8 (CH), 123.9 (CH), 121.5 (C), 37.4 (CH_2), 32.5 (CH_2), 32.4 (CH_2), 31.5 (CH_2), 30.8 (CH_2), 23.2 (CH_2), 23.0 (CH_2), 18.2 (CH_3), 14.2 (2x CH_3); Found (APCI): $[M+H]^+$ 322.1831, $C_{18}H_{28}NO_2S$ requires 322.1835.

General procedure D for 3x-3aa, 24 and 25 (secondary or primary allylic alcohol substrates):
4-Nitrothiophenol **2g** (1.1 equiv.) and **5** (5 mol%) were added to a solution of allylic alcohol (1 equiv.) in chloroform (0.386 M) in a 1 dram vial. The vial was capped and solution allowed to stir at 45 °C for 72 h. The mixture was then filtered through a plug of silica with diethyl ether. The filtrate was concentrated under reduced pressure. Purification of the crude material was done by flash column chromatography.

Cyclohex-2-enyl(4-nitrophenyl)sulfane (3x)

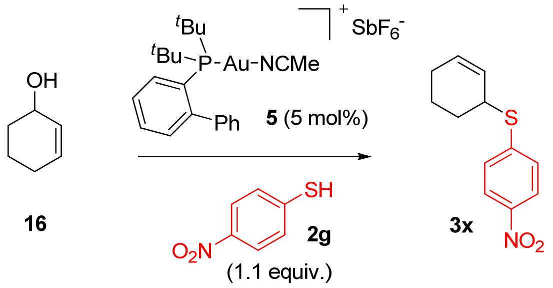

General procedure D. Purified using flash column chromatography using a gradient eluent system of neat hexane to 100:1 hexane:diethyl ether. Product **3x** was obtained as yellow liquid (32.2 mg, 137 μmol, 89%).

ν_{max}/cm^{-1} 2927, 2834, 1645, 1593, 1575, 1505, 1478, 1331, 1084, 852, 836, 741; δ_H (300 MHz, $CDCl_3$) 8.12 (2 H, d, J = 9.0 Hz, Ar-H), 7.38 (2 H, d, J = 9.0 Hz, Ar-H), 5.93 (1 H, m, C=CH), 5.75 (1 H, m, C=CH), 4.07 (1 H, m, SCH), 2.17-1.59 (6 H, m, CH_2); δ_C (75 MHz, $CDCl_3$) 147.4 (C), 145.3 (C), 132.1 (CH), 127.4 (CH), 125.3 (CH), 124.1 (CH), 42.2 (CH), 28.6 (CH_2), 24.9 (CH_2), 19.5 (CH_2); Found (APCI): $[M+H]^+$ 236.0739, $C_{12}H_{14}NO_2S$ requires 236.0740.

Cinnamyl(4-nitrophenyl)sulfane (3y)

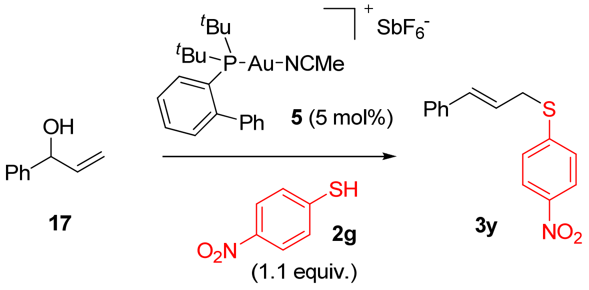

General procedure D. Purified using flash column chromatography using a gradient eluent system of neat hexane to 50:1 hexane:diethyl ether. Product **3y** was obtained as yellow solid (19.4 mg, 71.5 µmol, 64%). M.p. 90-92 °C; ν_{max}/cm^{-1} 2923, 2853, 1674, 1573, 1507, 1477, 1330, 1090, 1078, 968, 851, 835, 760, 739, 692, 681; δ_H (300 MHz, $CDCl_3$) 8.13 (2 H, d, *J* = 9.0 Hz, SAr-H), 7.38 (2 H, d, *J* = 9.0 Hz, SAr-H), 7.37-7.20 (5 H, m, Ar-H), 6.63 (1 H, d, *J* = 15.7 Hz, HC=CHPh), 6.24 (1 H, dt, *J* = 15.7, 6.9 Hz, HC=CHPh), 3.86 (2 H, d, *J* = 6.9 Hz, SCH_2); δ_C (75 MHz, $CDCl_3$) 147.0 (C), 145.4 (C), 136.3 (C), 134.2 (CH), 128.8 (CH), 128.2 (CH), 127.1 (CH), 126.6 (CH), 124.1 (CH), 123.2 (CH), 35.2 (CH_2); Found (APCI): $[M+H]^+$ 272.0740, $C_{15}H_{14}NO_2S$ requires 272.0735.

(*E*)-(4-Nitrophenyl)(4-phenylbut-3-en-2-yl)sulfane (33z)

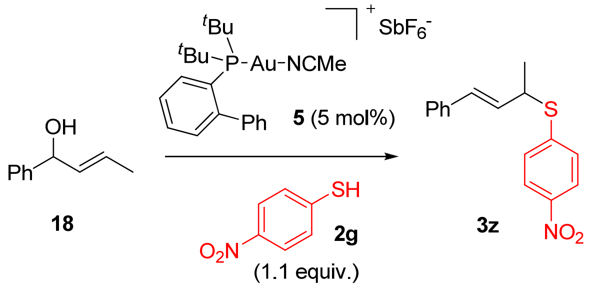

General procedure D. Purified using flash column chromatography using a gradient eluent system of neat hexane to 100:1 hexane:diethyl ether. Product **3z** was obtained as yellow oil (20.7 mg, 72.5 µmol, 72%).

ν_{max}/cm^{-1} 3026, 2968, 2924, 1576, 1507, 1333, 1082, 963, 852, 836, 741, 692; δ_H (300 MHz, $CDCl_3$) 8.10 (2 H, d, *J* = 9.0 Hz, SAr-H), 7.43 (2 H, d, *J* = 9.0 Hz, SAr-H), 7.34-7.06 (5 H, m, C=CH-Ar-H), 6.48 (1 H, d, *J* = 15.8 Hz, Ph-CH), 6.17 (1 H, dd, *J* = 15.8, 8.2 Hz, Ph-CH=CH), 4.16 (1 H, dq, *J* = 8.2, 6.9 Hz, S-CH), 1.57 (3 H, d, *J* = 6.9 Hz, CH_3); δ_C (75 MHz, $CDCl_3$) 146.1 (C), 145.8 (C), 136.3 (C), 131.3 (CH), 130.4 (CH), 129.3 (CH), 128.8 (CH), 128.1 (CH), 126.5 (CH), 123.9 (CH), 45.0 (CH), 20.8 (CH_3); Found (APCI): $[M+NH_4]^+$ 303.1158, $C_{16}H_{15}NO_2SNH_4$ requires 303.1162.

(4-Nitrophenyl)(oct-3-en-2-yl)sulfane (3aa)

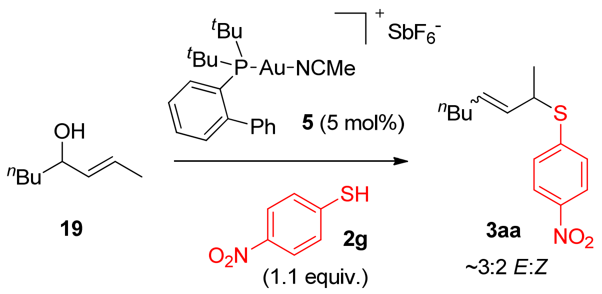

General procedure D. Purified using flash column chromatography using 99:1 hexane-Et_2O as eluent. Product **3aa** (as an inseparable mixture of ~3:2 *E*:*Z* and 8:1 S_N2':S_N2) was obtained as a colourless oil (20 mg, 75.4 µmol, 65%). The spectrum for this mixture is shown below, with the C<u>H</u>S peak of each integrated.

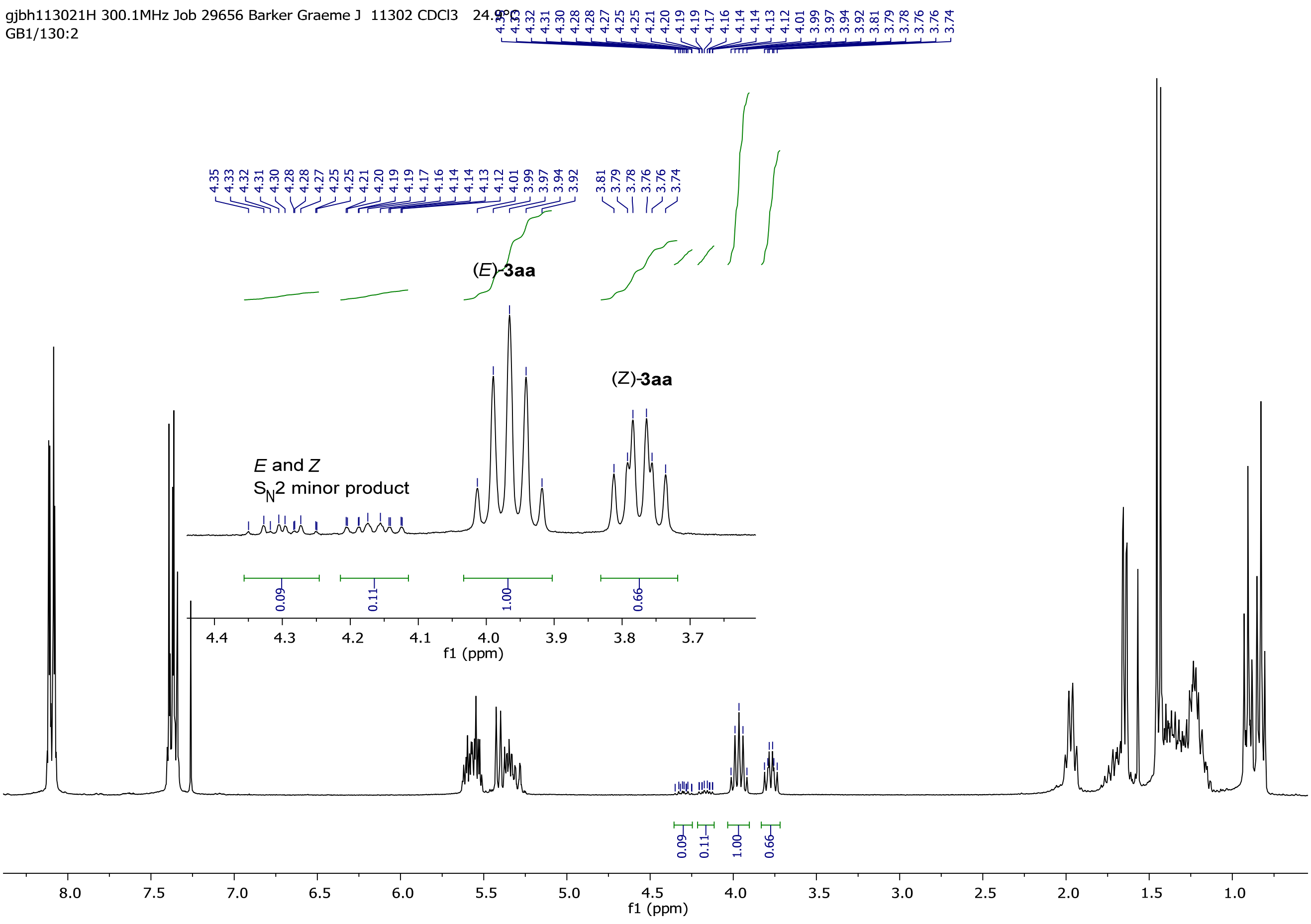

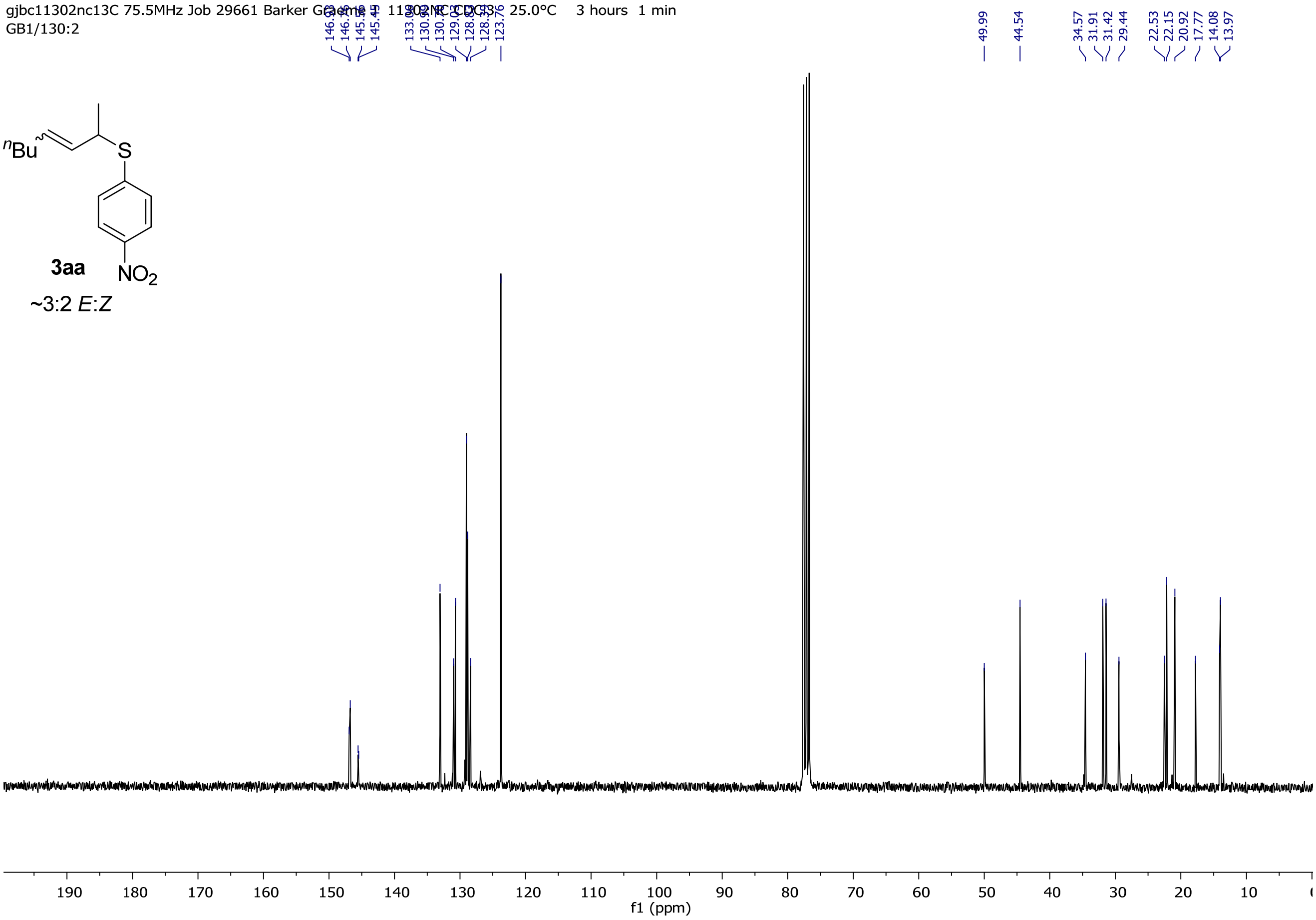

(75 MHz, $CDCl_3$: *E*/*Z* isomers of **3aa**) δ_C 146.9 (C), 146.8 (C), 145.6 (C), 145.5 (C), 133.1 (CH), 131.0 (CH), 130.7 (CH), 129.0 (CH), 128.8 (CH), 128.4 (CH), 123.8 (2 × CH), 50.0 (CH), 44.5 (CH), 34.6 (CH_2), 31.9 (CH_2), 31.4 (CH_2), 29.4 (CH_2), 22.5 (CH_2), 22.1 (CH_2), 20.9 (CH_3), 17.8 (CH_3), 14.1 (CH_3), 14.0 (CH_3).

(*E*)-(4-cyclohexylbut-3-en-2-yl)(4-nitrophenyl)sulfane (3ab)

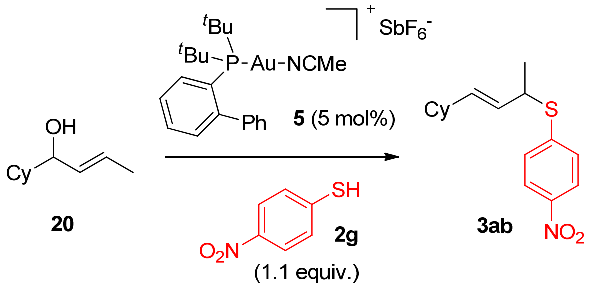

General procedure D. Purified using flash column chromatography using 19:1 hexane-Et_2O as eluent. Product **3aa** (>20:1 *E*:*Z*) was obtained as a colourless oil (24 mg, 82 μmol, 85%), as an inseparable mixture of 2:1 S_N2':S_N2 products. The spectrum for this mixture is shown below, with the CHS peak of each integrated.

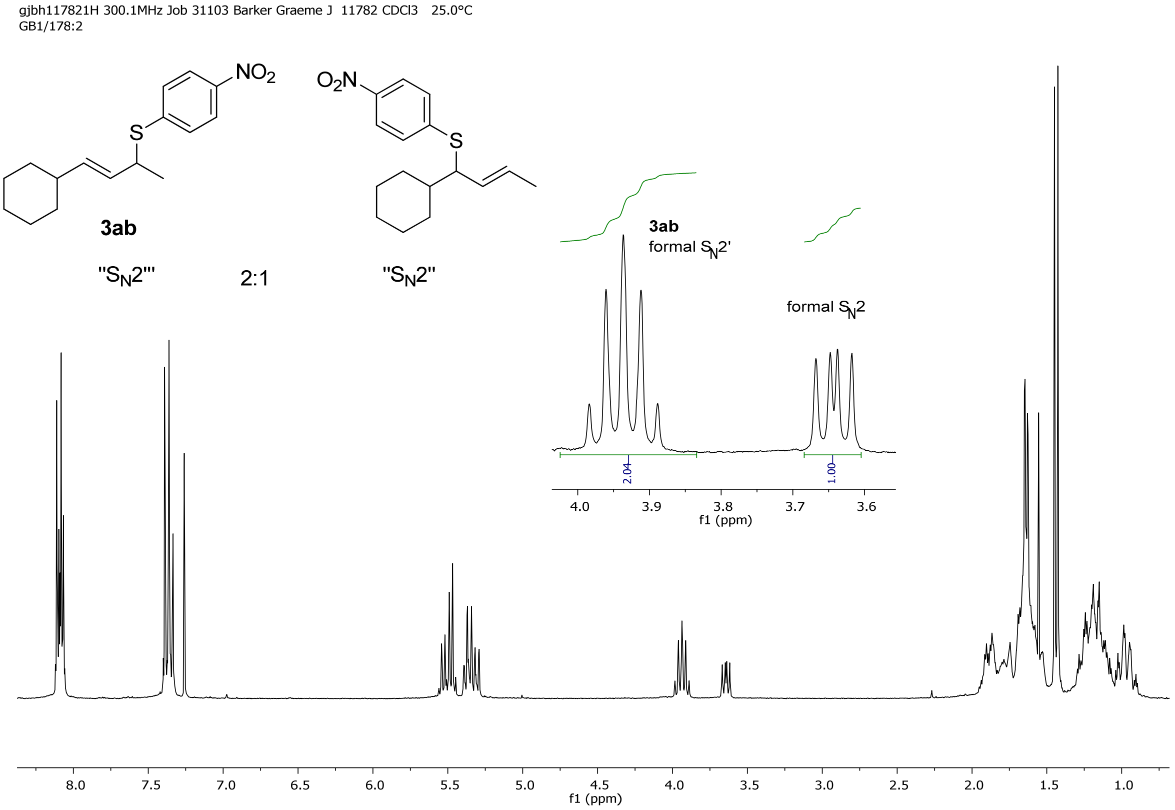

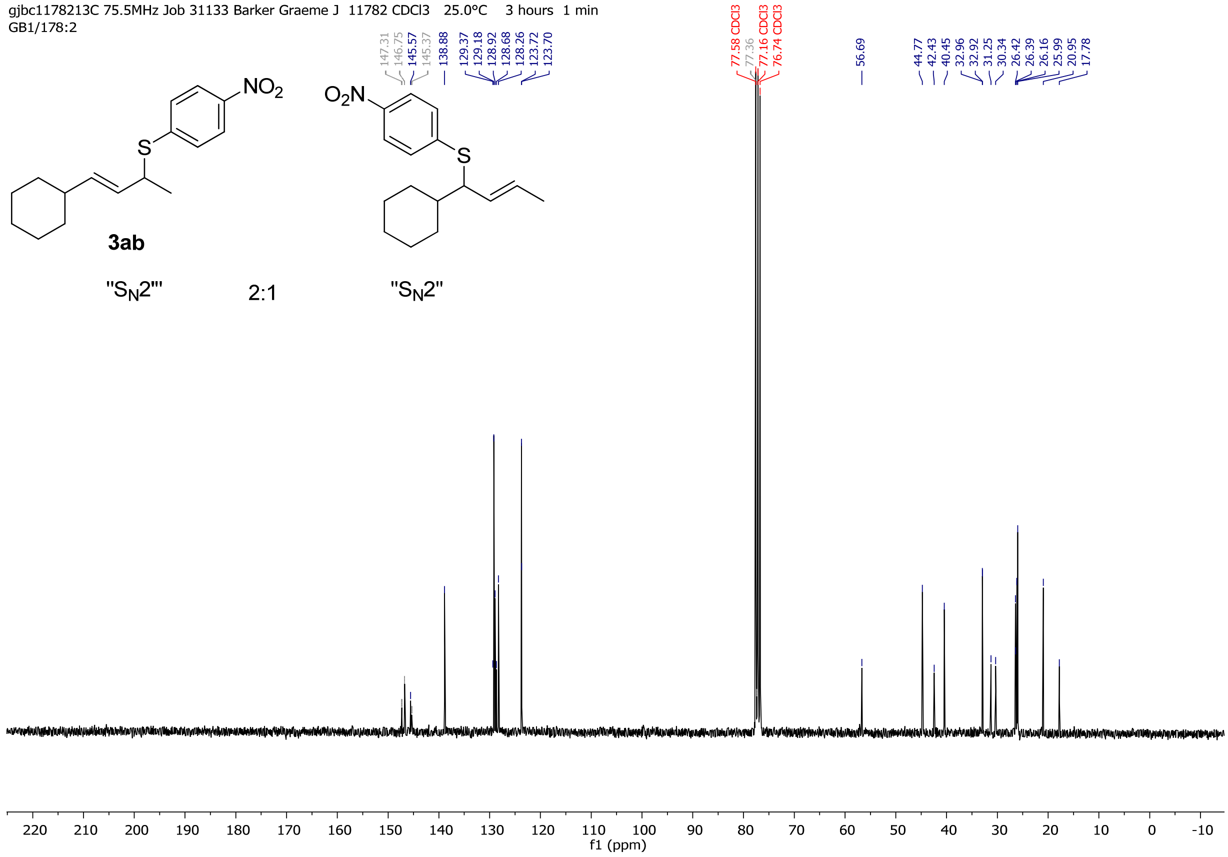

Found (APCI): $[M+H]^+$ 292.1362, $C_{16}H_{22}NO_2S$ requires 292.1366.

(*E*)-(5,5-Dimethylhex-3-en-2-yl)(4-nitrophenyl)sulfane (3ac)

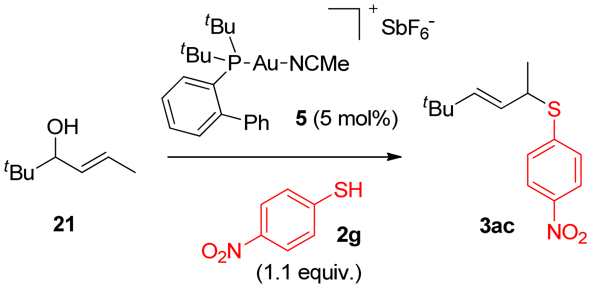

General procedure D. Purified using flash column chromatography using 19:1 hexane-Et_2O as eluent. Product **3ac** (4:1 *E*:*Z* ratio) was obtained as a colourless oil (22 mg, 83 μmol, 71%).

ν_{max}/cm^{-1} 2960, 2866, 1577, 1511, 1476, 1336, 1180, 1088, 971, 852, 838, 742 cm^{-1}; ^1H NMR (300 MHz, $CDCl_3$) (4:1 mixture of *E*:*Z* alkene isomers) δ_H 8.12-8.05 (2.5 H, m, ArH), 7.41-7.34 (2.5 H, m, ArH), 5.56 (1 H, dd, *J* = 15.6, 0.8 Hz, *E* ^{t}BuC<u>H</u>=), 5.46-5.41 (0.5 H, m, *Z* ^{t}BuC<u>H</u>= and ^{t}BuCH=C<u>H</u>), 5.27 (1 H, dd, *J* = 15.5, 8.4 Hz, *E* ^{t}BuCH=C<u>H</u>), 3.99-3.89 (1 H, m, *E* SCH), 3.54-3.50 (0.25 H, m, *Z* SCH), 1.63-1.61 (0.75 H, m, *E* SCH<u>Me</u>), 1.44 (1 H, d, *J* = 8.4 Hz, *E* SCH<u>Me</u>), 1.07 (2.25 H, s, *Z* CMe_3), 0.92 (9 H, s, *E* CMe_3); ^{13}C NMR (75 MHz, $CDCl_3$) (mixture of alkene isomers) δ_C 146.6 (C-*E*), 145.8 (C-*Z*), 144.0 (CH × 2, *E*+*Z*), 129.7 (CH × 2, *E*+*Z*), 129.0 (C-*E*), 128.6 (C-*Z*), 125.8 (CH × 2, *E*+*Z*), 123.7 (CH-*E*), 123.6 (CH-*Z*), 45.2 (CH × 2, *E*+*Z*), 35.2 (C-*Z*), 33.1 (C-*E*), 29.6 (CH_3 × 2, *E*+*Z*), 28.1 (CH_3-*Z*), 21.1 (CH_3-*E*); Found (APCI): $[M+H]^+$ 266.1208, $C_{14}H_{20}NO_2S$ requires 266.1209.

(3-Methylbut-2-enyl)(4-nitrophenyl)sulfane (25)

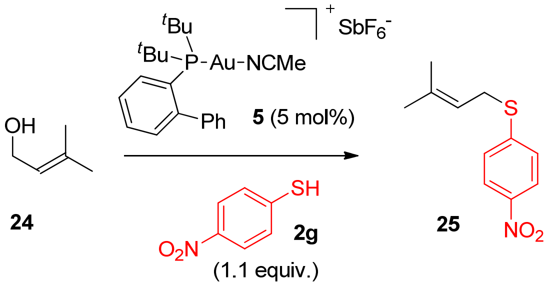

General procedure D. Purified using flash column chromatography using a gradient eluent system of neat hexane to 150:1 hexane:diethyl ether. Product **25** was obtained as colourless liquid (22.8 mg, 102 μmol, 59%).

ν_{max}/cm^{-1} 2972, 2915, 1667, 1593, 1577, 1507, 1331, 1090, 852, 835, 740; δ_H (300 MHz, $CDCl_3$) 8.11 (2 H, d, *J* = 9.0 Hz, Ar-<u>H</u>), 7.31 (2 H, d, *J* = 9.0 Hz, Ar-<u>H</u>), 5.30 (1 H, t, *J* = 7.5 Hz, C=C<u>H</u>), 3.65 (2 H, d, *J* = 7.5 Hz, C<u>H</u>$_2$), 1.75 (3 H, s, C<u>H</u>$_3$), 1.73 (3 H, s, C<u>H</u>$_3$); δ_C (75 MHz, $CDCl_3$) 148.3 (C), 145.1 (C), 138.3 (C), 126.5 (CH), 124.0 (CH), 117.7 (CH), 30.6 (CH_2), 25.8 (CH_3), 18.1 (CH_3); Found (APCI): $[M+H]^+$ 224.0740, $C_{11}H_{14}NO_2S$ requires 224.0740.

(4-Methylpent-3-en-2-yl)(4-nitrophenyl)sulfane (27)

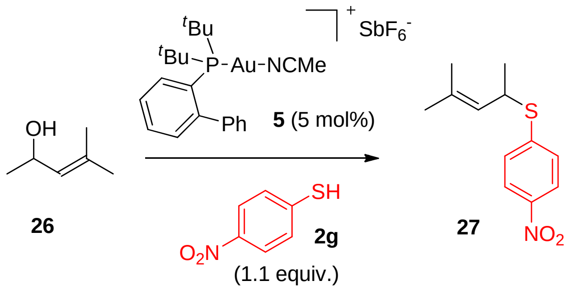

General procedure D. Purified using flash column chromatography using a gradient eluent system of neat hexane to 100:1 hexane:diethyl ether. Product **27** was obtained as yellow liquid (30.9 mg, 130 μmol, 87%).

ν_{max}/cm^{-1} 2970, 2924, 2866, 1576, 1508, 1333, 1092, 1061, 852, 835, 741; δ_H (300 MHz, $CDCl_3$) 8.09 (2 H, d, *J* = 9.0 Hz, Ar-H), 7.36 (2 H, d, *J* = 9.0 Hz, Ar-H), 5.11 (1 H, dsept, *J* = 9.6, 1.3 Hz, C=CH), 4.22 (1 H, dq, *J* = 9.6, 6.7 Hz, SCH), 1.69 (3 H, d, *J* = 1.3 Hz, HC=CCH_3), 1.66 (3 H, d, *J* = 1.3 Hz, HC=CCH_3), 1.39 (3 H, d, *J* = 6.7 Hz, SCHCH_3); δ_C (75 MHz, $CDCl_3$) 147.1 (C), 145.6 (C), 135.1 (C), 129.1 (CH), 126.3 (CH), 123.8 (CH), 40.8 (CH), 25.7 (CH_3), 21.5 (CH_3), 18.3 (CH_3); Found (APCI): $[M+H]^+$ 238.0899, $C_{12}H_{16}NO_2S$ requires 238.0896.

The following 4 procedures describe the 4 steps towards enantioenriched (*R,E*)-(4-cyclohexylbut-3-en-2-yl)(4-nitrophenyl)sulfane 3ab using a general procedure by Gais[14]:

4-Cyclohexylbut-3-yn-2-ol

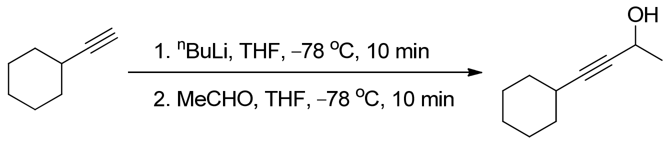

4-cyclohexylbut-3-yn-2-ol

^{n}BuLi (15.01 mL of a 1.6 M solution, 24.02 mmol) was added dropwise to a stirred solution of cyclohexylacetylene (2.0 g, 2.41 mL, 18.5 mmol) in THF (20 mL) at –78 °C under Ar. The resulting solution was stirred at –78 °C for 10 minutes, and acetaldehyde (1.06 g, 1.36 mL, 24.0 mmol) was added dropwise. The resulting solution was stirred at –78 °C for 10 minutes then allowed to warm to rt. Saturated NH_4Cl (aq) (20 mL) was added and the layers were separated, extracting the aqueous with Et_2O (3 × 20 mL). The combined organic layers were dried ($MgSO_4$) and concentrated under reduced pressure to give the crude product. Purification by flash column chromatography on silica with 9:1 pentane:Et_2O as eluent gave 4-cyclohexylbut-3-yn-2-ol (2.41 g, 86%) as a colourless oil. R_F 0.2 (9:1 pentane:Et_2O); ν_{max}/cm^{-1} 3326 (br), 2927, 2853, 1448, 1368, 1327, 1296, 1157, 1132, 1111, 1075, 1017, 976, 896, 878, 844, 634; δ_H (300 MHz, $CDCl_3$) 4.50 (1H, dqd, *J* = 6.5, 5.0, 1.6 Hz, OC<u>H</u>), 2.40-2.27 (1H, m, ≡CC<u>H</u>), 2.15 (1H, br d, *J* = 6.5 Hz, O<u>H</u>), 1.82-1.58 (4H, m, Cy-<u>H</u>), 1.50-1.23 (6H, m, Cy-<u>H</u>), 1.40 (3H, d, *J* = 5.0 Hz, OCHC<u>H</u>$_3$); δ_C (75 MHz, $CDCl_3$) 88.7 (C), 82.3 (C), 58.6 (CH), 32.7 (CH_2), 29.0 (CH), 25.9 (CH_2), 25.0 (CH_3), 24.9 (CH_2). Data consistent with that reported in the literature.[15]

(*E*)-4-Cyclohexylbut-3-en-2-ol

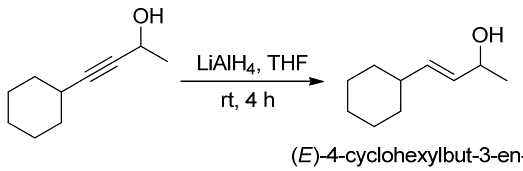

(*E*)-4-cyclohexylbut-3-en-2-ol

A solution of 4-cyclohexylbut-3-yn-2-ol (1.8 g, 11.8 mmol) in THF (10 mL) was added dropwise to a stirred suspension of $LiAlH_4$ (897 mg, 23.6 mmol) in THF (10 mL) at RT under Ar. The resulting solution was stirred at RT for 4 h. A 1 M solution of NaOH (aq.) was added dropwise until a white precipitate was observed. The solids were filtered off over Celite®, washing with Et_2O (50 mL). The filtrate was concentrated under reduced pressure to give the crude product. Purification by flash column chromatography on silica with 9:1 pentane:Et_2O as eluent gave (*E*)-4-cyclohexylbut-3-en-2-ol

(1.43 g, 78%) as a colourless oil. R_F 0.1 (9:1 pentane:Et_2O); ν_{max}/cm^{-1} 3331 (br), 2969, 2921, 2850, 1448, 1367, 1130, 1057, 966, 941, 891, 864, 842, 569; δ_H (300 MHz, $CDCl_3$) 5.54 (1H, dd, J = 15.7, 6.3 Hz, =C<u>H</u>), 5.42 (1H, ddd, J = 15.7, 6.2, 0.7 Hz, =C<u>H</u>), 4.21 (1H, dq, J = 6.3, 6.3 Hz, OC<u>H</u>), 1.97-1.81 (1H, m, =CHC<u>H</u>), 1.83 (1H, br s, O<u>H</u>), 1.75-1.58 (4H, m, Cy-<u>H</u>), 1.33-0.94 (6H, m, Cy-<u>H</u>), 1.21 (3H, d, J = 6.3 Hz, OCHC<u>H</u>$_3$); δ_C (75 MHz, $CDCl_3$) 136.8 (CH), 131.7 (CH), 69.1 (CH), 40.2 (CH), 32.9 (CH_2), 26.2 (CH_2), 26.1 (CH_2), 23.5 (CH_3). Data Consistent with that reported in the literature.[16]

(*E*)-O-4-cyclohexylbut-3-en-2-yl propylcarbamothioate

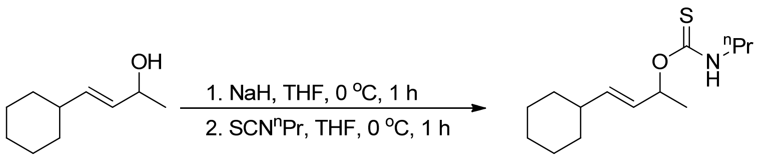

(*E*)-*O*-(4-cyclohexylbut-3-en-2-yl) propylcarbamothioate

A solution of (*E*)-4-cyclohexylbut-3-en-2-ol (500 mg, 3.24 mmol) in THF (2 mL) was added dropwise to a stirred suspension of NaH (145 mg of a 60% dispersion in mineral oil, 3.60 mmol) in THF (2 mL) at 0 °C under Ar. The resulting solution was stirred at 0 °C for 1 h. Then, a solution of ^{n}propylisothiocyanate (329 mg, 336 μL, 3.25 mmol) in THF (1 mL) was added. The resulting solution was stirred at 0 °C for 1 h. Then, a saturated solution of $NaHCO_3$ (aq.) (3 mL) was added and the layers were separated, extracting the aqueous with EtOAc (3 × 10 mL). The combined organic layers were dried ($MgSO_4$) and concentrated under reduced pressure to give the crude product. Purification by flash column chromatography on silica with 9:1 pentane:Et_2O as eluent gave (*E*)-O-4-cyclohexylbut-3-en-2-yl propylcarbamothioate (587 mg, 71%) as a colourless oil, R_F 0.2 (9:1 pentane:Et_2O); ν_{max}/cm^{-1} 3290, 2964, 2924, 2852, 1657, 1514, 1448, 1400, 1378, 1354, 1263, 1193 (C=S), 1129, 1038, 964, 928, 892, 809, 736, 703, 606; δ_H (300 MHz, $CDCl_3$) (4:1 mixture of rotamers) 6.69 (0.3H, br s, N-<u>H</u>), 6.26 (1H, br s, N-<u>H</u>), 5.97-5.84 (0.3H, m, =C<u>H</u>), 5.75-5.24 (2.3H, m, =C<u>H</u>), 3.96-3.83 (1H, m, OC<u>H</u>), 3.53-3.43 (0.3H, m, OC<u>H</u>), 3.32-3.09 (2.6H, m, NC<u>H</u>$_2$), 1.86-1.41 (13H, m, Cy-<u>H</u> + ^{n}Pr-<u>H</u>), 1.39-0.80 (10.4H, m, Cy-<u>H</u> + ^{n}Pr-<u>H</u>); δ_C (75 MHz, $CDCl_3$) (mixture of rotamers) 166.9 (2 × C), 139.2 (CH), 1302. (CH), 127.3 (CH), 126.8 (CH), 53.7 (2 × CH), 46.3 (CH_2), 44.8 (CH_2), 43.1 (2 × CH_2), 42.6 (CH), 40.3 (CH), 32.7 (CH_2), 30.9 (cH_2), 30.3 (CH_2), 26.4 (CH_2), 26.4 (CH_2), 26.2 (CH_2), 26.1 (CH_2), 23.1 (CH_2), 22.6 (CH_2), 17.9 (2 × CH_3), 11.4 (CH_3), 11.4 (CH_3); Found (NSI): $[M+H]^+$ 256.1730, $C_{14}H_{25}NOS$ requires 256.1730.

(*R*,*E*)-(4-cyclohexylbut-3-en-2-yl)(4-nitrophenyl)sulfane (3ab) [+ *rac*-29]

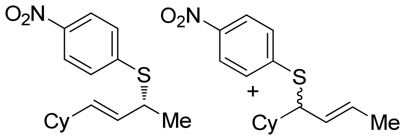

(*R*)-**3ab** 80:20 er

rac-**29** 50:50 e9

2:1 **3ab**: **29**

$Pd_2(dba)_3$ (45 mg, 0.044 mmol, 7.5 mol%) and (*R*,*R*)-DACH-phenyl Trost ligand (73 mg, 0.105 mmol) were stirred in CH_2Cl_2 (4 mL) in a Schlenk tube at rt under Ar until a colour change from red to orange was observed. Then, (*E*)-O-4-cyclohexylbut-3-en-2-yl propylcarbamothioate (300 mg, 1.17 mmol) in CH_2Cl_2 (2 mL) was added. The resulting solution was stirred at RT under Ar for 6 days. Then, a saturated solution of $NH_4Cl_{(aq)}$ (10 mL) was added. The resulting solution was stirred at RT for 30 min. The layers were separated, extracting the aqueous with CH_2Cl_2 (3 × 20 mL). The combined organic layers were dried ($MgSO_4$) and concentrated under reduced pressure. The residue was passed over a plug of silica, using 9:1 pentane:Et_2O as eluent, to give 183 mg of a yellow oil. 27 mg of this residue was taken up in dioxane (1 mL) at rt under Ar. Then, 1-iodo-4-nitrobenzene (26 mg, 0.106 mmol), $Pd(OAc)_2$ (3 mg, 0.011 mmol), triphenylphosphine (9 mg, 0.032 mmol), $^{n}Bu_4NI$ (39 mg, 0.106 mmol) and K_2CO_3 (19 mg, 0.137 mmol) were added, The resulting solution was stirred at reflux under Ar for 4 h. Then, hexane (6 mL) was added, and the solution allowed to cool to rt. The resulting suspension was filtered over Celite®, washing with Et_2O (20 mL). The filtrate was washed with H_2O (10 mL), dried ($MgSO_4$) and evaporated under reduced pressure to give the crude product. Purification by flash column chromatography on silica with 19:1 pentane:Et_2O as eluent 2:1 mixture of (*R*)-**3ab** and **29**, 80:20 er ((*R*)-**3ab**), 50:50 er (**29**) (combined yield 24 mg, 54%) as a colourless oil.

HPLC trace of 3ab (80:20 er)

Page 1 of 1

Area % Report

Data File: C:\EZChrom Elite\Enterprise\Projects\Default\Data\GB2-255-2run2 26-2-14.dat
Method: C:\EZChrom Elite\Enterprise\Projects\Default\Method\GB1-187-2.met
Acquired: 26/02/2014 16:29:59
Printed: 27/03/2014 17:02:43

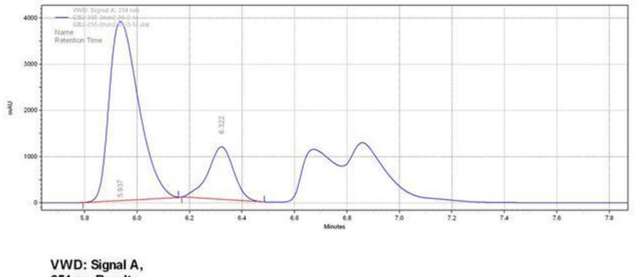

VWD: Signal A, 254 nm Results

Retention Time	Area	Area %	Height	Height %
5.937	506206485	79.68	64990110	77.35
6.322	129055871	20.32	19035174	22.65
Totals	635262356	100.00	84025284	100.00

HPLC conditions: Daicel CHIRALPAK® IA 99:1 hexane:IPA, 1 mL/min, 25 °C.

HPLC trace of 29 (racemic)

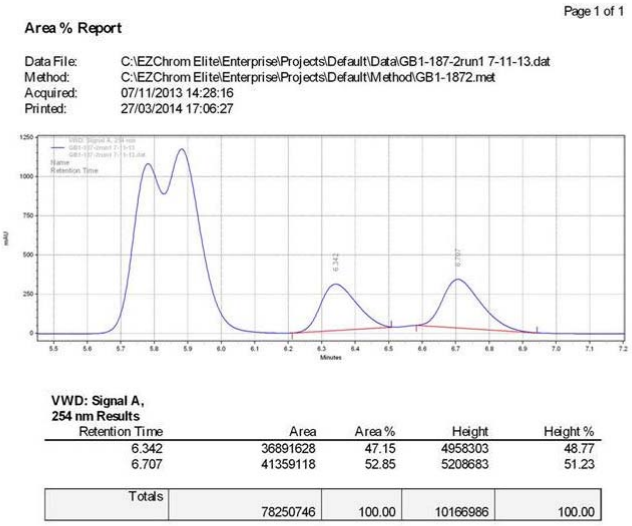
Page 1 of 1

Area % Report

Data File: C:\EZChrom Elite\Enterprise\Projects\Default\Data\GB1-187-2run1 7-11-13.dat
Method: C:\EZChrom Elite\Enterprise\Projects\Default\Method\GB1-1872.met
Acquired: 07/11/2013 14:28:16
Printed: 27/03/2014 17:06:27

VWD: Signal A, 254 nm Results

Retention Time	Area	Area %	Height	Height %
6.342	36891628	47.15	4958303	48.77
6.707	41359118	52.85	5208683	51.23
Totals	78250746	100.00	10166986	100.00

HPLC conditions: Daicel CHIRALPAK® IA, 99.4:0.6 hexane:IPA, 1mL/min, 25 °C.

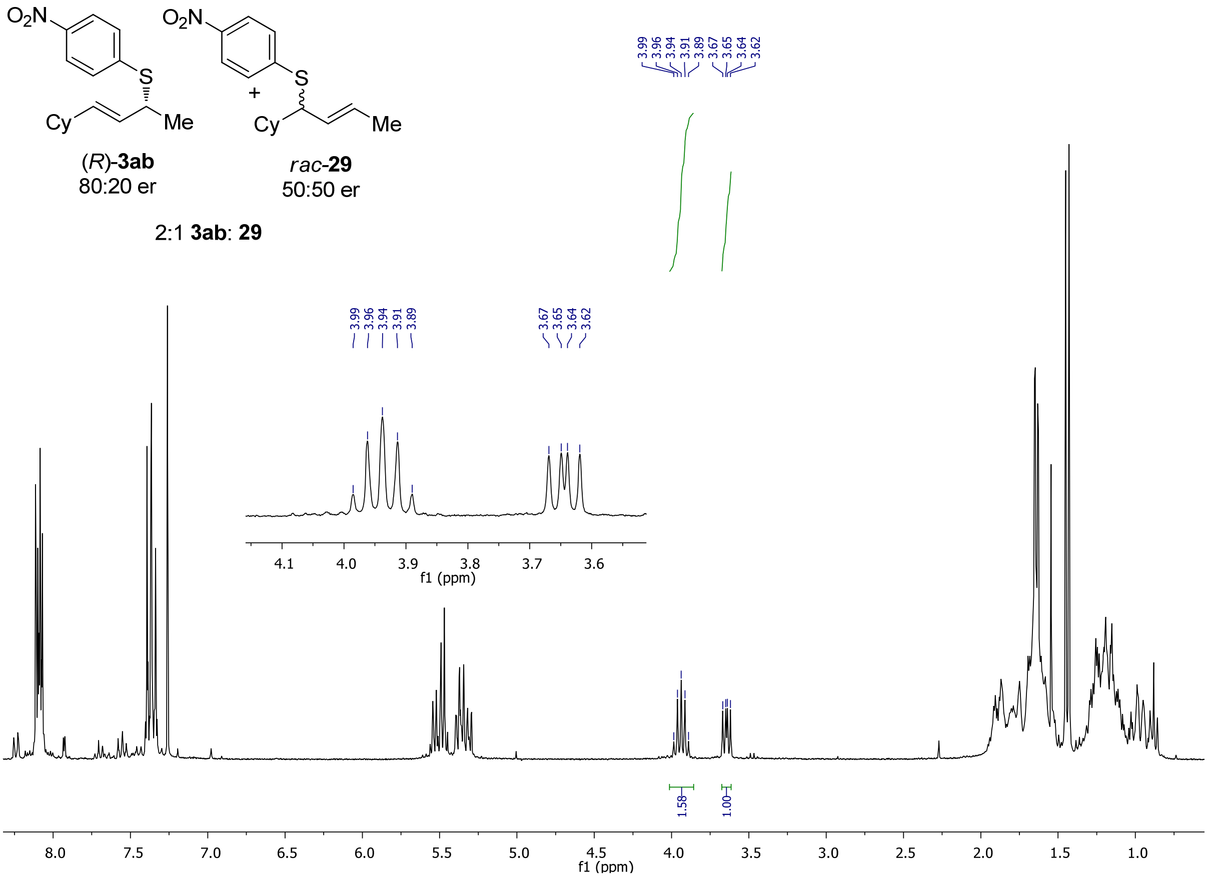

Resubjection of enantioenriched 3ab (+29)

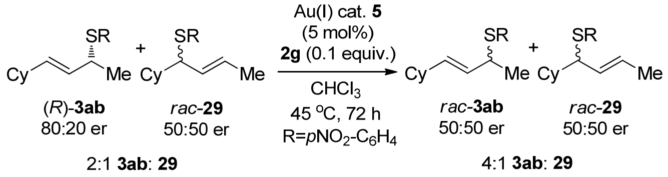

A 2:1 mixture of (*R*)-**3ab** and **29**, 80:20 er (**3ab**), 50:50 er (**29**) (12 mg, 0.041 mmol) from the previous reaction, *p*-nitrothiophenol (0.6 mg, 0.004 mmol) and catalyst **5** (2 mg, 0.002 mmol, 5 mol%) were stirred in $CHCl_3$ (107 µL) at 45 °C under air for 3 days. The resulting solution was filtered over a plug of silica, washing with 9:1 pentane:Et_2O. The filtrate was concentrated under reduced pressure to give the crude product. Purification by flash column chromatography gave a 4:1 mixture of **3ab:29** (10 mg, 83%, both 50:50 er).

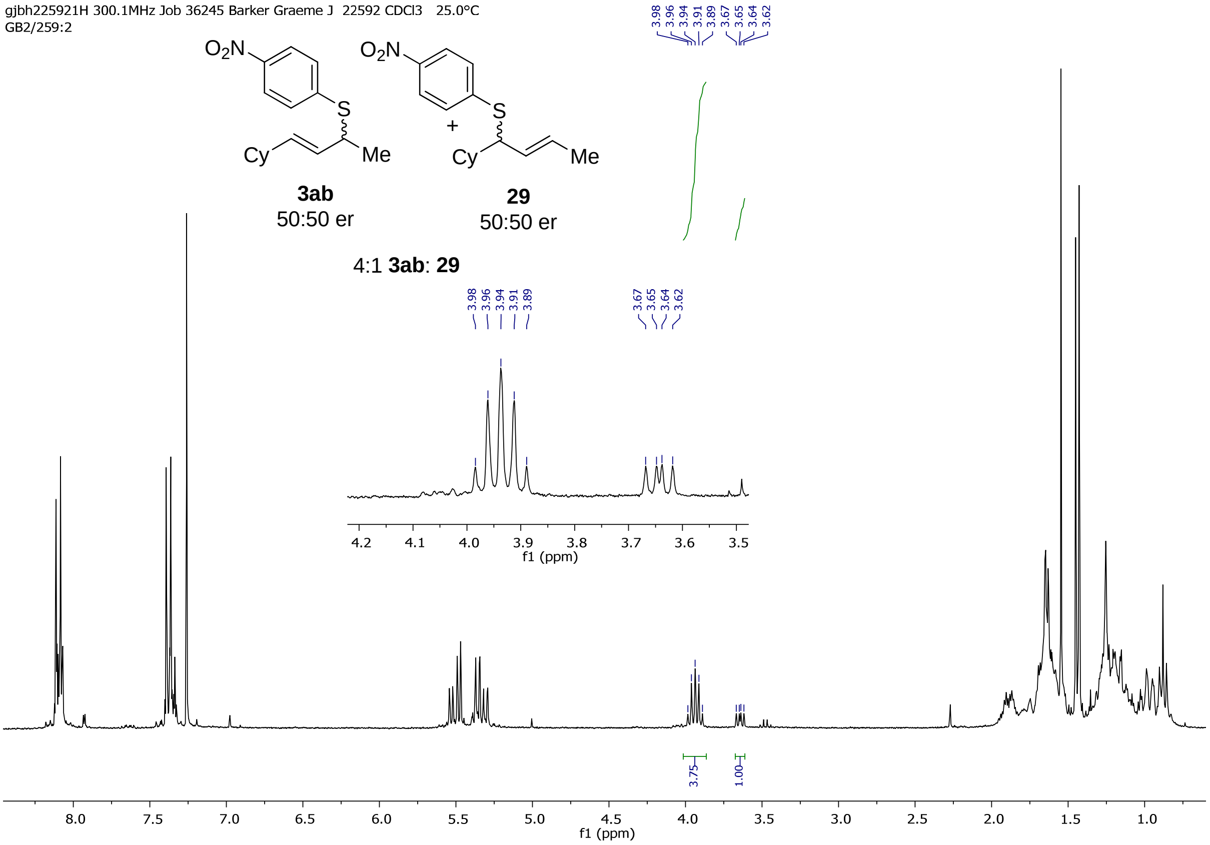

HPLC trace of product (3ab) - racemic

Page 1 of 1

Area % Report

Data File: C:\EZChrom Elite\Enterprise\Projects\Default\Data\GB2-259-2run3 13-3-14.dat
Method: C:\EZChrom Elite\Enterprise\Projects\Default\Method\GB1-187-2.met
Acquired: 13/03/2014 16:34:52
Printed: 27/03/2014 17:08:53

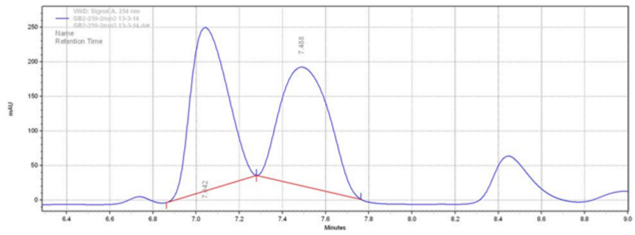

VWD: Signal A, 254 nm Results

Retention Time	Area	Area %	Height	Height %
7.042	46411448	50.44	3966051	57.96
7.488	45599245	49.56	2876903	42.04
Totals	92010693	100.00	6842954	100.00

HPLC conditions: Daicel CHIRALPAK® IA 99:1 hexane:IPA, 1 mL/min, 25 °C.

HPLC trace of product (29) - racemic

Page 1 of 1

Area % Report

Data File: C:\EZChrom Elite\Enterprise\Projects\Default\Data\GB2-259-2run1 13-3-14.dat
Method: C:\EZChrom Elite\Enterprise\Projects\Default\Method\GB1-187-2.met
Acquired: 13/03/2014 15:06:29
Printed: 27/03/2014 17:10:05

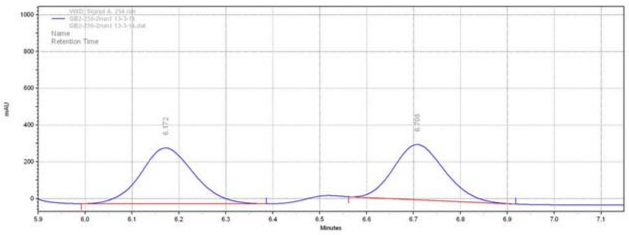

VWD: Signal A, 254 nm Results

Retention Time	Area	Area %	Height	Height %
6.172	40499072	51.28	5092604	50.19
6.708	38479994	48.72	5053779	49.81
Totals	78979066	100.00	10146383	100.00

HPLC conditions: Daicel CHIRALPAK® IA, 99.4:0.6 hexane:IPA, 1 mL/min, 25 °C.

4) ^{1}H NMR and ^{13}C NMR spectra of synthesised compounds

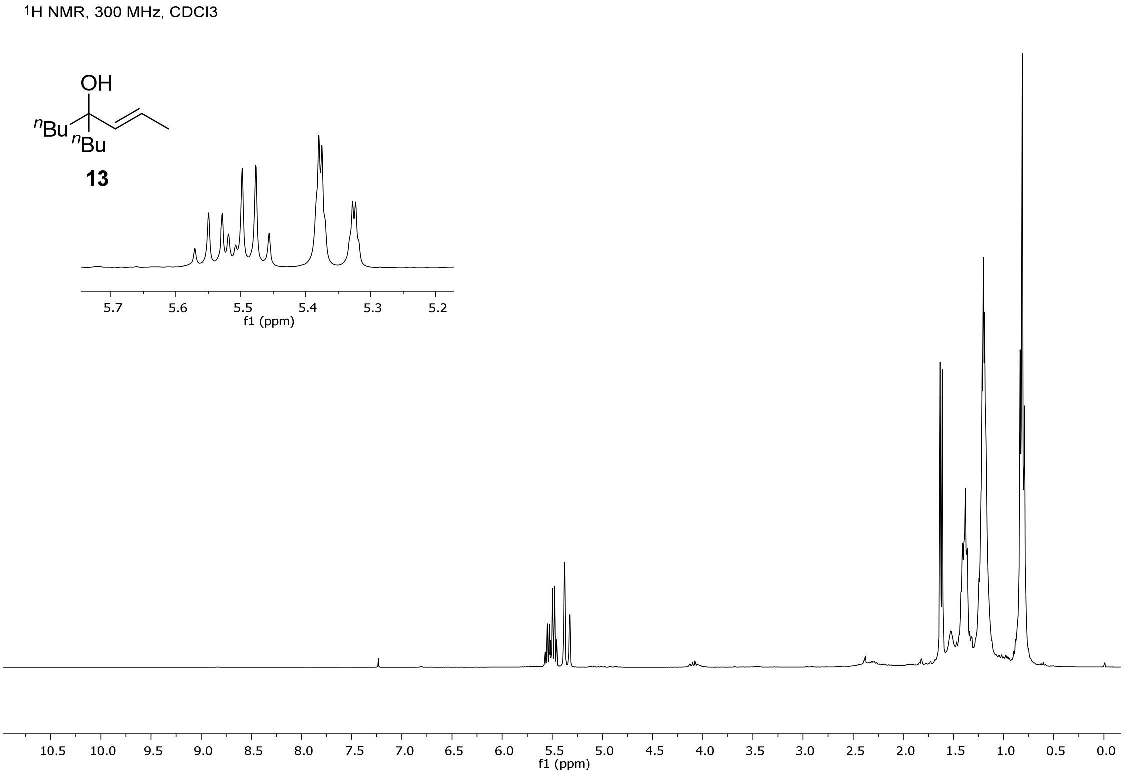

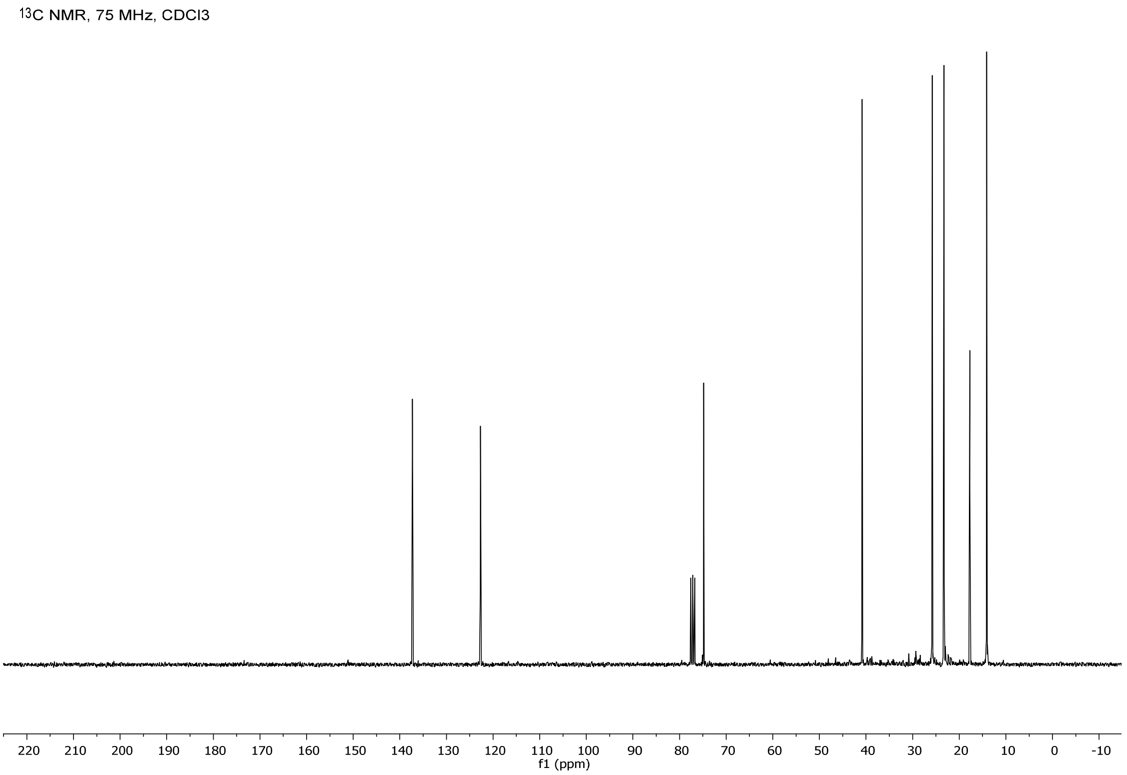

gjbh11063

¹H NMR 300 MHz CDCl3

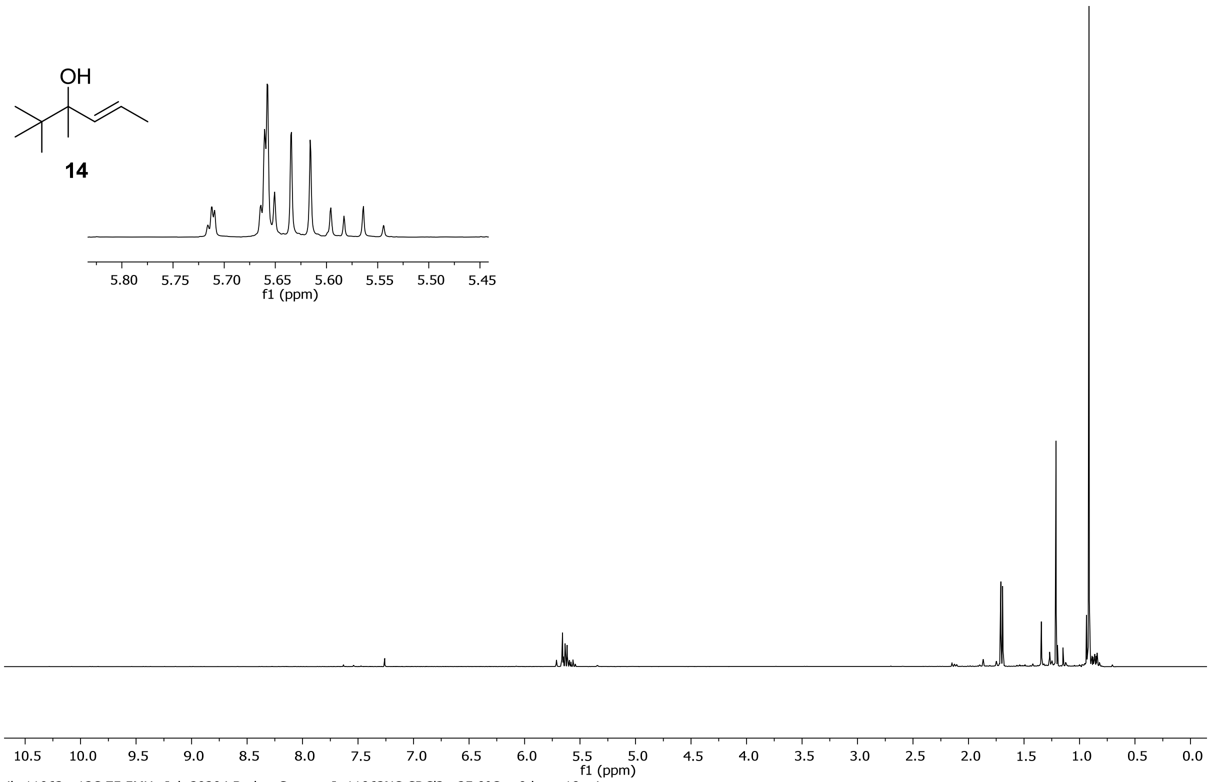

14

gjbc11063nc13C 75.5MHz Job 29204 Barker Graeme J 11063NC CDCl3 25.0°C 0 hour 18 min
GB1/106:3

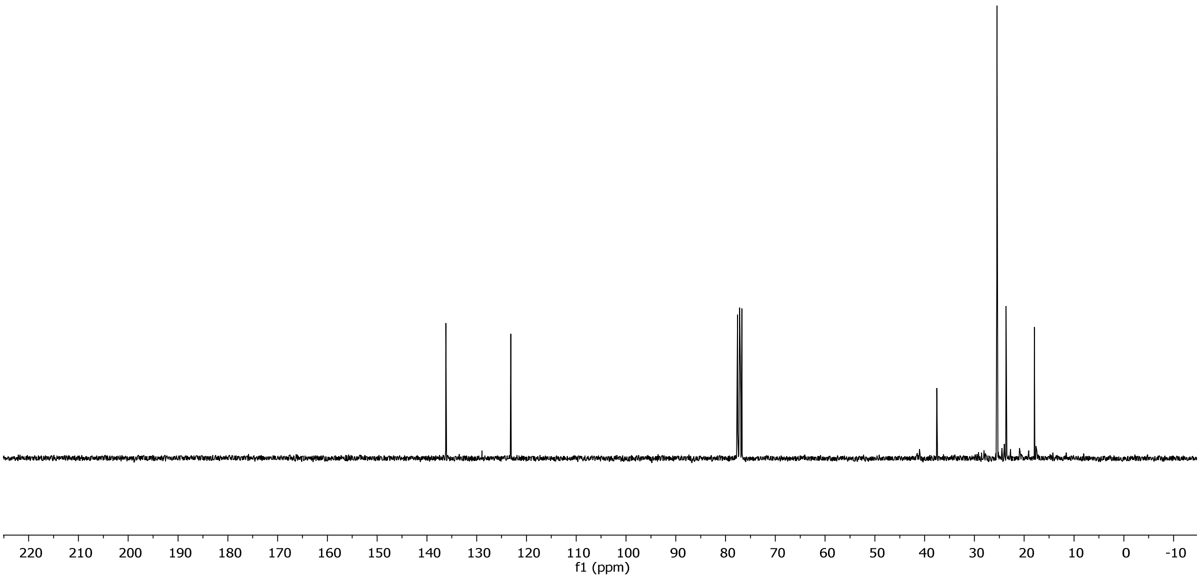

^{1}H NMR 300 MHz CDCl3

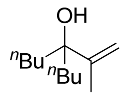

15

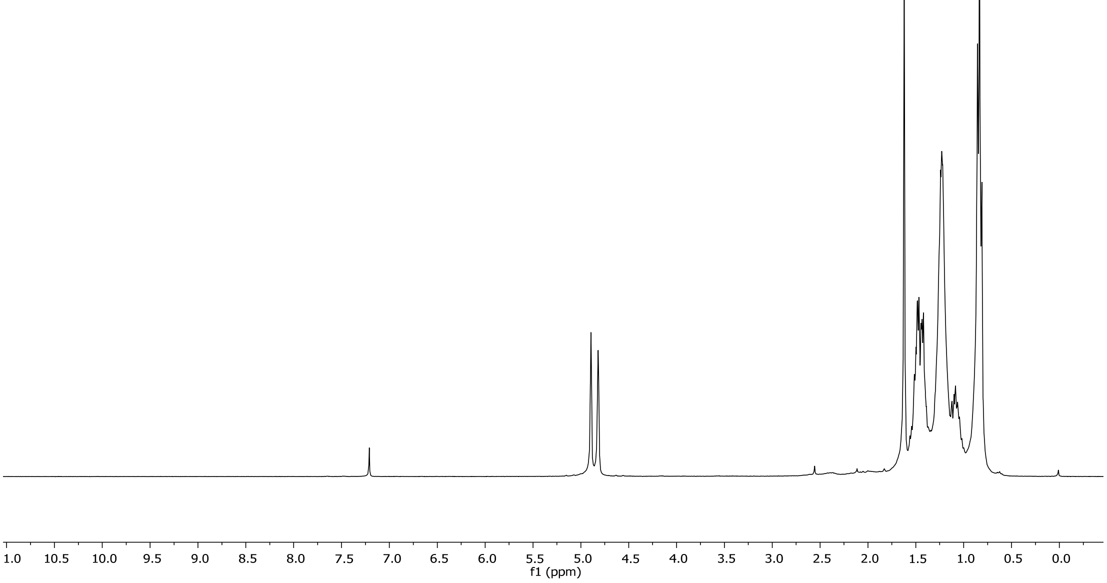

^{13}C NMR 300 MHz CDCl3

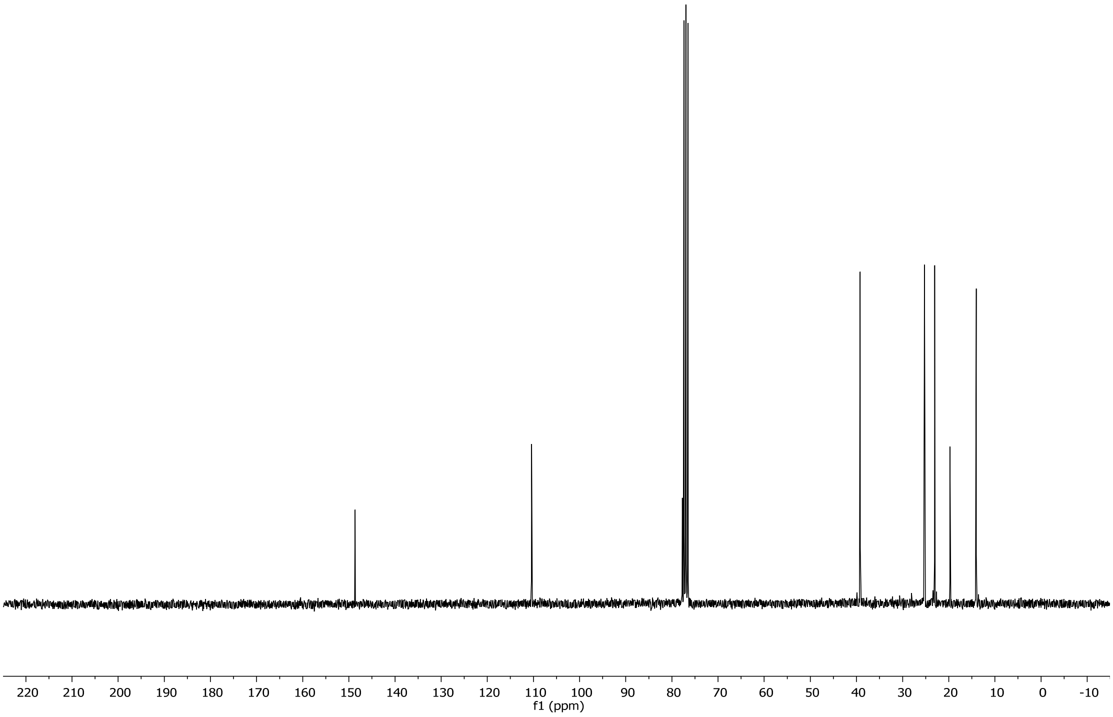

s5gha061
1H 300.1MHz Job 20639 Green Samantha L A061 CDCl3 24.9°C
*

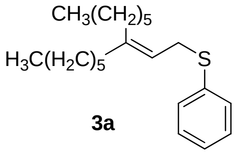

3a

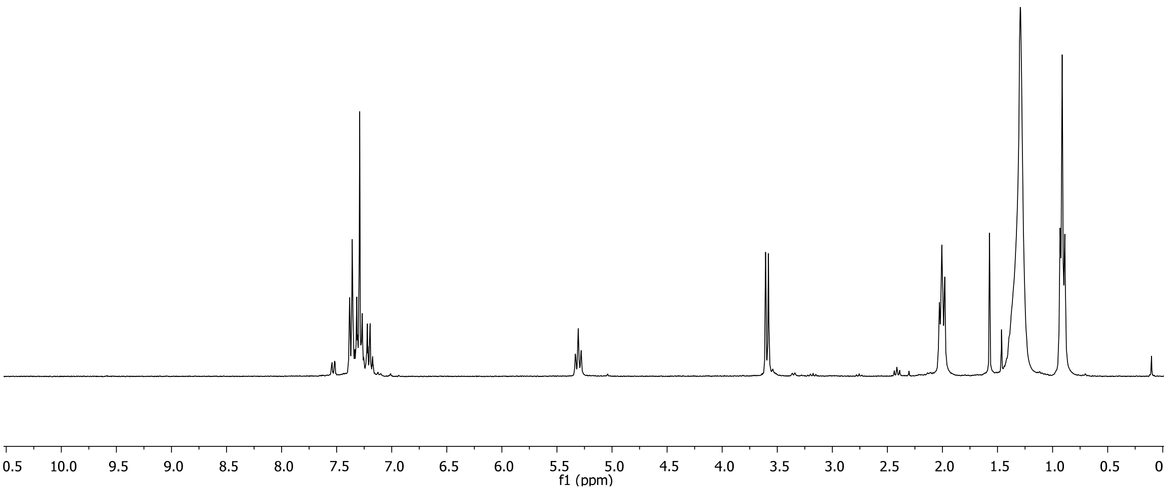

s5gca061
13C 75.5MHz Job 20876 Green Samantha L A061 CDCl3 24.9°C 3 hours 1 min
*

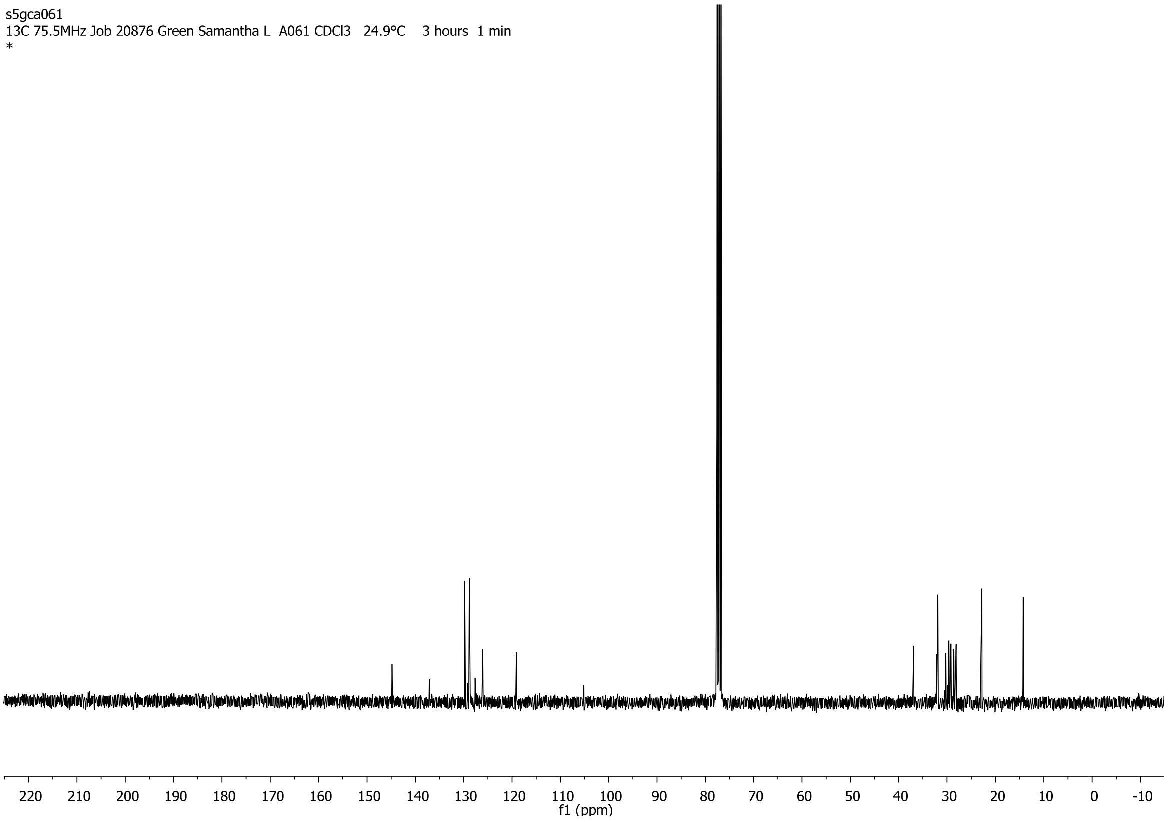

s5ghc129
1H 300.1MHz Job 22858 Green Samantha L C129 CDCl3   24.9°C
*

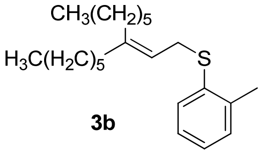

3b

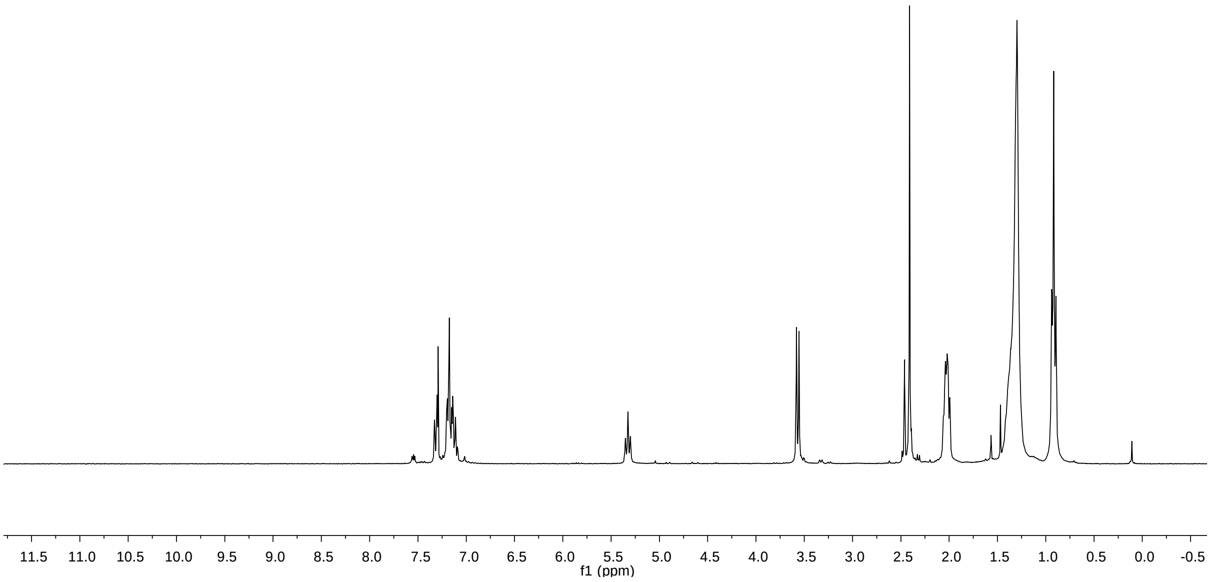

s5gcc129
13C 75.5MHz Job 22900 Green Samantha L C129 CDCl3   25.1°C   3 hours 1 min
*

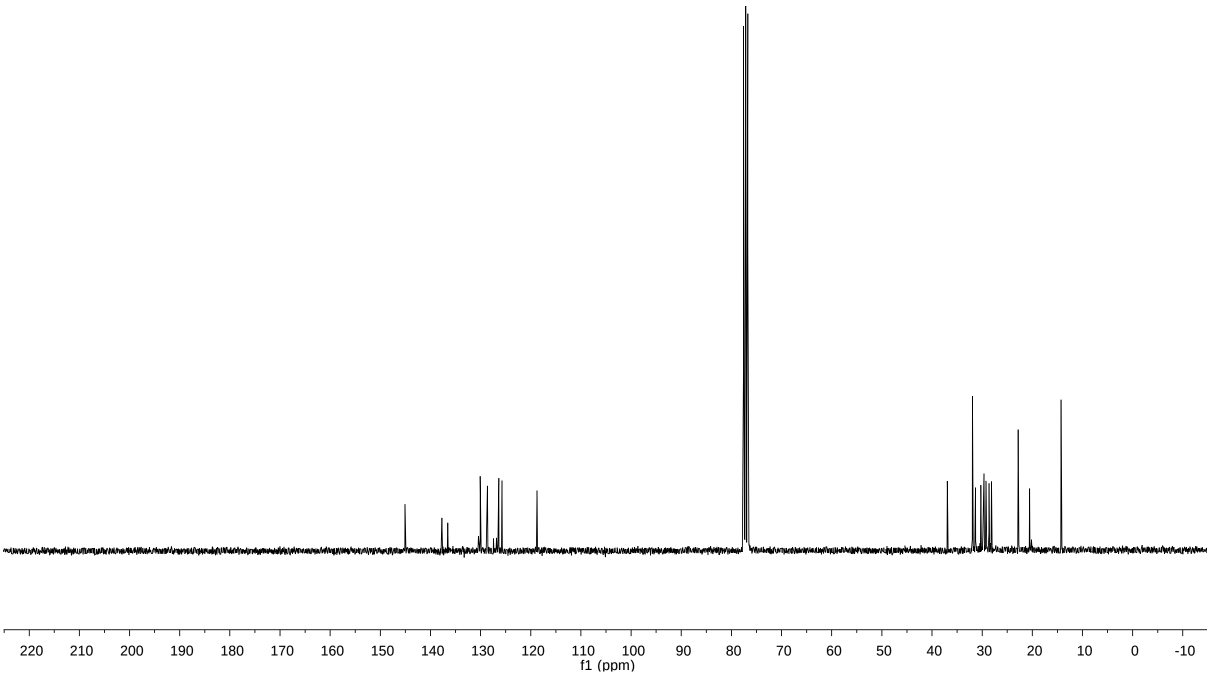

s5ghc143
1H 300.1MHz Job 23387 Green Samantha L C143 CDCl3   25.2°C
*

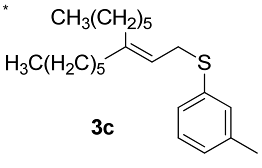

3c

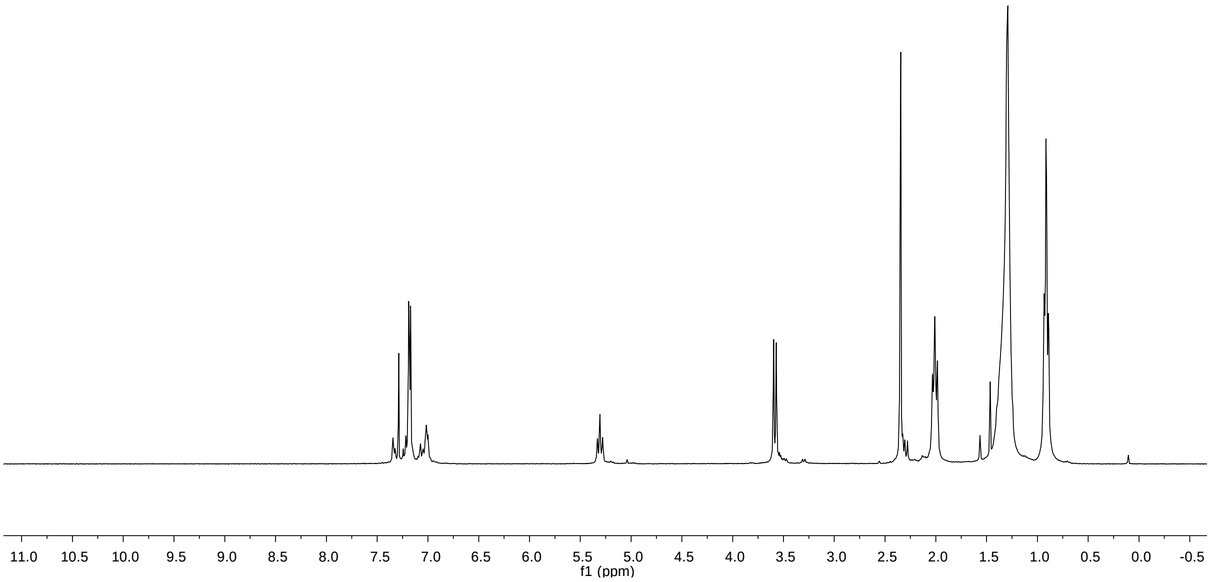

s5gcc143
13C 75.5MHz Job 23558 Green Samantha L C143 CDCl3   25.0°C   3 hours 1 min
*

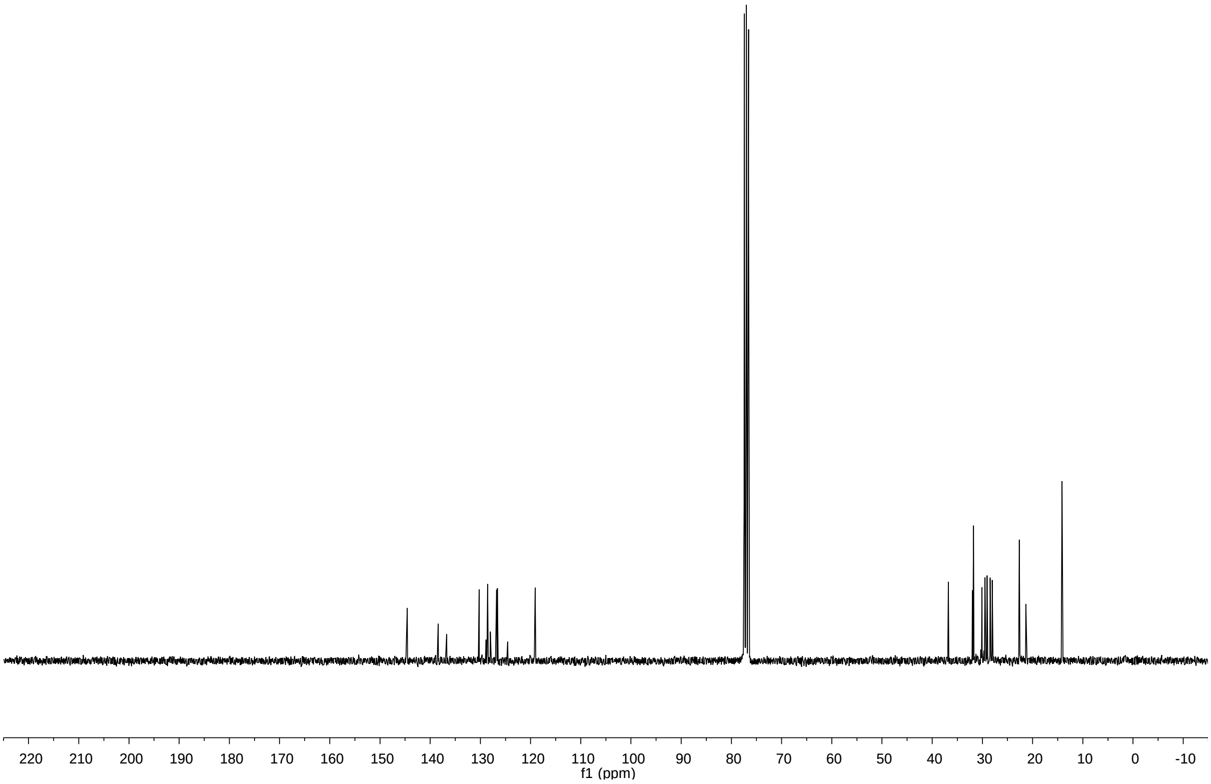

s5ghc139
1H 300.1MHz Job 23234 Green Samantha L C139 CDCl3   24.9°C
*

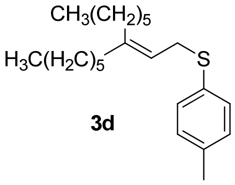

3d

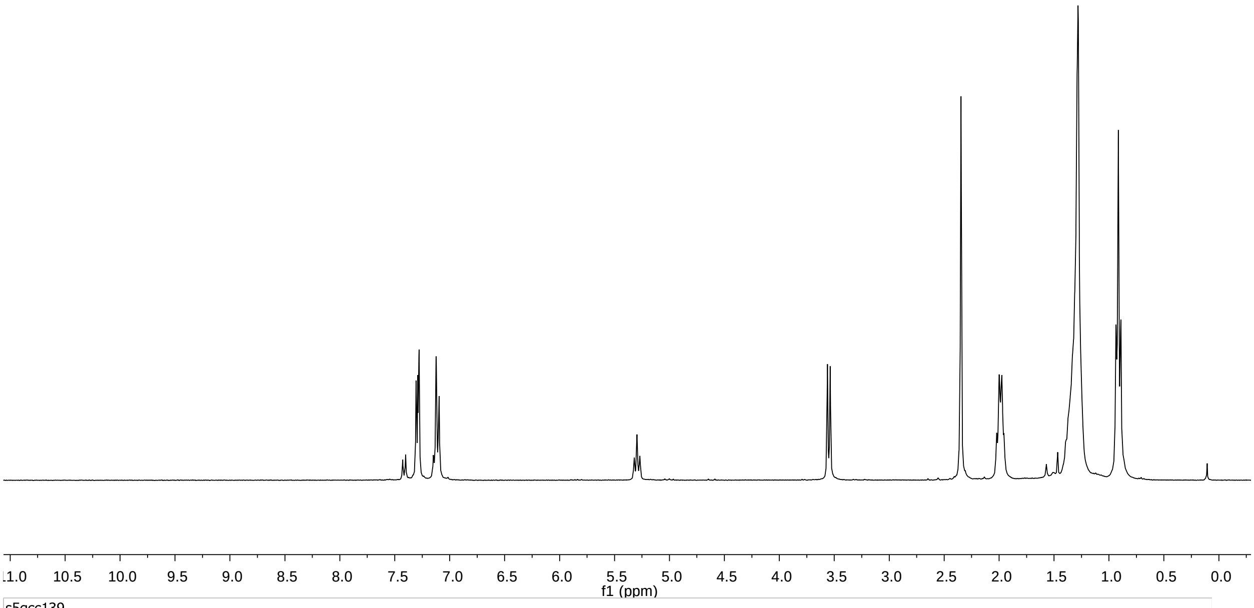

s5gcc139
13C 75.5MHz Job 23557 Green Samantha L C139 CDCl3   25.0°C   3 hours 1 min
*

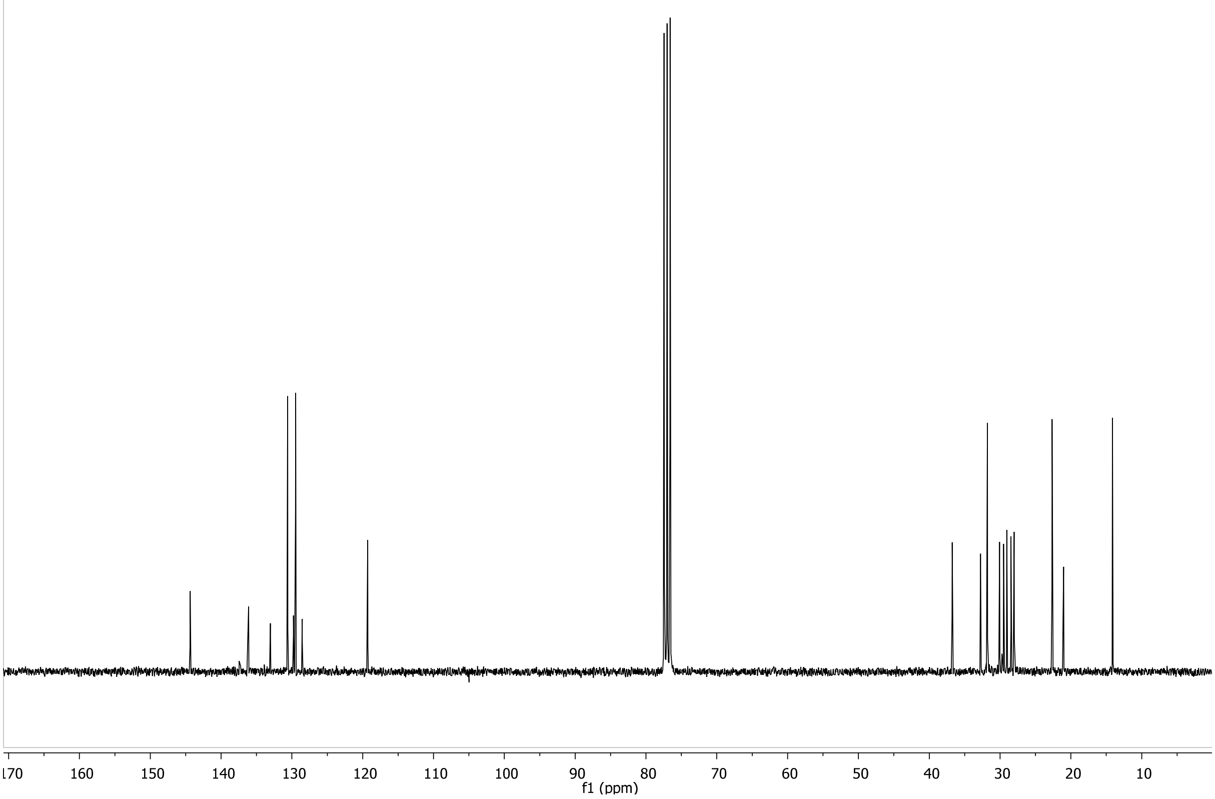

s5ghc147
1H 300.1MHz Job 23437 Green Samantha L C147 CDCl3   25.1°C
*

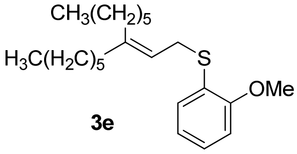

3e

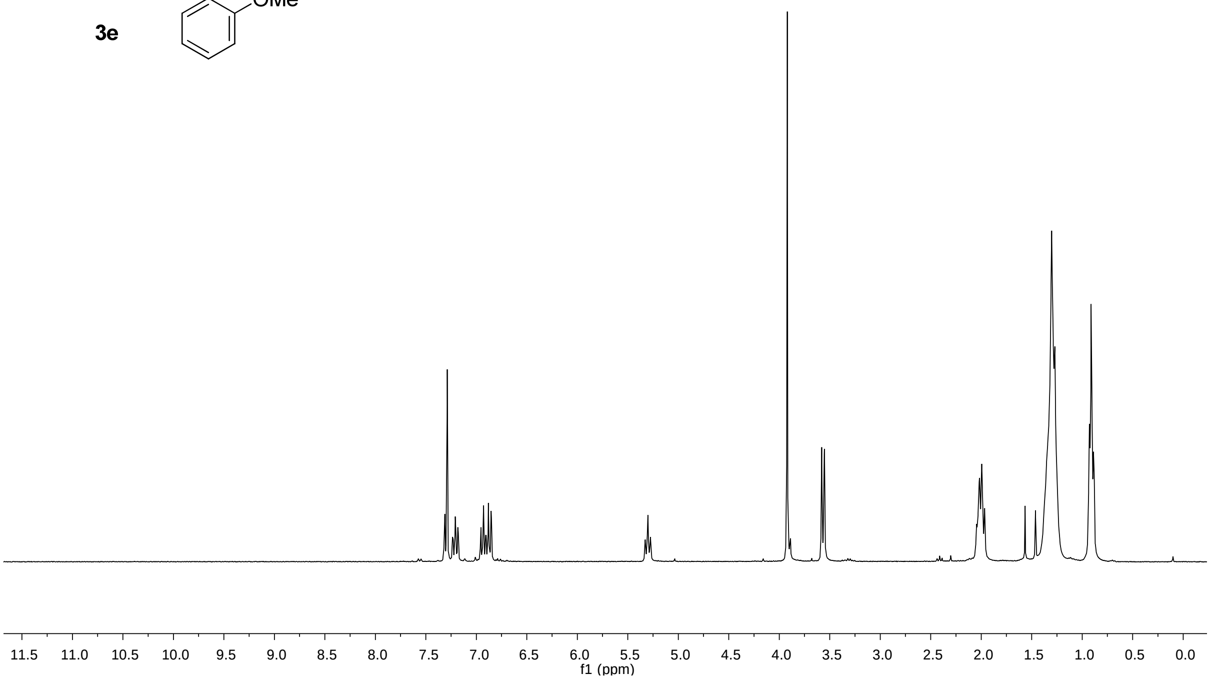

s5gcc147
13C 75.5MHz Job 23451 Green Samantha L C147 CDCl3   25.0°C   3 hours 1 min
*

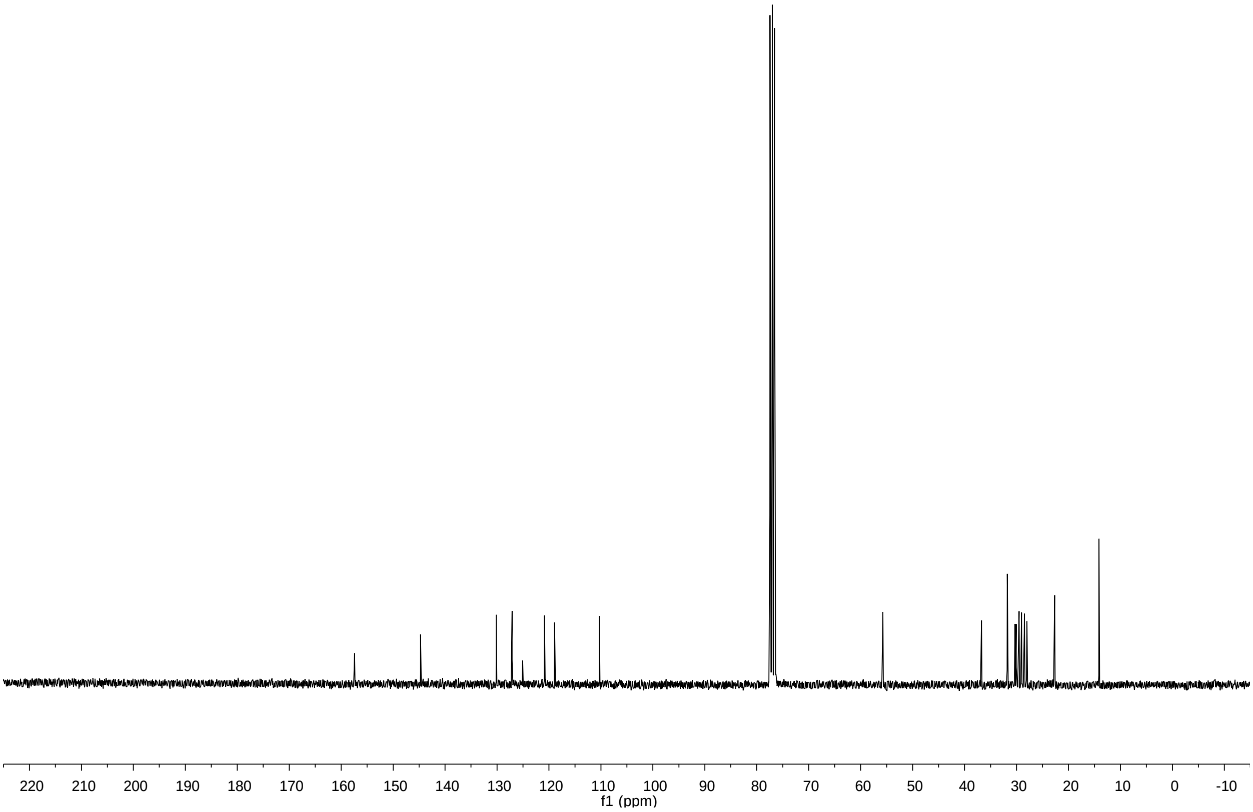

s5ghd119
1H 300.1MHz Job 22536 Green Samantha L D119 CDCl3   24.9°C
ISOLATED

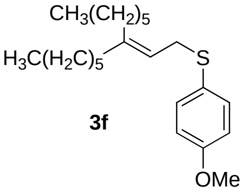

3f

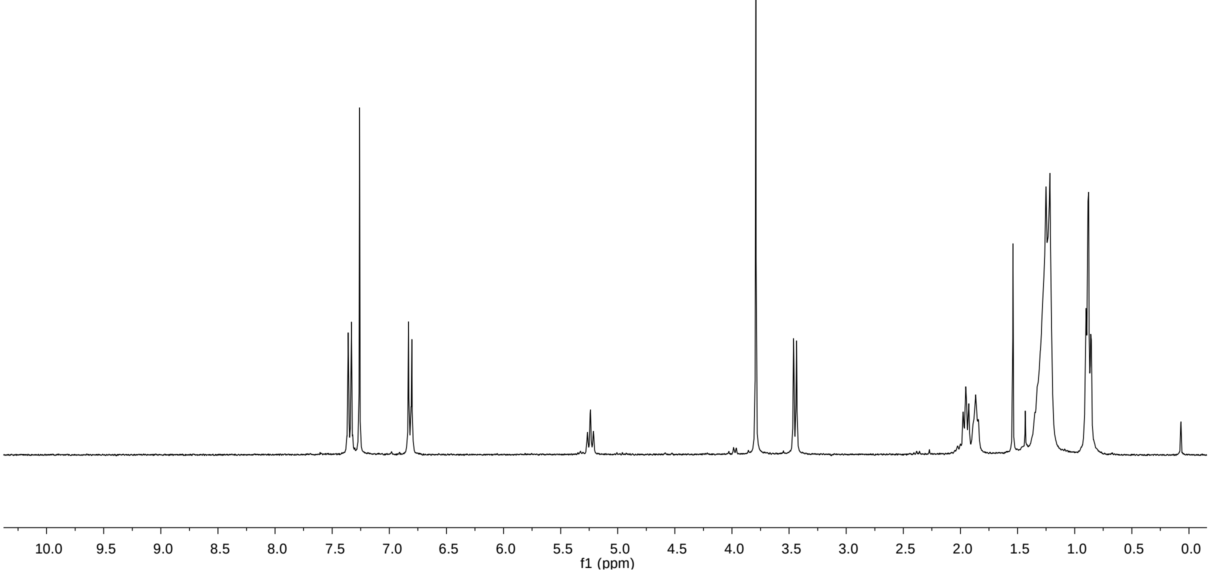

s5gcd119
13C 100.6MHz Job 20011 Green Samantha L D119 CDCl3   25.0°C   16 hours 2 min
*

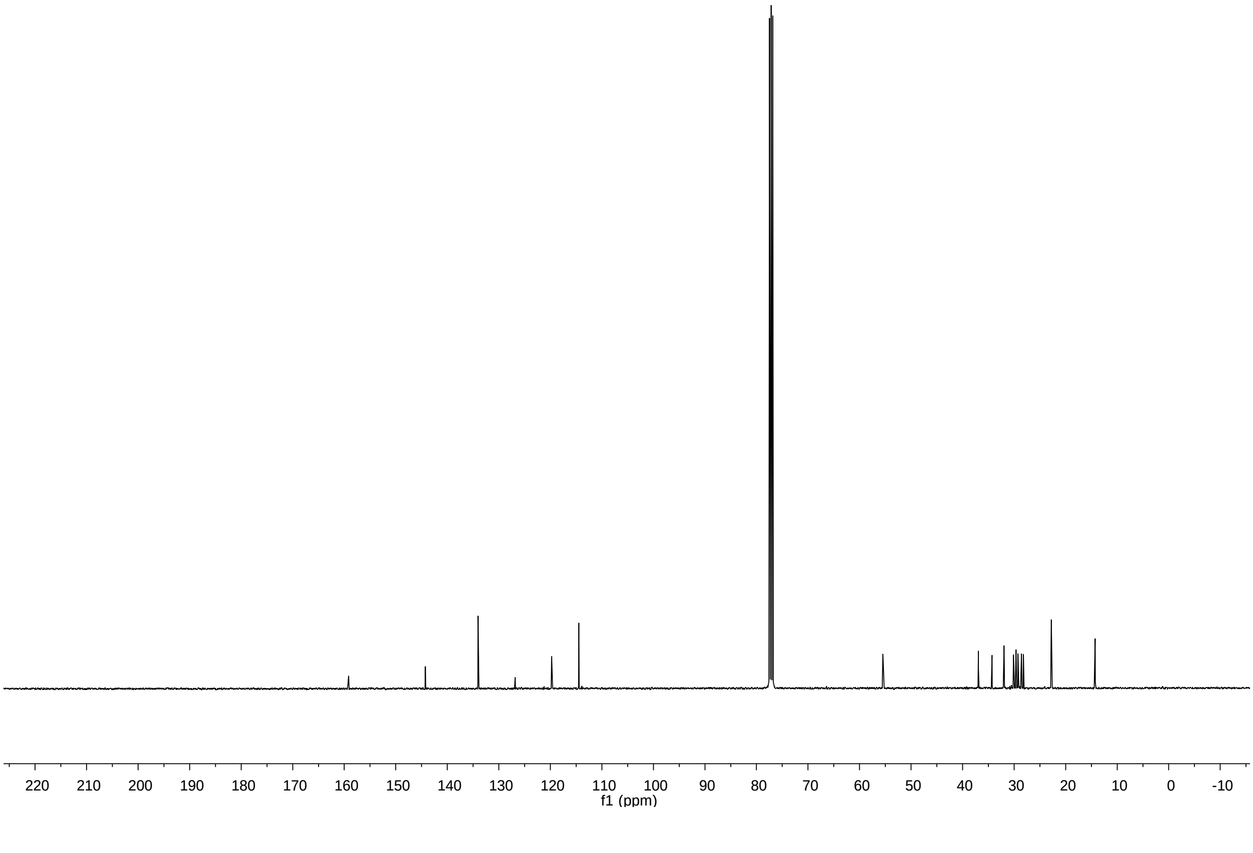

s5ghd149
1H 300.1MHz Job 23489 Green Samantha L D149 CDCl3   24.8°C
*

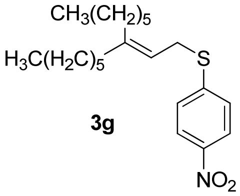

3g

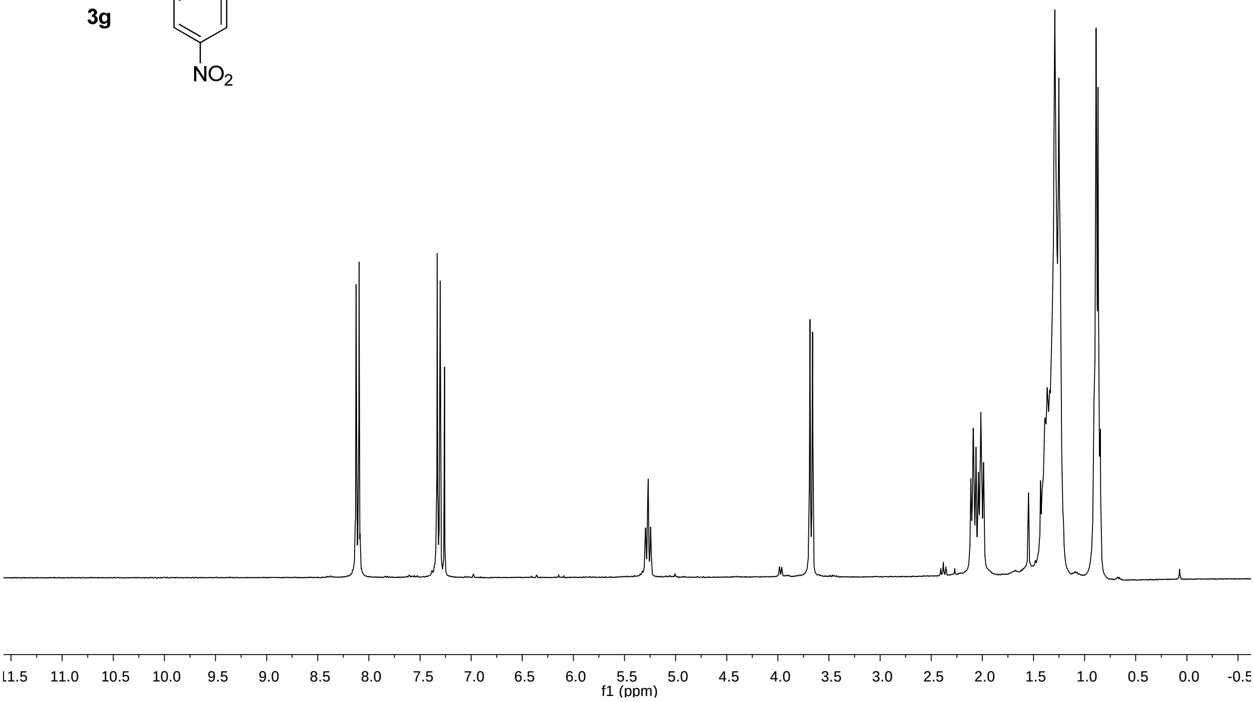

s5gcd149
13C 75.5MHz Job 23559 Green Samantha L D149 CDCl3   25.0°C   3 hours 1 min
*

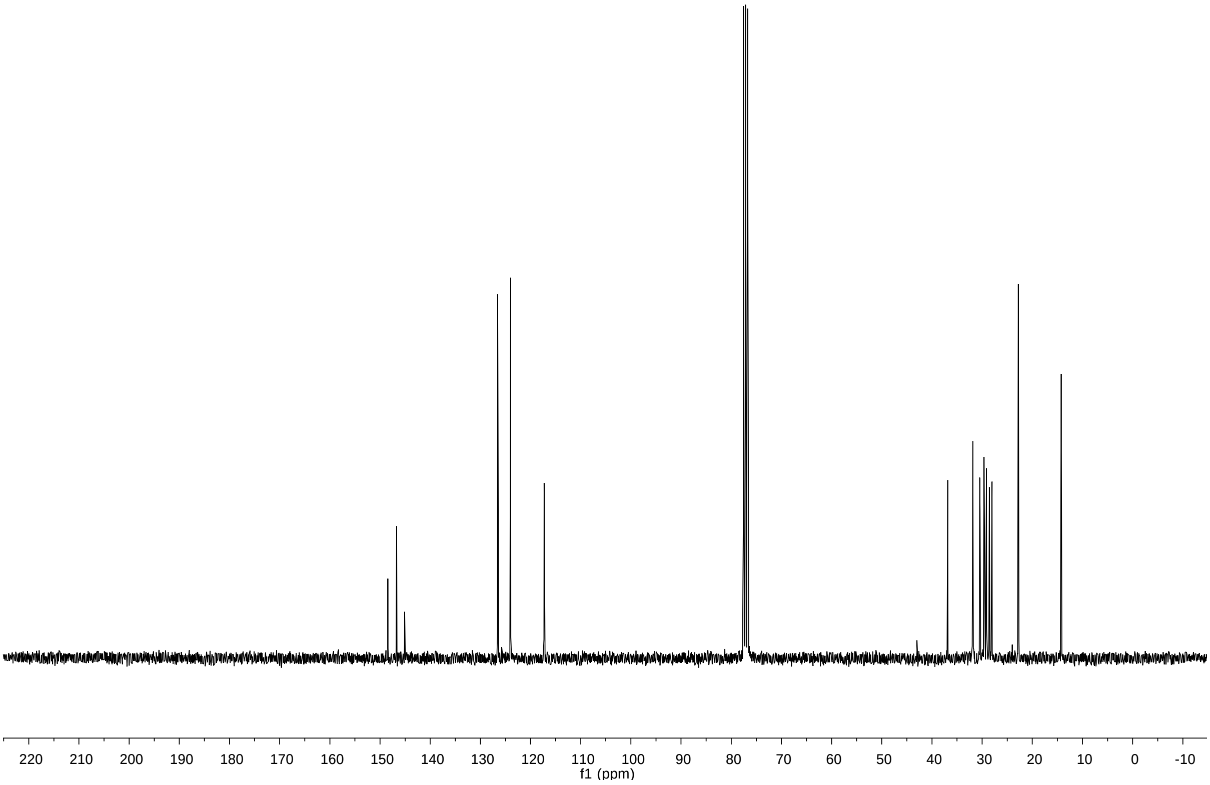

s5ghb145
1H 300.1MHz Job 23414 Green Samantha L B145 CDCl3 25.1°C
*

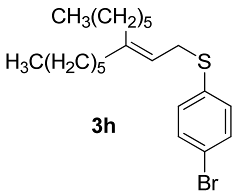

3h

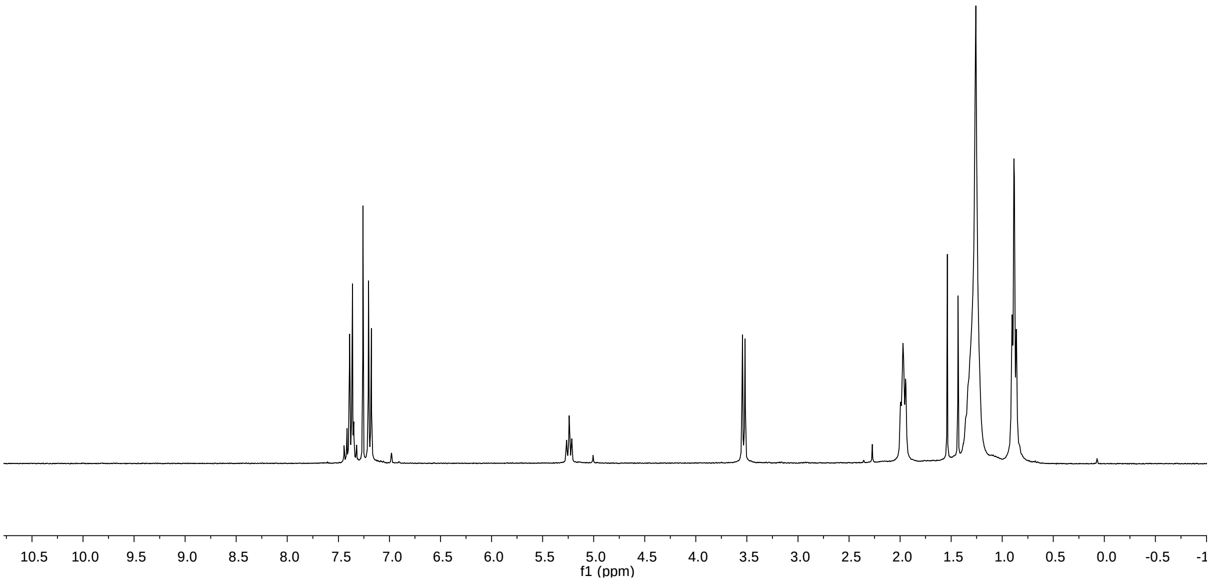

s5gcb145
13C 75.5MHz Job 23452 Green Samantha L B145 CDCl3 25.0°C 3 hours 1 min
*

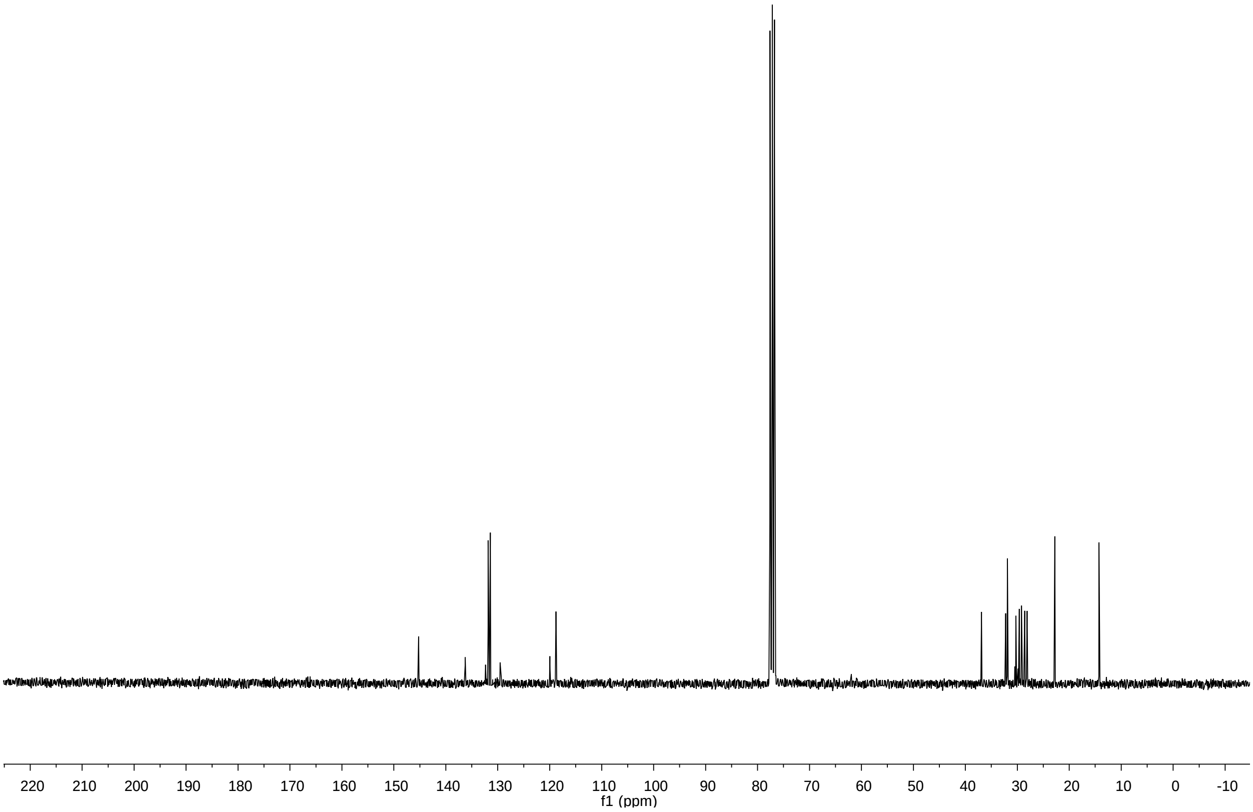

s5ghc153
1H 300.1MHz Job 23699 Green Samantha L C153 CDCl3 25.1°C
*

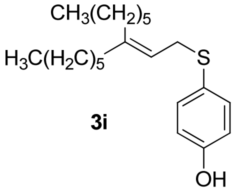

3i

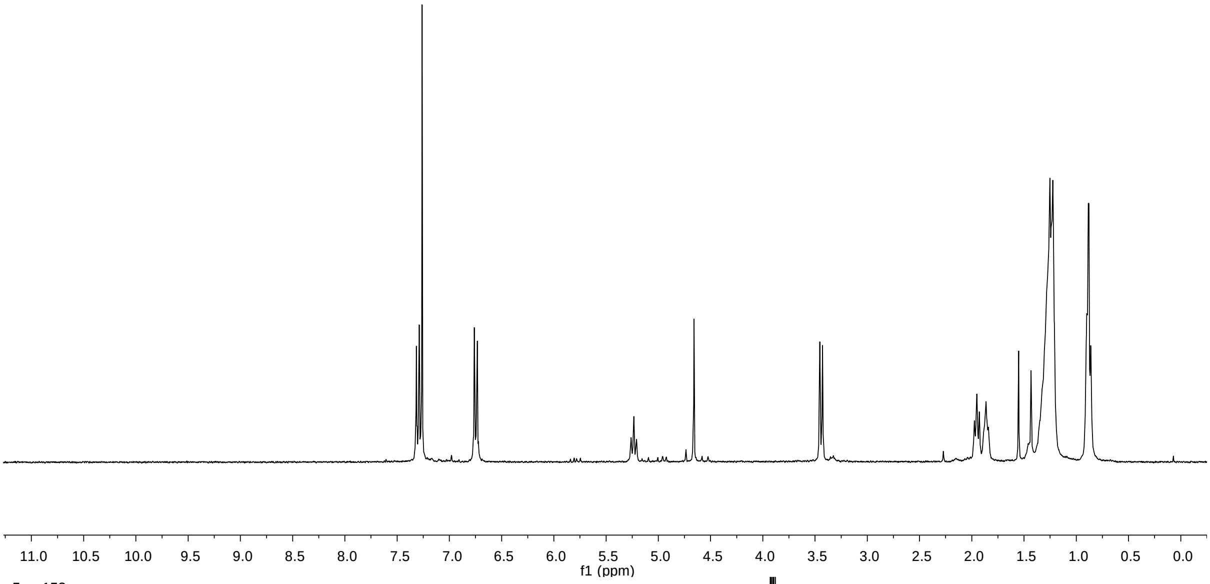

s5gcc153
13C 75.5MHz Job 23769 Green Samantha L C153 CDCl3 25.0°C 3 hours 1 min
*

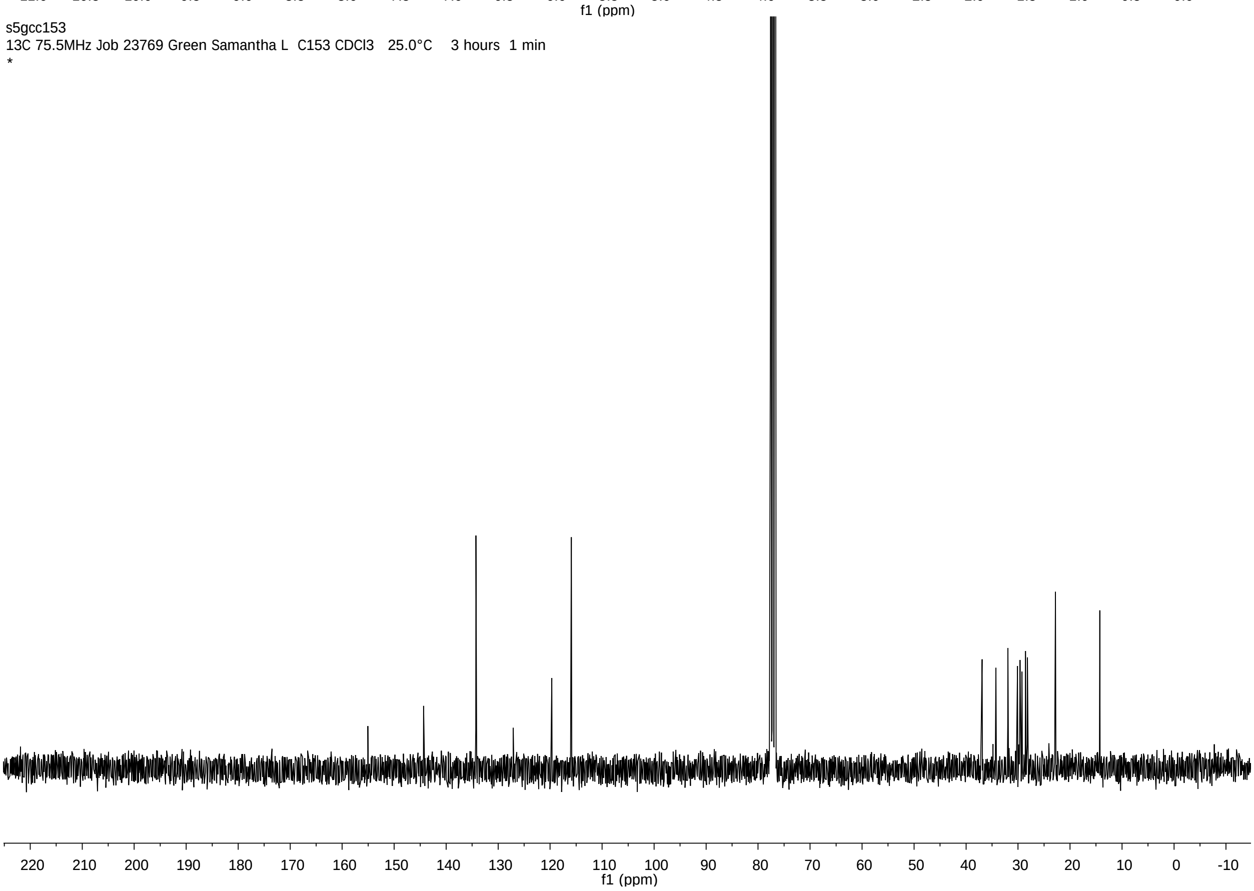

s5ghg083
1H 300.1MHz Job 21456 Green Samantha L G083 CDCl3 25.0°C
*

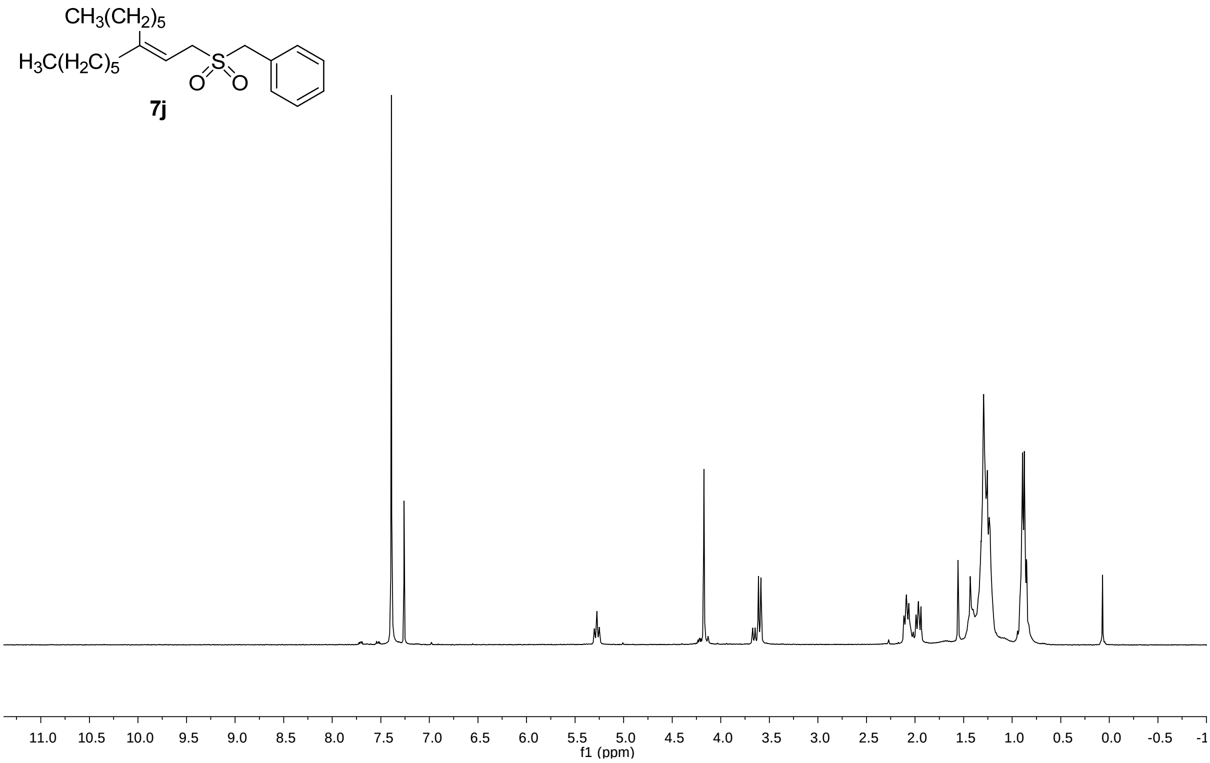

s5gca059
13C 75.5MHz Job 20779 Green Samantha L A059 CDCl3 25.0°C 3 hours 1 min
*

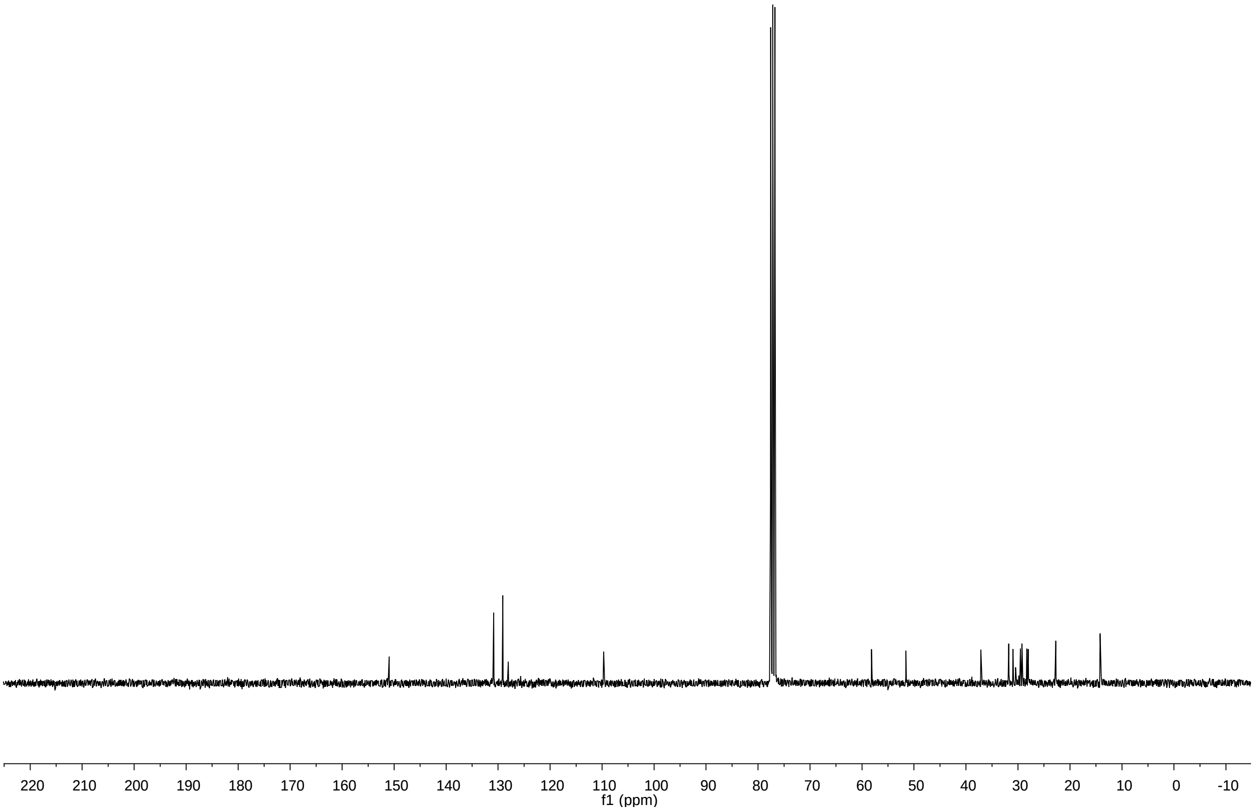

lrhhc0281H 300.1MHz Job 27242 Herkert Lorena C028 CDCl3 24.9°C
*

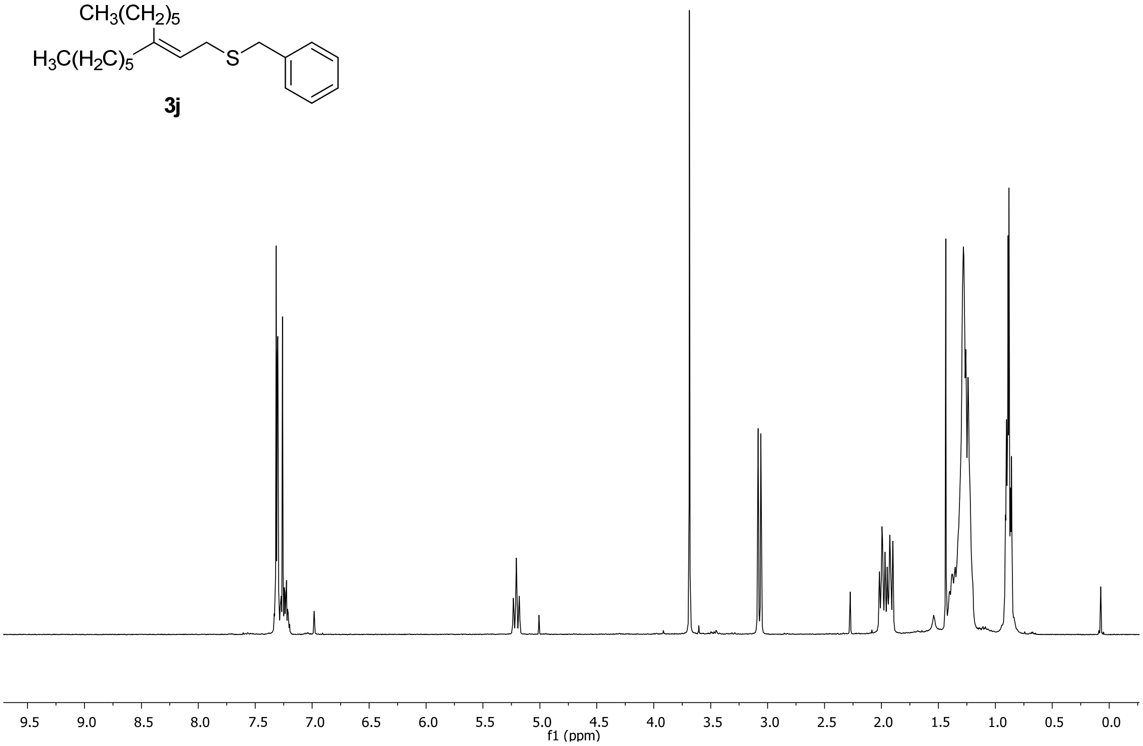

lrhcd02813C 75.5MHz Job 27342 Herkert Lorena D028 CDCl3 25.0°C 3 hours 1 min
*

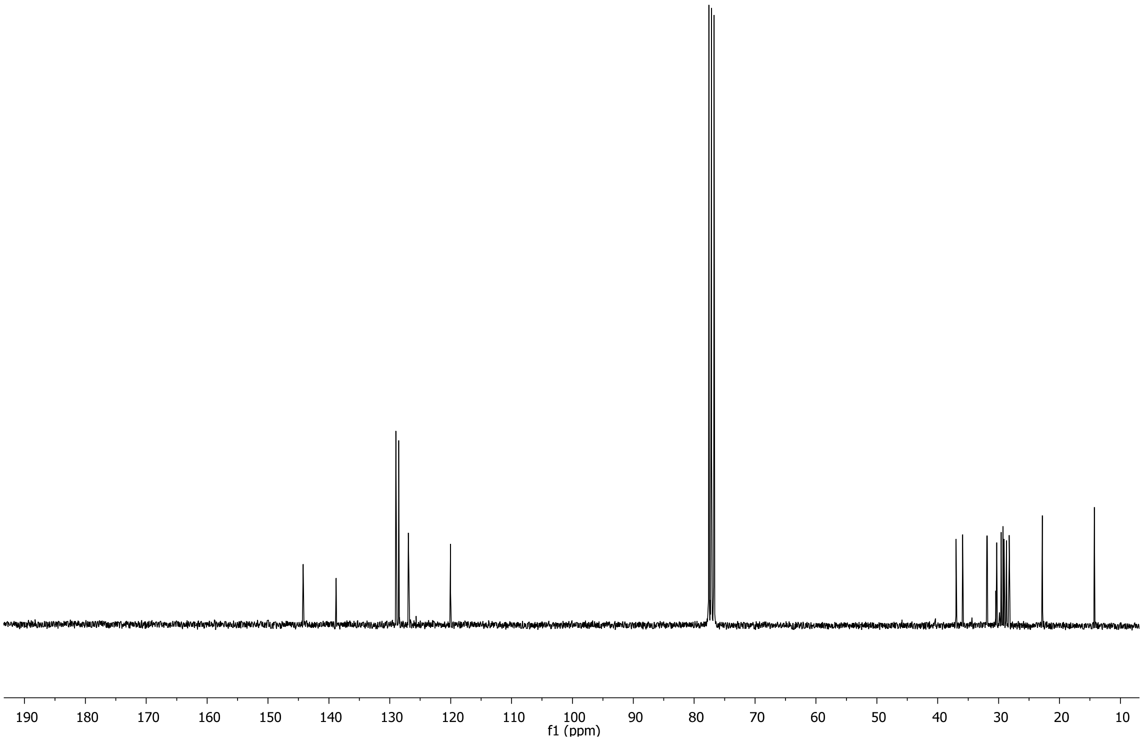

lrhhe0341H 300.1MHz Job 27602 Herkert Lorena E034 CDCl3 24.9°C
*

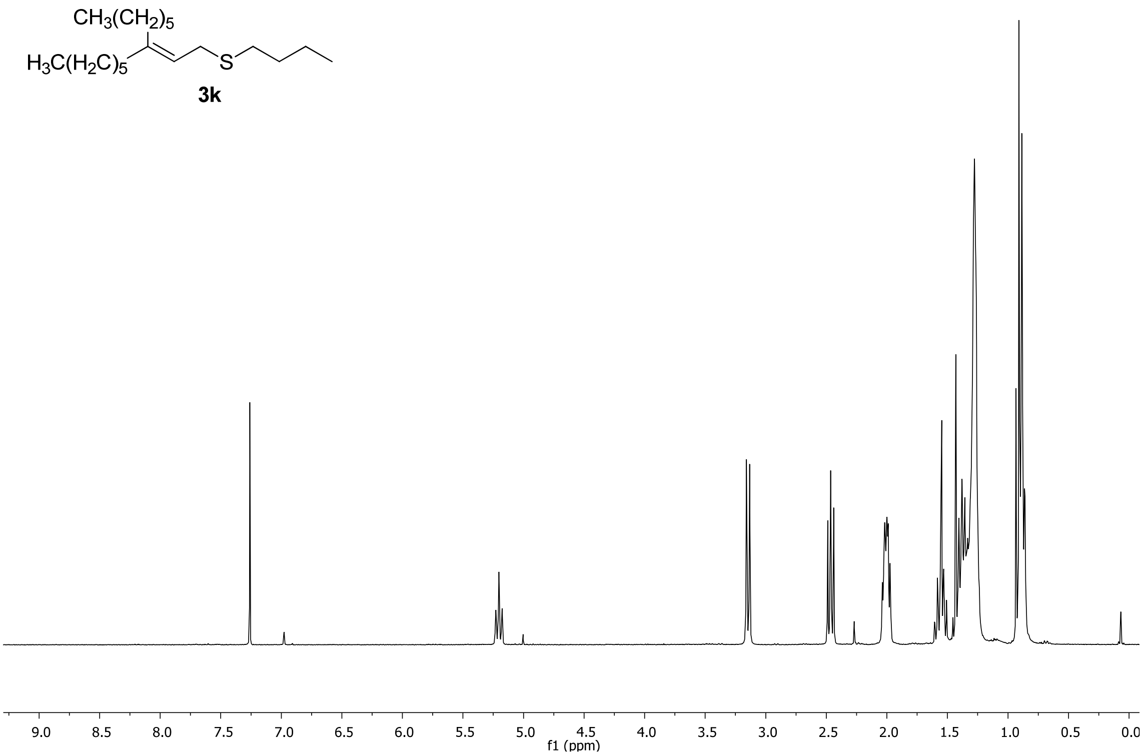

lrhce03413C 75.5MHz Job 27662 Herkert Lorena E034 CDCl3 25.0°C 3 hours 1 min
*

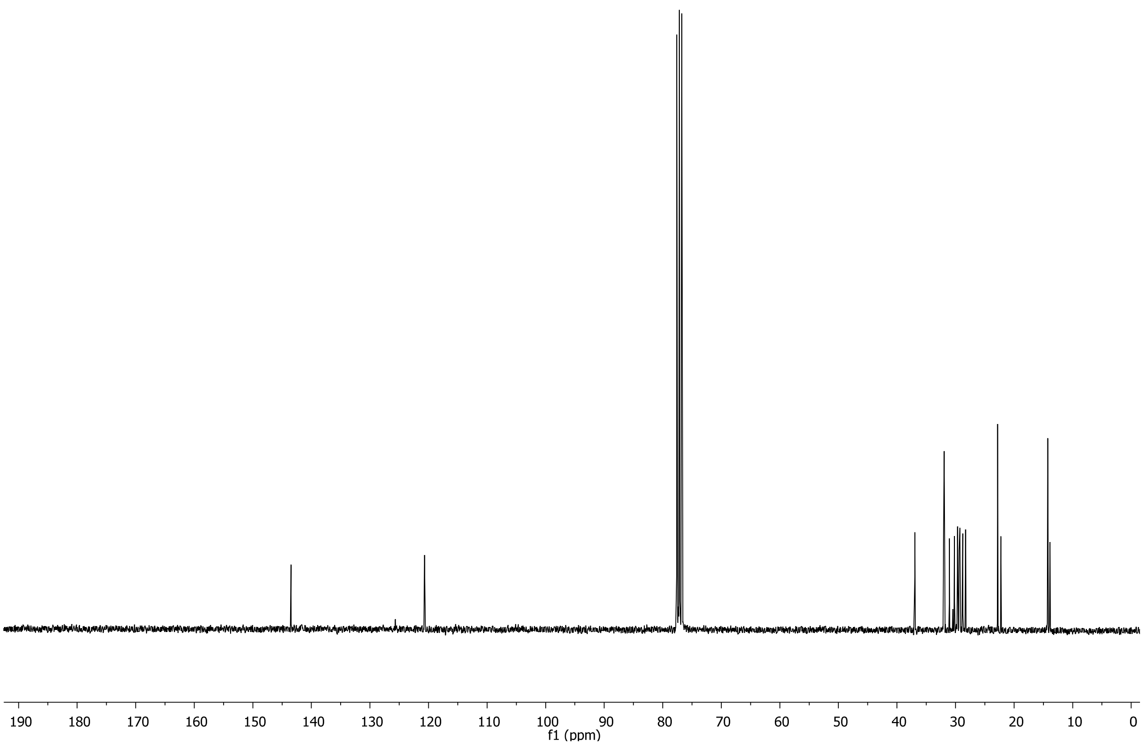

lrhhc0261H 300.1MHz Job 27181 Herkert Lorena C026 CDCl3 24.9°C
*

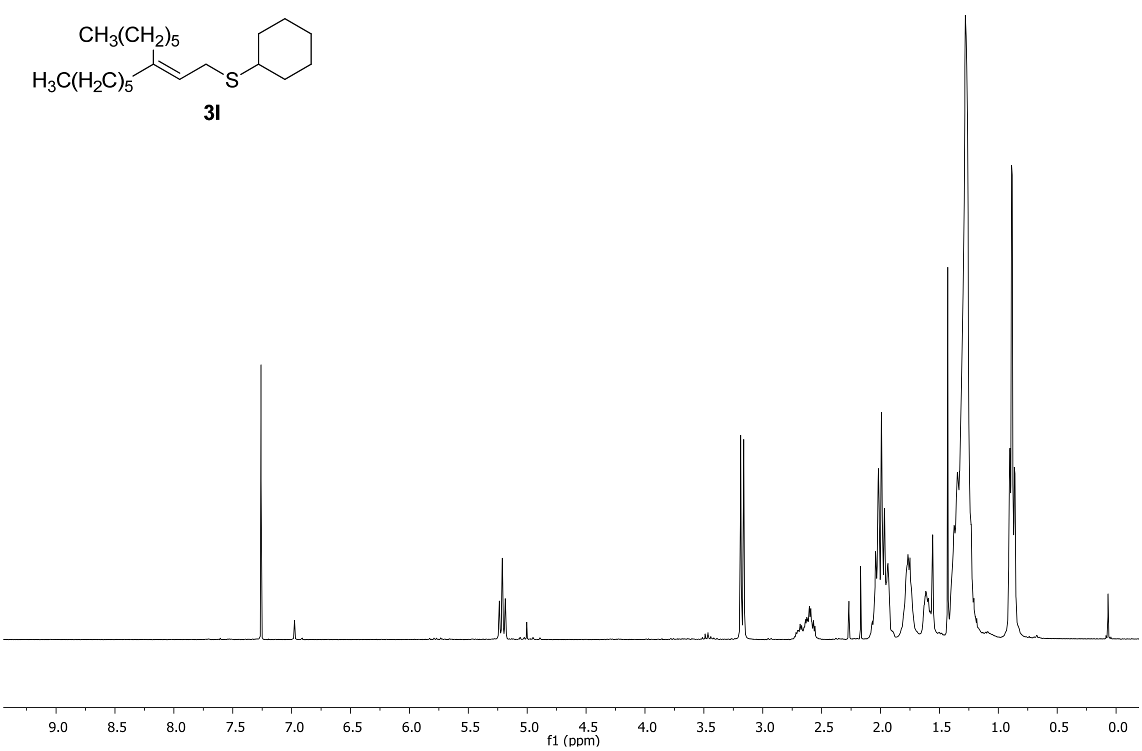

lrhcb02213C 75.5MHz Job 27290 Herkert Lorena B022 CDCl3 25.0°C 3 hours 1 min
*

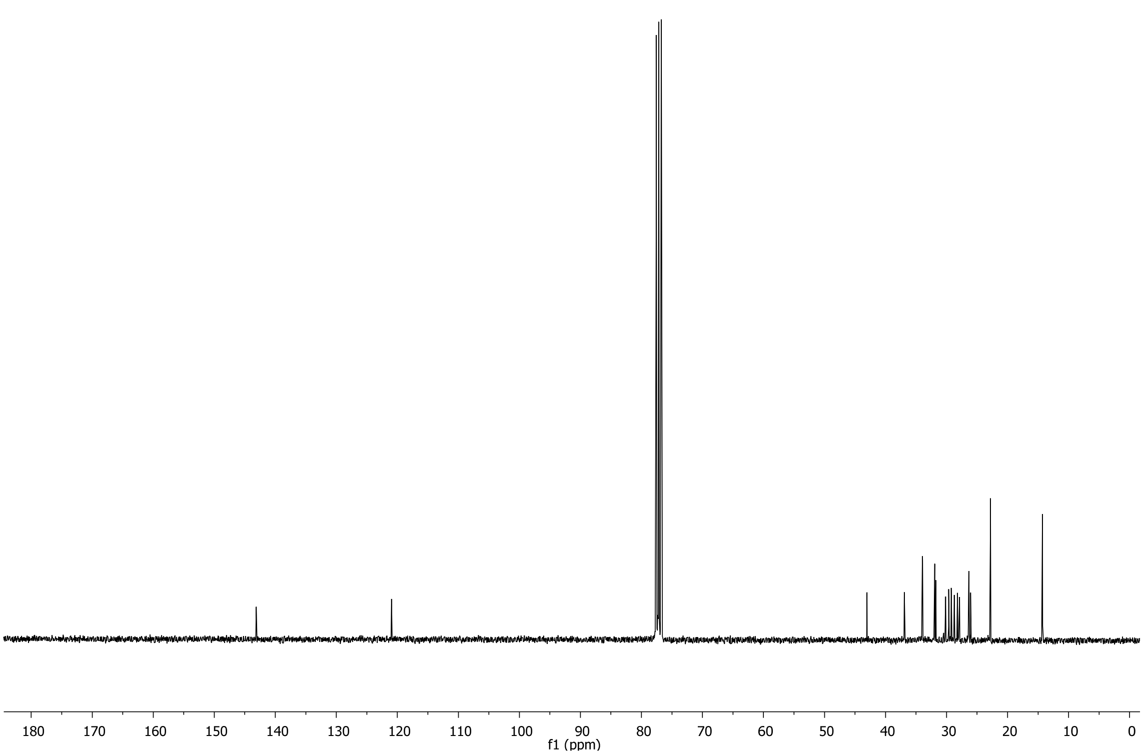

lrhhg0391H 300.1MHz Job 27926 Herkert Lorena G039 CDCl3 24.9°C
*

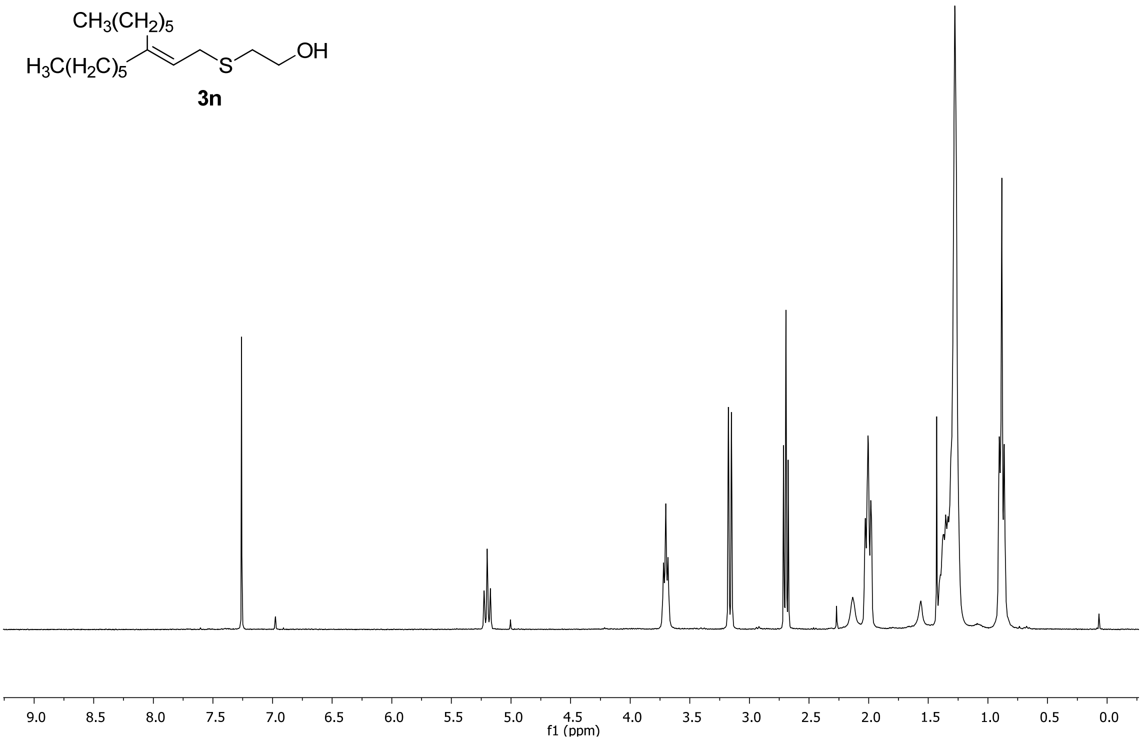

lrhcg03913C 75.5MHz Job 27944 Herkert Lorena G039 CDCl3 25.0°C 3 hours 1 min
*

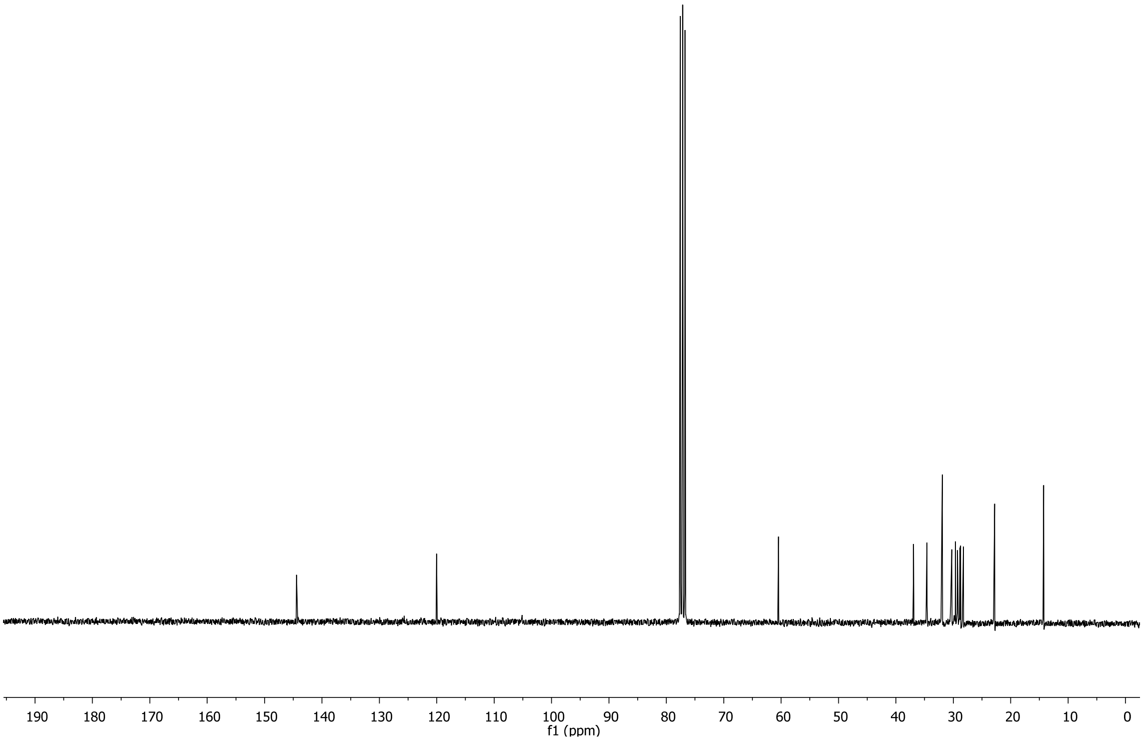

lrhhd046
1H 300.1MHz Job 28675 Herkert Lorena D046 CDCl3 25.1°C
*

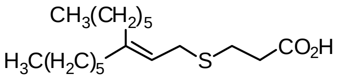

3o

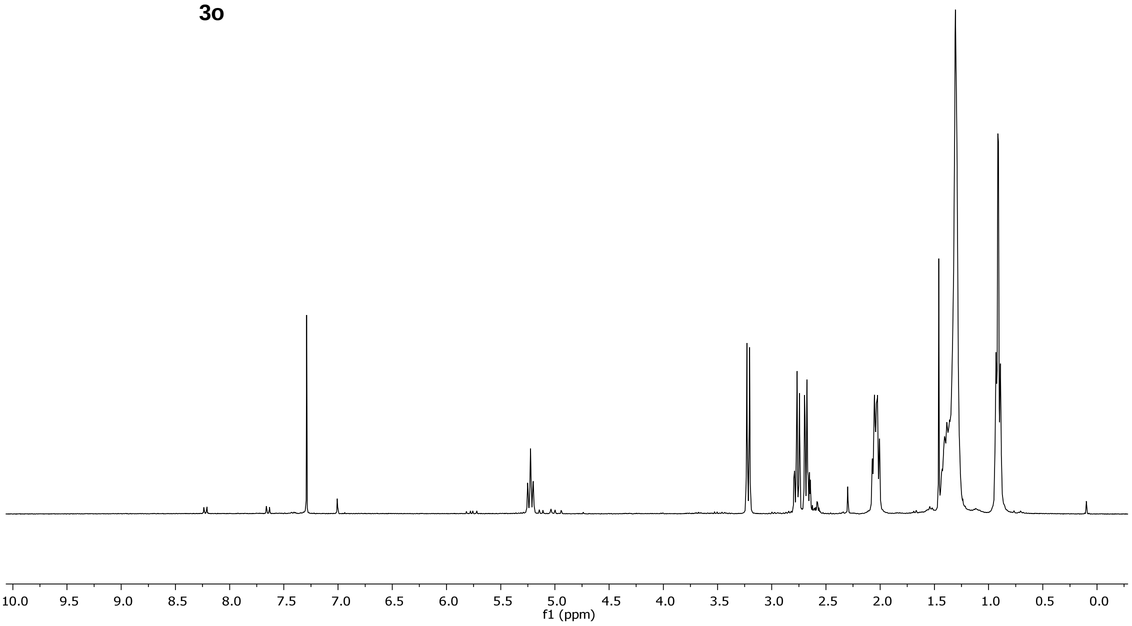

lrhcd046
13C 75.5MHz Job 28720 Herkert Lorena D046 CDCl3 25.0°C 3 hours 1 min
*

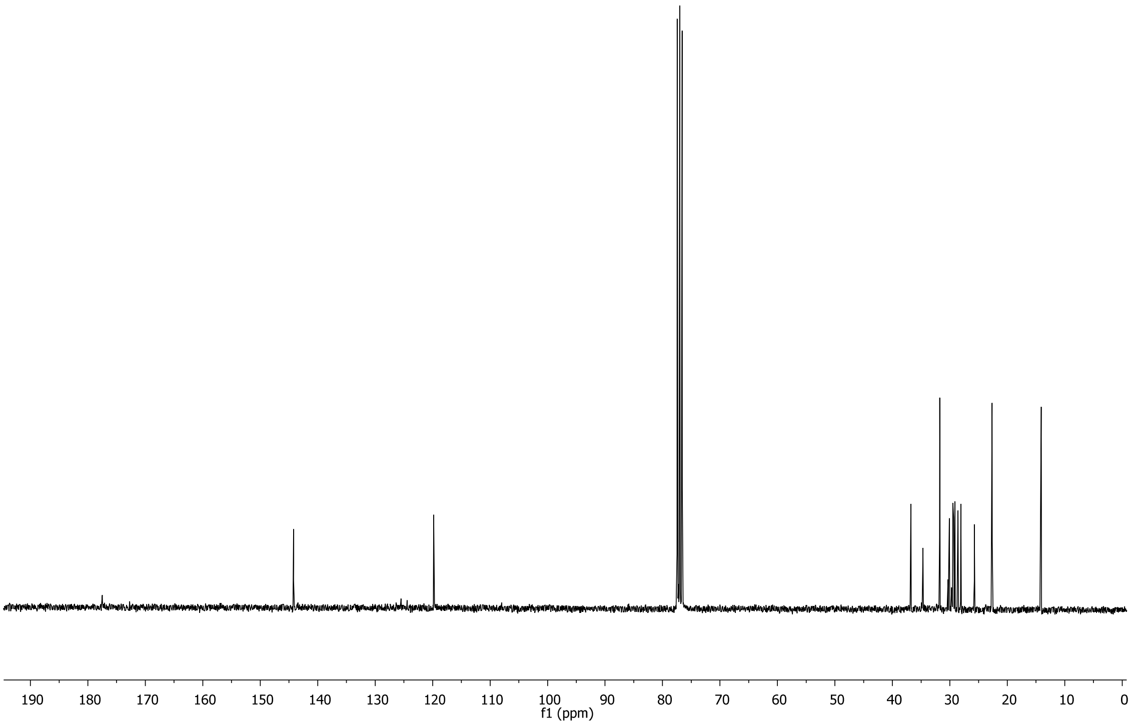

lrhhf0011H 300.1MHz Job 26061 Herkert Lorena F001 CDCl3 25.1°C
*

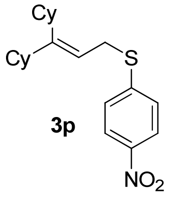

3p

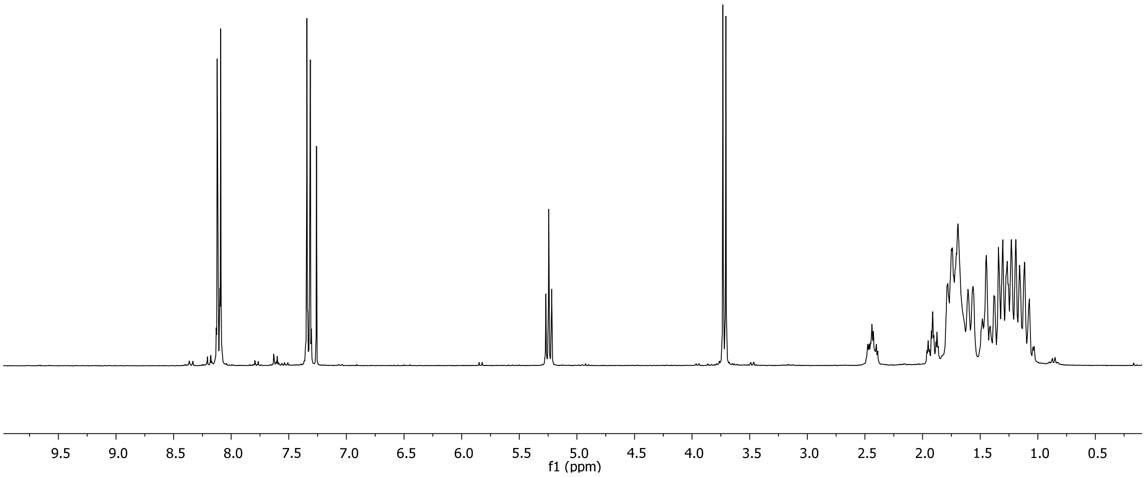

lrhcg00113C 75.5MHz Job 26178 Herkert Lorena G001 CDCl3 25.0°C 2 hours 43 min
*

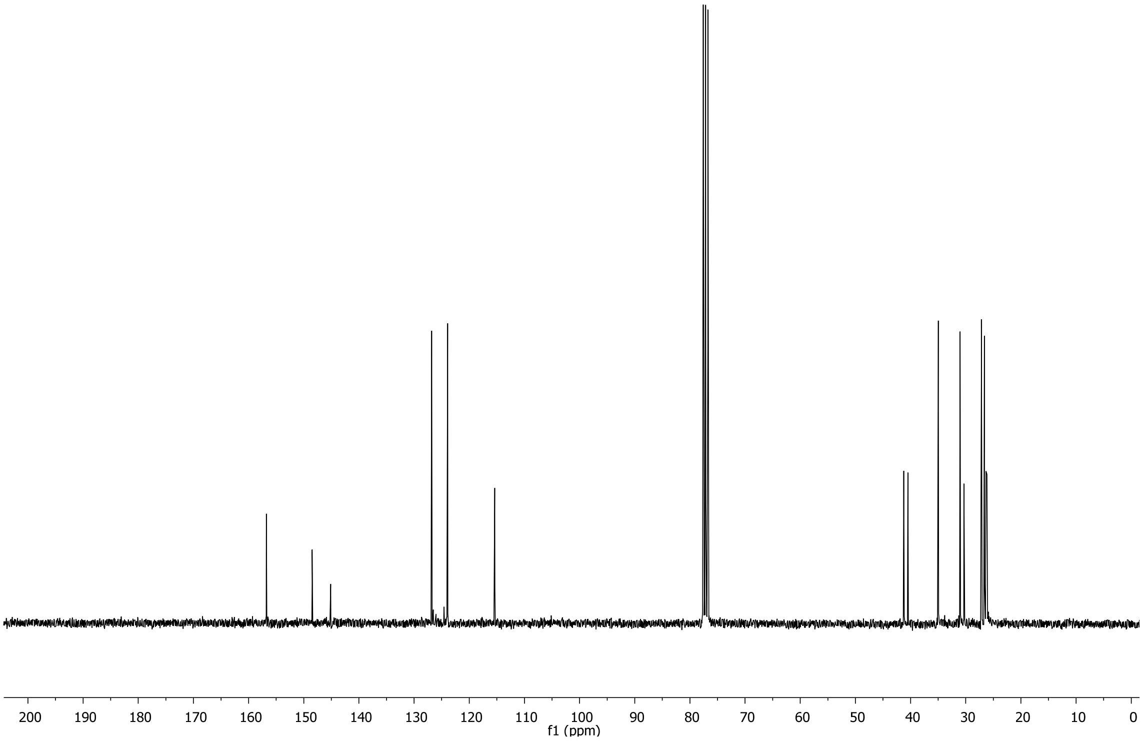

lrhhc0021H 300.1MHz Job 26031 Herkert Lorena C002 CDCl3 25.0°C
*

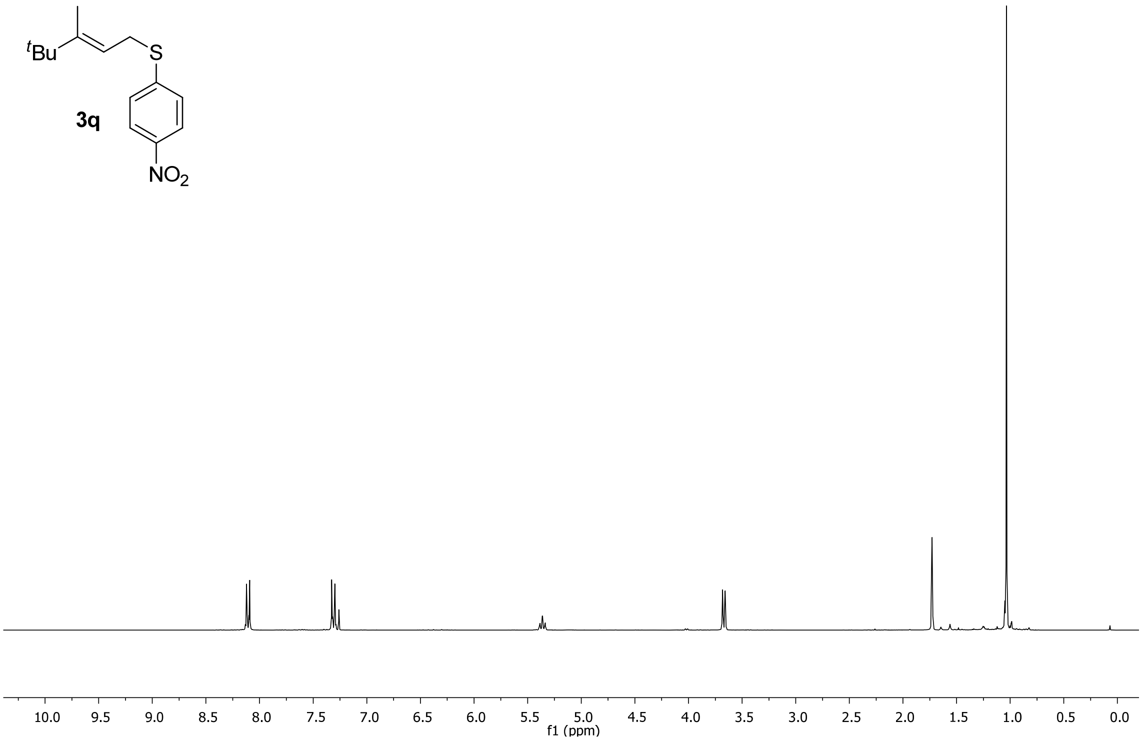

lrhce00213C 75.5MHz Job 26332 Herkert Lorena E002 CDCl3 25.0°C 0 hour 54 min
*

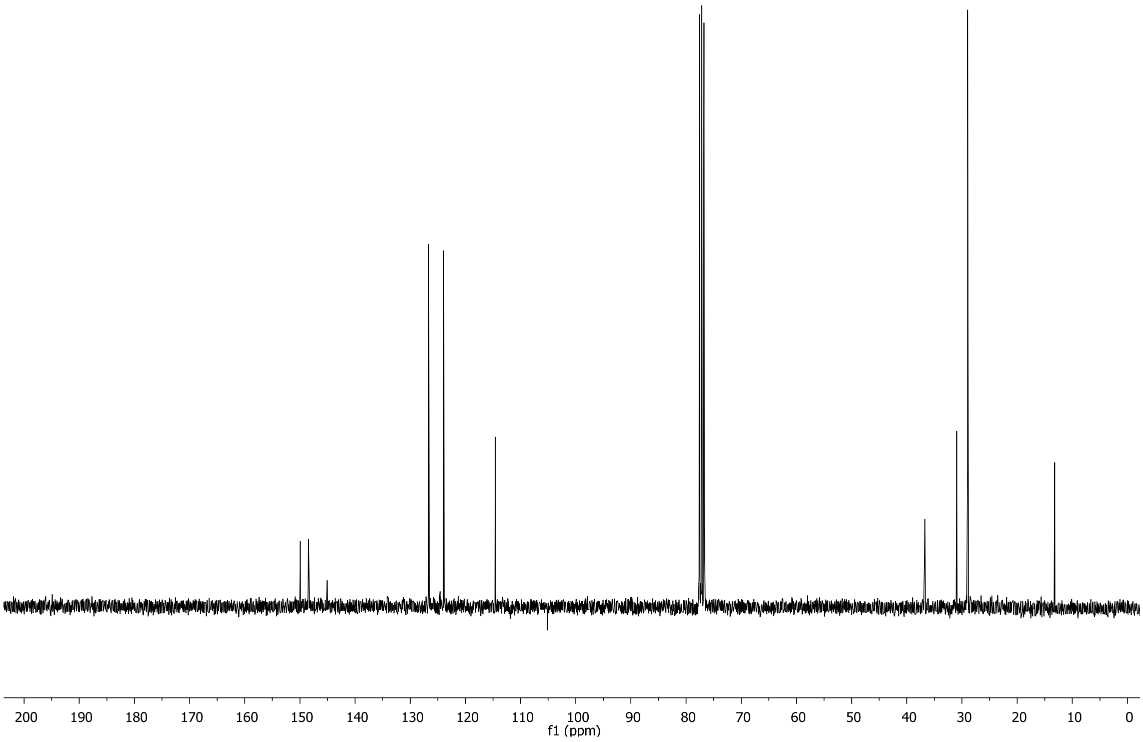

lrhhb0031H 300.1MHz Job 26022 Herkert Lorena B003 CDCl3   25.0°C
*

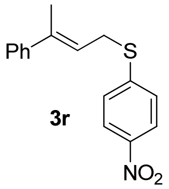

3r

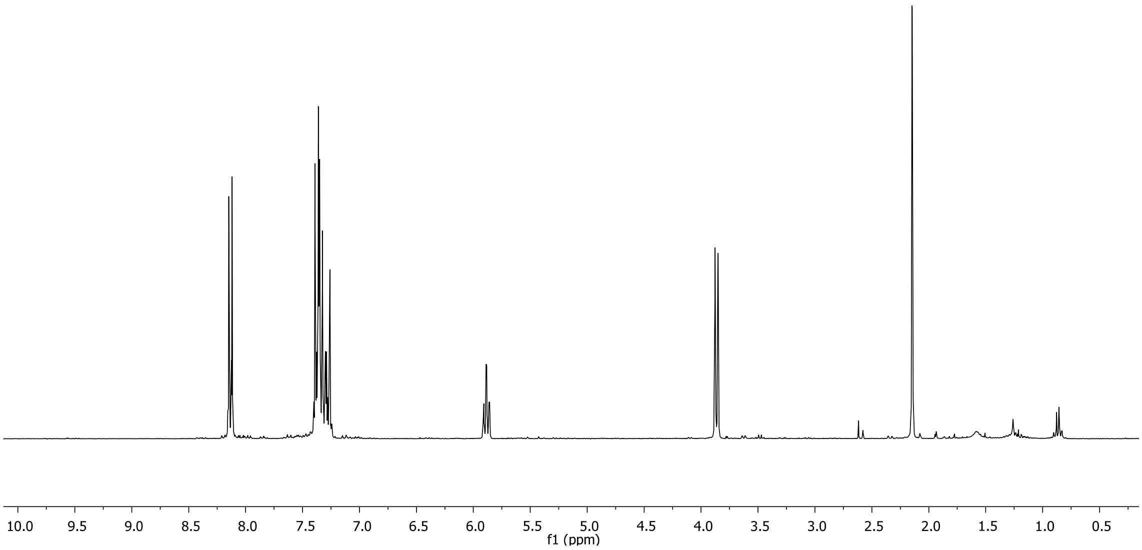

lrhcb003113C 75.5MHz Job 26129 Herkert Lorena B0031 CDCl3   25.0°C   3 hours 1 min
*

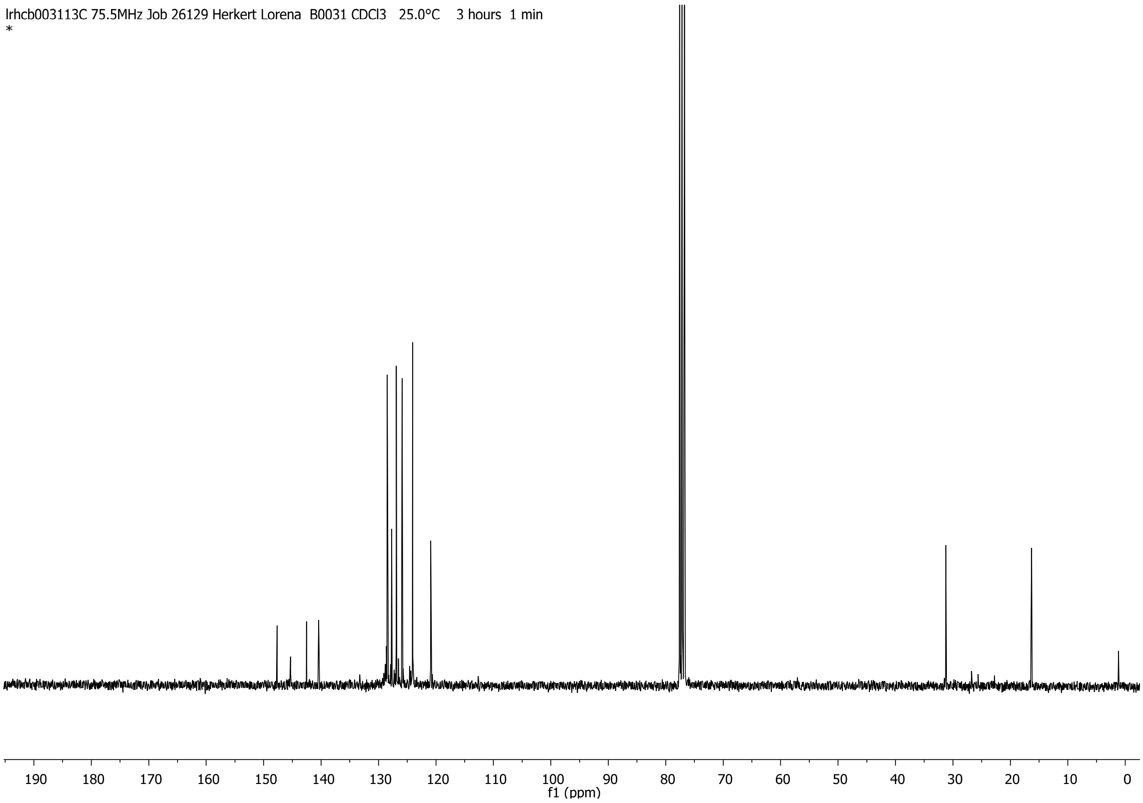

lrhhb0041H 300.1MHz Job 26060 Herkert Lorena B004 CDCl3 24.9°C
*

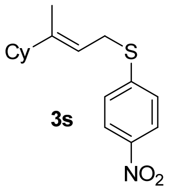

3s

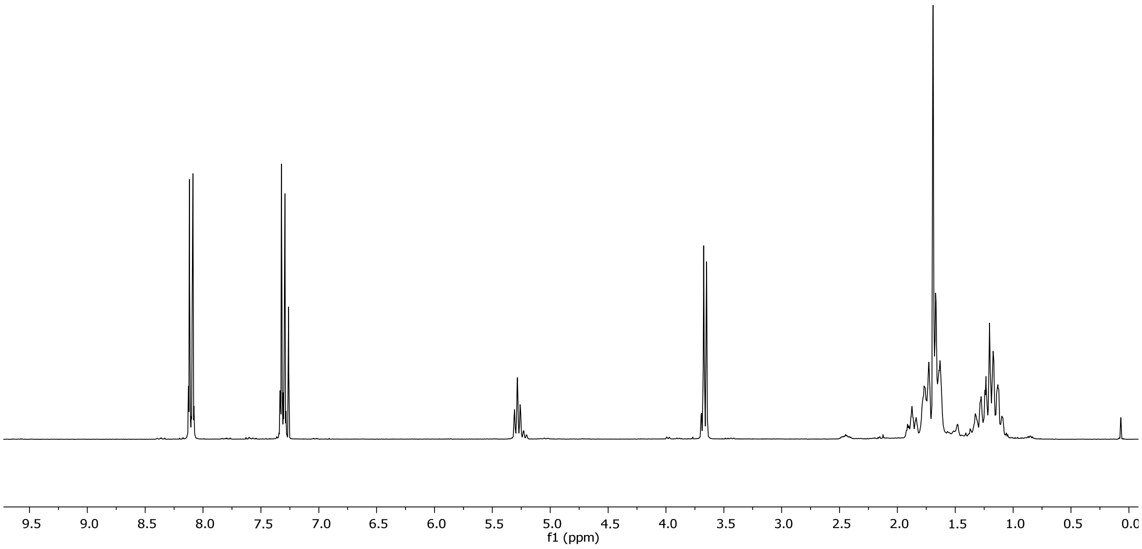

lrhcb00413C 75.5MHz Job 26128 Herkert Lorena B004 CDCl3 25.0°C 1 hour 12 min
*

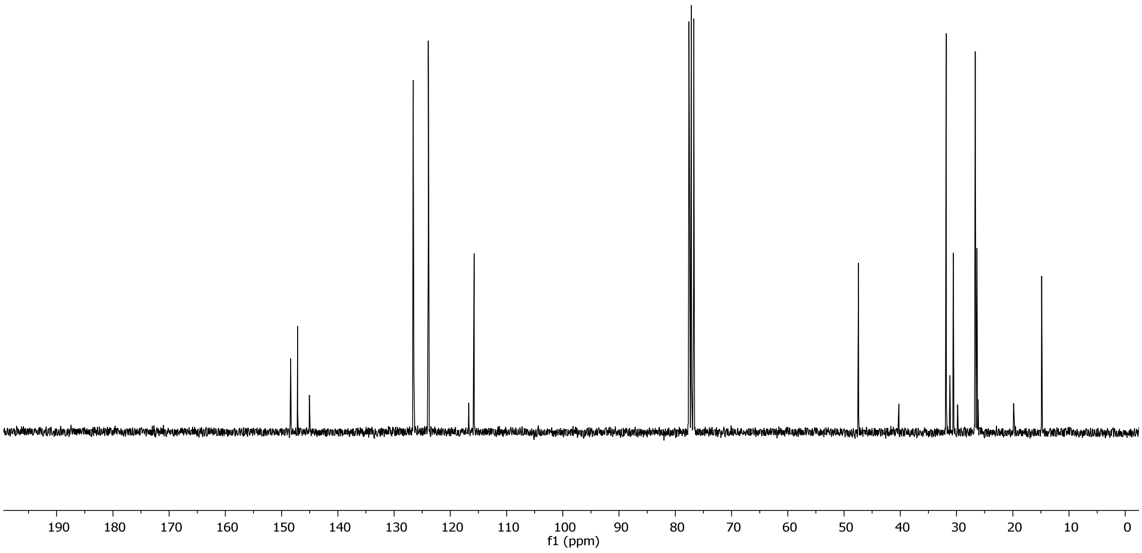

lrhhd0051H 300.1MHz Job 26171 Herkert Lorena D005 CDCl3 25.1°C
*

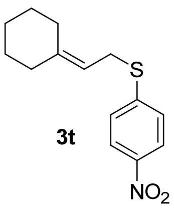

3t

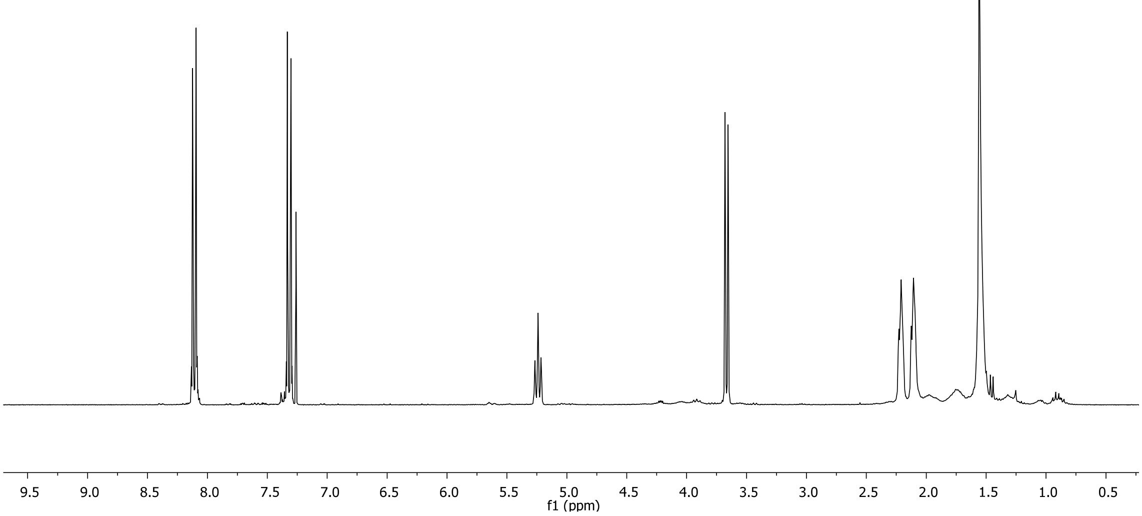

lrhcd00513C 75.5MHz Job 26226 Herkert Lorena D005 CDCl3 25.0°C 3 hours 1 min
*

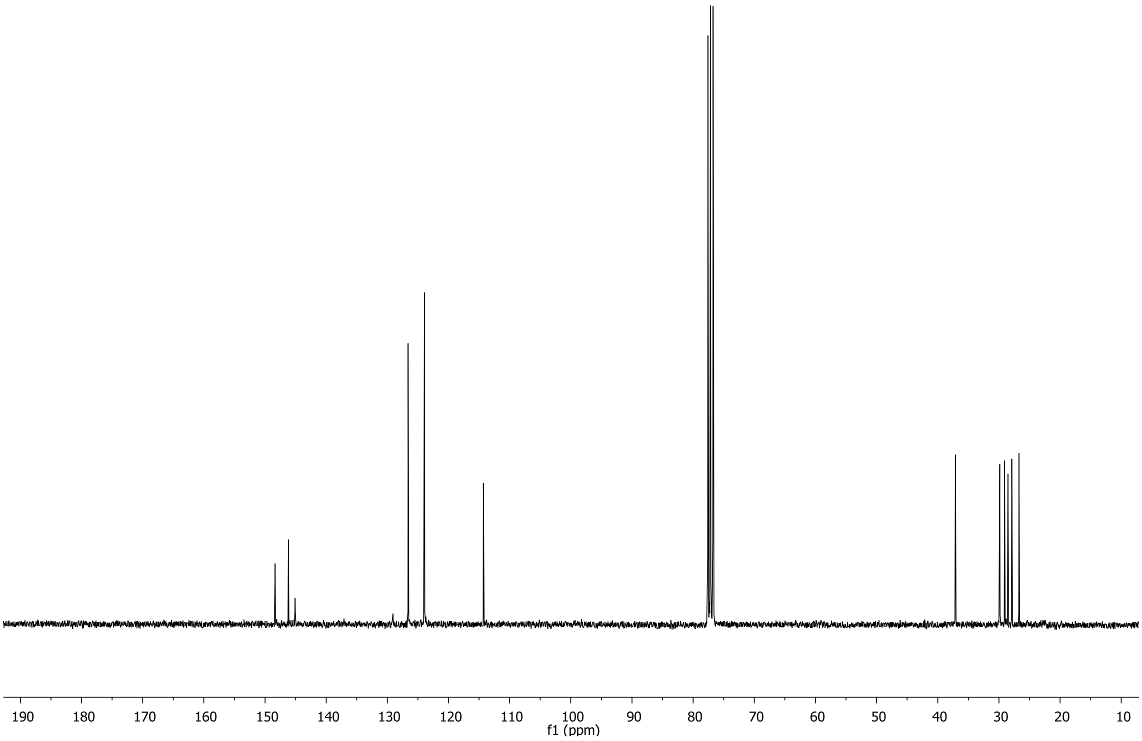

lrhhb0061H 300.1MHz Job 26174 Herkert Lorena B006 CDCl3 25.0°C
*

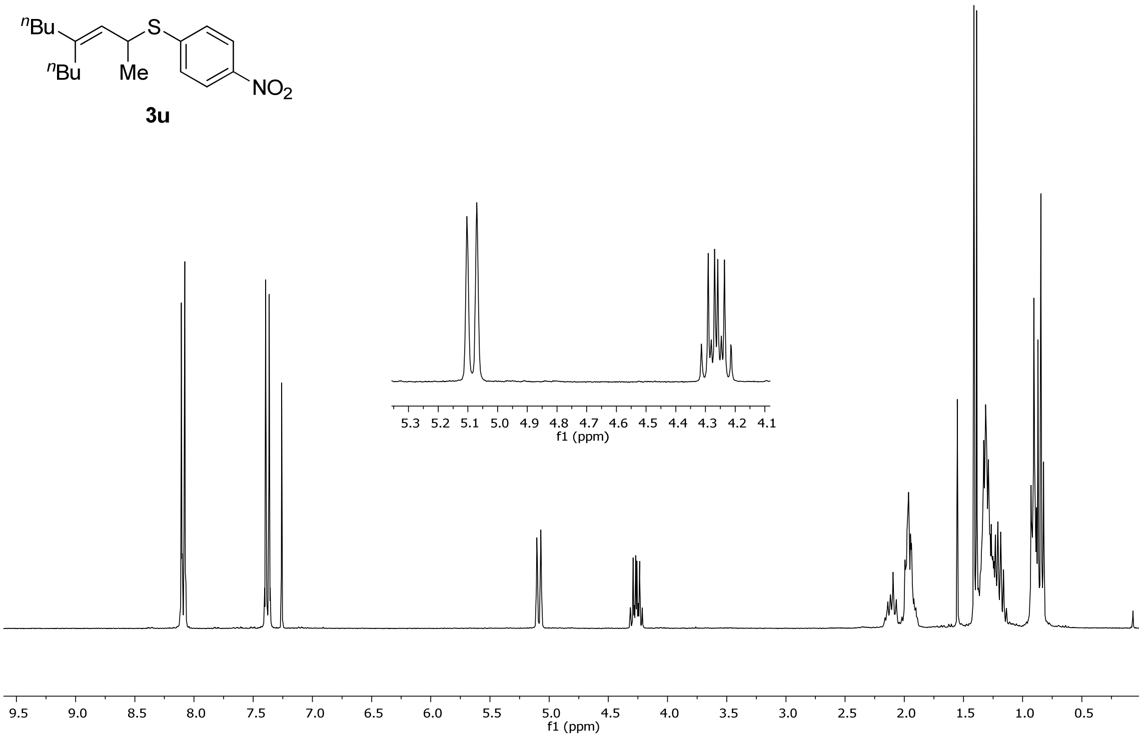

lrhcb00613C 75.5MHz Job 26227 Herkert Lorena B006 CDCl3 25.0°C 3 hours 1 min
*

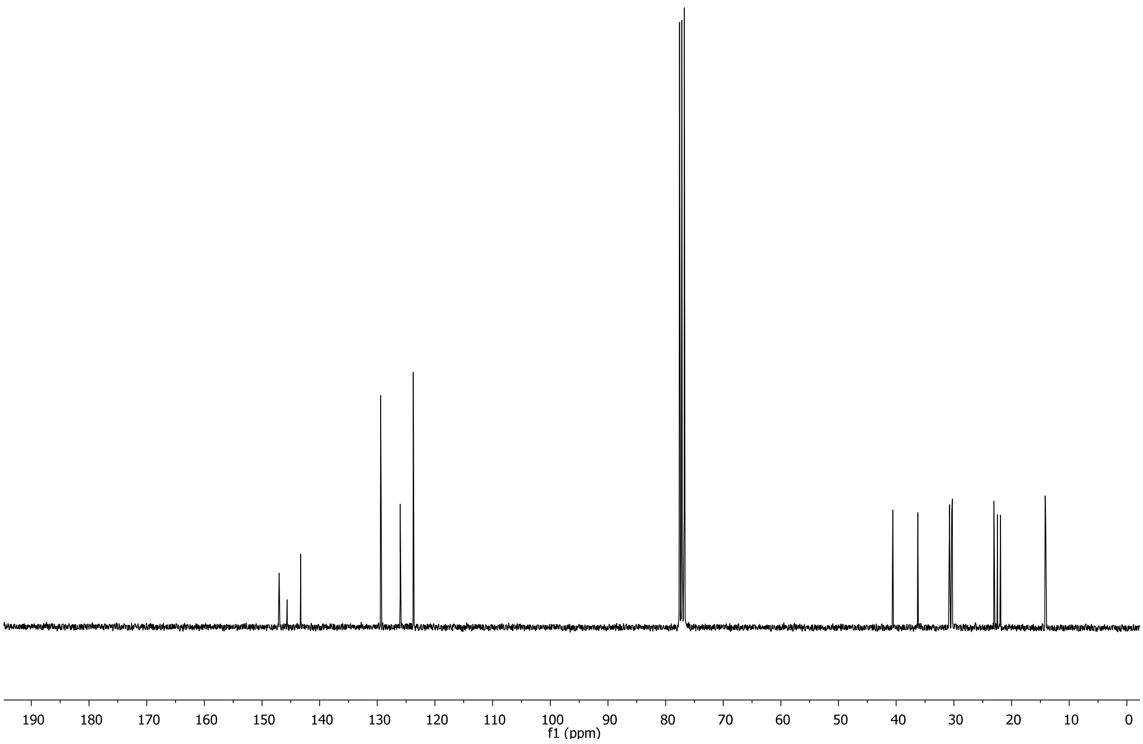

gjbh112321H 300.1MHz Job 29479 Barker Graeme J 11232 CDCl3   25.0°C
GB1/123:2

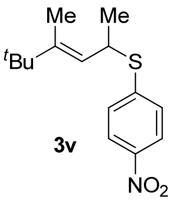

3v

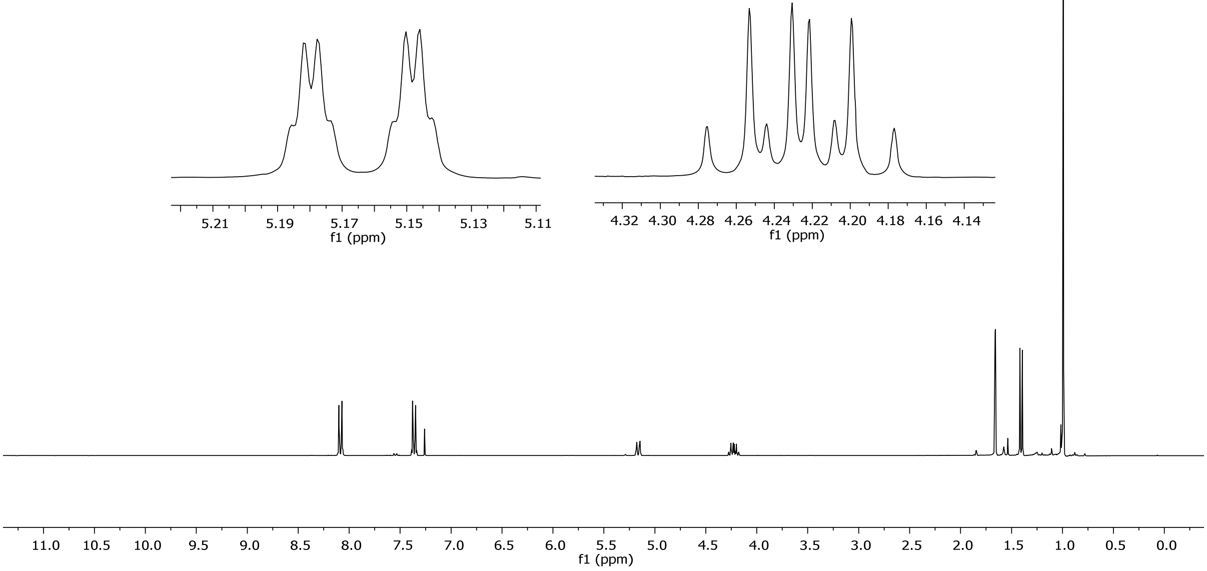

gjbc11232nc13C 75.5MHz Job 29496 Barker Graeme J 11232NC CDCl3   25.0°C   0 hour 54 min
GB1/123:2

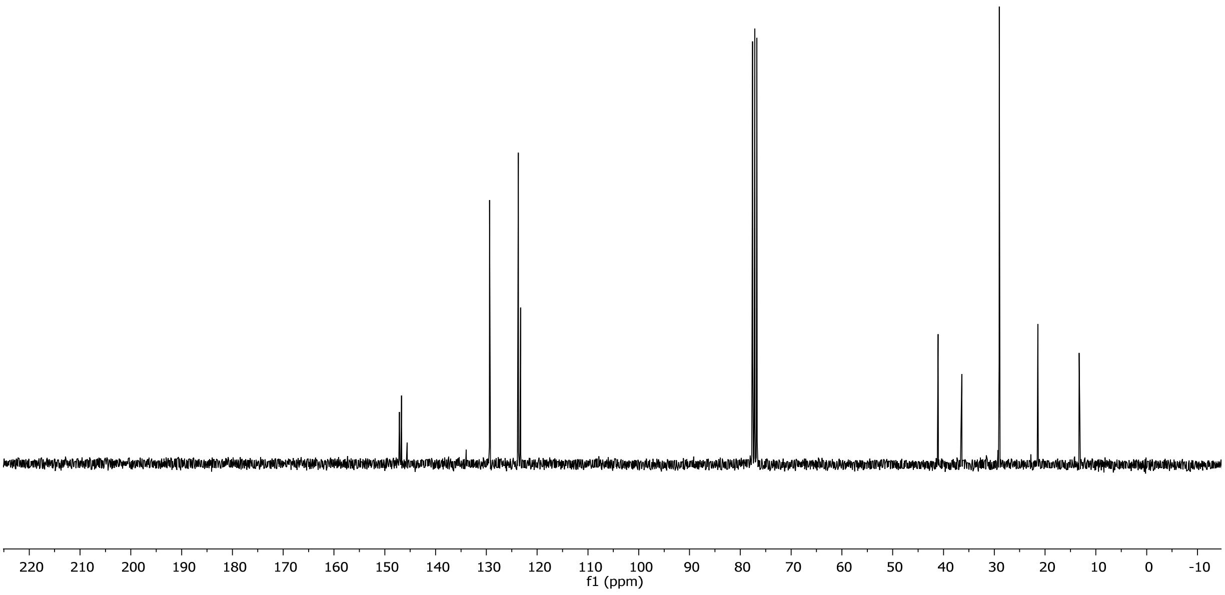

lrhhb0071H 300.1MHz Job 26214 Herkert Lorena B007 CDCl3 24.9°C
*

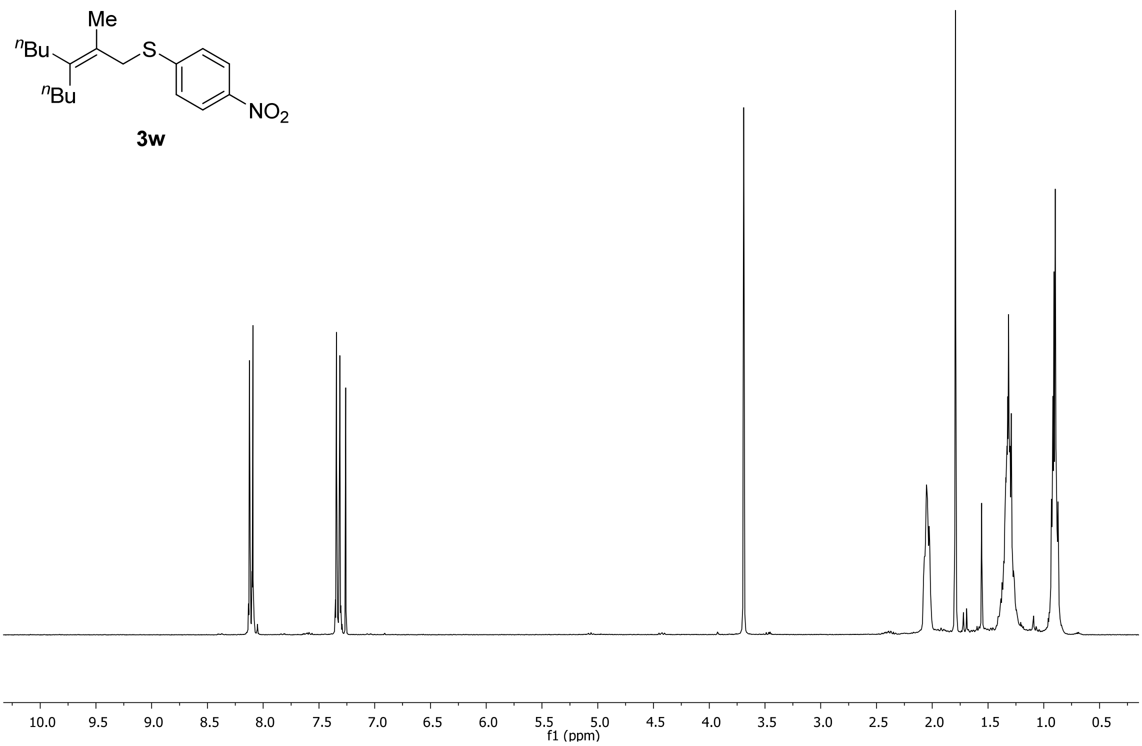

lrhcb00713C 75.5MHz Job 26283 Herkert Lorena B007 CDCl3 25.0°C 3 hours 1 min
*

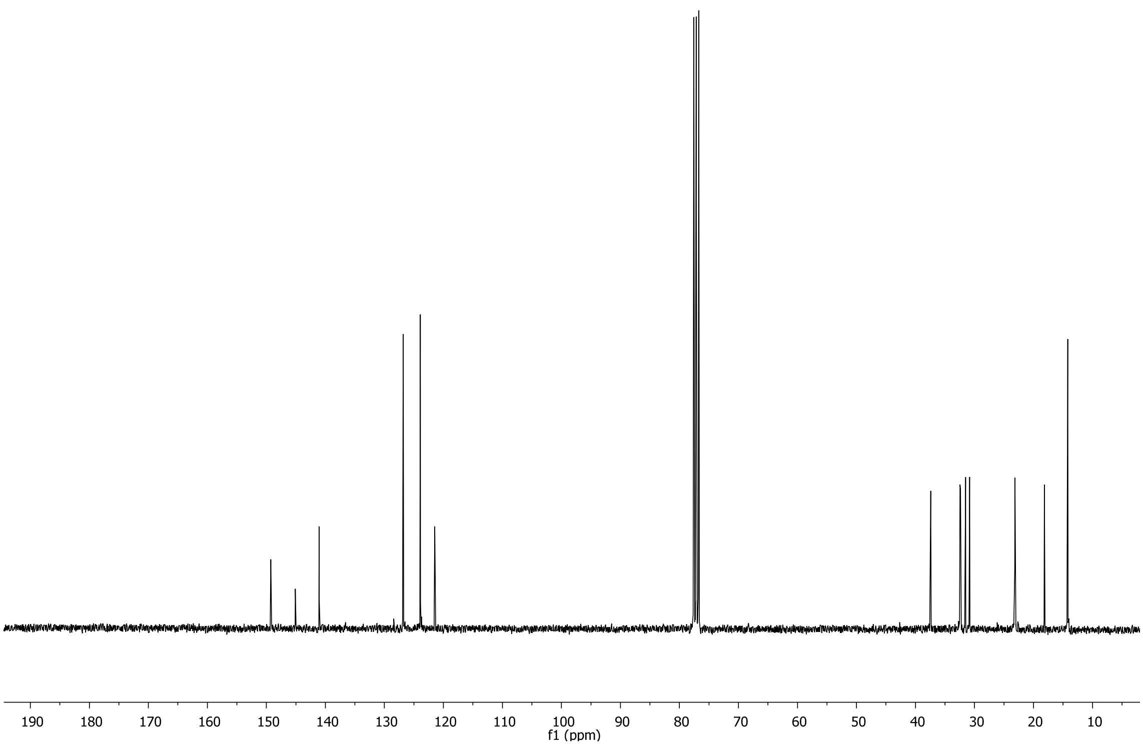

lrhhb0091H 300.1MHz Job 26362 Herkert Lorena B009 CDCl3   25.0°C
*

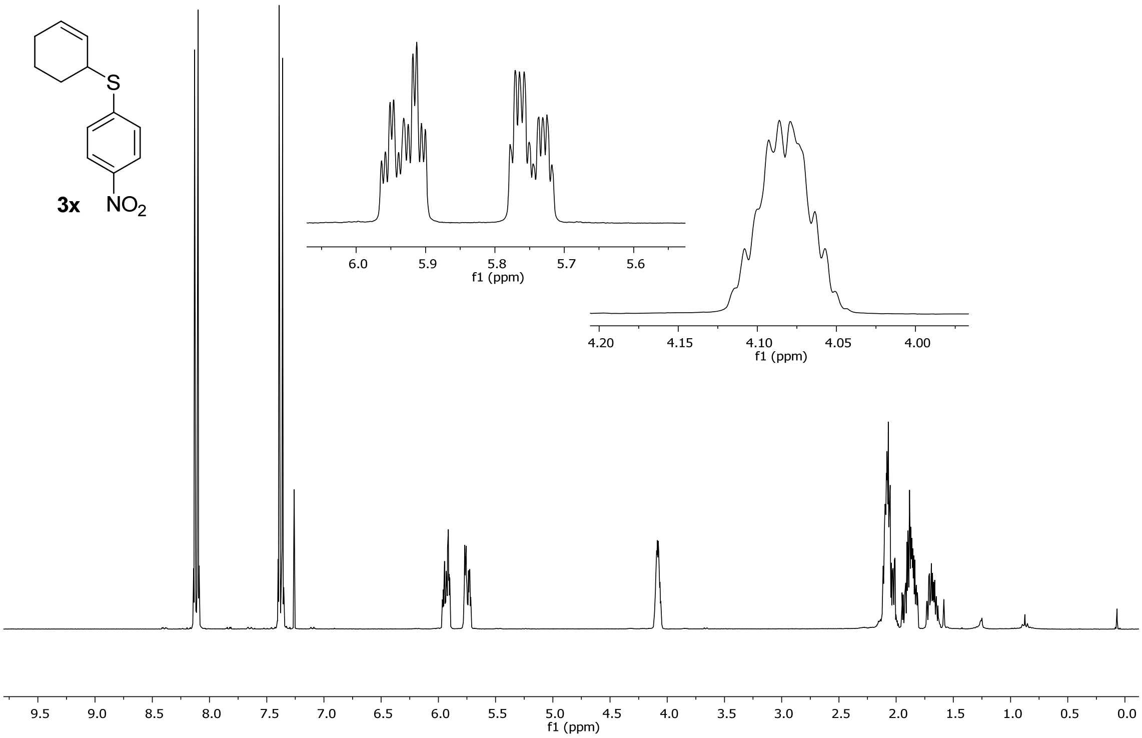

lrhcb00913C 75.5MHz Job 26395 Herkert Lorena B009 CDCl3   25.0°C   0 hour 36 min
*

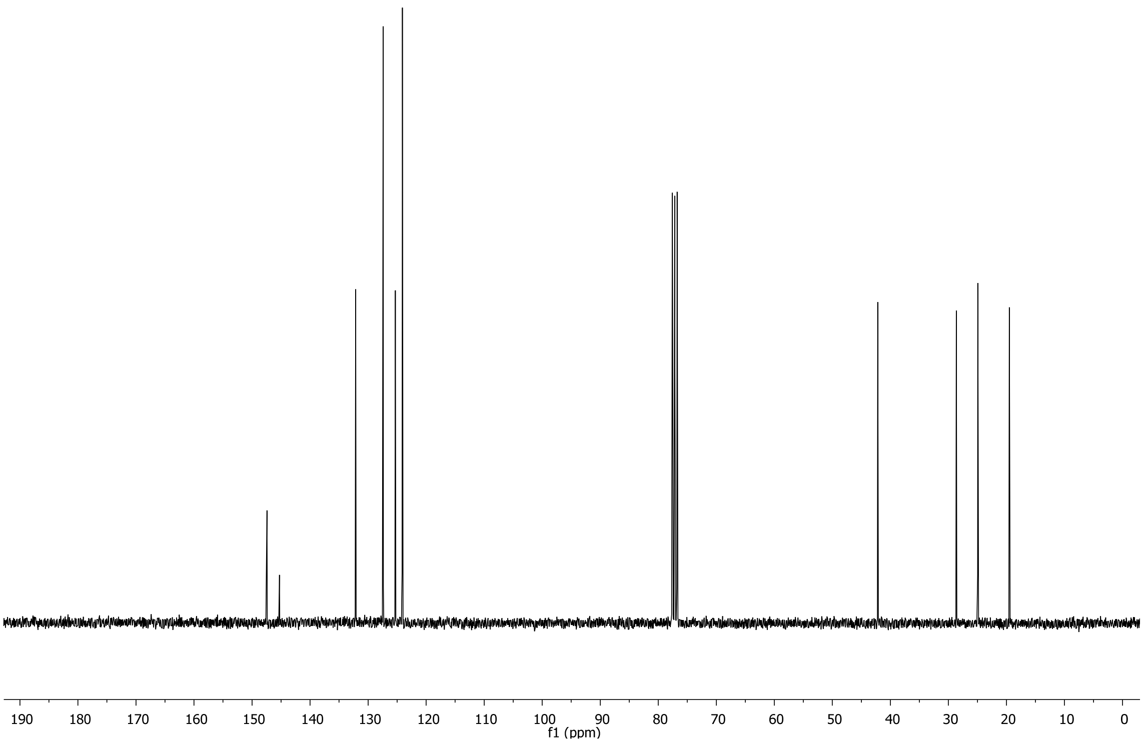

lrhhd0271H 300.1MHz Job 27243 Herkert Lorena D027 CDCl3 24.9°C
*

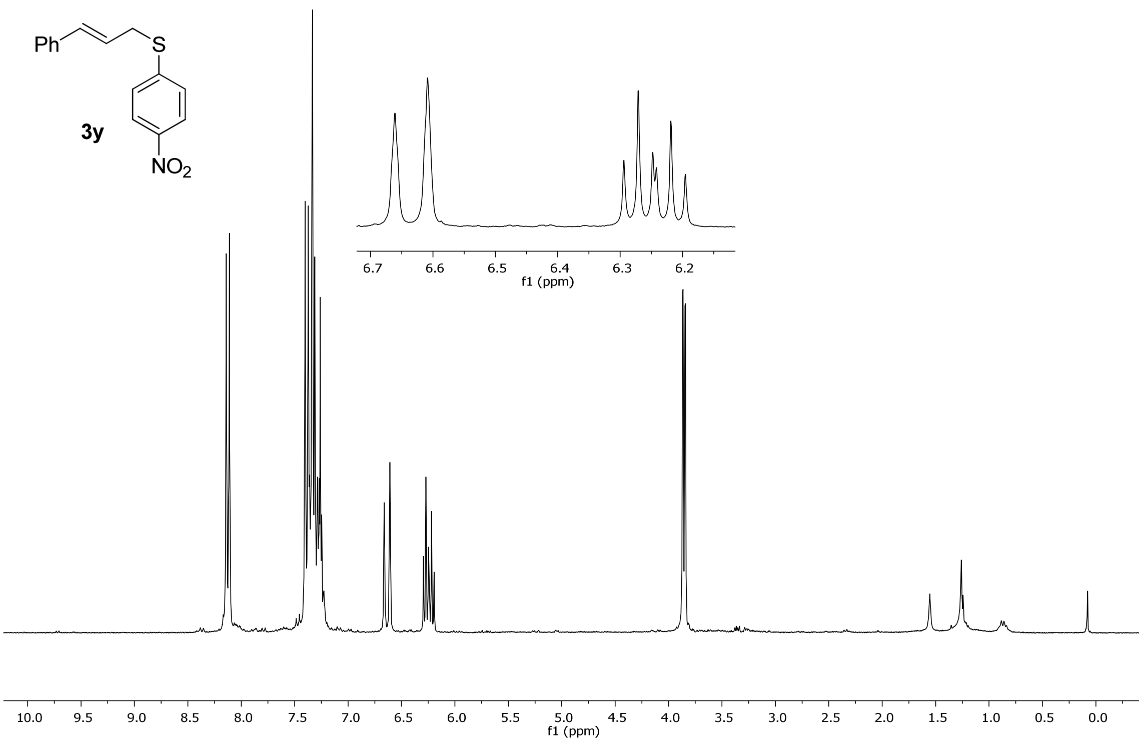

lrhcd02713C 75.5MHz Job 27288 Herkert Lorena D027 CDCl3 24.9°C 3 hours 1 min
*

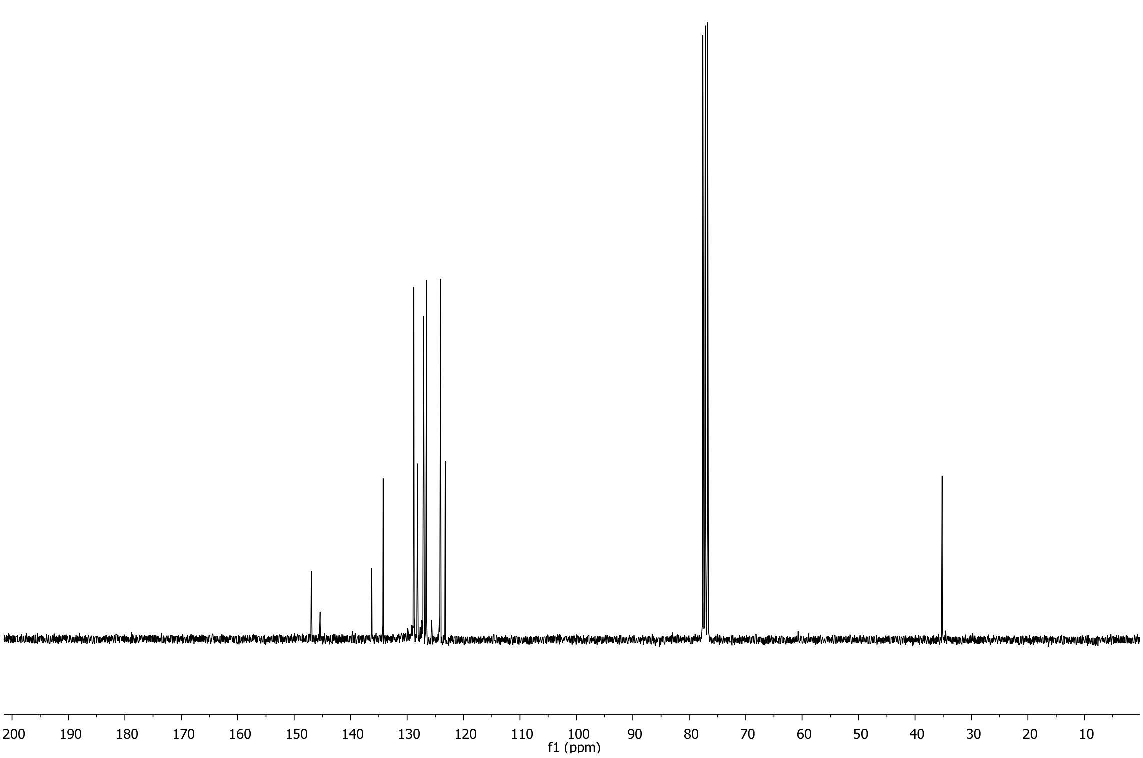

lrhhb0351H 300.1MHz Job 27515 Herkert Lorena B035 CDCl3 25.0°C
*

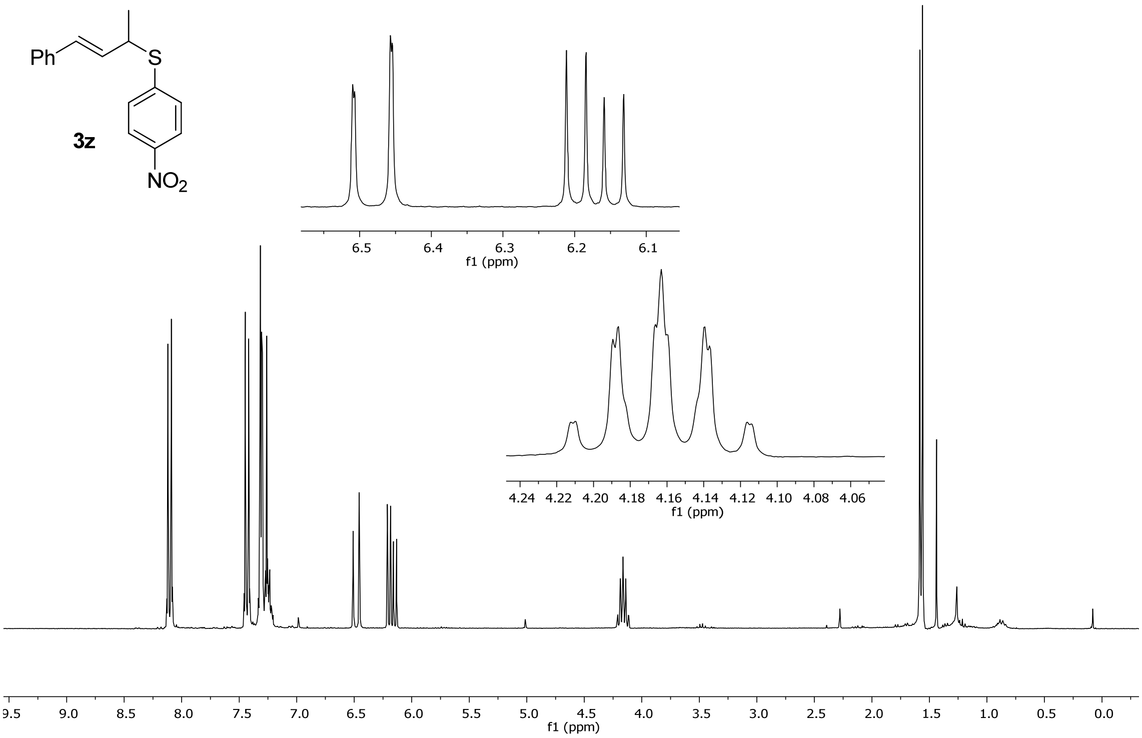

lrhcc03213C 75.5MHz Job 27617 Herkert Lorena C032 CDCl3 25.0°C 3 hours 1 min
*

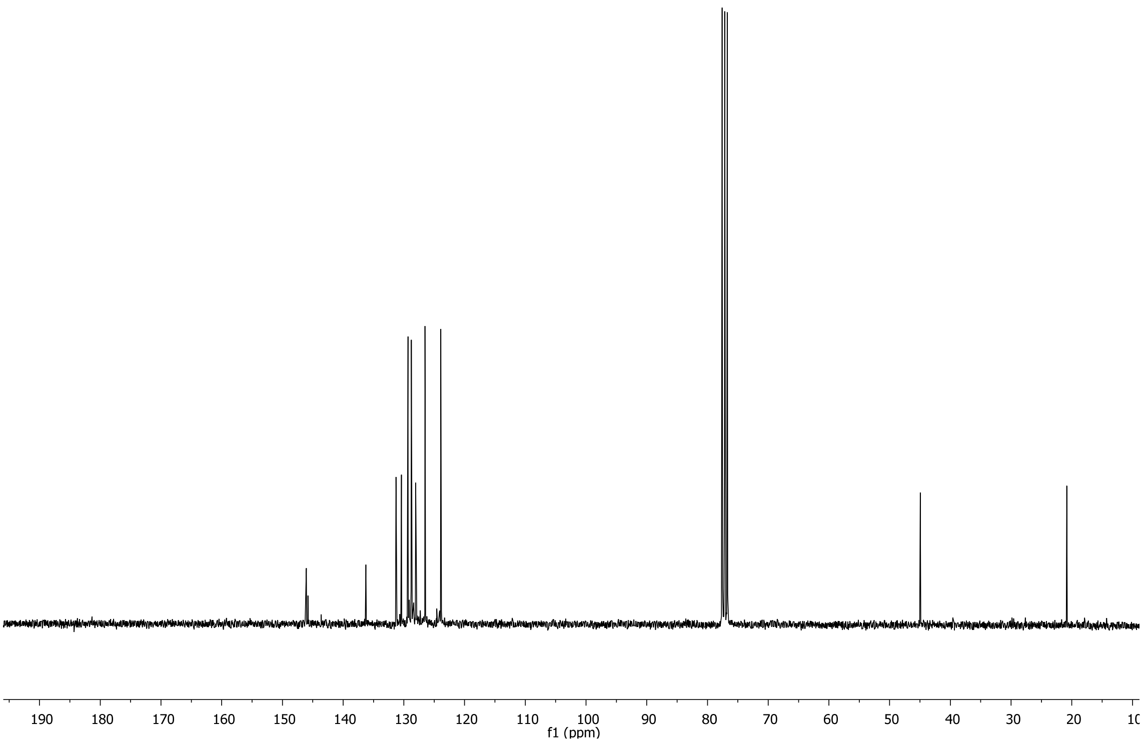

gjbh116921H 400.1MHz Job 20289 Barker Graeme J 11692 CDCl3 22.4°C
*

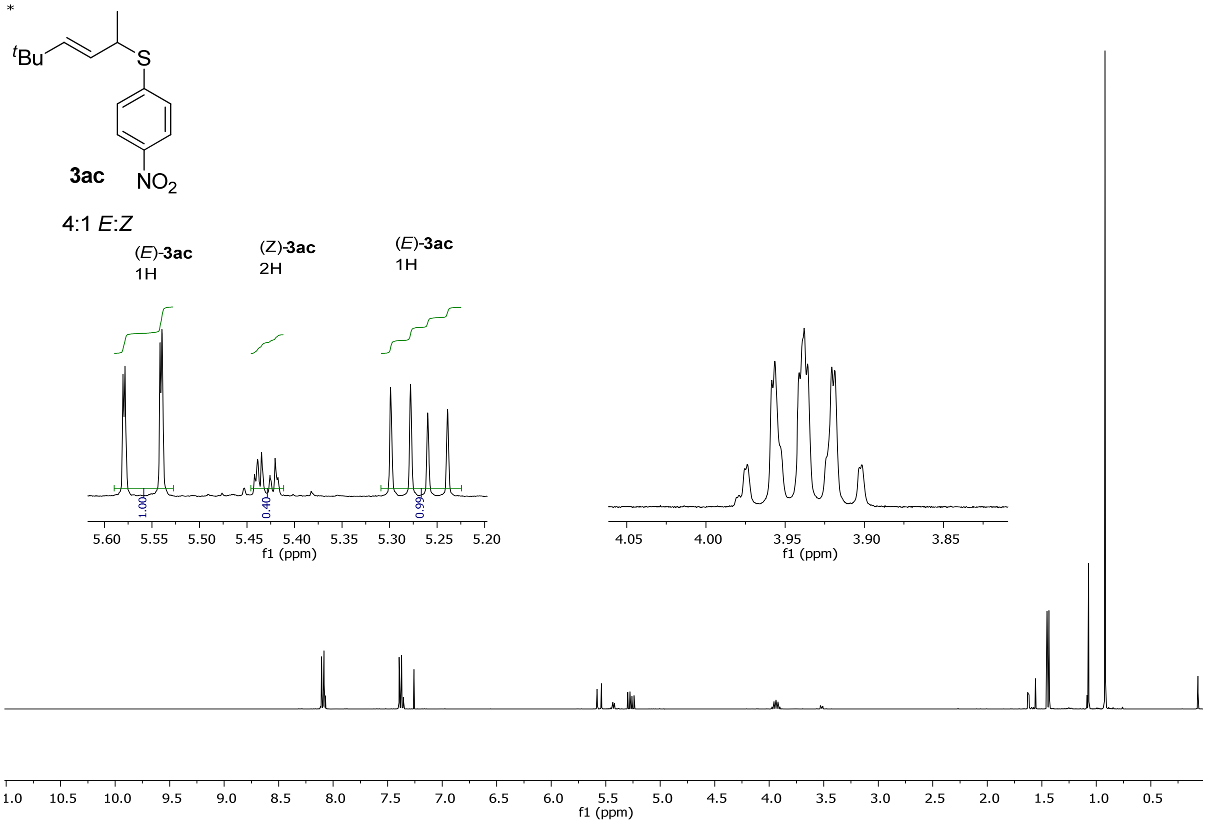

gjbc1169213C 100.6MHz Job 20288 Barker Graeme J 11692 CDCl3 26.0°C 0 hour 43 min
*

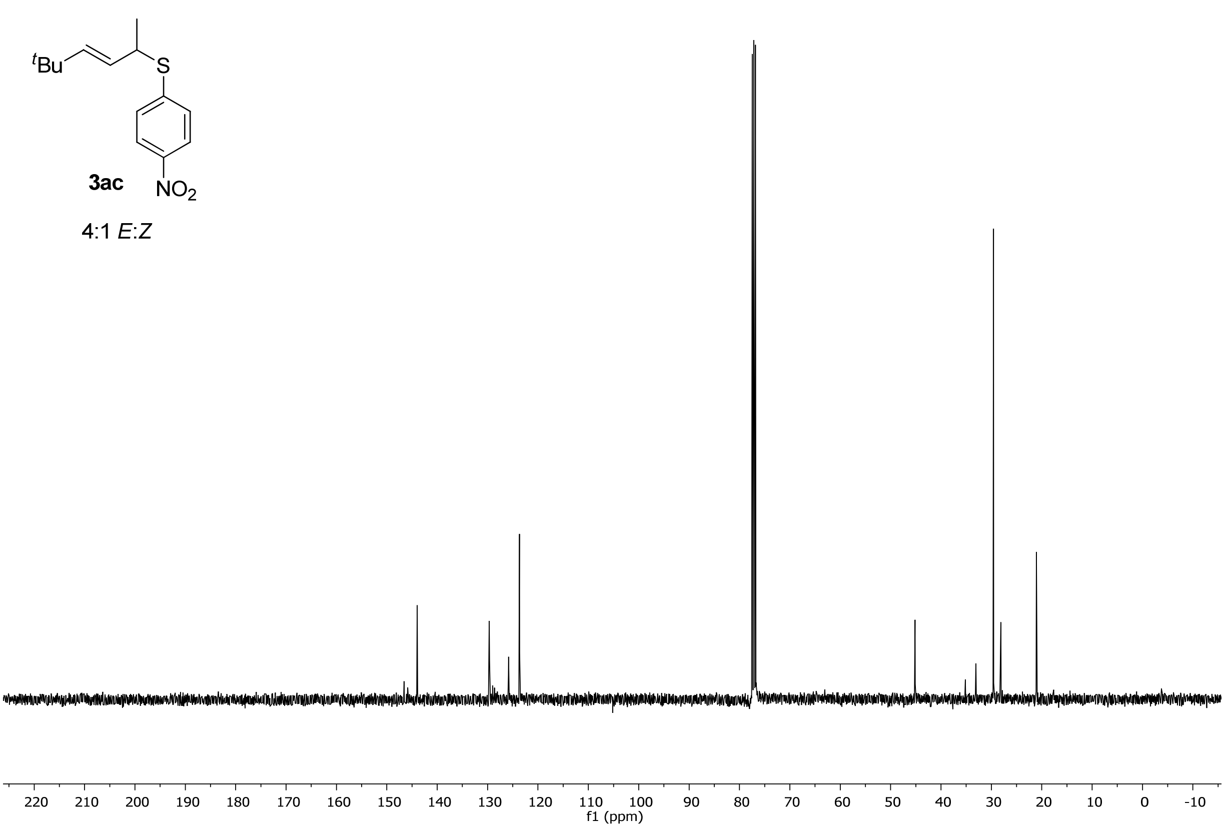

lrhhb0191H 300.1MHz Job 26855 Herkert Lorena B019 CDCl3 25.1°C
*

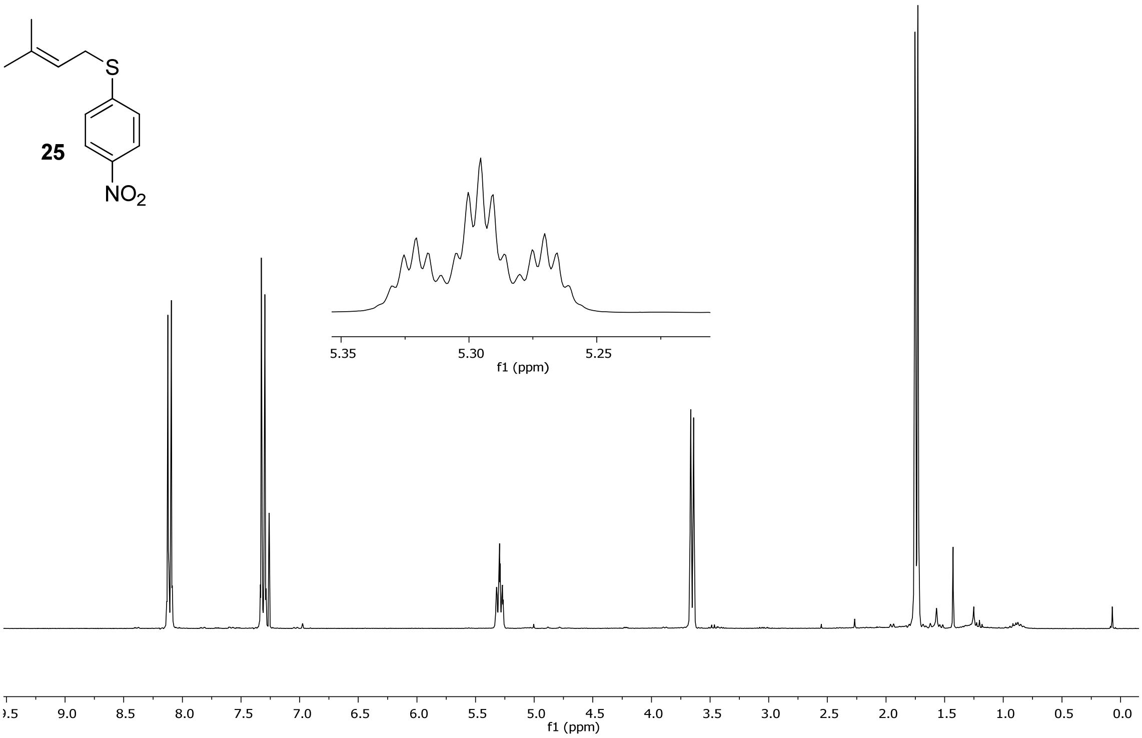

lrhcc01613C 75.5MHz Job 26702 Herkert Lorena C016 CDCl3 25.0°C 3 hours 1 min
*

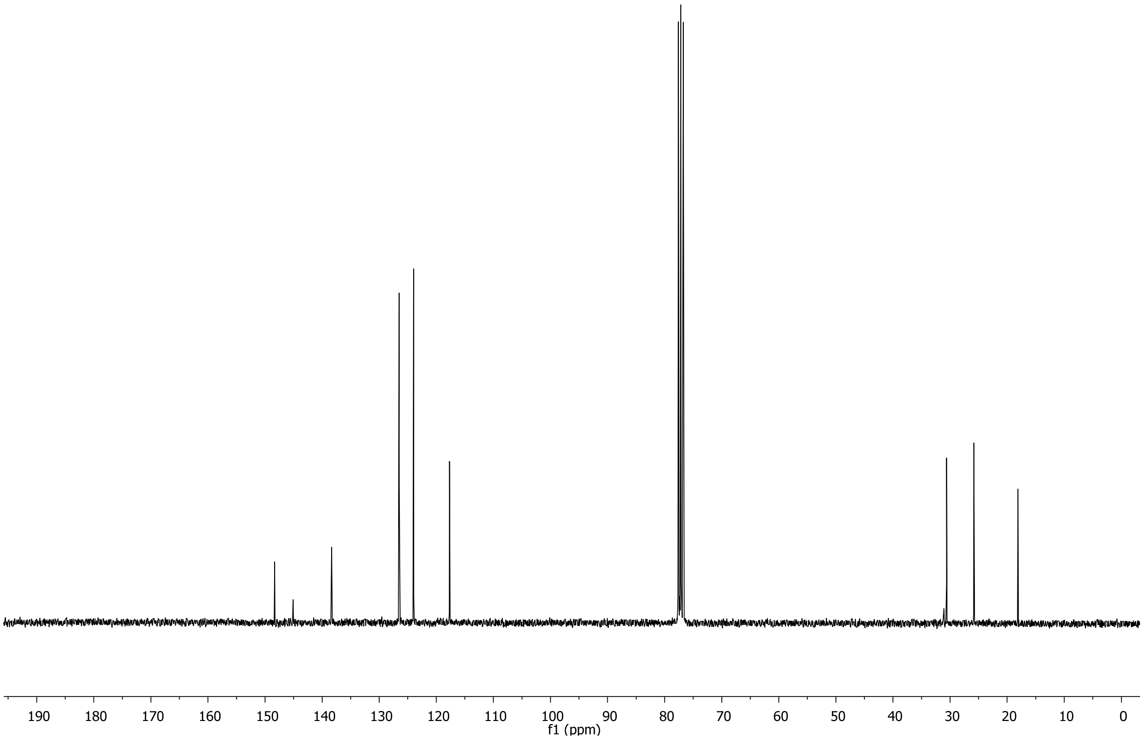

lrhhb0421H 300.1MHz Job 27990 Herkert Lorena B042 CDCl3 25.0°C
*

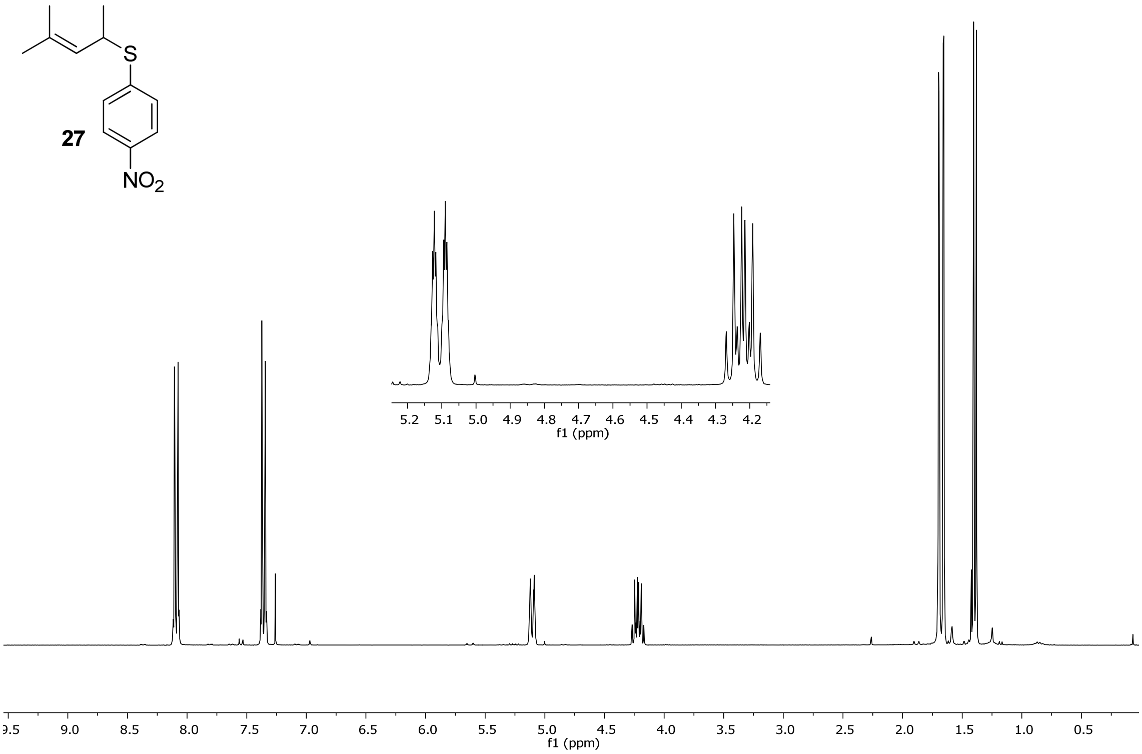

lrhcc04213C 75.5MHz Job 28033 Herkert Lorena C042 CDCl3 25.0°C 0 hour 36 min
*

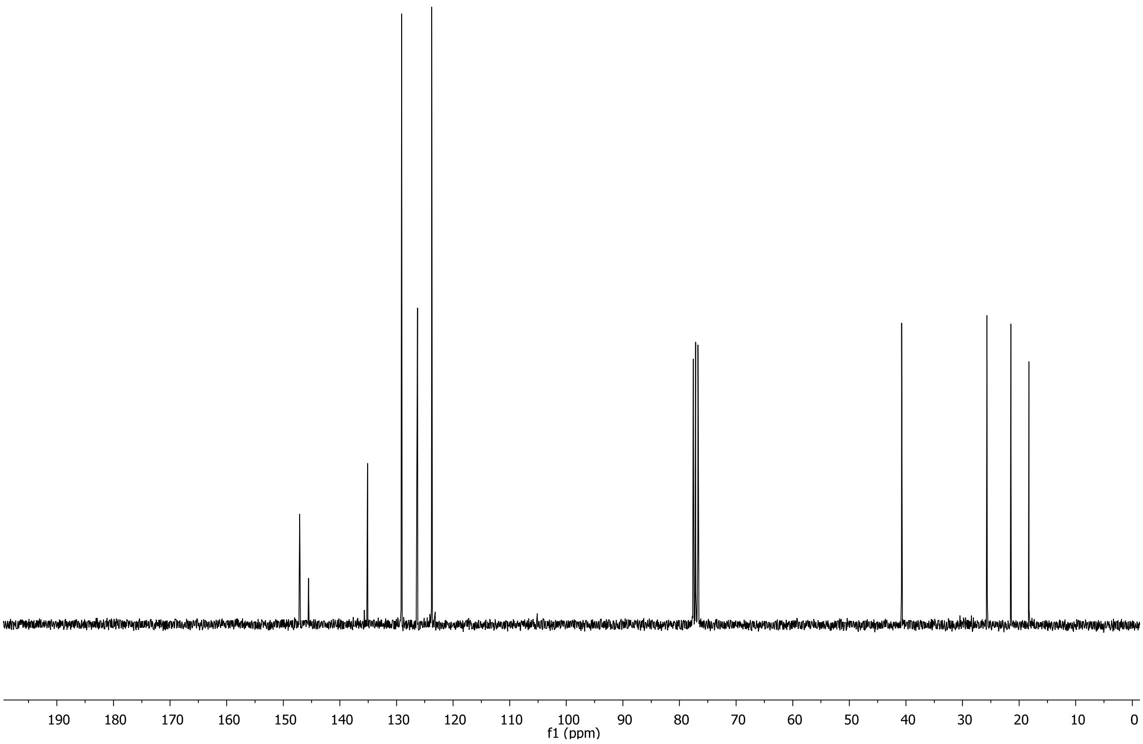

gjbh224521H 300.1MHz Job 35424 Barker Graeme J 22452 CDCl3 25.1°C
GB2/245:2

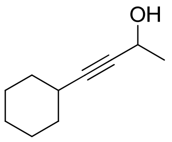

4-cyclohexylbut-3-yn-2-ol

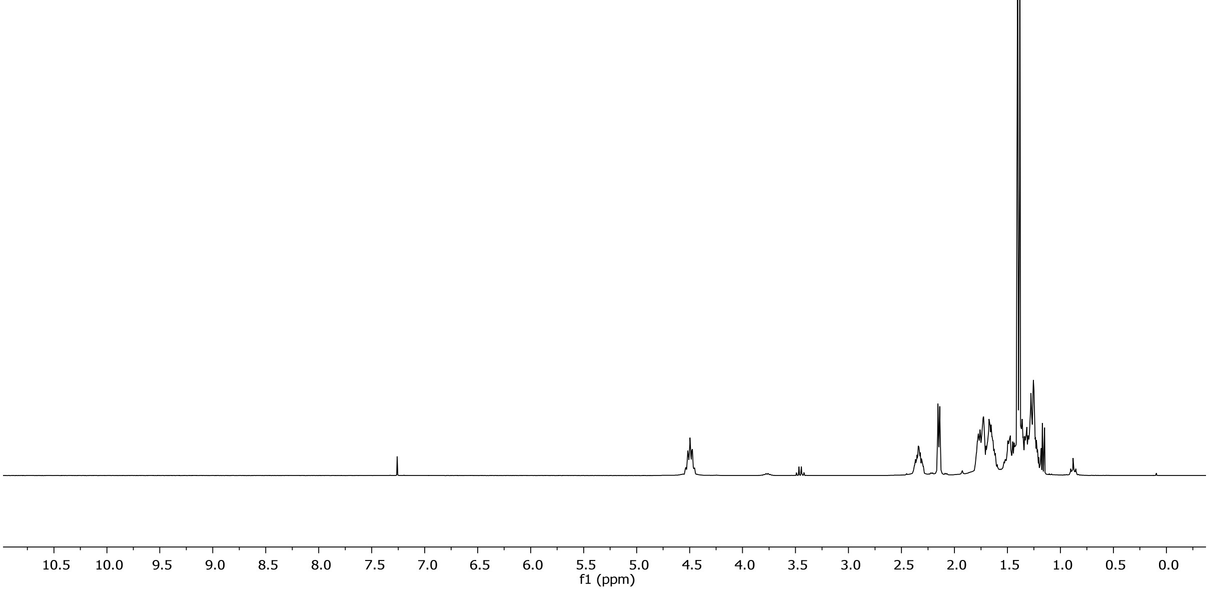

gjbc22452nc13C 75.5MHz Job 35451 Barker Graeme J 22452NC CDCl3 25.0°C 0 hour 18 min
GB2/245:2

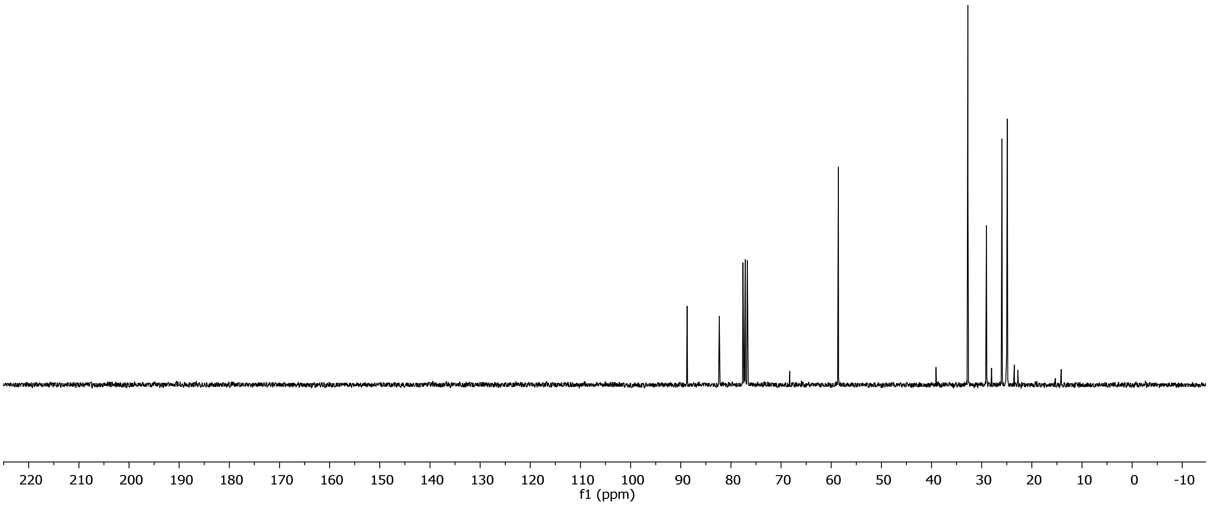

gjbh22472

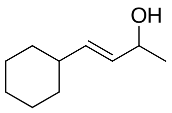

(*E*)-4-cyclohexylbut-3-en-2-ol

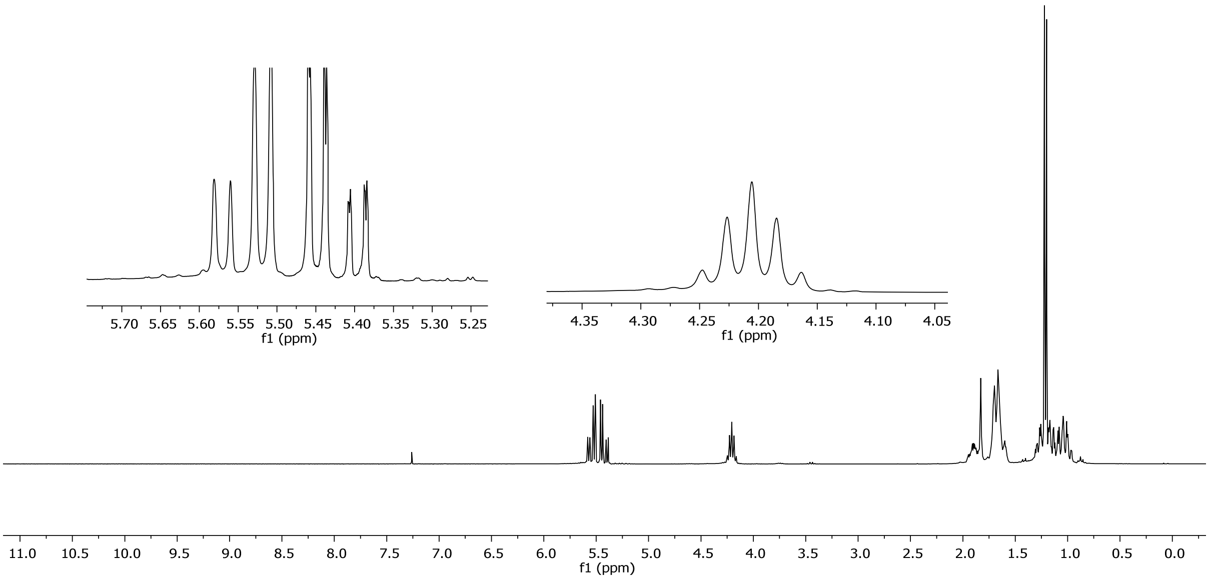

gjbc22472nc13C 75.5MHz Job 35538 Barker Graeme J 22472NC CDCl3 25.0°C 0 hour 18 min
GB2/247:2

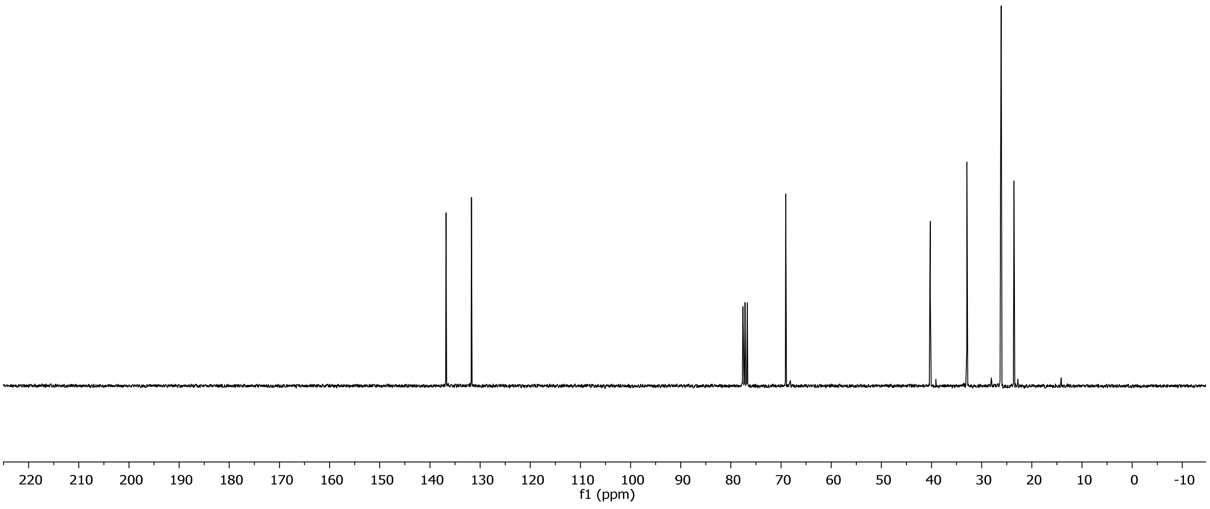

gjbh22482r21H 300.1MHz Job 36691 Barker Graeme J 22482R2 CDCl3 25.0°C
GB2/248:2

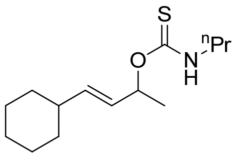

(*E*)-*O*-(4-cyclohexylbut-3-en-2-yl) propylcarbamothioate

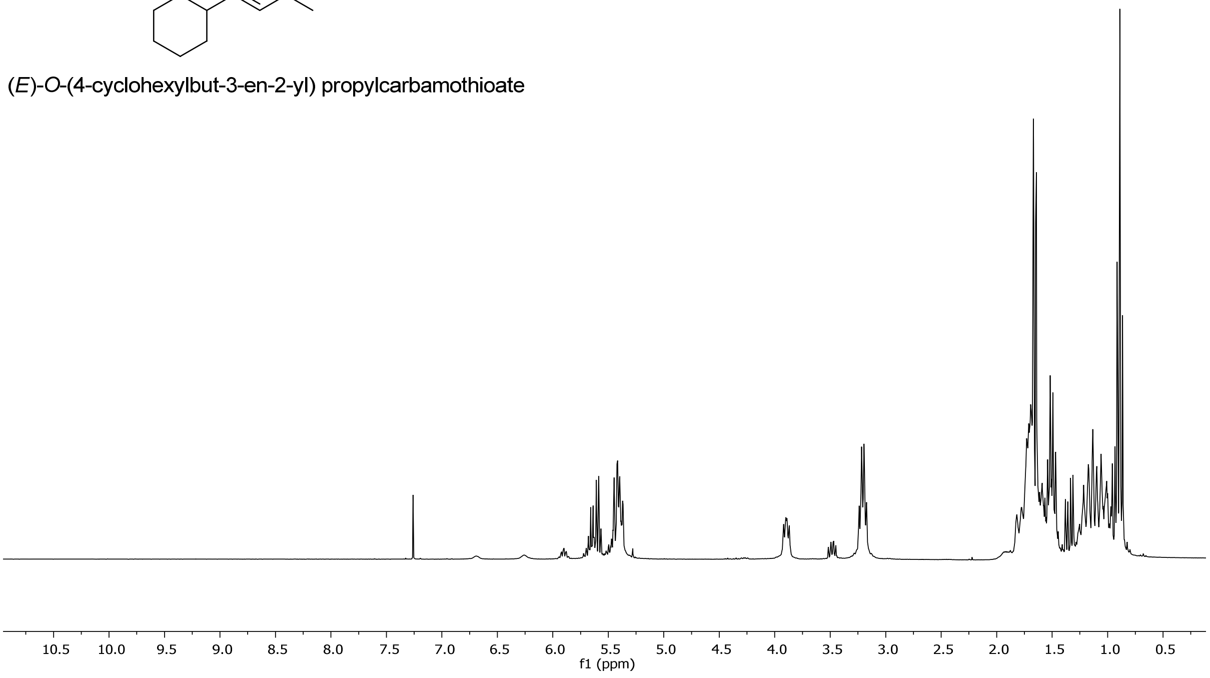

gjbc22482nc13C 75.5MHz Job 36706 Barker Graeme J 22482NC CDCl3 25.0°C 0 hour 18 min
GB2/248:2

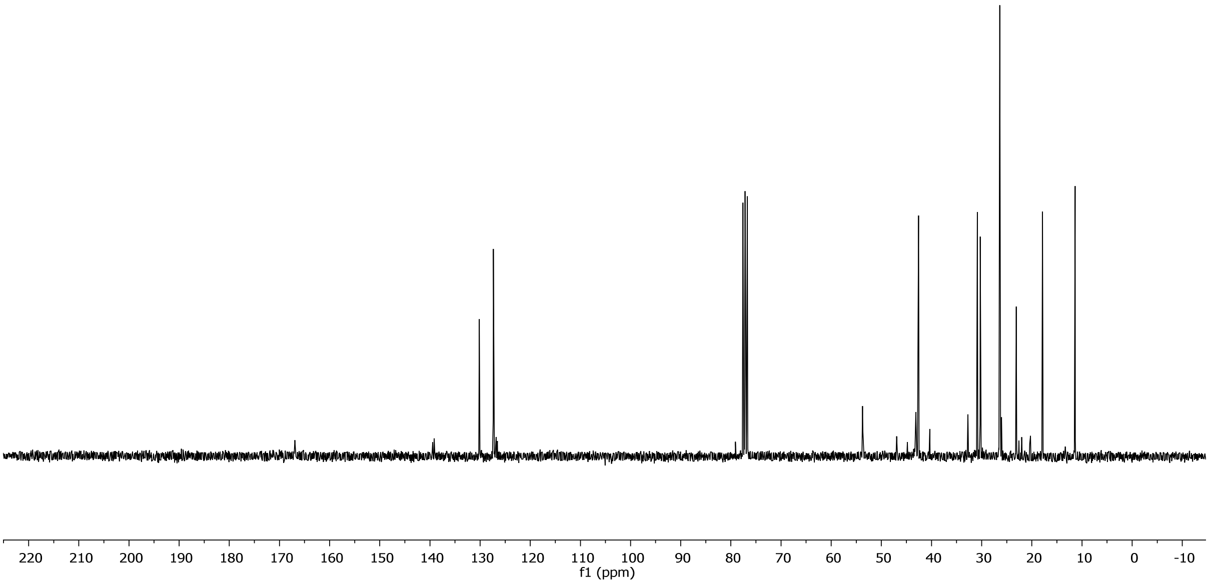

5) References

[1] Y. Masuda, M. Hoshi, A. Arase, *Bull. Chem. Soc. Jpn.* **1992**, *65*, 3294-3299.

[2] F. K. Cheung, A. M. Hayes, D. J. Morris, M. Wills, *Org. Biomol. Chem.* **2007**, *5*, 1093-1103.

[3] C. Morrill, R. H. Grubbs, *J. Am. Chem. Soc.* **2005**, *127*, 2842-2843.

[4] Q. Zhao, D. P. Curran, M. Malacria, L. Fensterbank, J.-P. Goddard, E. Lacôte, *Chem. Eur. J.* **2011**, *17*, 9911-9914.

[5] J. W. Kim, T. Koike, M. Kotani, K. Yamaguchi, N. Mizuno, *Chem. Eur. J.* **2008**, *14*, 4104-4109.

[6] P. C. Young, N. A. Schopf, A.-L. Lee, *Chem. Commun.* **2013**, *49*, 4262-4264.

[7] N. Harrington-Frost, H. Leuser, M. I. Calaza, F. F. Kneisel, P. Knochel, *Org. Lett.* **2003**, *5*, 2111-2114.

[8] J. L. Belelie, J. M. Chong, *J.Org. Chem.* **2001**, *66*, 5552-5555.

[9] H. Yamamoto, E. Ho, I. Sasaki, M. Mitsutake, Y. Takagi, H. Imagawa, M. Nishizawa, *Eur. J. Org. Chem.* **2011**, *2011*, 2417-2420.

[10] A. Fernandez-Mateos, S. Encinas Madrazo, P. Herrero Teijon, R. Rubio Gonzalez, *Eur. J. Org. Chem.* **2010**, 856-861.

[11] S. F. Mayer, A. Steinreiber, R. V. A. Orru, K. Faber, *J.Org. Chem.* **2002**, *67*, 9115-9121.

[12] A. Kulshrestha, J. M. Schomaker, D. Holmes, R. J. Staples, J. E. Jackson, B. Borhan, *Chemistry* **2011**, *17*, 12326-12339.

[13] M. E. Cain, *J. Chem. Soc.* **1964**, 3532-3535.

[14] H.-J. Gais, A. Böhme, *J.Org. Chem.* **2002**, *67*, 1153-1161.

[15] K. M. Buchner, T. B. Clark, J. M. N. Loy, T. X. Nguyen, K. A. Woerpel, *Org. Lett.* **2009**, *11*, 2173-2175.

[16] J. Ye, J. Zhao, J. Xu, Y. Mao, Y. J. Zhang, *Chem. Commun.* **2013**, *49*, 9761-9763.

Computational Supporting Information for:

Gold(I)-Catalysed Direct Thioetherifications using Allylic Alcohols – an Experimental and Computational Study

Lorena Herkert, Samantha L. J. Green, Graeme Barker, David G. Johnson, Paul C. Young, Stuart A. Macgregor* and Ai-Lan Lee*[a]

Institute of Chemical Sciences,
Heriot-Watt University,
Edinburgh EH14 4AS United Kingdom.
Fax: (+)44 131 4513180
E-mail: S.A.Macgregor@hw.ac.uk; A.Lee@hw.ac.uk

Table of Contents

1. Computational Details and References pg 73
2. Reactions of Substrate **4'** pg 75
3. Reactions of Substrate **24** pg 103
4. Reactions of Substrate **4'** with PhSH at [(Johnphos)Au(NCMe)]$^{+}$, **5**. pg 127
5. Reactions of **9**, **10** and **11** with PhSH pg 149
6. Reactions of Substrate **17** pg 162

1. Computational Details and References.

Calculations were run with Gaussian 03 Revision D.01[1] with PCM solvent corrections run with Gaussian 09, Revision A.02.[2] Geometry optimisations were performed using the BP86 functional[3] with Au, P and S centres described with the Stuttgart RECPs and associated basis sets[4] (with added d-orbital polarisation on P (ζ = 0.387) and S (ζ = 0.503)[5] and 6-31G** basis sets for all other atoms.[6] All stationary points were fully characterized via analytical frequency calculations as either minima (all positive eigenvalues) or transition states (one negative eigenvalue) and IRC calculations and subsequent geometry optimizations were used to confirm the minima linked by each transition state. Frequency calculations also provided a free energy in the gas-phase, computed at 298.15 K and 1 atm. Energies reported in the text are based on the gas-phase free energies and incorporate a correction for dispersion effects using Grimme's D3 parameter set[7] (i.e. BP86-D3) as well as solvation (PCM approach) in $CHCl_3$.

1. Gaussian 03, Revision C.02, M. J. Frisch, G. W. Trucks, H. B. Schlegel, G. E. Scuseria, M. A. Robb, J. R. Cheeseman, J. A. Montgomery, Jr., T. Vreven, K. N. Kudin, J. C. Burant, J. M. Millam, S. S. Iyengar, J. Tomasi, V. Barone, B. Mennucci, M. Cossi, G. Scalmani, N. Rega, G. A. Petersson, H. Nakatsuji, M. Hada, M. Ehara, K. Toyota, R. Fukuda, J. Hasegawa, M. Ishida, T. Nakajima, Y. Honda, O. Kitao, H. Nakai, M. Klene, X. Li, J. E. Knox, H. P. Hratchian, J. B. Cross, C. Adamo, J. Jaramillo, R. Gomperts, R. E. Stratmann, O. Yazyev, A. J. Austin, R. Cammi, C. Pomelli, J. W. Ochterski, P. Y. Ayala, K. Morokuma, G. A. Voth, P. Salvador, J. J. Dannenberg, V. G. Zakrzewski, S. Dapprich, A. D. Daniels, M. C. Strain, O. Farkas, D. K. Malick, A. D. Rabuck, K. Raghavachari, J. B. Foresman, J. V. Ortiz, Q. Cui, A. G. Baboul, S. Clifford, J. Cioslowski, B. B. Stefanov, G. Liu, A. Liashenko, P. Piskorz, I. Komaromi, R. L. Martin, D. J. Fox, T. Keith, M. A. Al-Laham, C. Y. Peng, A. Nanayakkara, M. Challacombe, P. M. W. Gill, B. Johnson, W. Chen, M. W. Wong, C. Gonzalez, and J. A. Pople, Gaussian, Inc., Wallingford CT, 2004.
2. Gaussian 09, Revision A.02, M. J. Frisch, G. W. Trucks, H. B. Schlegel, G. E. Scuseria, M. A. Robb, J. R. Cheeseman, G. Scalmani, V. Barone, B. Mennucci, G. A. Petersson, H. Nakatsuji, M. Caricato, X. Li, H. P. Hratchian, A. F. Izmaylov, J. Bloino, G. Zheng, J. L. Sonnenberg, M. Hada, M. Ehara, K. Toyota, R. Fukuda, J. Hasegawa, M. Ishida, T. Nakajima, Y. Honda, O. Kitao, H. Nakai, T. Vreven, J. A. Montgomery, Jr., J. E. Peralta, F. Ogliaro, M. Bearpark, J. J. Heyd, E. Brothers, K. N. Kudin, V. N. Staroverov, R. Kobayashi, J. Normand, K. Raghavachari, A. Rendell, J. C. Burant, S. S. Iyengar, J. Tomasi, M. Cossi, N. Rega, J. M. Millam, M. Klene, J. E. Knox, J. B. Cross, V. Bakken, C. Adamo, J. Jaramillo, R. Gomperts, R. E. Stratmann, O. Yazyev, A. J. Austin, R. Cammi, C. Pomelli, J. W. Ochterski, R. L. Martin, K. Morokuma, V. G. Zakrzewski, G. A. Voth, P. Salvador, J. J. Dannenberg, S. Dapprich, A. D. Daniels, O. Farkas, J. B. Foresman, J. V. Ortiz, J. Cioslowski, and D. J. Fox, Gaussian, Inc., Wallingford CT, 2009.
3. (a) A. D. Becke, *Phys. Rev. A* **1988**, *38*, 3098. (b) J. P. Perdew, *Physical Review B* **1986**, *33*, 8822.
4. D. Andrae, U. Häußermann, M. Dolg, H. Stoll and H. Preuß, *Theor. Chim. Acta* **1990**, *77*, 123.
5. A. Hollwarth, M. Bohme, S. Dapprich, A. W. Ehlers, A. Gobbi, V. Jonas, K. F. Kohler, R. Stegmann, A. Veldkamp and G. Frenking, *Chem. Phys. Lett.* **1993**, *208*, 237.
6. (a) W. J. Hehre, R. Ditchfield and J. A. Pople, *J. Chem. Phys.* **1972**, *56*, 2257. (b) P. C. Hariharan and J. A. Pople, *Theor. Chim. Acta.* **1973**, *28*, 213.
7. S. Grimme, J. Antony, S. Ehrlich and H. Krieg, *J. Chem. Phys.* **2010**, *132*, 154104.

2. Reactions of Model Substrate 4'

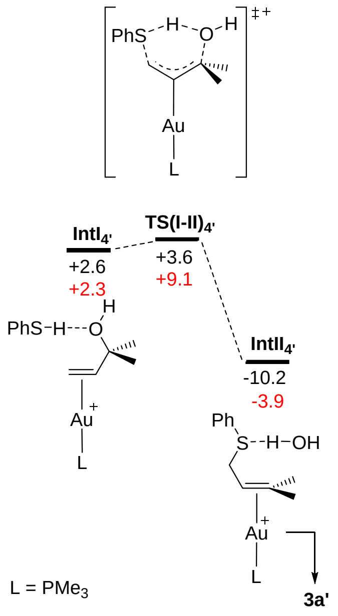

Figure S1. First S_N2' step via *anti* attack of PhSH. Gas phase SCF energies (BP86) are shown in black with free energies at the BP86-D3($CHCl_3$) in red. All energies are in kcal/mol and are quote relative to **5'** and the separated reactants set to zero.

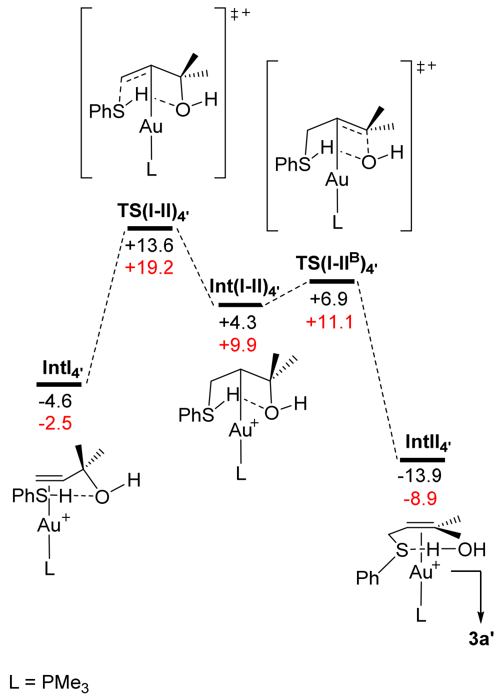

Figure S2. First S_N2' step via *syn* attack of PhSH. Gas phase SCF energies (BP86) are shown in black with free energies at the BP86-D3($CHCl_3$) in red. All energies are in kcal/mol and are quoted relative to **5'** and the separated reactants set to zero.

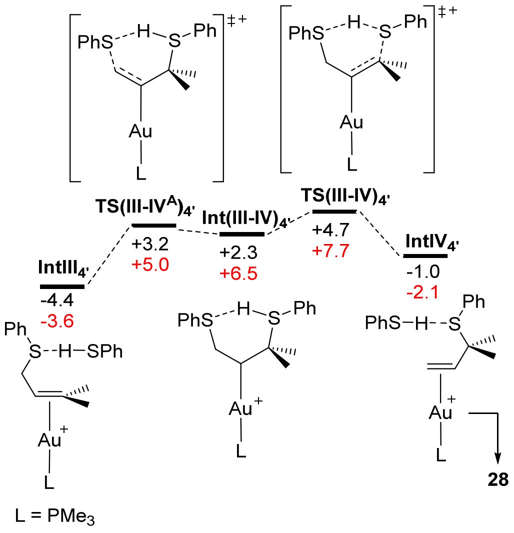

Figure S3. Second S_N2' step to form the formal S_N2 product **28** via *syn* attack of PhSH. Gas phase SCF energies (BP86) are shown in black with free energies at the BP86-D3($CHCl_3$) in red. All energies are in kcal/mol and are quoted relative to **5'** and the separated reactants set to zero.

Table S1. Computed relative energies (kcal/mol) for the reactions **4'** with PhSH. Energies are reported as ΔE (gas-phase SCF energies), ΔH_{298} (zero-point energy corrected energies at 298.15 K), ΔG (free energies at 298.15 K and 1 atm), ΔG_{disp} (including a correction for dispersion effects using Grimme's D3 parameter set) and $\Delta G_{CHCl3+disp}$ including an additional correction for $CHCl_3$ solvent via the PCM approach.

	**ΔE**	**ΔH_{298}**	**ΔG**	**ΔG_{disp}**	**$\Delta G_{CHCl3+disp}$**
**5'**	0.0	0.0	0.0	0.0	0.0
***IntI$_{4'}$ (anti)**	+7.2	+7.1	+7.5	+3.5	+0.9
***IntI$_{4'}$ (syn)**	+2.1	+2.1	+2.7	-1.2	-2.4
**IntI$_{4'}$ (anti)**	+2.6	+4.5	+14.2	+3.7	+2.3
**IntI$_{4'}$ (syn)**	-4.6	-2.6	+8.1	-5.4	-2.5
**TS(I-II)$_{4'}$ (anti)**	+3.6	+4.9	+17.2	+5.5	+9.1
**TS(I-II)$_{4'}$ (syn)**	+13.6	+14.4	+26.5	+14.6	+19.2
**Int(I-II)$_{4'}$ (syn)**	+4.3	+6.1	+19.3	+6.3	+9.9
**TS(I-II^{B})$_{4'}$ (syn)**	+6.9	+8.9	+21.3	+7.9	+11.1
**IntII$_{4'}$ (anti)**	-10.2	-7.0	+3.8	-6.5	-3.9
**IntII$_{4'}$ (syn)**	-13.9	-10.7	+0.5	-12.1	-8.9
***IntII$_{4'}$ (anti)**	-1.5	-0.4	+0.4	-5.4	-6.7
***IntII$_{4'}$ (syn)**	-1.5	-0.4	-0.2	-6.5	-6.8
**IntIII$_{4'}$ (anti)**	-4.4	-1.5	+8.3	-5.0	-3.6
**TS(III-IV^{A})$_{4'}$ (anti)**	+3.2	+5.2	+19.3	+0.4	+5.0
**Int(III-IV)$_{4'}$ (anti)**	+2.3	+4.3	+19.3	+0.1	+6.5
**TS(III-IV)$_{4'}$ (anti)**	+4.7	+6.5	+21.0	+2.7	+7.7
**IntIV$_{4'}$ (anti)**	-1.0	+1.8	+10.1	-4.4	-2.1
***IntIV$_{4'}$ (anti)**	+2.6	+3.5	+5.0	-3.0	-3.0
**$[(Me_3P)Au(SHPh)]^+$**	+3.0	+3.2	+3.5	+0.8	+0.3

*Complex computed in the absence of PhSH nucleophile

4′

16

```
C  -0.99096 -0.15998 -0.68191
C  -2.17978 -0.01659 -0.07576
C   0.35717  0.00249  0.01047
O   0.20519  0.14049  1.43509
C   1.03076  1.30972 -0.45019
C   1.25736 -1.21481 -0.30513
H   2.22975 -1.10254  0.20221
H   1.43651 -1.31724 -1.38957
H   0.78570 -2.15082  0.04411
H   1.16932  1.31718 -1.54380
H   2.01740  1.41503  0.03148
H   0.40537  2.17124 -0.16821
H  -0.22765 -0.68029  1.74234
H  -2.23746  0.25721  0.98320
H  -3.12229 -0.14623 -0.61626
H  -0.94546 -0.41252 -1.75106
```

SCF(BP86) = -271.747350648
H 0K = -271.610487
H 298K = -271.602862
G 298K = -271.641019
Solvent Correction(CHCl3) = -0.00299837
BP86-D3 Correction = -0.01360767
Lowest frequencies = 114.4346 cm-1, 221.6111 cm-1

Acetonitrile

6

```
C  -0.27724  0.00001  0.00000
N  -1.45073 -0.00001 -0.00000
C   1.18521 -0.00000 -0.00000
H   1.56908 -0.75455 -0.70605
H   1.56909 -0.23420  1.00648
H   1.56911  0.98872 -0.30042
```

SCF(BP86) = -132.751073663
H 0K = -132.706992
H 298K = -132.703349
G 298K = -132.731053
Solvent Correction(CH3Cl) = -0.00550907
BP86-D3 Correction = -0.00127441
Lowest frequencies = 373.9768 cm-1, 373.9955 cm-1

2a

13

```
S  -2.30589 -0.08411 -0.00001
C  -0.50937  0.00314 -0.00001
H  -2.52407  1.25935  0.00017
C   2.31065  0.00157 -0.00001
C   1.60229  1.21347  0.00000
C   0.19915  1.22104  0.00000
C   0.19915 -1.21626  0.00001
C   1.60116 -1.21052  0.00001
H   3.40497  0.00149 -0.00001
H   2.14160  2.16667  0.00001
H  -0.34123  2.17370 -0.00002
H  -0.34553 -2.16639  0.00003
H   2.14038 -2.16366  0.00001
```

SCF(BP86) = -242.466521945
H 0K = -242.370169
H 298K = -242.363647
G 298K = -242.401071
Solvent Correction(CHCl3) = -0.00332263
BP86-D3 Correction = -0.00877366
Lowest frequencies = 95.0392 cm-1, 178.2693 cm-1

3a'

```
26

C  -2.16709  0.60438 -0.17525
C  -1.37907  1.66760  0.51651
S   0.18249  2.17621 -0.41776
C  -2.33255 -0.68389  0.21555
C   1.17361  0.67743 -0.25689
C  -1.73770 -1.29090  1.46500
C  -3.16912 -1.63372 -0.61323
H  -4.01542 -2.03642 -0.02447
H  -2.57355 -2.50909 -0.93569
H  -3.57922 -1.14705 -1.51285
H  -1.09235 -2.15014  1.20379
H  -2.53297 -1.68554  2.12551
H  -1.12806 -0.58548  2.04853
H  -1.93699  2.61994  0.55964
H  -1.08109  1.40184  1.54148
H  -2.63601  0.91759 -1.11834
C   2.77000 -1.63199 -0.02612
C   2.93508 -0.57000  0.87853
C   2.13900  0.58100  0.76698
C   1.01646 -0.38677 -1.16911
C   1.81079 -1.53863 -1.04808
H   3.39273 -2.52858  0.06134
H   3.68607 -0.63610  1.67305
H   2.25997  1.41365  1.46719
H   0.27505 -0.29967 -1.96800
H   1.68555 -2.36129 -1.76038


SCF(BP86) = -437.80402827
H 0K = -437.591476
H 298K = -437.578329
G 298K = -437.632829
Solvent Correction(CHCl3) = -0.00345369
BP86-D3 Correction = -0.02579540
Lowest frequencies = 24.0999 cm-1, 37.3450 cm-1
```

28

26

```
C   1.83749  1.37291 -0.15835
C   1.38694  2.34833  0.65188
C   1.93154 -0.09594  0.16767
S   0.72440 -1.02266 -1.00338
C   1.62145 -0.44487  1.62664
C   3.30955 -0.64785 -0.25394
H   3.35762 -1.74061 -0.11044
H   4.09713 -0.18044  0.36243
H   3.52729 -0.42509 -1.31240
H   2.32493  0.07962  2.29855
H   1.72673 -1.52936  1.79043
H   0.59622 -0.15415  1.90682
C  -0.89811 -0.46442 -0.45291
H   1.03371  2.15172  1.66881
H   1.35732  3.38978  0.31690
H   2.17315  1.63194 -1.17248
C  -3.48689  0.33057  0.32829
C  -2.93943 -0.86425  0.82366
C  -1.64953 -1.26170  0.43715
C  -1.45503  0.73213 -0.95233
C  -2.74336  1.12657 -0.55867
H  -4.49422  0.63806  0.62825
H  -3.51750 -1.49097  1.51110
H  -1.21652 -2.19345  0.81391
H  -0.87435  1.34108 -1.65027
H  -3.16967  2.05564 -0.95197
```

SCF(BP86) = -437.796345583
H 0K = -437.583963
H 298K = -437.571076
G 298K = -437.623488
Solvent Correction(CHCl3) = -0.00327463
BP86-D3 Correction = -0.02890771
Lowest frequencies = 30.5930 cm-1, 62.5830 cm-1

5′

20

```
Au  0.00000  0.00000  0.27882
P   0.00000  0.00000 -2.01876
C   0.00002  1.70667 -2.73020
H  -0.89591  2.24702 -2.38882
H   0.89598  2.24699 -2.38883
C  -1.47803 -0.85332 -2.73020
H  -1.42910 -0.82507 -3.83091
H  -1.49802 -1.89939 -2.38882
H  -2.39394 -0.34756 -2.38883
C   1.47801 -0.85335 -2.73020
H   1.42908 -0.82510 -3.83091
H   2.39393 -0.34763 -2.38882
H   1.49796 -1.89943 -2.38883
N   0.00000  0.00000  2.34123
C   0.00000  0.00000  3.50951
C   0.00000  0.00000  4.96207
H   0.00002  1.65017 -3.83091
H  -0.79509  0.66685  5.33517
H   0.97506  0.35514  5.33517
H  -0.17997 -1.02200  5.33517
```

SCF(BP86) = -394.816760183
H 0K = -394.658816
H 298K = -394.645144
G 298K = -394.701058
Solvent Correction(CHCl3) = -0.05231851
BP86-D3 Correction = -0.01603484
Lowest frequencies = 14.6238 cm-1, 37.9964 cm-1

*IntI$_{4'}$ (anti)

30

```
P  -2.38553  0.39539  0.03669
C  -2.61981  1.77228  1.25112
C  -2.98787  1.04302 -1.58944
C  -3.58805 -0.92231  0.53129
Au -0.18921 -0.42411 -0.05359
C   2.13004 -0.59200 -0.54573
C   1.65623 -1.70250  0.14346
C   2.97359  0.55663  0.02488
H  -3.67524  2.08986  1.25560
H  -1.98209  2.62441  0.97111
H  -2.33606  1.42724  2.25700
H  -3.54842 -1.74829 -0.19501
H  -4.60747 -0.50412  0.55807
H  -3.32304 -1.30823  1.52739
H  -4.02909  1.39042 -1.48920
H  -2.93810  0.24331 -2.34396
H  -2.35091  1.88039 -1.91245
O   4.30846  0.14845 -0.34949
C   2.96534  0.63856  1.55409
C   2.60379  1.89832 -0.62370
H   3.32689  2.67106 -0.31612
H   1.59601  2.22839 -0.31760
H   2.61427  1.82819 -1.72688
H   1.94971  0.83132  1.93965
H   3.62149  1.46171  1.87563
H   3.35342 -0.28925  2.00295
H   4.48308  0.45840 -1.26069
H   1.81333 -1.80573  1.22339
H   1.37898 -2.61448 -0.39935
H   2.11635 -0.63019 -1.64621
```

SCF(BP86) = -533.80159362
H 0K = -533.551183
H 298K = -533.533388
G 298K = -533.599102
Solvent Correction(CHCl3) = -0.05404296
BP86-D3 Correction = -0.03464755
Lowest frequencies = 9.3216 cm-1, 36.9935 cm-1

$*IntI_{4'}$ (syn)

```
30

P  -2.36256  0.37978  0.04411
C  -2.95662  1.11438 -1.54786
C  -3.57013 -0.96003  0.46082
C  -2.60012  1.69007  1.32916
Au -0.16563 -0.42041 -0.06584
C   2.15513 -0.62043 -0.58531
C   1.68152 -1.70422  0.13830
C   2.88651  0.55953  0.03127
H  -3.99815  1.45688 -1.43479
H  -2.90258  0.35646 -2.34400
H  -2.31740  1.96735 -1.82180
H  -2.32145  1.28918  2.31556
H  -3.65379  2.01295  1.34575
H  -1.95465  2.55143  1.09972
H  -4.58923 -0.54250  0.50460
H  -3.31053 -1.39889  1.43618
H  -3.52710 -1.74602 -0.30834
O   2.47393  0.59799  1.40674
C   2.52964  1.87532 -0.69009
C   4.40599  0.26438 -0.07686
H   4.97124  1.11490  0.34298
H   4.72759  0.14029 -1.12382
H   4.66456 -0.64581  0.48618
H   2.80167  1.83813 -1.75843
H   3.08010  2.71555 -0.23424
H   1.44978  2.08442 -0.60620
H   3.01751  1.26930  1.86191
H   1.83336 -1.74102  1.22316
H   1.38964 -2.62910 -0.37291
H   2.17974 -0.67551 -1.68229


SCF(BP86) = -533.809627303
H 0K = -533.559084
H 298K = -533.541282
G 298K = -533.606765
Solvent Correction(CHCl3) = -0.05168875
BP86-D3 Correction = -0.03461175
Lowest frequencies = 10.8845 cm-1, 37.5183 cm-1
```

IntI$_{4'}$ (anti)

43

```
P  -3.73380 -0.91048  0.30784
C  -3.62067 -2.29129  1.53623
C  -4.87765  0.33603  1.06182
C  -4.64360 -1.60524 -1.14716
Au -1.64309 -0.01581 -0.27087
C   0.15984  1.33598 -0.72341
C   0.54476  0.03207 -1.03435
S   3.55044  0.32616 -1.86055
C   0.66153  2.14593  0.48600
H  -4.62901 -2.67363  1.76405
H  -3.14909 -1.92369  2.46024
H  -3.00522 -3.10326  1.11995
H  -4.80525 -0.81148 -1.89228
H  -5.61615 -2.00981 -0.82273
H  -4.04576 -2.40805 -1.60486
H  -5.84197 -0.14051  1.30227
H  -5.04289  1.16076  0.35198
H  -4.42845  0.73918  1.98212
C   4.44426 -0.47334 -0.51697
H   3.16533  1.43452 -1.15648
O   1.88654  2.77176  0.00899
C   1.07801  1.28595  1.68372
C  -0.35580  3.22365  0.88639
H   0.07380  3.87921  1.66075
H  -1.27667  2.76825  1.28982
H  -0.64436  3.84625  0.01960
H   0.22663  0.69315  2.05934
H   1.43089  1.94071  2.49545
H   1.90343  0.60406  1.42358
H   1.64450  3.56822 -0.50478
H   1.09473 -0.59356 -0.32481
H   0.43776 -0.36111 -2.05098
H  -0.22618  1.95718 -1.54726
C   5.87903 -1.80491  1.50403
C   5.48241 -0.46972  1.68174
C   4.77020  0.20328  0.67645
C   4.83977 -1.81395 -0.70071
C   5.55834 -2.47050  0.30945
H   6.44088 -2.32116  2.28806
H   5.73759  0.06185  2.60429
H   4.48371  1.25122  0.81209
H   4.59223 -2.33928 -1.62923
H   5.86871 -3.50930  0.15822
```

SCF(BP86) = -776.275343906
H 0K = -775.927161
H 298K = -775.901166
G 298K = -775.989486
Solvent Correction(CHCl3) = -0.05535675
BP86-D3 Correction = -0.05392868
Lowest frequencies = 6.6475 cm-1, 9.2485 cm-1

$IntI_{4'}$ (syn)

43

```
P  -2.61653 -1.48207  0.13651
C  -2.26076 -3.18549 -0.50037
C  -2.85611 -1.69235  1.96182
C  -4.29183 -1.05569 -0.53135
Au -0.91314  0.02842 -0.43884
C  -0.21221  2.25303 -0.96721
C   0.69090  1.29385 -1.40328
S   1.25962 -1.08569  1.40801
C  -0.04509  3.09290  0.28825
H  -3.06558 -3.87629 -0.20090
H  -1.30231 -3.53450 -0.08719
H  -2.18822 -3.16009 -1.59837
H  -4.60759 -0.07994 -0.13196
H  -5.02426 -1.82696 -0.24190
H  -4.23956 -0.99007 -1.62875
H  -3.63732 -2.44513  2.15635
H  -3.15472 -0.73168  2.40836
H  -1.90875 -2.01929  2.41723
C   2.87293 -1.07622  0.60274
H   1.20433  0.25298  1.67391
O   0.85864  2.37521  1.16120
C   0.59798  4.43618 -0.14137
C  -1.39646  3.32818  0.99062
H  -2.10983  3.84146  0.32408
H  -1.25649  3.96350  1.88178
H  -1.83799  2.36967  1.30971
H   0.71621  5.08734  0.74279
H  -0.03311  4.97680 -0.86546
H   1.58787  4.26481 -0.59199
H   1.06394  2.96833  1.90955
H   0.64377  0.92970 -2.43703
H   1.61164  1.09168 -0.84551
H  -1.00159  2.59698 -1.64823
C   5.38831 -1.17046 -0.64611
C   4.60443 -2.33223 -0.54739
C   3.34724 -2.29292  0.07370
C   3.65331  0.09427  0.50994
C   4.90941  0.03777 -0.11681
H   6.36924 -1.20892 -1.12853
H   4.97261 -3.27998 -0.95241
H   2.74535 -3.20429  0.15278
H   3.28662  1.03775  0.92798
H   5.51614  0.94658 -0.18250
```

SCF(BP86) = -776.286893066
H 0K = -775.938492
H 298K = -775.912506
G 298K = -775.999131
Solvent Correction(CHCl3) = -0.04861740
BP86-D3 Correction = -0.05865049
Lowest frequencies = 15.5028 cm-1, 16.0381 cm-1

TS(I-II)$_{4'}$ (syn)

43

```
P  -2.80862 -1.61166  0.34082
C  -2.16429 -3.28119 -0.14009
C  -3.07187 -1.70484  2.17303
C  -4.50936 -1.51671 -0.38973
Au -1.40441  0.14163 -0.30252
C  -0.14375  1.86244 -0.92947
C   1.00788  1.30340 -1.53696
S   1.71095 -0.69863 -0.14968
C   0.04790  2.87291  0.21937
H  -2.84856 -4.06982  0.21297
H  -1.16888 -3.42816  0.30615
H  -2.07429 -3.33532 -1.23574
H  -4.98748 -0.57465 -0.08085
H  -5.11909 -2.36882 -0.04767
H  -4.43590 -1.53361 -1.48781
H  -3.73089 -2.55305  2.42016
H  -3.53110 -0.76838  2.52466
H  -2.10083 -1.83449  2.67476
C   3.48699 -0.90738  0.05756
H   1.52960  0.28311  0.78949
O   0.78770  2.12132  1.23286
C   0.88145  4.09336 -0.22412
C  -1.30188  3.33237  0.79546
H  -1.89934  3.86030  0.03248
H  -1.14841  4.03040  1.63678
H  -1.88411  2.46825  1.15966
H   1.02319  4.79109  0.62026
H   0.37200  4.64903 -1.02874
H   1.87696  3.79257 -0.58854
H   0.96913  2.72796  1.97709
H   0.92119  0.77714 -2.49293
H   2.01439  1.64481 -1.27820
H  -0.93693  2.12418 -1.65915
C   6.25377 -1.28611  0.27962
C   5.48804 -2.15513 -0.51573
C   4.10286 -1.97252 -0.63208
C   4.24928 -0.02620  0.85234
C   5.63286 -0.22590  0.96137
H   7.33372 -1.43569  0.36944
H   5.96849 -2.98295 -1.04601
H   3.50573 -2.65512 -1.24583
H   3.76612  0.80225  1.38041
H   6.22677  0.45159  1.58264
```

SCF(BP86) = -776.257851827
H 0K = -775.910311
H 298K = -775.885327
G 298K = -775.969884
Solvent Correction(CHCl3) = -0.04588647
BP86-D3 Correction = -0.05602818
Lowest frequencies = -134.4843 cm-1, 11.1992 cm-1

TS(I-II)$_{4'}$ (anti)

43

```
P  -3.70942 -0.92471  0.29568
C  -3.65999 -2.12435  1.70806
C  -4.99335  0.32550  0.76958
C  -4.45292 -1.86699 -1.11681
Au -1.63046  0.03454 -0.21831
C   0.22368  1.10549 -0.72420
C   0.86713 -0.14912 -0.93666
S   3.22569  0.28112 -1.70107
C   0.68863  2.08118  0.38097
H  -4.66671 -2.53244  1.89462
H  -3.29856 -1.61088  2.61205
H  -2.96917 -2.94629  1.46596
H  -4.58417 -1.19291 -1.97697
H  -5.43023 -2.28305 -0.82257
H  -3.77701 -2.68578 -1.40695
H  -5.94990 -0.17686  0.98698
H  -5.13308  1.03860 -0.05720
H  -4.65772  0.87618  1.66159
C   4.30624 -0.47692 -0.47995
H   2.94862  1.45092 -1.00665
O   1.95362  2.68420 -0.07434
C   1.03621  1.39432  1.70708
C  -0.33544  3.20639  0.58569
H   0.06125  3.96064  1.28436
H  -1.27705  2.80782  1.00024
H  -0.58054  3.70430 -0.37058
H   0.15606  0.86771  2.11215
H   1.36312  2.14879  2.43952
H   1.85613  0.66780  1.58716
H   1.73213  3.40240 -0.70117
H   1.26528 -0.72935 -0.09879
H   0.66398 -0.71322 -1.85137
H  -0.06789  1.63409 -1.64902
C   6.02382 -1.73326  1.34580
C   5.57974 -0.42220  1.58468
C   4.71985  0.21473  0.67718
C   4.74947 -1.79166 -0.72925
C   5.60849 -2.41320  0.18890
H   6.69762 -2.22138  2.05610
H   5.90841  0.11581  2.47937
H   4.38583  1.24102  0.85861
H   4.43448 -2.32056 -1.63481
H   5.95740 -3.43191 -0.00615
```

SCF(BP86) = -776.27388003
H 0K = -775.925124
H 298K = -775.900556
G 298K = -775.984692
Solvent Correction(CHCl3) = -0.04746359
BP86-D3 Correction = -0.05570422
Lowest frequencies = -99.9387 cm-1, 3.1439 cm-1

Int(I-II)$_{4'}$ (syn)

43

```
P  -3.41031 -1.05216  0.31517
C  -3.32654 -2.86044 -0.09873
C  -3.81282 -1.01735  2.12802
C  -4.96727 -0.46625 -0.51038
Au -1.47758  0.15878 -0.28993
C   0.25840  1.21534 -0.93402
C   1.39283  0.24185 -1.28195
S   1.80047 -0.78010  0.25950
C   0.67746  2.38281 -0.02636
H  -4.25475 -3.37118  0.20503
H  -2.46976 -3.31386  0.42295
H  -3.18112 -2.97853 -1.18353
H  -5.14754  0.58663 -0.24417
H  -5.82802 -1.07755 -0.19401
H  -4.84825 -0.53715 -1.60249
H  -4.72158 -1.60556  2.33506
H  -3.97068  0.02457  2.44651
H  -2.96779 -1.43561  2.69625
C   3.60501 -0.93614  0.19892
H   1.40241  0.71347  1.15878
O   0.91650  1.70096  1.38712
C   2.00278  3.07319 -0.37554
C  -0.43918  3.39107  0.21792
H  -0.65815  3.91716 -0.72653
H  -0.14604  4.15070  0.96213
H  -1.35629  2.88230  0.55250
H   2.22735  3.89643  0.32601
H   1.91975  3.52160 -1.37948
H   2.85907  2.38007 -0.38912
H   1.52796  2.25776  1.91985
H   1.07719 -0.46680 -2.06141
H   2.34361  0.70806 -1.59633
H  -0.07028  1.68564 -1.88272
C   6.38786 -1.26640  0.11097
C   5.55666 -2.11061 -0.64365
C   4.16253 -1.95640 -0.59668
C   4.43467 -0.09191  0.96264
C   5.82716 -0.26204  0.91649
H   7.47372 -1.39618  0.07747
H   5.99170 -2.89995 -1.26417
H   3.51184 -2.62524 -1.16866
H   4.00010  0.68568  1.59932
H   6.47244  0.39007  1.51312
```

SCF(BP86) = -776.27276943
H 0K = -775.922795
H 298K = -775.898535
G 298K = -775.981265
Solvent Correction(CHCl3) = -0.04741320
BP86-D3 Correction = -0.05791403
Lowest frequencies = 5.6008 cm-1, 14.7306 cm-1

TS(I-II^B)$_{4'}$ (syn)

43

```
P  -3.32714 -1.11009  0.30878
C  -3.15344 -2.90343 -0.13090
C  -3.68312 -1.10305  2.12939
C  -4.91820 -0.58082 -0.48526
Au -1.46818  0.17792 -0.30475
C   0.27436  1.28679 -0.96121
C   1.40583  0.32540 -1.33158
S   1.76583 -0.78325  0.14908
C   0.62026  2.43522 -0.10959
H  -4.04669 -3.46512  0.18743
H  -2.26188 -3.31536  0.36606
H  -3.02681 -3.00034 -1.22002
H  -5.13970  0.45954 -0.20198
H  -5.74481 -1.23399 -0.16145
H  -4.81425 -0.63465 -1.57976
H  -4.56095 -1.73125  2.35188
H  -3.87632 -0.07088  2.45944
H  -2.80718 -1.48982  2.67235
C   3.57086 -0.91965  0.12553
H   1.00486  0.75184  1.43114
O   0.51231  1.60750  1.67753
C   2.05515  2.90992 -0.00243
C  -0.41612  3.52219  0.03411
H  -0.26807  4.24807 -0.78783
H  -0.29563  4.07953  0.97600
H  -1.43897  3.12225 -0.02494
H   2.17807  3.69668  0.75921
H   2.32190  3.36331 -0.97686
H   2.77497  2.09895  0.18403
H   1.09503  2.07414  2.31573
H   1.09928 -0.32301 -2.16519
H   2.35870  0.81275 -1.60265
H  -0.22290  1.70008 -1.86553
C   6.36355 -1.21468  0.14604
C   5.62157 -1.61648 -0.97744
C   4.22446 -1.48639 -0.98756
C   4.31184 -0.51600  1.25412
C   5.70734 -0.67349  1.26272
H   7.45156 -1.32994  0.15315
H   6.12874 -2.05002 -1.84505
H   3.64533 -1.83229 -1.84945
H   3.80088 -0.09051  2.12422
H   6.28052 -0.36526  2.14272
```

SCF(BP86) = -776.268523852
H 0K = -775.918748
H 298K = -775.894168
G 298K = -775.978096
Solvent Correction(CHCl3) = -0.04805956
BP86-D3 Correction = -0.05855697
Lowest frequencies = -201.7304 cm-1, 6.6371 cm-1

IntII$_{4'}$ (anti)

43

```
P  -3.73735 -0.91805  0.22963
C  -3.74926 -2.09233  1.66126
C  -5.11296  0.27884  0.55325
C  -4.27872 -1.91279 -1.23565
Au -1.66783  0.12516 -0.10587
C   0.37540  0.78274 -0.82265
C   1.33418 -0.38453 -0.82643
S   2.97704  0.21204 -1.56012
C   0.15022  1.66343  0.25320
H  -4.74497 -2.55443  1.76042
H  -3.50338 -1.54698  2.58512
H  -2.99444 -2.87660  1.49838
H  -4.35927 -1.25647 -2.11549
H  -5.25710 -2.37768 -1.03214
H  -3.53496 -2.69729 -1.44232
H  -6.06065 -0.26715  0.68966
H  -5.20566  0.96979 -0.29841
H  -4.88905  0.85903  1.46130
C   4.17484 -0.57586 -0.45706
H   3.06170  2.38179 -0.53682
O   2.94116  3.08984  0.13914
C   0.70534  1.43844  1.63569
C  -0.38832  3.04903 -0.01194
H   0.49425  3.71889  0.02012
H  -1.09099  3.38339  0.76840
H  -0.85991  3.14518 -1.00233
H   0.02133  1.82468  2.40882
H   1.64101  2.03068  1.68977
H   0.94500  0.38785  1.85632
H   3.67555  3.71071 -0.01803
H   1.55617 -0.76745  0.17932
H   0.97372 -1.20921 -1.45972
H   0.12380  1.17315 -1.82155
C   6.10015 -1.77649  1.18886
C   5.63554 -0.48326  1.48158
C   4.67235  0.12460  0.66124
C   4.64564 -1.86950 -0.76076
C   5.60705 -2.46703  0.06909
H   6.85406 -2.24405  1.82987
H   6.02797  0.05864  2.34800
H   4.30859  1.13634  0.87248
H   4.26880 -2.39275 -1.64495
H   5.97695 -3.47002 -0.16578
```

SCF(BP86) = -776.29580923
H 0K = -775.945849
H 298K = -775.919443
G 298K = -776.006059
Solvent Correction(CHCl3) = -0.04908504
BP86-D3 Correction = -0.05346922
Lowest frequencies = 15.1505 cm-1, 18.8582 cm-1

IntII$_{4'}$ (syn)

43

```
P  -2.65898 -1.59693 -0.05565
C  -2.01567 -2.85410 -1.24640
C  -2.61823 -2.40879  1.60232
C  -4.44318 -1.33471 -0.47875
Au -1.39788  0.37412 -0.10215
C   0.17430  2.01032 -0.42460
C   1.53307  1.50892  0.00575
S   2.01648  0.02067 -1.01497
C  -0.77465  2.62684  0.40455
H  -2.63720 -3.76326 -1.20168
H  -0.98257 -3.07831 -0.93690
H  -2.02525 -2.44632 -2.26834
H  -4.89682 -0.63985  0.24424
H  -4.97885 -2.29750 -0.44695
H  -4.52306 -0.90414 -1.48851
H  -3.18883 -3.35138  1.57238
H  -3.05321 -1.73712  2.35776
H  -1.56133 -2.60646  1.83881
C   3.69052 -0.26671 -0.38699
H   1.02397 -1.71177  0.15843
O   0.60672 -2.38360  0.75357
C  -0.62068  2.74333  1.90482
C  -1.86835  3.48195 -0.20362
H  -1.61603  4.54535 -0.02866
H  -2.84267  3.30586  0.28153
H  -1.97102  3.33133 -1.28920
H  -1.59846  2.66826  2.40811
H  -0.21872  3.74736  2.14069
H   0.06085  1.99796  2.33960
H   1.19864 -3.15637  0.69534
H   2.28374  2.29505 -0.19502
H   1.57915  1.24567  1.07265
H   0.06964  2.18804 -1.50609
C   6.29057 -0.73711  0.54544
C   6.09269 -0.02758 -0.65037
C   4.79218  0.21214 -1.12254
C   3.88398 -0.99017  0.80758
C   5.18835 -1.21767  1.27254
H   7.30616 -0.92128  0.90906
H   6.95142  0.34011 -1.22058
H   4.62900  0.76021 -2.05568
H   3.01824 -1.37739  1.35615
H   5.34335 -1.77717  2.20048
```

SCF(BP86) = -776.301666871
H 0K = -775.951726
H 298K = -775.925364
G 298K = -776.011234
Solvent Correction(CHCl3) = -0.04793618
BP86-D3 Correction = -0.05730587
Lowest frequencies = 11.1702 cm-1, 30.5728 cm-1

$*IntII_{4'}$ (anti)

40

```
P   3.27725 -1.13717 -0.06390
C   4.83835 -0.15424  0.10148
C   3.36543 -2.43969  1.24879
C   3.42496 -2.03788 -1.67496
Au  1.34575  0.18055  0.08479
C  -0.82149  0.91213  0.06022
C  -1.45535  0.63916 -1.27909
S  -3.22402  1.25576 -1.22530
C  -0.07662  2.05114  0.41760
H   5.71088 -0.82356  0.02678
H   4.84967  0.35660  1.07625
H   4.88418  0.59998 -0.69874
H   2.55776 -2.70300 -1.80514
H   4.35125 -2.63507 -1.68698
H   3.44528 -1.31114 -2.50128
H   4.29136 -3.02647  1.13437
H   2.49431 -3.10691  1.16285
H   3.35248 -1.96241  2.24046
C  -4.04351 -0.11123 -0.37405
C   0.30772  3.12768 -0.57340
C   0.06745  2.43235  1.87762
H  -0.62784  3.26839  2.08654
H   1.08091  2.79839  2.11143
H  -0.18552  1.60519  2.55894
H   1.27460  3.58673 -0.31015
H  -0.45251  3.93139 -0.52401
H   0.35374  2.77736 -1.61481
H  -0.97857  1.17921 -2.11082
H  -1.47983 -0.43384 -1.52093
H  -1.24932  0.32897  0.88987
C  -5.42052 -2.18843  0.92159
C  -4.98082 -2.35627 -0.40297
C  -4.30251 -1.31762 -1.05809
C  -4.48825  0.06023  0.95256
C  -5.18000 -0.97992  1.59485
H  -5.96101 -2.99643  1.42434
H  -5.18456 -3.29078 -0.93537
H  -3.98989 -1.43000 -2.10139
H  -4.30307  1.00788  1.46785
H  -5.53218 -0.84292  2.62217
```

SCF(BP86) = -699.864008888
H 0K = -699.538119
H 298K = -699.514645
G 298K = -699.595954
Solvent Correction(CHCl3) = -0.05030723
BP86-D3 Correction = -0.04641935
Lowest frequencies = 12.8400 cm-1, 15.6727 cm-1

***IntII$_{4'}$ (syn)**

40

```
P  -2.45782 -1.76917  0.07319
C  -1.69391 -3.02897 -1.04621
C  -2.39898 -2.50495  1.77068
C  -4.24999 -1.70010 -0.38596
Au -1.34118  0.27753 -0.08079
C   0.15087  1.95741 -0.57046
C   1.52458  1.53510 -0.10669
S   1.83300 -0.22865 -0.63989
C  -0.82629  2.61068  0.19489
H  -2.20327 -3.99869 -0.92414
H  -0.62695 -3.13417 -0.79720
H  -1.78351 -2.69341 -2.09056
H  -4.77758 -1.01398  0.29386
H  -4.69506 -2.70577 -0.31234
H  -4.34940 -1.32848 -1.41712
H  -2.90583 -3.48371  1.77281
H  -2.89867 -1.83051  2.48251
H  -1.34899 -2.63298  2.07463
C   3.57893 -0.41919 -0.20306
C  -0.68001  2.88845  1.67468
C  -1.96059  3.34414 -0.49322
H  -1.75991  4.43081 -0.43094
H  -2.92583  3.17328  0.01143
H  -2.05503  3.07708 -1.55700
H  -1.65238  2.80837  2.18775
H  -0.33785  3.93273  1.80626
H   0.04273  2.23096  2.17889
H   2.27696  2.17665 -0.59901
H   1.65237  1.61525  0.98252
H   0.02731  1.99809 -1.66357
C   6.28651 -0.77498  0.43594
C   5.91585 -0.42188 -0.87192
C   4.56187 -0.24329 -1.19780
C   3.94722 -0.78516  1.10725
C   5.30378 -0.95637  1.42356
H   7.34295 -0.91480  0.68512
H   6.68048 -0.28734 -1.64341
H   4.26390  0.02570 -2.21598
H   3.17471 -0.94024  1.86732
H   5.59219 -1.23938  2.44085
```

SCF(BP86) = -699.864072054
H 0K = -699.538288
H 298K = -699.514708
G 298K = -699.596959
Solvent Correction(CHCl3) = -0.04872543
BP86-D3 Correction = -0.04717095
Lowest frequencies = 7.7748 cm-1, 14.7686 cm-1

IntIII$_{4'}$ (anti)

53

P	4.490712	0.178740	0.093389
C	5.026624	0.788416	1.757543
C	5.467477	-1.364632	-0.212322
C	5.130910	1.420263	-1.122370
Au	2.180732	-0.139717	-0.081018
C	-0.029874	-0.012470	-0.508187
C	-0.555575	1.315101	-0.014514
S	-2.396844	1.354517	-0.405641
C	0.018706	-1.204775	0.241844
H	6.120038	0.926292	1.773123
H	4.737145	0.056640	2.526990
H	4.531800	1.747559	1.973296
H	4.904641	1.082671	-2.145210
H	6.220942	1.532698	-1.004238
H	4.640438	2.390311	-0.949767
H	6.545168	-1.148542	-0.130101
H	5.244510	-1.745971	-1.220383
H	5.188245	-2.128855	0.528806
C	-2.738127	3.121491	-0.251058
H	-3.937492	-0.453626	1.172138
S	-4.083014	-1.629330	1.852439
C	-0.270355	-1.261072	1.719524
C	0.117609	-2.538123	-0.462059
H	-0.872468	-3.031579	-0.399472
H	0.836966	-3.211031	0.034091
H	0.382083	-2.438384	-1.525971
H	0.345017	-2.028010	2.217650
H	-1.332405	-1.559834	1.853835
H	-0.133624	-0.298566	2.233849
C	-3.743914	-2.710851	0.452942
H	-0.407415	1.469541	1.064773
H	-0.101763	2.151795	-0.567443
H	-0.049711	-0.131577	-1.602634
C	-3.358508	5.849919	-0.038731
C	-3.308367	5.050558	1.115780
C	-3.003358	3.684592	1.014442
C	-2.792091	3.921111	-1.411182
C	-3.103737	5.285286	-1.299464
H	-3.604561	6.913161	0.043899
H	-3.518063	5.487863	2.097066
H	-2.982149	3.051976	1.907414
H	-2.601355	3.469532	-2.389642
H	-3.152719	5.905453	-2.200071
C	-3.224998	-4.506186	-1.660294
C	-3.113463	-4.932474	-0.325762
C	-3.362706	-4.040997	0.729962
C	-3.872459	-2.284464	-0.885496
C	-3.604824	-3.181098	-1.932394
H	-3.031452	-5.204285	-2.480309
H	-2.830569	-5.965851	-0.099969
H	-3.267177	-4.378818	1.767471
H	-4.195615	-1.263053	-1.109731
H	-3.713099	-2.841501	-2.967849

SCF(BP86) = -942.335190669
H 0K = -941.912104
H 298K = -941.880100
G 298K = -941.984512
Solvent Correction(CHCl3) = -0.04929878
BP86-D3 Correction = -0.06699874
Lowest frequencies = 11.0747 cm-1, 12.3887 cm-1

TS(III-IV^{A})$_{4'}$ (anti)

53

P	-4.372496	-0.404963	-0.057398
C	-4.906877	-2.180546	-0.046621
C	-5.246250	0.365093	1.386117
C	-5.184714	0.329486	-1.553505
Au	-2.050923	-0.115748	-0.038941
C	0.123505	0.143556	-0.218214
C	0.580734	-1.069794	-1.025388
S	2.380324	-1.049261	-1.645740
C	0.518887	0.348160	1.161910
H	-6.006786	-2.247884	-0.069060
H	-4.527048	-2.671240	0.862474
H	-4.489509	-2.692647	-0.927007
H	-4.977407	1.409935	-1.588029
H	-6.273788	0.164117	-1.516431
H	-4.772221	-0.139239	-2.459980
H	-6.332886	0.198307	1.306590
H	-5.041099	1.446378	1.402613
H	-4.873856	-0.082043	2.320435
C	3.168985	-2.381480	-0.709831
H	2.910180	0.363472	-0.177359
S	2.900377	1.155302	0.995262
C	0.840806	-0.835521	2.052313
C	0.022008	1.591330	1.876038
H	0.672628	1.849661	2.726566
H	-0.986604	1.398493	2.287463
H	-0.048354	2.457432	1.199109
H	-0.106635	-1.336013	2.332234
H	1.327816	-0.513441	2.985153
H	1.477907	-1.585617	1.558890
C	2.717071	2.751997	0.201765
H	0.471416	-2.014931	-0.473340
H	-0.008157	-1.149376	-1.950711
H	0.136250	1.082770	-0.801215
C	4.509782	-4.449953	0.643352
C	5.089899	-3.175027	0.556848
C	4.422011	-2.135110	-0.111186
C	2.598239	-3.669717	-0.651121
C	3.264638	-4.693532	0.039251
H	5.030812	-5.255559	1.169236
H	6.064558	-2.980278	1.015055
H	4.879972	-1.142888	-0.174127
H	1.652636	-3.880807	-1.160979
H	2.818229	-5.692049	0.082988
C	2.424708	5.304363	-0.932984
C	2.574497	5.165668	0.457247
C	2.721695	3.894644	1.030716
C	2.571764	2.885331	-1.195890
C	2.427717	4.164925	-1.754128
H	2.317896	6.298935	-1.376099
H	2.586382	6.050824	1.100662
H	2.855640	3.789913	2.112355
H	2.599160	2.003957	-1.843460
H	2.327399	4.268468	-2.839170

SCF(BP86) = -942.323020106
H 0K = -941.899615
H 298K = -941.869452
G 298K = -941.966890
Solvent Correction(CHCl3) = -0.04410199
BP86-D3 Correction = -0.07614278
Lowest frequencies = -84.1086 cm-1, 8.0984 cm-1

Int(III-IV)$_{4'}$ (anti)

53

P	-4.354402	-0.457534	-0.045515
C	-4.870386	-2.240980	-0.052479
C	-5.218403	0.265453	1.430459
C	-5.234757	0.282856	-1.502564
Au	-2.027570	-0.121476	-0.074629
C	0.101863	0.201119	-0.207045
C	0.617129	-1.014182	-0.965553
S	2.470233	-0.958180	-1.541011
C	0.657596	0.498042	1.168671
H	-5.969334	-2.324915	-0.045766
H	-4.458351	-2.743327	0.836132
H	-4.470411	-2.732101	-0.952804
H	-5.051534	1.368054	-1.525610
H	-6.318556	0.093779	-1.436104
H	-4.840620	-0.160522	-2.429840
H	-6.302747	0.076389	1.373561
H	-5.036821	1.350703	1.463440
H	-4.815106	-0.188773	2.348544
C	3.240019	-2.343759	-0.668821
H	2.768915	0.245056	-0.206553
S	2.606797	1.127989	1.015311
C	0.838891	-0.714711	2.090861
C	-0.026933	1.670376	1.875639
H	0.523138	1.968995	2.784415
H	-1.042155	1.360902	2.180017
H	-0.125306	2.546405	1.214453
H	-0.151561	-1.177055	2.252103
H	1.241315	-0.416814	3.072486
H	1.502246	-1.485587	1.664832
C	2.553708	2.729000	0.194389
H	0.550144	-1.952066	-0.394474
H	0.075599	-1.140906	-1.913226
H	0.153482	1.111910	-0.834113
C	4.522965	-4.493475	0.598959
C	5.076581	-3.205449	0.669175
C	4.437805	-2.123691	0.040910
C	2.692408	-3.638781	-0.763274
C	3.332953	-4.706980	-0.116631
H	5.023220	-5.332240	1.092069
H	6.008815	-3.034924	1.216465
H	4.877098	-1.122480	0.093879
H	1.785842	-3.816807	-1.350340
H	2.907998	-5.712896	-0.190118
C	2.530913	5.277549	-0.968700
C	2.667555	5.144046	0.423128
C	2.682785	3.871150	1.011809
C	2.424547	2.854651	-1.204359
C	2.414111	4.135606	-1.777997
H	2.525923	6.272322	-1.424228
H	2.770856	6.031526	1.054688
H	2.804078	3.762610	2.094089
H	2.355270	1.970947	-1.844140
H	2.321992	4.237403	-2.863664

SCF(BP86) = -942.324574194
H 0K = -941.900928
H 298K = -941.870780
G 298K = -941.966986
Solvent Correction(CHCl3) = -0.04143085
BP86-D3 Correction = -0.07643137
Lowest frequencies = 9.6317 cm-1, 18.3463 cm-1

Ts(III-IV)$_{4'}$ (anti)

53

P	-4.289409	-0.645725	-0.031448
C	-4.748839	-2.070986	1.062798
C	-5.289773	0.781612	0.601624
C	-5.032422	-1.051300	-1.681667
Au	-1.986666	-0.204095	-0.116918
C	0.140709	0.358120	-0.186299
C	0.487724	-0.860245	-0.877301
S	2.759818	-0.943864	-1.531867
C	0.680557	0.690922	1.206889
H	-5.841158	-2.218219	1.059681
H	-4.408766	-1.867609	2.089744
H	-4.253735	-2.984581	0.699817
H	-4.868012	-0.210113	-2.372275
H	-6.113975	-1.236548	-1.577983
H	-4.543484	-1.947909	-2.092206
H	-6.360822	0.522019	0.613057
H	-5.130408	1.655818	-0.047983
H	-4.962623	1.034036	1.621856
C	3.426457	-2.353983	-0.633161
H	2.911315	0.038560	-0.500524
S	2.497833	1.352059	1.042937
C	0.846265	-0.533415	2.122934
C	-0.149972	1.786308	1.889295
H	0.306527	2.086026	2.846854
H	-1.166871	1.402824	2.095951
H	-0.247661	2.680958	1.253044
H	-0.133091	-1.030028	2.249079
H	1.205330	-0.227106	3.118457
H	1.555938	-1.276409	1.721460
C	2.345531	2.939382	0.197107
H	0.580778	-1.797913	-0.318957
H	0.144055	-0.975095	-1.909711
H	0.099017	1.250491	-0.834313
C	4.477132	-4.605312	0.666083
C	4.767304	-3.317111	1.145009
C	4.247557	-2.183911	0.500547
C	3.135226	-3.642816	-1.124061
C	3.664483	-4.764735	-0.468318
H	4.890402	-5.483162	1.171497
H	5.409412	-3.187329	2.021736
H	4.492850	-1.181962	0.865297
H	2.511200	-3.768286	-2.015100
H	3.443662	-5.765721	-0.851572
C	2.219547	5.454534	-1.054027
C	2.245889	4.284586	-1.830863
C	2.313782	3.026829	-1.211151
C	2.330765	4.115832	0.976088
C	2.266111	5.368827	0.347116
H	2.174324	6.433307	-1.541361
H	2.226308	4.348842	-2.923364
H	2.361735	2.122022	-1.824555
H	2.379936	4.043560	2.066747
H	2.258918	6.278986	0.954881

SCF(BP86) = -942.320597146
H 0K = -941.897246
H 298K = -941.867302
G 298K = -941.964176
Solvent Correction(CHCl3) = -0.04361495
BP86-D3 Correction = -0.07516386
Lowest frequencies = -134.7252 cm-1, 8.1177 cm-1

IntIV$_{4'}$ (anti)

53

```
P  -2.47560 -2.95185  0.13673
C  -3.29745 -3.30943 -1.48445
C  -1.61238 -4.52206  0.60963
C  -3.85667 -2.76674  1.35715
Au -1.06974 -1.07875  0.09426
C   1.08538  0.17834 -0.06546
C  -0.08873  0.90428  0.16738
S  -2.21492  3.47905  2.53546
C   1.79484 -0.01404 -1.37383
H  -3.95765 -4.18671 -1.38827
H  -2.53104 -3.50852 -2.24890
H  -3.89055 -2.43556 -1.79439
H  -3.43800 -2.62101  2.36458
H  -4.49143 -3.66765  1.34817
H  -4.46325 -1.88684  1.09394
H  -2.33288 -5.35595  0.63068
H  -1.15775 -4.40348  1.60491
H  -0.81909 -4.74009 -0.12135
C  -2.09419  3.70335  0.75566
H  -1.29234  4.41941  2.87677
S   3.22344  1.23737 -1.07753
C   1.04665  0.51993 -2.60045
C   2.30294 -1.44294 -1.57836
H   2.94431 -1.50021 -2.47251
H   1.44063 -2.11861 -1.72977
H   2.87895 -1.80642 -0.71285
H   0.11201 -0.05242 -2.74565
H   1.66583  0.40210 -3.50299
H   0.78135  1.58536 -2.50230
C   4.40287  0.37818 -0.02006
H  -0.55681  1.48883 -0.63405
H  -0.32170  1.23527  1.18847
H   1.61810 -0.23553  0.80301
C  -1.96724  3.89511 -2.05793
C  -0.97962  4.49753 -1.25958
C  -1.03321  4.39881  0.14048
C  -3.09639  3.11101 -0.04225
C  -3.02526  3.20460 -1.44135
H  -1.92696  3.98533 -3.14760
H  -0.16189  5.05807 -1.72442
H  -0.25289  4.86801  0.74871
H  -3.94227  2.60131  0.43137
H  -3.81831  2.75762 -2.05015
C   6.34826 -0.82478  1.61291
C   5.35769 -0.01018  2.18670
C   4.38660  0.59602  1.37478
C   5.40858 -0.42729 -0.59733
C   6.37484 -1.02924  0.22321
H   7.10815 -1.29034  2.24815
H   5.34634  0.16355  3.26732
H   3.62871  1.25356  1.81214
H   5.43665 -0.56571 -1.68215
H   7.15593 -1.65017 -0.22648
```

SCF(BP86) = -942.329758986
H 0K = -941.906907
H 298K = -941.874851
G 298K = -941.981637
Solvent Correction(CHCl3) = -0.04793721
BP86-D3 Correction = -0.06889157
Lowest frequencies = 4.3216 cm-1, 6.8323 cm-1

$*IntIV_{4'}$ (anti)

40

P	-3.534725	0.807865	0.405949
C	-4.475792	-0.264810	1.586615
C	-3.237253	2.398222	1.309254
C	-4.733389	1.225847	-0.942668
Au	-1.587367	-0.198902	-0.422788
C	0.906204	-0.693604	-0.598948
C	0.000883	-1.312626	-1.474922
C	1.502105	-1.261582	0.650829
H	-5.389245	0.253244	1.921196
H	-3.843986	-0.495126	2.457905
H	-4.749990	-1.206242	1.086567
H	-4.260773	1.924211	-1.650035
H	-5.636073	1.690431	-0.513719
H	-5.013445	0.308151	-1.482051
H	-4.196640	2.821758	1.648611
H	-2.736935	3.112592	0.637956
H	-2.589882	2.211850	2.179487
S	3.233048	-1.624364	-0.111732
C	0.954808	-2.627379	1.077673
C	1.560376	-0.263449	1.807882
H	2.157563	-0.670710	2.639409
H	0.535858	-0.078046	2.179972
H	1.997908	0.700311	1.503858
H	-0.113376	-2.535648	1.347051
H	1.501076	-2.995304	1.959542
H	1.044637	-3.386572	0.283154
C	4.122227	-0.058994	-0.070446
H	-0.253966	-2.371069	-1.342980
H	-0.081643	-0.930751	-2.500808
H	1.282537	0.303407	-0.868770
C	5.659529	2.291597	-0.065423
C	4.962598	1.912658	-1.225147
C	4.196222	0.737172	-1.234090
C	4.836413	0.313178	1.089468
C	5.598774	1.491089	1.087630
H	6.261698	3.205385	-0.064444
H	5.023816	2.526709	-2.129061
H	3.673418	0.421927	-2.142518
H	4.804069	-0.326130	1.976531
H	6.155272	1.776824	1.985622

SCF(BP86) = -699.857546383
H 0K = -699.531619
H 298K = -699.508456
G 298K = -699.588660
Solvent Correction(CHCl3) = -0.04815896
BP86-D3 Correction = -0.04991336
Lowest frequencies = 6.2402 cm-1, 17.2375 cm-1

[(Me$_3$P)Au(SHPh)]$^+$

27

P	-2.704648	0.609773	0.162628
C	-2.972175	1.282798	1.865295
C	-2.942733	2.036522	-0.991416
Au	-0.638153	-0.426568	-0.076165
S	1.462838	-1.586863	-0.242029
H	1.495078	-1.775867	-1.594527
C	-4.115635	-0.543542	-0.157012
H	-4.066302	-1.386958	0.548318
H	-4.046963	-0.932207	-1.184390
H	-5.071185	-0.009613	-0.027812
H	-2.859665	1.685451	-2.031215
H	-2.165356	2.792976	-0.804943
H	-3.937446	2.483877	-0.832553
H	-2.193501	2.026564	2.092782
H	-2.908716	0.463208	2.597109
H	-3.965197	1.756996	1.927762
C	2.773154	-0.323710	-0.106827
C	4.829320	1.527636	0.214783
C	3.852007	1.730389	-0.775560
C	2.806882	0.808766	-0.939430
C	3.742743	-0.546642	0.884737
C	4.773157	0.396274	1.042808
H	5.638136	2.253870	0.337647
H	3.898577	2.610850	-1.423485
H	2.040135	0.968155	-1.703893
H	3.704264	-1.440049	1.515305
H	5.536174	0.233924	1.809715

SCF(BP86) = -504.527406283
H 0K = -504.316802
H 298K = -504.300275
G 298K = -504.365534
Solvent Correction(CH3Cl) = -0.051002
BP86-D3 Correction = -0.02773234
Lowest frequencies = 10.3816 cm-1, 15.8397 cm-1

3. Reactions of Substrate 24

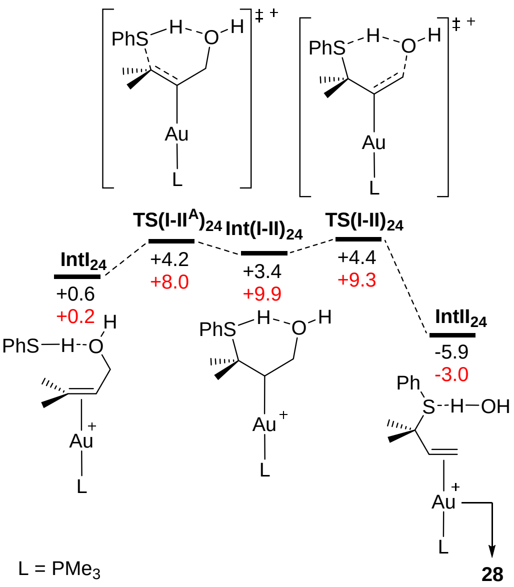

Figure S4. First S_N2' step to form **28** via *anti* attack of PhSH. Gas phase SCF energies (BP86) are shown in black with free energies at the BP86-D3($CHCl_3$) in red. All energies are in kcal/mol and are quoted relative to **5'** and the separated reactants set to zero.

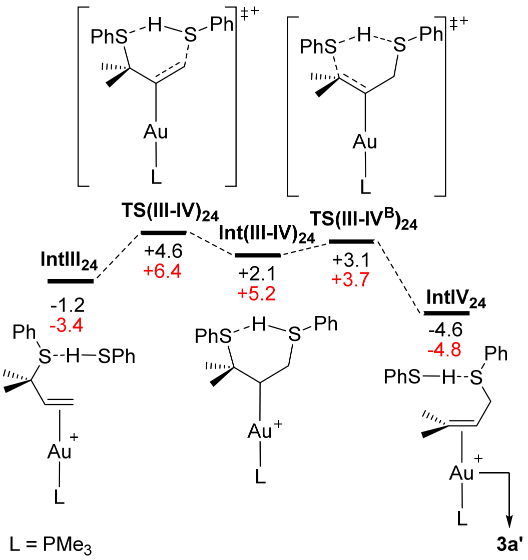

Figure S5. Second S_N2' step to form **3a'** via *anti* attack of PhSH. Gas phase SCF energies (BP86) are shown in black with free energies at the BP86-D3($CHCl_3$) in red. All energies are in kcal/mol and are quoted relative to **5'** and the separated reactants set to zero.

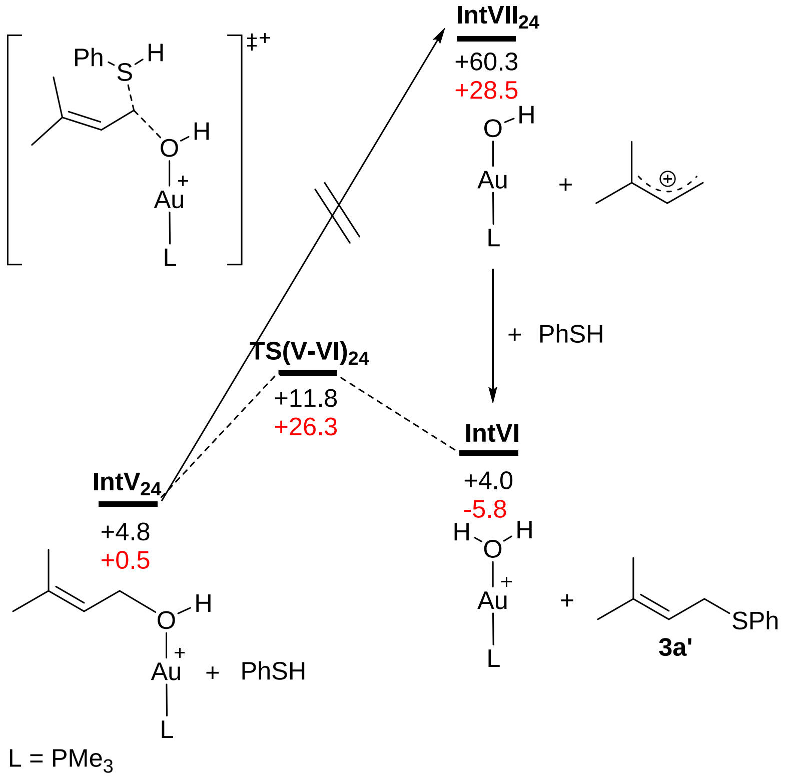

Figure S6. Alternative routes to the formation of **3a'** via either C–O bond cleavage and formation of a allylic cation or direct SN2 attack at a *O*-bound form of **24**. Gas phase SCF energies (BP86) are shown in black with free energies at the BP86-D3($CHCl_3$) in red. All energies are in kcal/mol and are quoted relative to **5'** and the separated reactants set to zero.

Table S2. Computed relative energies (kcal/mol) for the reactions **24** with PhSH. Energies are reported as ΔE (gas-phase SCF energies), ΔH_{298} (zero-point energy corrected energies at 298.15 K), ΔG (free energies at 298.15 K and 1 atm), ΔG_{disp} (including a correction for dispersion effects using Grimme's D3 parameter set) and $\Delta G_{CHCl3+disp}$ including an additional correction for $CHCl_3$ solvent via the PCM approach.

	**ΔE**	**ΔH_{298}**	**ΔG**	**ΔG_{disp}**	**$\Delta G_{CHCl3+disp}$**
**5'**	0.0	0.0	0.0	0.0	0.0
***$IntI_{24}$ (anti)**	+5.5	+5.2	+5.3	+0.6	-0.7
**$IntI_{24}$ (anti)**	+0.6	+2.2	+12.5	-0.5	+0.2
**$IntV_{24}$**	+4.8	+4.9	+4.9	+1.8	+0.5
**$IntVII_{24}$**	+60.3	+56.0	+44.6	+46.9	+28.5
**[1]$TS(V\text{-}VI)_{24}$**	+19.3	+19.5	+28.2	+22.0	+26.3
**[2]$TS(V\text{-}VI)_{24}$**	+11.8	+13.6	+31.5	+18.1	+26.3
**$IntVI_{24}$**	+4.0	+5.2	+4.7	+0.9	-5.8
**$TS(I\text{-}II^{A})_{24}$ (anti)**	+4.2	+5.3	+18.7	+3.7	+8.0
**$Int(I\text{-}II)_{24}$ (anti)**	+3.4	+4.9	+19.7	+4.4	+9.9
**$TS(I\text{-}II)_{24}$ (anti)**	+4.4	+5.7	+21.2	+5.8	+9.3
**$IntII_{24}$ (anti)**	-5.9	-3.5	+6.6	-6.3	-3.0
***$IntII_{24}$ (anti)**	+2.4	+2.8	+5.4	-4.4	-4.3
**$IntIII_{24}$ (anti)**	-1.2	+1.1	+10.4	-5.8	-3.4
**$TS(III\text{-}IV)_{24}$ (anti)**	+4.6	+5.8	+21.3	+1.2	+6.4
**$Int(III\text{-}IV)_{24}$ (anti)**	+2.1	+3.6	+19.6	-1.4	+5.2
**$TS(III\text{-}IV^{B})_{24}$ (anti)**	+3.1	+4.4	+19.6	-1.1	+3.7
**$IntIV_{24}$ (anti)**	-4.6	-2.2	+8.6	-6.5	-4.8
***$IntIV_{24}$ (anti)**	-1.6	-1.1	+0.7	-6.9	-8.0

*Complex computed in the absence of PhSH nucleophile

[1]Denotes the presence of one PhSH nucleophile in the calculations

[2]Denotes the presence of two PhSH nucleophiles in the calculations

24

16

```
C  -0.28617 -0.52100 -0.30302
C   0.95468 -0.04520 -0.04044
C  -1.57041  0.25049 -0.44988
O  -2.59701 -0.25016  0.42604
H  -2.20451 -0.25250  1.31983
C   1.30218  1.41799  0.12424
C   2.12973 -0.98954  0.09457
H  -0.41044 -1.60890 -0.40531
H  -1.41253  1.34016 -0.30715
H   2.91139 -0.76297 -0.65554
H   1.82999 -2.04226 -0.03197
H   2.61278 -0.88383  1.08490
H   0.42461  2.08166  0.10214
H   1.98994  1.74741 -0.67757
H   1.83757  1.58591  1.07771
H  -1.98270  0.12023 -1.46819
```

SCF(BP86) = -271.74708883
H 0K = -271.609674
H 298K = -271.601733
G 298K = -271.641506
Solvent Correction(CH3Cl) = -0.00331222
BP86-D3 Correction = -0.01074018
Lowest frequencies = 68.3286 cm-1, 140.9430 cm-1

***IntI$_{24}$ (anti)**

30

P	2.397271	-0.120167	0.105000
C	3.268823	1.428639	-0.414521
C	2.950736	-0.453221	1.839812
Au	0.069643	0.008993	-0.123911
C	-2.105691	-0.311215	-0.575839
C	-2.182981	0.936414	0.064925
C	-2.586292	-1.614186	0.048178
O	-4.011037	-1.551989	-0.014827
H	-4.359729	-2.459073	0.073384
C	-2.533939	1.093095	1.523300
C	-2.156595	2.212357	-0.744479
H	-2.118335	-0.292631	-1.676159
C	3.128611	-1.474793	-0.923545
H	-2.220944	-1.721283	1.092157
H	-2.175962	-2.463092	-0.533768
H	-3.184235	2.623696	-0.763077
H	-1.838274	2.050362	-1.785550
H	-1.520157	2.983733	-0.279487
H	-3.592180	1.410585	1.583346
H	-1.936056	1.889452	1.996525
H	-2.434611	0.164845	2.102728
H	4.050595	-0.515012	1.876207
H	2.517612	-1.403503	2.187271
H	2.892219	-1.299948	-1.984161
H	3.032033	1.647874	-1.466750
H	4.357661	1.300228	-0.301284
H	2.931214	2.268766	0.211166
H	2.606407	0.358551	2.498426
H	2.701800	-2.441714	-0.616537
H	4.222312	-1.496389	-0.788874

SCF(BP86) = -533.804016643
H 0K = -533.553574
H 298K = -533.535182
G 298K = -533.603079
Solvent Correction(CH3Cl) = -0.05218225
BP86-D3 Correction = -0.03300075
Lowest frequencies = 6.2137 cm-1, 36.7849 cm-1

IntI$_{24}$ (anti)

43

P	3.930913	0.385565	0.131871
C	4.673302	-0.410738	1.629477
C	4.921331	-0.233053	-1.305142
C	4.328284	2.188701	0.271634
Au	1.641307	-0.038928	-0.111251
C	-0.423857	-0.665639	-0.711500
C	-0.749731	0.051926	0.456123
S	-4.424821	-1.747551	1.004546
C	-0.484004	-2.179129	-0.847617
H	5.750398	-0.182510	1.680482
H	4.530752	-1.500682	1.574252
H	4.173787	-0.030655	2.533540
H	3.970056	2.713864	-0.626822
H	5.417643	2.325695	0.368591
H	3.825069	2.608727	1.155737
H	5.988771	-0.008056	-1.148103
H	4.574652	0.255828	-2.228276
H	4.786362	-1.320882	-1.404064
H	-3.530078	-2.244127	0.095375
O	-1.834073	-2.493315	-1.232837
H	-1.835094	-3.382867	-1.634913
C	-0.967424	-0.600546	1.793639
C	-1.118771	1.512269	0.367753
H	-0.558783	-0.128059	-1.662449
H	0.229963	-2.504214	-1.628563
H	-2.213274	1.590358	0.522081
H	-0.883857	1.953141	-0.612637
H	-0.641422	2.104500	1.166394
H	-2.060334	-0.782477	1.897288
H	-0.664209	0.061884	2.619980
H	-0.466000	-1.573050	1.902153
C	-4.576571	-0.122981	0.239711
C	-4.888580	2.449874	-0.873145
C	-5.198938	2.220849	0.478152
C	-5.040014	0.943382	1.038895
C	-4.274786	0.100672	-1.120209
C	-4.427062	1.385420	-1.665817
H	-5.016795	3.446165	-1.306999
H	-5.567730	3.039753	1.104342
H	-5.277655	0.773429	2.094348
H	-3.925539	-0.726219	-1.745782
H	-4.198507	1.548454	-2.724566
H	-0.204468	-2.684889	0.097592

SCF(BP86) = -776.278347859
H 0K = -775.929987
H 298K = -775.903601
G 298K = -775.992594
Solvent Correction(CH3Cl) = -0.05227167
BP86-D3 Correction = -0.05513402
Lowest frequencies = 5.4116 cm-1, 16.7055 cm-1

IntV$_{24}$

30

P	-2.245853	0.700910	0.008037
C	-2.742409	1.181946	1.722992
C	-2.056045	2.280042	-0.934776
Au	-0.372108	-0.595194	-0.031314
C	3.105374	-0.274599	-0.490438
C	3.369927	0.983129	-0.042700
C	2.693022	-1.440286	0.337160
O	1.318431	-1.955656	-0.022629
H	1.379977	-2.398960	-0.893609
C	3.275081	1.428361	1.397171
C	3.852014	2.042996	-1.004236
H	3.269135	-0.485059	-1.556853
C	-3.707700	-0.161155	-0.725274
H	2.611095	-1.221511	1.409582
H	3.352297	-2.315222	0.203518
H	4.280282	1.679147	1.782873
H	2.834634	0.682161	2.075318
H	2.679924	2.356088	1.472545
H	3.903627	1.677656	-2.041411
H	4.857170	2.400546	-0.714131
H	3.191569	2.929009	-0.975175
H	-1.931558	1.758409	2.193326
H	-3.657807	1.794938	1.686392
H	-2.929117	0.275253	2.318080
H	-2.993890	2.857116	-0.885641
H	-1.236483	2.871509	-0.499706
H	-1.817064	2.053787	-1.984878
H	-3.908776	-1.085012	-0.162140
H	-4.589214	0.499041	-0.679660
H	-3.494478	-0.419588	-1.773509

SCF(BP86) = -533.805135892
H 0K = -533.553730
H 298K = -533.535652
G 298K = -533.603627
Solvent Correction(CH3Cl) = -0.05229451
BP86-D3 Correction = -0.03049079
Lowest frequencies = 12.2397 cm-1, 15.6594 cm-1

IntVII$_{24}$

16

P	1.503761	-0.000061	-0.000004
C	2.302151	-0.838280	-1.456704
C	2.269239	1.694470	-0.003594
C	2.302211	-0.832060	1.460236
Au	-0.765869	-0.002113	0.000006
O	-2.764469	-0.108785	0.000076
H	-3.097160	0.810694	-0.000444
H	1.977592	-1.889851	-1.486154
H	3.401791	-0.791769	-1.388904
H	1.965591	-0.345201	-2.381494
H	1.977852	-1.883551	1.494086
H	1.965531	-0.335151	2.382926
H	3.401851	-0.785619	1.392266
H	1.929338	2.242769	0.888326
H	1.929048	2.239099	-0.897654
H	3.369989	1.631971	-0.003654

SCF(BP86) = -338.074767856
H 0K = -337.950612
H 298K = -337.939568
G 298K = -337.988751
Solvent Correction(CH3Cl) = -0.01518402
BP86-D3 Correction = -0.01355728
Lowest frequencies = 42.6019 cm-1, 91.7081 cm-1

Cationic Organic fragment of $IntVII_{24}$

14

C	0.851058	-0.696785	-0.013964
C	-0.410020	-0.013885	-0.009865
C	2.066693	-0.065162	0.011590
C	-0.557563	1.457139	0.000333
C	-1.642364	-0.834554	-0.003551
H	0.824315	-1.792322	-0.043729
H	2.174088	1.024062	0.039452
H	-1.002729	1.755043	-0.973797
H	0.363422	2.031507	0.157616
H	-1.321374	1.752785	0.745193
H	-1.529296	-1.774497	-0.569974
H	-2.539833	-0.280688	-0.316216
H	-1.810257	-1.149757	1.053312
H	2.994840	-0.646648	0.000881

SCF(BP86) = -195.641962192
H 0K = -195.521546
H 298K = -195.514690
G 298K = -195.551649
Solvent Correction(CH3Cl) = -0.06433655
BP86-D3 Correction = -0.00830891
Lowest frequencies = 78.1467 cm-1, 108.6905 cm-1

^{1}TS(V-VI)$_{24}$

43

P	-3.799690	-0.918414	0.432955
C	-3.582143	-1.816938	2.042044
C	-5.171082	0.287082	0.758010
Au	-1.905767	0.049607	-0.385517
C	2.157451	2.620610	-0.594288
C	1.926719	3.661421	0.269712
C	2.068377	1.208830	-0.277031
O	-0.207925	0.848481	-1.182939
H	-0.445654	1.741770	-1.502560
C	1.549723	3.492289	1.716885
C	2.027505	5.082749	-0.215961
H	2.440584	2.866456	-1.626008
C	-4.548371	-2.179367	-0.702967
H	1.568939	0.881463	0.636749
H	2.020637	0.487517	-1.093094
H	2.283004	4.007368	2.364219
H	1.478041	2.444740	2.042411
H	0.576556	3.978720	1.912750
H	2.301968	5.147645	-1.279798
H	2.775221	5.643870	0.375006
H	1.067341	5.611323	-0.069128
H	-3.217961	-1.112190	2.804992
H	-4.538453	-2.255197	2.371142
H	-2.836498	-2.616383	1.913731
H	-6.063393	-0.236867	1.137477
H	-4.835219	1.028419	1.498930
H	-5.421735	0.811506	-0.176666
H	-3.819141	-2.983993	-0.882774
H	-5.465236	-2.602682	-0.261298
H	-4.788123	-1.701599	-1.665102
S	4.150569	0.673999	0.398573
C	4.365277	-1.098040	0.156860
H	4.717101	1.097294	-0.765892
C	4.622188	-3.866963	-0.081519
C	4.591014	-3.275200	1.191566
C	4.464646	-1.883897	1.321536
C	4.385605	-1.679117	-1.125959
C	4.523424	-3.070059	-1.235301
H	4.729577	-4.951464	-0.176643
H	4.677468	-3.893658	2.089808
H	4.461778	-1.417025	2.311567
H	4.303300	-1.061598	-2.026173
H	4.553157	-3.530424	-2.227336

SCF(BP86) = -776.248466267
H 0K = -775.902639
H 298K = -775.876167
G 298K = -775.967703
Solvent Correction(CH3Cl) = -0.04657893
BP86-D3 Correction = -0.04413852
Lowest frequencies = -133.7005 cm-1, 7.4236 cm-1

^{2}**TS(V-VI)$_{24}$**

69

P	5.578518	0.469272	-0.679652
C	5.437732	1.972075	-1.754348
C	6.716188	0.964865	0.696199
Au	3.565067	-0.339440	0.021899
C	-0.352018	-0.846474	2.419998
C	-0.027444	-0.842264	3.757663
C	0.122969	0.080054	1.430226
O	1.716684	-1.102330	0.559405
H	1.852642	-1.772214	1.260494
C	0.875802	0.171814	4.404541
C	-0.609185	-1.880941	4.677320
H	-1.045468	-1.618875	2.064980
C	6.540572	-0.753144	-1.685478
H	0.827543	0.876107	1.667066
H	-0.244945	0.034790	0.406947
H	0.315290	0.736534	5.172699
H	1.312993	0.894865	3.701608
H	1.698674	-0.334668	4.941440
H	-1.263905	-2.591654	4.150814
H	-1.193539	-1.398767	5.483375
H	0.193687	-2.447645	5.184998
H	4.939233	2.777100	-1.193362
H	6.438321	2.307749	-2.072090
H	4.831624	1.729251	-2.640364
H	7.669196	1.337551	0.286765
H	6.239293	1.753970	1.297182
H	6.908366	0.094261	1.341544
H	5.950504	-1.040296	-2.569075
H	7.498739	-0.313199	-2.006613
H	6.731738	-1.652684	-1.080781
S	-1.807377	1.866323	1.679200
C	-1.703772	2.960792	0.253491
H	-2.662006	0.904104	1.135475
C	-1.477090	4.753007	-1.894678
C	-1.472314	5.233588	-0.575052
C	-1.587730	4.343217	0.503621
C	-1.694727	2.469065	-1.068278
C	-1.589929	3.373412	-2.136634
H	-1.395098	5.451642	-2.732894
H	-1.390957	6.307509	-0.379989
H	-1.608606	4.718193	1.531878
H	-1.753776	1.393273	-1.264608
H	-1.590596	2.992878	-3.163177
S	-0.191125	-1.237187	-2.184817
S	-4.076244	-0.859508	0.631102
H	0.631470	-1.480909	-1.108934
C	-1.421129	-2.496210	-1.824496
C	-5.624122	-0.030862	0.167214
H	-3.720780	-1.332781	-0.603546
C	-8.046427	1.249444	-0.433190
C	-6.842216	1.734758	-0.970083
C	-5.625459	1.105309	-0.664279
C	-6.826535	-0.522262	0.707698
C	-8.036264	0.125831	0.407680
H	-8.991253	1.748730	-0.669276
H	-6.845510	2.613815	-1.622539
H	-4.684792	1.496122	-1.064894
H	-6.811893	-1.408831	1.348688
H	-8.971965	-0.257052	0.827219
C	-3.408454	-4.452415	-1.369725
C	-2.249075	-4.420105	-0.577716
C	-1.256564	-3.451647	-0.799880
C	-2.589262	-2.522710	-2.618733
C	-3.571276	-3.501789	-2.391312
H	-4.174085	-5.214294	-1.197441
H	-2.105951	-5.161722	0.215204
H	-0.346457	-3.448047	-0.190964
H	-2.720964	-1.792625	-3.424700
H	-4.465979	-3.519319	-3.021990

SCF(BP86) = -1261.20309496

H 0K = -1260.662270
H 298K = -1260.619397
G 298K = -1260.754490
Solvent Correction(CH3Cl) = -0.04399081
BP86-D3 Correction = -0.08945173
Lowest frequencies = -191.5982 cm-1, 6.2344 cm-1

$IntVI_{24}$

```
17

P   1.56824 -0.00018  0.00384
C   2.25994  1.50957 -0.80440
C   2.27024 -0.05866  1.71099
Au -0.70889  0.00025 -0.00110
O  -2.90139 -0.00067 -0.07673
H  -3.32762 -0.78829  0.31684
C   2.25924 -1.45168 -0.90541
H   1.90955 -1.43311 -1.94867
H   1.91920 -2.38277 -0.42747
H   3.36050 -1.40654 -0.88367
H   1.92882 -0.97325  2.21883
H   1.92898  0.81918  2.28011
H   3.37118 -0.05683  1.65448
H   1.92150  2.40604 -0.26332
H   1.90910  1.56295 -1.84608
H   3.36118  1.46190 -0.78723
H  -3.32881  0.78359  0.32220


SCF(BP86) = -338.468872417
H 0K = -338.332280
H 298K = -338.320605
G 298K = -338.372269
Solvent Correction(CHCl3) = -0.06056610
BP86-D3 Correction = -0.01455309
Lowest frequencies = 9.8094 cm-1, 85.9645 cm-1
```

TS(I-II^{A})$_{24}$ (anti)

43

P	-3.716318	0.694155	-0.075301
C	-4.728902	-0.213478	-1.336018
C	-4.659440	0.521424	1.511073
C	-3.868102	2.484285	-0.532696
Au	-1.501925	-0.043162	0.091021
C	0.566074	-0.679132	0.441391
C	0.962913	-1.102395	-0.881471
S	3.527912	-1.527089	-0.592725
C	0.526588	-1.662375	1.608130
H	-5.757952	0.181127	-1.352524
H	-4.750390	-1.284894	-1.084870
H	-4.272797	-0.090199	-2.330119
H	-3.351101	3.098286	0.220444
H	-4.929747	2.776576	-0.580627
H	-3.395768	2.652988	-1.512458
H	-5.690951	0.887634	1.382081
H	-4.158685	1.104111	2.299261
H	-4.680332	-0.537468	1.810779
H	3.097339	-1.918652	0.659033
O	1.887461	-1.947253	2.030214
H	1.860852	-2.593332	2.761742
C	0.688445	-2.507311	-1.374134
C	1.151806	-0.048887	-1.953750
H	1.013921	0.289590	0.728806
H	-0.027289	-1.209940	2.451761
H	1.800322	-0.414291	-2.764608
H	1.571699	0.883363	-1.543909
H	0.171356	0.192046	-2.407145
H	1.300445	-2.747547	-2.256299
H	-0.372109	-2.575298	-1.685211
H	0.854492	-3.275814	-0.604026
C	4.114558	0.105905	-0.141238
C	5.048726	2.685901	0.445611
C	5.214576	2.154842	-0.844748
C	4.751676	0.865935	-1.145391
C	3.943053	0.631806	1.157321
C	4.416818	1.922283	1.440849
H	5.420540	3.688459	0.677138
H	5.715842	2.741050	-1.621083
H	4.898069	0.447038	-2.146423
H	3.457263	0.031636	1.932530
H	4.296494	2.327402	2.450663
H	0.006329	-2.597757	1.326265

SCF(BP86) = -776.272652256
H 0K = -775.923762
H 298K = -775.898798
G 298K = -775.982831
Solvent Correction(CH3Cl) = -0.04655497
BP86-D3 Correction = -0.05811568
Lowest frequencies = -59.1187 cm-1, 6.2128 cm-1

Int(I-II)$_{24}$ (anti)

43

P	-3.717196	0.675698	-0.069349
C	-4.781974	-0.421285	-1.122722
C	-4.616466	0.773365	1.551533
C	-3.918046	2.365878	-0.810608
Au	-1.483567	-0.031193	0.133188
C	0.560154	-0.637407	0.416613
C	1.167236	-1.004747	-0.916063
S	3.193040	-1.447410	-0.635704
C	0.555103	-1.698419	1.508438
H	-5.813012	-0.033946	-1.163861
H	-4.788491	-1.437569	-0.699775
H	-4.366291	-0.465164	-2.141106
H	-3.379688	3.100555	-0.192411
H	-4.984305	2.639987	-0.862620
H	-3.487556	2.374329	-1.823711
H	-5.653822	1.112098	1.397084
H	-4.094617	1.479585	2.215377
H	-4.621600	-0.219711	2.026416
H	2.823767	-1.916511	0.677272
O	1.948742	-1.999030	1.941045
H	1.957863	-2.835484	2.448223
C	0.709415	-2.330329	-1.535135
C	1.202943	0.138890	-1.928853
H	1.035319	0.286661	0.798953
H	0.017518	-1.331879	2.398426
H	1.796764	-0.125484	-2.819962
H	1.610525	1.062627	-1.487544
H	0.171352	0.351036	-2.260402
H	1.229157	-2.535068	-2.484740
H	-0.372704	-2.257446	-1.744607
H	0.856781	-3.195768	-0.867536
C	3.983397	0.095082	-0.150815
C	5.273254	2.490374	0.492884
C	5.312657	2.007593	-0.825966
C	4.671477	0.804886	-1.156240
C	3.940922	0.567020	1.177195
C	4.593207	1.769938	1.488914
H	5.782433	3.424883	0.746891
H	5.851881	2.561550	-1.600159
H	4.714678	0.416895	-2.178787
H	3.420879	-0.003241	1.951843
H	4.573410	2.139914	2.518592
H	0.081215	-2.638306	1.174366

SCF(BP86) = -776.273864487
H 0K = -775.923994
H 298K = -775.899299
G 298K = -775.981250
Solvent Correction(CH3Cl) = -0.04467395
BP86-D3 Correction = -0.05863422
Lowest frequencies = 9.6369 cm-1, 19.7427 cm-1

$TS(I\text{-}II)_{24}$ (anti)

43

P	3.695474	0.697884	0.049061
C	4.618561	-0.041516	1.478814
C	4.757455	0.340388	-1.429837
C	3.837367	2.531223	0.296138
Au	1.478814	-0.055092	-0.167182
C	-0.591449	-0.652987	-0.368852
C	-1.251030	-0.942855	0.986114
S	-3.142026	-1.404027	0.699749
C	-0.457795	-1.755743	-1.331249
H	5.645055	0.357587	1.520378
H	4.654827	-1.135660	1.363814
H	4.093981	0.198823	2.416256
H	3.381027	3.050876	-0.560251
H	4.895611	2.826265	0.384206
H	3.298647	2.819420	1.211818
H	5.778440	0.723475	-1.269985
H	4.319958	0.821395	-2.318030
H	4.795709	-0.746749	-1.598131
H	-2.599498	-2.084455	-0.937915
O	-2.011318	-2.293669	-1.821108
H	-2.011893	-3.266519	-1.957047
C	-0.719431	-2.200587	1.694706
C	-1.212363	0.275555	1.915074
H	-1.046749	0.238330	-0.838148
H	-0.068251	-1.479175	-2.316886
H	-1.774709	0.085200	2.843824
H	-1.630676	1.171772	1.429055
H	-0.163408	0.494330	2.185240
H	-1.215033	-2.343084	2.668119
H	0.364944	-2.084552	1.870600
H	-0.866890	-3.124995	1.109261
C	-3.929590	0.128403	0.159330
C	-5.251423	2.484810	-0.604861
C	-5.211866	2.110217	0.748321
C	-4.555902	0.931868	1.135354
C	-3.978624	0.496088	-1.201954
C	-4.638817	1.676841	-1.576804
H	-5.770339	3.400672	-0.903744
H	-5.700331	2.730587	1.506016
H	-4.535714	0.627519	2.186113
H	-3.522304	-0.140254	-1.965811
H	-4.682024	1.958683	-2.633480
H	-0.026380	-2.691892	-0.952687

SCF(BP86) = -776.272353426
H 0K = -775.922103
H 298K = -775.898032
G 298K = -775.978815
Solvent Correction(CH3Cl) = -0.04788165
BP86-D3 Correction = -0.05879518
Lowest frequencies = -178.4263 cm-1, 8.4192 cm-1

IntII$_{24}$ (anti)

43

P	3.601087	0.461874	0.838389
C	4.034874	-0.793258	2.130702
C	5.103544	0.590390	-0.238105
C	3.504223	2.079516	1.737100
Au	1.673990	-0.055436	-0.389553
C	-0.799311	-0.417689	-0.779557
C	-1.414217	-1.487735	0.068975
S	-3.138770	-1.429681	-0.789677
C	0.103128	-0.578735	-1.842369
H	4.961857	-0.497864	2.648634
H	4.177751	-1.774807	1.653615
H	3.214474	-0.867480	2.860596
H	3.324686	2.888843	1.013108
H	4.446612	2.269768	2.276132
H	2.669410	2.051529	2.453797
H	5.986466	0.838793	0.372943
H	4.946616	1.374865	-0.994041
H	5.269678	-0.369153	-0.751170
H	-2.037534	1.752936	-3.369478
O	-1.529442	2.114208	-2.620032
H	-1.577949	3.079006	-2.749287
C	-0.877043	-2.903121	-0.163936
C	-1.490279	-1.123563	1.552260
H	-1.166971	0.600266	-0.593684
H	0.173399	0.243974	-2.565646
H	-2.124467	-1.841901	2.096149
H	-1.896692	-0.112407	1.709809
H	-0.474624	-1.162328	1.987117
H	-1.433627	-3.626404	0.451366
H	0.189248	-2.951837	0.124172
H	-0.961787	-3.219164	-1.216838
C	-4.065600	-0.161891	0.095048
C	-5.657617	1.744123	1.404443
C	-5.720194	0.395439	1.791415
C	-4.924609	-0.560726	1.141891
C	-4.009851	1.190955	-0.305398
C	-4.803033	2.138462	0.361088
H	-6.280624	2.487508	1.911494
H	-6.391209	0.084033	2.597995
H	-4.973491	-1.614683	1.431625
H	-3.349180	1.497874	-1.124788
H	-4.761673	3.189082	0.055781
H	0.359903	-1.580885	-2.206757

SCF(BP86) = -776.288770059
H 0K = -775.939895
H 298K = -775.912779
G 298K = -776.002068
Solvent Correction(CH3Cl) = -0.04810439
BP86-D3 Correction = -0.05487416
Lowest frequencies = 12.7900 cm-1, 16.0278 cm-1

***IntII$_{24}$ (anti)**

40

P	3.531053	0.811305	0.406067
C	4.160702	0.054699	1.975242
C	4.937936	0.698155	-0.793266
C	3.337010	2.617903	0.767961
Au	1.586954	-0.201199	-0.423556
C	-0.903745	-0.696104	-0.599698
C	-1.499057	-1.257875	0.653286
S	-3.230727	-1.624949	-0.105481
C	0.000516	-1.319847	-1.473462
H	5.088050	0.559515	2.291445
H	4.362833	-1.014629	1.810457
H	3.400221	0.156720	2.764278
H	3.033698	3.144031	-0.149954
H	4.290964	3.032967	1.132095
H	2.558878	2.758347	1.533464
H	5.832728	1.183549	-0.370878
H	4.658778	1.196401	-1.734158
H	5.157926	-0.359849	-1.002412
C	-0.951013	-2.621301	1.086759
C	-1.556446	-0.253622	1.805116
H	-1.280336	0.299510	-0.874314
H	0.083051	-0.942599	-2.501023
H	-2.152100	-0.656881	2.639684
H	-1.995293	0.708095	1.496542
H	-0.531558	-0.065124	2.174686
H	-1.496516	-2.984862	1.970907
H	0.117373	-2.527912	1.354833
H	-1.041256	-3.384591	0.296220
C	-4.120805	-0.059969	-0.069125
C	-5.659369	2.289816	-0.070740
C	-5.597675	1.492946	1.084777
C	-4.834638	0.315486	1.089953
C	-4.195787	0.732495	-1.235225
C	-4.962753	1.907622	-1.229586
H	-6.262020	3.203286	-0.072345
H	-6.153920	1.781180	1.982128
H	-4.801468	-0.321005	1.979011
H	-3.673303	0.414652	-2.142932
H	-5.024709	2.518825	-2.135379
H	0.255183	-2.377701	-1.336677

SCF(BP86) = -699.857560117
H 0K = -699.531650
H 298K = -699.508482
G 298K = -699.588500
Solvent Correction(CH3Cl) = -0.04827478
BP86-D3 Correction = -0.04993104
Lowest frequencies = 8.0078 cm-1, 17.0987 cm-1

$IntIII_{24}$ (anti)

53

```
P  -2.47560 -2.95185  0.13673
C  -3.29745 -3.30943 -1.48445
C  -1.61238 -4.52206  0.60963
C  -3.85667 -2.76674  1.35715
Au -1.06974 -1.07875  0.09426
C   1.08538  0.17834 -0.06546
C  -0.08873  0.90428  0.16738
S  -2.21492  3.47905  2.53546
C   1.79484 -0.01404 -1.37383
H  -3.95765 -4.18671 -1.38827
H  -2.53104 -3.50852 -2.24890
H  -3.89055 -2.43556 -1.79439
H  -3.43800 -2.62101  2.36458
H  -4.49143 -3.66765  1.34817
H  -4.46325 -1.88684  1.09394
H  -2.33288 -5.35595  0.63068
H  -1.15775 -4.40348  1.60491
H  -0.81909 -4.74009 -0.12135
C  -2.09419  3.70335  0.75566
H  -1.29234  4.41941  2.87677
S   3.22344  1.23737 -1.07753
C   1.04665  0.51993 -2.60045
C   2.30294 -1.44294 -1.57836
H   2.94431 -1.50021 -2.47251
H   1.44063 -2.11861 -1.72977
H   2.87895 -1.80642 -0.71285
H   0.11201 -0.05242 -2.74565
H   1.66583  0.40210 -3.50299
H   0.78135  1.58536 -2.50230
C   4.40287  0.37818 -0.02006
H  -0.55681  1.48883 -0.63405
H  -0.32170  1.23527  1.18847
H   1.61810 -0.23553  0.80301
C  -1.96724  3.89511 -2.05793
C  -0.97962  4.49753 -1.25958
C  -1.03321  4.39881  0.14048
C  -3.09639  3.11101 -0.04225
C  -3.02526  3.20460 -1.44135
H  -1.92696  3.98533 -3.14760
H  -0.16189  5.05807 -1.72442
H  -0.25289  4.86801  0.74871
H  -3.94227  2.60131  0.43137
H  -3.81831  2.75762 -2.05015
C   6.34826 -0.82478  1.61291
C   5.35769 -0.01018  2.18670
C   4.38660  0.59602  1.37478
C   5.40858 -0.42729 -0.59733
C   6.37484 -1.02924  0.22321
H   7.10815 -1.29034  2.24815
H   5.34634  0.16355  3.26732
H   3.62871  1.25356  1.81214
H   5.43665 -0.56571 -1.68215
H   7.15593 -1.65017 -0.22648
```

SCF(BP86) = -942.329758986
H 0K = -941.906907
H 298K = -941.874851
G 298K = -941.981637
Solvent Correction(CHCl3) = -0.04793721
BP86-D3 Correction = -0.06889157
Lowest frequencies = 4.3216 cm-1, 6.8323 cm-1

TS(III-IV)$_{24}$ (anti)

53

P	-4.289409	-0.645725	-0.031448
C	-4.748839	-2.070986	1.062798
C	-5.289773	0.781612	0.601624
C	-5.032422	-1.051300	-1.681667
Au	-1.986666	-0.204095	-0.116918
C	0.140709	0.358120	-0.186299
C	0.487724	-0.860245	-0.877301
S	2.759818	-0.943864	-1.531867
C	0.680557	0.690922	1.206889
H	-5.841158	-2.218219	1.059681
H	-4.408766	-1.867609	2.089744
H	-4.253735	-2.984581	0.699817
H	-4.868012	-0.210113	-2.372275
H	-6.113975	-1.236548	-1.577983
H	-4.543484	-1.947909	-2.092206
H	-6.360822	0.522019	0.613057
H	-5.130408	1.655818	-0.047983
H	-4.962623	1.034036	1.621856
C	3.426457	-2.353983	-0.633161
H	2.911315	0.038560	-0.500524
S	2.497833	1.352059	1.042937
C	0.846265	-0.533415	2.122934
C	-0.149972	1.786308	1.889295
H	0.306527	2.086026	2.846854
H	-1.166871	1.402824	2.095951
H	-0.247661	2.680958	1.253044
H	-0.133091	-1.030028	2.249079
H	1.205330	-0.227106	3.118457
H	1.555938	-1.276409	1.721460
C	2.345531	2.939382	0.197107
H	0.580778	-1.797913	-0.318957
H	0.144055	-0.975095	-1.909711
H	0.099017	1.250491	-0.834313
C	4.477132	-4.605312	0.666083
C	4.767304	-3.317111	1.145009
C	4.247557	-2.183911	0.500547
C	3.135226	-3.642816	-1.124061
C	3.664483	-4.764735	-0.468318
H	4.890402	-5.483162	1.171497
H	5.409412	-3.187329	2.021736
H	4.492850	-1.181962	0.865297
H	2.511200	-3.768286	-2.015100
H	3.443662	-5.765721	-0.851572
C	2.219547	5.454534	-1.054027
C	2.245889	4.284586	-1.830863
C	2.313782	3.026829	-1.211151
C	2.330765	4.115832	0.976088
C	2.266111	5.368827	0.347116
H	2.174324	6.433307	-1.541361
H	2.226308	4.348842	-2.923364
H	2.361735	2.122022	-1.824555
H	2.379936	4.043560	2.066747
H	2.258918	6.278986	0.954881

SCF(BP86) = -942.320597146
H 0K = -941.897246
H 298K = -941.867302
G 298K = -941.964176
Solvent Correction(CHCl3) = -0.04361495
BP86-D3 Correction = -0.07516386
Lowest frequencies = -134.7252 cm-1, 8.1177 cm-1

Int(III-IV)$_{24}$ (anti)

53

P	-4.354402	-0.457534	-0.045515
C	-4.870386	-2.240980	-0.052479
C	-5.218403	0.265453	1.430459
C	-5.234757	0.282856	-1.502564
Au	-2.027570	-0.121476	-0.074629
C	0.101863	0.201119	-0.207045
C	0.617129	-1.014182	-0.965553
S	2.470233	-0.958180	-1.541011
C	0.657596	0.498042	1.168671
H	-5.969334	-2.324915	-0.045766
H	-4.458351	-2.743327	0.836132
H	-4.470411	-2.732101	-0.952804
H	-5.051534	1.368054	-1.525610
H	-6.318556	0.093779	-1.436104
H	-4.840620	-0.160522	-2.429840
H	-6.302747	0.076389	1.373561
H	-5.036821	1.350703	1.463440
H	-4.815106	-0.188773	2.348544
C	3.240019	-2.343759	-0.668821
H	2.768915	0.245056	-0.206553
S	2.606797	1.127989	1.015311
C	0.838891	-0.714711	2.090861
C	-0.026933	1.670376	1.875639
H	0.523138	1.968995	2.784415
H	-1.042155	1.360902	2.180017
H	-0.125306	2.546405	1.214453
H	-0.151561	-1.177055	2.252103
H	1.241315	-0.416814	3.072486
H	1.502246	-1.485587	1.664832
C	2.553708	2.729000	0.194389
H	0.550144	-1.952066	-0.394474
H	0.075599	-1.140906	-1.913226
H	0.153482	1.111910	-0.834113
C	4.522965	-4.493475	0.598959
C	5.076581	-3.205449	0.669175
C	4.437805	-2.123691	0.040910
C	2.692408	-3.638781	-0.763274
C	3.332953	-4.706980	-0.116631
H	5.023220	-5.332240	1.092069
H	6.008815	-3.034924	1.216465
H	4.877098	-1.122480	0.093879
H	1.785842	-3.816807	-1.350340
H	2.907998	-5.712896	-0.190118
C	2.530913	5.277549	-0.968700
C	2.667555	5.144046	0.423128
C	2.682785	3.871150	1.011809
C	2.424547	2.854651	-1.204359
C	2.414111	4.135606	-1.777997
H	2.525923	6.272322	-1.424228
H	2.770856	6.031526	1.054688
H	2.804078	3.762610	2.094089
H	2.355270	1.970947	-1.844140
H	2.321992	4.237403	-2.863664

SCF(BP86) = -942.324574194
H 0K = -941.900928
H 298K = -941.870780
G 298K = -941.966986
Solvent Correction(CHCl3) = -0.04143085
BP86-D3 Correction = -0.07643137
Lowest frequencies = 9.6317 cm-1, 18.3463 cm-1

TS(III-IV^B)$_{24}$ (anti)

53

P	-4.372496	-0.404963	-0.057398
C	-4.906877	-2.180546	-0.046621
C	-5.246250	0.365093	1.386117
C	-5.184714	0.329486	-1.553505
Au	-2.050923	-0.115748	-0.038941
C	0.123505	0.143556	-0.218214
C	0.580734	-1.069794	-1.025388
S	2.380324	-1.049261	-1.645740
C	0.518887	0.348160	1.161910
H	-6.006786	-2.247884	-0.069060
H	-4.527048	-2.671240	0.862474
H	-4.489509	-2.692647	-0.927007
H	-4.977407	1.409935	-1.588029
H	-6.273788	0.164117	-1.516431
H	-4.772221	-0.139239	-2.459980
H	-6.332886	0.198307	1.306590
H	-5.041099	1.446378	1.402613
H	-4.873856	-0.082043	2.320435
C	3.168985	-2.381480	-0.709831
H	2.910180	0.363472	-0.177359
S	2.900377	1.155302	0.995262
C	0.840806	-0.835521	2.052313
C	0.022008	1.591330	1.876038
H	0.672628	1.849661	2.726566
H	-0.986604	1.398493	2.287463
H	-0.048354	2.457432	1.199109
H	-0.106635	-1.336013	2.332234
H	1.327816	-0.513441	2.985153
H	1.477907	-1.585617	1.558890
C	2.717071	2.751997	0.201765
H	0.471416	-2.014931	-0.473340
H	-0.008157	-1.149376	-1.950711
H	0.136250	1.082770	-0.801215
C	4.509782	-4.449953	0.643352
C	5.089899	-3.175027	0.556848
C	4.422011	-2.135110	-0.111186
C	2.598239	-3.669717	-0.651121
C	3.264638	-4.693532	0.039251
H	5.030812	-5.255559	1.169236
H	6.064558	-2.980278	1.015055
H	4.879972	-1.142888	-0.174127
H	1.652636	-3.880807	-1.160979
H	2.818229	-5.692049	0.082988
C	2.424708	5.304363	-0.932984
C	2.574497	5.165668	0.457247
C	2.721695	3.894644	1.030716
C	2.571764	2.885331	-1.195890
C	2.427717	4.164925	-1.754128
H	2.317896	6.298935	-1.376099
H	2.586382	6.050824	1.100662
H	2.855640	3.789913	2.112355
H	2.599160	2.003957	-1.843460
H	2.327399	4.268468	-2.839170

SCF(BP86) = -942.323020106
H 0K = -941.899615
H 298K = -941.869452
G 298K = -941.966890
Solvent Correction(CHCl3) = -0.04410199
BP86-D3 Correction = -0.07614278
Lowest frequencies = -84.1086 cm-1, 8.0984 cm-1

IntIV$_{24}$ (anti)

53

P	4.490712	0.178740	0.093389
C	5.026624	0.788416	1.757543
C	5.467477	-1.364632	-0.212322
C	5.130910	1.420263	-1.122370
Au	2.180732	-0.139717	-0.081018
C	-0.029874	-0.012470	-0.508187
C	-0.555575	1.315101	-0.014514
S	-2.396844	1.354517	-0.405641
C	0.018706	-1.204775	0.241844
H	6.120038	0.926292	1.773123
H	4.737145	0.056640	2.526990
H	4.531800	1.747559	1.973296
H	4.904641	1.082671	-2.145210
H	6.220942	1.532698	-1.004238
H	4.640438	2.390311	-0.949767
H	6.545168	-1.148542	-0.130101
H	5.244510	-1.745971	-1.220383
H	5.188245	-2.128855	0.528806
C	-2.738127	3.121491	-0.251058
H	-3.937492	-0.453626	1.172138
S	-4.083014	-1.629330	1.852439
C	-0.270355	-1.261072	1.719524
C	0.117609	-2.538123	-0.462059
H	-0.872468	-3.031579	-0.399472
H	0.836966	-3.211031	0.034091
H	0.382083	-2.438384	-1.525971
H	0.345017	-2.028010	2.217650
H	-1.332405	-1.559834	1.853835
H	-0.133624	-0.298566	2.233849
C	-3.743914	-2.710851	0.452942
H	-0.407415	1.469541	1.064773
H	-0.101763	2.151795	-0.567443
H	-0.049711	-0.131577	-1.602634
C	-3.358508	5.849919	-0.038731
C	-3.308367	5.050558	1.115780
C	-3.003358	3.684592	1.014442
C	-2.792091	3.921111	-1.411182
C	-3.103737	5.285286	-1.299464
H	-3.604561	6.913161	0.043899
H	-3.518063	5.487863	2.097066
H	-2.982149	3.051976	1.907414
H	-2.601355	3.469532	-2.389642
H	-3.152719	5.905453	-2.200071
C	-3.224998	-4.506186	-1.660294
C	-3.113463	-4.932474	-0.325762
C	-3.362706	-4.040997	0.729962
C	-3.872459	-2.284464	-0.885496
C	-3.604824	-3.181098	-1.932394
H	-3.031452	-5.204285	-2.480309
H	-2.830569	-5.965851	-0.099969
H	-3.267177	-4.378818	1.767471
H	-4.195615	-1.263053	-1.109731
H	-3.713099	-2.841501	-2.967849

SCF(BP86) = -942.335190669
H 0K = -941.912104
H 298K = -941.880100
G 298K = -941.984512
Solvent Correction(CHCl3) = -0.04929878
BP86-D3 Correction = -0.06699874
Lowest frequencies = 11.0747 cm-1, 12.3887 cm-1

*IntIV$_{24}$ (anti)

```
40

P   3.27725 -1.13717 -0.06390
C   4.83835 -0.15424  0.10148
C   3.36543 -2.43969  1.24879
C   3.42496 -2.03788 -1.67496
Au  1.34575  0.18055  0.08479
C  -0.82149  0.91213  0.06022
C  -1.45535  0.63916 -1.27909
S  -3.22402  1.25576 -1.22530
C  -0.07662  2.05114  0.41760
H   5.71088 -0.82356  0.02678
H   4.84967  0.35660  1.07625
H   4.88418  0.59998 -0.69874
H   2.55776 -2.70300 -1.80514
H   4.35125 -2.63507 -1.68698
H   3.44528 -1.31114 -2.50128
H   4.29136 -3.02647  1.13437
H   2.49431 -3.10691  1.16285
H   3.35248 -1.96241  2.24046
C  -4.04351 -0.11123 -0.37405
C   0.30772  3.12768 -0.57340
C   0.06745  2.43235  1.87762
H  -0.62784  3.26839  2.08654
H   1.08091  2.79839  2.11143
H  -0.18552  1.60519  2.55894
H   1.27460  3.58673 -0.31015
H  -0.45251  3.93139 -0.52401
H   0.35374  2.77736 -1.61481
H  -0.97857  1.17921 -2.11082
H  -1.47983 -0.43384 -1.52093
H  -1.24932  0.32897  0.88987
C  -5.42052 -2.18843  0.92159
C  -4.98082 -2.35627 -0.40297
C  -4.30251 -1.31762 -1.05809
C  -4.48825  0.06023  0.95256
C  -5.18000 -0.97992  1.59485
H  -5.96101 -2.99643  1.42434
H  -5.18456 -3.29078 -0.93537
H  -3.98989 -1.43000 -2.10139
H  -4.30307  1.00788  1.46785
H  -5.53218 -0.84292  2.62217


SCF(BP86) = -699.864008888
H 0K = -699.538119
H 298K = -699.514645
G 298K = -699.595954
Solvent Correction(CHCl3) = -0.05030723
BP86-D3 Correction = -0.04641935
Lowest frequencies = 12.8400 cm-1, 15.6727 cm-1
```

4. Reactions of Substrate **4'** with PhSH at [(Johnphos)Au(NCMe)]$^{+}$, **5**.

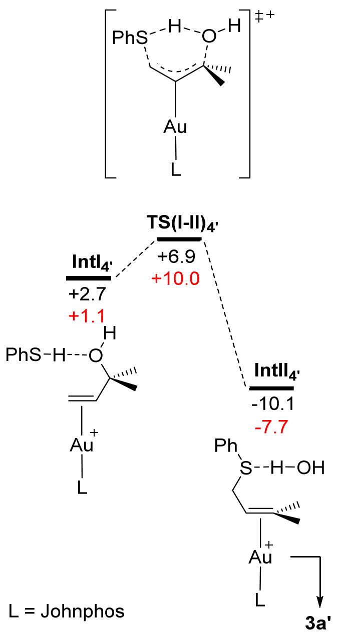

Figure S7. First S_N2' step to form **3a'** via *anti* attack of PhSH using the full experimental catalyst. Gas phase SCF energies (BP86) are shown in black with free energies at the BP86-D3($CHCl_3$) in red. All energies are in kcal/mol and are quoted relative to **5** and the separated reactants set to zero.

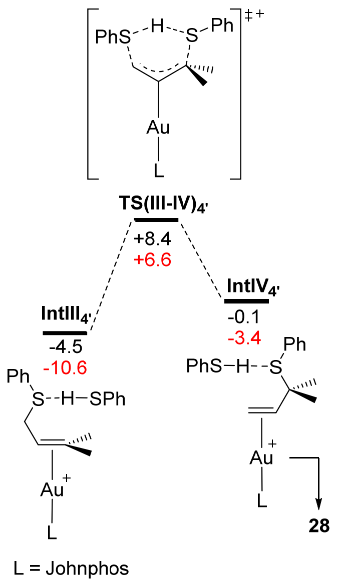

Figure S8. Second S_N2' step to form **28** via *anti* attack of PhSH using the full experimental catalyst. Gas phase SCF energies (BP86) are shown in black with free energies at the BP86-D3($CHCl_3$) in red. All energies are in kcal/mol and are quoted relative to **5** and the separated reactants set to zero.

Table S3. Computed relative energies (kcal/mol) for the reactions **4'** with PhSH at the full experimental catalyst, [(Johnphos)Au(NCMe)]$^+$, **5**. Energies are reported as ΔE (gas-phase SCF energies), ΔH_{298} (zero-point energy corrected energies at 298.15 K), ΔG (free energies at 298.15 K and 1 atm), ΔG_{disp} (including a correction for dispersion effects using Grimme's D3 parameter set) and $\Delta G_{CHCl3+disp}$ including an additional correction for $CHCl_3$ solvent via the PCM approach.

	**ΔE**	**ΔH_{298}**	**ΔG**	**ΔG_{disp}**	**$\Delta G_{CHCl3+disp}$**
**5**	0.0	0.0	0.0	0.0	0.0
***IntI$_{4'}$ (anti)**	+5.8	+5.8	+8.4	+1.1	-1.2
**IntI$_{4'}$ (anti)**	+2.7	+4.5	+15.5	+1.3	+1.1
**TS(I-II)$_{4'}$ (anti)**	+6.9	+8.0	+23.0	+7.5	+10.0
**IntII$_{4'}$ (anti)**	-10.1	-6.9	+4.8	-9.6	-7.7
***IntII$_{4'}$ (ant)**	-0.7	+0.5	+2.7	-6.8	-8.7
**IntIII$_{4'}$ (anti)**	-4.5	-1.5	+10.3	-11.0	-10.6
**TS(III-IV)$_{4'}$ (anti)**	+8.4	+9.8	+26.9	+3.6	+6.6
**IntIV$_{4'}$ (anti)**	-0.1	+2.7	+12.9	-4.2	-3.4
***IntIV$_{4'}$ (anti)**	+2.9	+3.9	+8.0	+3.8	-4.9
**[(Johnphos)Au(SHPh)]$^+$**	-0.1	0.0	+2.8	-3.4	-3.4

*Complex computed in the absence of PhSH nucleophile

5

```
55

P  -1.27163 -0.65909  0.03615
C  -1.89274 -1.56822 -1.56789
C  -1.82140 -1.46815  1.70650
Au  1.04556 -0.69156 -0.03819
N   3.09801 -0.93359 -0.13331
C  -1.37656 -2.94693  1.70152
C  -3.33605 -1.37026  1.98470
C  -1.06180 -0.69998  2.81282
C  -3.36372 -2.02985 -1.51025
C  -0.98853 -2.80063 -1.80725
C  -1.69159 -0.56455 -2.72614
C   4.25692 -1.07043 -0.18607
C  -1.96717  1.06891 -0.00569
C  -3.20804  3.60818 -0.24587
C  -3.99639  2.45050 -0.19420
C  -3.37655  1.20013 -0.07920
C  -1.16474  2.24673 -0.04235
C  -1.81463  3.49739 -0.17019
H  -3.67323  4.59416 -0.34029
H  -5.08740  2.51365 -0.24740
H  -4.00807  0.31014 -0.05144
C   0.33218  2.31334  0.05557
H  -1.19548  4.39968 -0.19730
C   3.11010  2.81669  0.28076
C   2.47681  2.90086 -0.97042
C   1.09750  2.65889 -1.08204
C   0.98256  2.22582  1.30923
C   2.36066  2.47649  1.41951
H   4.17804  3.03988  0.37245
H   3.04981  3.18179 -1.86008
H   0.60003  2.75900 -2.05240
H   0.39531  2.00203  2.20524
H   2.84099  2.43081  2.40236
H  -0.64931 -0.20337 -2.77899
H  -2.35920  0.30751 -2.64611
H  -1.91409 -1.07766 -3.67870
H  -4.07285 -1.19828 -1.37990
H  -3.53936 -2.77782 -0.72056
H  -3.61219 -2.51186 -2.47289
H  -1.29644 -3.27964 -2.75418
H  -1.07427 -3.55611 -1.01123
H   0.07405 -2.51799 -1.90113
H  -1.96593 -3.55931  1.00002
H  -1.53330 -3.36631  2.71147
H  -0.30640 -3.05856  1.45480
H   0.03279 -0.77822  2.69423
H  -1.32901 -1.13461  3.79281
H  -1.33962  0.36722  2.83611
H  -3.54245 -1.88473  2.94043
H  -3.95033 -1.86035  1.21370
H  -3.67069 -0.32758  2.09851
C   5.69810 -1.25387 -0.25207
H   6.16022 -0.94029  0.69845
H   6.11611 -0.64865 -1.07324
H   5.93224 -2.31599 -0.43281


SCF(BP86) = -1053.45861377
H 0K = -1053.005853
H 298K = -1052.975617
G 298K = -1053.069791
Solvent Correction(CH3Cl) = -0.04347255
BP86-D3 Correction = -0.09110686
Lowest frequencies = 17.6703 cm-1, 18.8994 cm-1
```

***IntI$_{4'}$ (anti)**

65

```
P  -1.23303 -0.99748  0.02463
C  -1.58949 -1.98839 -1.61319
C  -1.20099 -2.06716  1.64109
Au  0.89540 -0.00025 -0.26283
C   3.18552  0.57635  0.02416
C   2.68266  1.00604 -1.19606
C   4.14297 -0.59910  0.26323
C  -0.19901 -3.22387  1.43890
C  -2.57549 -2.62981  2.05941
C  -0.69249 -1.12082  2.75291
C  -2.72561 -3.02583 -1.50185
C  -0.28793 -2.70889 -2.03618
C  -1.94212 -0.92285 -2.67634
O   5.43699  0.04843  0.28468
C   4.19644 -1.60689 -0.88863
C   3.85981 -1.28558  1.60734
H   4.64931 -2.02370  1.82279
H   2.88838 -1.80802  1.59346
H   3.83311 -0.55077  2.43326
H   3.21330 -2.08000 -1.04787
H   4.93024 -2.39184 -0.64928
H   4.52134 -1.12295 -1.82316
H   5.58101  0.41025  1.18159
H   2.88616  0.45012 -2.11816
H   2.30752  2.03158 -1.30491
H   3.11155  1.27277  0.87353
C  -2.60435  0.25345  0.20642
C  -4.84664  1.99246  0.27707
C  -5.04076  0.60499  0.30905
C  -3.92966 -0.24627  0.26880
C  -2.40520  1.66567  0.19129
C  -3.54535  2.50453  0.21871
H  -5.70155  2.67499  0.30231
H  -6.04844  0.18175  0.35913
H  -4.10400 -1.32349  0.27925
C  -1.09042  2.38877  0.16507
H  -3.38845  3.58778  0.20967
C   1.18160  4.08454  0.21381
C   0.38918  3.99706 -0.94415
C  -0.74088  3.16239 -0.96585
C  -0.28062  2.47914  1.32252
C   0.84345  3.32283  1.34630
H   2.04222  4.76044  0.24173
H   0.63402  4.60024 -1.82446
H  -1.37521  3.12324 -1.85767
H  -0.56574  1.92612  2.22295
H   1.43482  3.40979  2.26399
H  -1.15296 -0.15513 -2.76690
H  -2.89643 -0.41703 -2.46190
H  -2.03408 -1.42115 -3.65787
H  -3.69767 -2.57375 -1.25319
H  -2.50197 -3.82035 -0.77211
H  -2.84464 -3.51503 -2.48540
H  -0.46277 -3.20497 -3.00777
H   0.02412 -3.48484 -1.32054
H   0.54875 -2.00053 -2.16559
H  -0.57664 -3.98706  0.73945
H  -0.03347 -3.72449  2.40965
H   0.77997 -2.86799  1.07346
H   0.31320 -0.72308  2.53074
H  -0.63284 -1.68454  3.70127
H  -1.37899 -0.27266  2.91434
H  -2.43241 -3.24347  2.96711
H  -3.02636 -3.28194  1.29536
H  -3.28969 -1.83233  2.31538
```

SCF(BP86) = -1192.44559865
H 0K = -1191.900055
H 298K = -1191.865950
G 298K = -1191.966443
Solvent Correction(CH3Cl) = -0.04452049

```
BP86-D3 Correction = -0.11504354
Lowest frequencies = 11.5954 cm-1, 23.7161 cm-1
```

IntI$_{4'}$ (anti)

```
78

P   2.39266 -0.92570  0.32330
C   2.08797 -1.86546  2.00133
C   3.15321 -2.00362 -1.09698
Au  0.27436 -0.15451 -0.40426
C  -1.69112  0.23371 -1.56888
C  -1.84470  0.71236 -0.27511
S  -4.95869  1.81895 -0.28738
C  -2.36971 -1.02112 -2.13658
C   2.25836 -3.24555 -1.29816
C   4.61298 -2.44150 -0.85604
C   3.10417 -1.11438 -2.36091
C   3.24278 -2.78931  2.43981
C   0.79680 -2.70458  1.85820
C   1.85512 -0.76314  3.05990
C  -5.71109  0.60019  0.80212
O  -3.62684 -0.45677 -2.61062
C  -2.65172 -2.11859 -1.09917
C  -1.56537 -1.56735 -3.32842
H  -2.10369 -2.40604 -3.80445
H  -0.58329 -1.94952 -3.00215
H  -1.40168 -0.78224 -4.08545
H  -1.71723 -2.50532 -0.65708
H  -3.16868 -2.96170 -1.58759
H  -3.30656 -1.75116 -0.29315
H  -3.97017 -1.04095 -3.31474
H  -2.36520  0.13617  0.49636
H  -1.60715  1.75622 -0.03984
H  -1.35302  0.94256 -2.33769
C  -6.91097 -1.22643  2.58078
C  -6.61170 -1.61332  1.26473
C  -6.01675 -0.70760  0.37214
C  -6.00999  0.99243  2.12296
C  -6.60992  0.07917  3.00252
H  -7.38171 -1.93378  3.27002
H  -6.85181 -2.62507  0.92114
H  -5.79778 -1.00886 -0.65738
H  -5.77910  2.00854  2.46019
H  -6.84438  0.39437  4.02443
C   3.59821  0.44971  0.68887
C   5.49507  2.39406  1.51226
C   5.80031  1.03128  1.62852
C   4.85712  0.07825  1.22486
C   3.29395  1.83661  0.55298
C   4.25875  2.77994  0.98179
H   6.21589  3.15486  1.82693
H   6.76235  0.70576  2.03546
H   5.10918 -0.97747  1.33805
C   2.04228  2.43042 -0.02302
H   4.02281  3.84329  0.87332
C  -0.12176  3.89248 -1.12702
C   0.10225  3.90028  0.26089
C   1.17909  3.18193  0.80727
C   1.80233  2.42388 -1.41792
C   0.73121  3.15219 -1.96418
H  -0.94224  4.47712 -1.55490
H  -0.54713  4.48722  0.91849
H   1.36991  3.21732  1.88507
H   2.49089  1.88944 -2.07985
H   0.58349  3.16500 -3.04923
H   1.04270 -0.07521  2.76542
H   2.76186 -0.16876  3.25248
H   1.55536 -1.24350  4.00839
H   4.18630 -2.24826  2.60750
H   3.42140 -3.61009  1.72679
H   2.96685 -3.25378  3.40373
H   0.57479 -3.17409  2.83340
H   0.89110 -3.51273  1.11656
H  -0.06903 -2.07886  1.58086
H   2.34787 -3.96486 -0.46821
H   2.57700 -3.76759 -2.21820
H   1.19392 -2.97711 -1.41590
H   2.07321 -0.80954 -2.61322
```

```
H   3.50769 -1.68650 -3.21569
H   3.72159 -0.20732 -2.24817
H   4.92663 -3.07227 -1.70734
H   4.73854 -3.04370  0.05705
H   5.30194 -1.58397 -0.81603
H  -4.70679  0.96387 -1.32262
```

SCF(BP86) = -1434.91707953
H 0K = -1434.274253
H 298K = -1434.231582
G 298K = -1434.356058
Solvent Correction(CH3Cl) = -0.04458081
BP86-D3 Correction = -0.13486028
Lowest frequencies = 5.4610 cm-1, 7.3449 cm-1

TS(I-II)$_{4'}$ (anti)

78

```
P  -2.34849 -1.00042 -0.16416
C  -2.03983 -2.23570 -1.64069
C  -3.15880 -1.81709  1.39520
Au -0.23494 -0.12274  0.45739
C   1.71809  0.60055  1.05922
C   2.36095  0.59126 -0.25424
S   4.36219  1.49711 -0.16838
C   2.34876 -0.20621  2.20865
C  -2.25453 -2.98264  1.85191
C  -4.60406 -2.31874  1.19770
C  -3.16316 -0.71198  2.47696
C  -3.17266 -3.24685 -1.91307
C  -0.73913 -3.01827 -1.34052
C  -1.80987 -1.34913 -2.88613
C   5.47543  0.29821 -0.91600
O   3.62023  0.51791  2.52156
C   2.69343 -1.65912  1.84744
C   1.48477 -0.13710  3.47545
H   1.99603 -0.61994  4.32705
H   0.52594 -0.65782  3.31509
H   1.27287  0.91013  3.74415
H   1.77788 -2.21830  1.59485
H   3.17232 -2.16764  2.70349
H   3.38205 -1.72080  0.98882
H   4.11189 -0.00298  3.18849
H   2.67547 -0.37002 -0.68012
H   1.93297  1.27272 -0.99798
H   1.48120  1.62474  1.39878
C   7.20937 -1.51602 -2.16063
C   6.92438 -1.64401 -0.79102
C   6.05495 -0.74137 -0.16109
C   5.76085  0.43769 -2.28884
C   6.62762 -0.47685 -2.90548
H   7.88997 -2.22206 -2.64551
H   7.38316 -2.44695 -0.20580
H   5.83764 -0.83606  0.90704
H   5.31917  1.25630 -2.86625
H   6.85394 -0.36960 -3.97065
C  -3.56964  0.26841 -0.79169
C  -5.46999  2.04068 -1.93968
C  -5.75222  0.67004 -1.86412
C  -4.80845 -0.19696 -1.29945
C  -3.29080  1.66497 -0.85314
C  -4.25482  2.52096 -1.43799
H  -6.19188  2.73456 -2.38134
H  -6.69778  0.27180 -2.24465
H  -5.04409 -1.26173 -1.25894
C  -2.06120  2.35699 -0.33831
H  -4.03460  3.59244 -1.48116
C   0.06260  3.99886  0.56879
C  -0.06164  3.69468 -0.79790
C  -1.11913  2.88722 -1.24908
C  -1.92349  2.66689  1.03402
C  -0.87168  3.48246  1.48264
H   0.86635  4.65645  0.91569
H   0.64570  4.11532 -1.52073
H  -1.23570  2.68237 -2.31870
H  -2.66894  2.29849  1.74557
H  -0.79735  3.73353  2.54580
H  -1.01399 -0.60303 -2.71363
H  -2.72323 -0.81698 -3.19489
H  -1.48974 -1.99190 -3.72601
H  -4.12562 -2.76640 -2.18197
H  -3.34351 -3.92706 -1.06318
H  -2.87914 -3.87438 -2.77445
H  -0.50249 -3.65558 -2.21207
H  -0.83212 -3.67786 -0.46364
H   0.11346 -2.33855 -1.16996
H  -2.30384 -3.84373  1.16549
H  -2.59592 -3.33417  2.84239
H  -1.19971 -2.67106  1.94878
H  -2.14623 -0.33933  2.69008
```

```
H  -3.57906 -1.12743  3.41279
H  -3.79501  0.14321  2.18324
H  -4.94258 -2.78124  2.14273
H  -4.69161 -3.08459  0.41161
H  -5.30003 -1.49625  0.97124
H   4.35021  1.00593  1.16525
```

SCF(BP86) = -1434.91043321
H 0K = -1434.266759
H 298K = -1434.226088
G 298K = -1434.344195
Solvent Correction(CH3Cl) = -0.04032521
BP86-D3 Correction = -0.13691659
Lowest frequencies = -96.1449 cm-1, 5.2745 cm-1

IntII$_{4'}$ (anti)

```
78

P  -2.39319 -1.00273 -0.11540
C  -2.03855 -2.30064 -1.52319
C  -3.27937 -1.72106  1.45120
Au -0.29163 -0.14786  0.54575
C   1.77292  0.72170  0.79081
C   2.57080  0.52805 -0.47649
S   4.23757  1.40731 -0.27361
C   1.72532 -0.17543  1.87312
C  -2.43392 -2.89486  1.99075
C  -4.73078 -2.18281  1.20447
C  -3.29298 -0.57066  2.48384
C  -3.19357 -3.28317 -1.80798
C  -0.78127 -3.10952 -1.12533
C  -1.72079 -1.47068 -2.78797
C   5.37635  0.20319 -0.99956
O   4.70064  0.32726  2.85135
C   2.34740 -1.54863  1.84445
C   1.34836  0.32893  3.24729
H   2.30351  0.46111  3.79298
H   0.74074 -0.39954  3.80865
H   0.82724  1.29800  3.21513
H   1.76311 -2.26250  2.44772
H   3.34605 -1.44984  2.31421
H   2.47711 -1.95799  0.83196
H   5.51173  0.64502  3.28785
H   2.79534 -0.52559 -0.69356
H   2.06911  0.98155 -1.34418
H   1.50609  1.76753  1.00997
C   7.20030 -1.60628 -2.12410
C   6.93463 -1.64372 -0.74508
C   6.02361 -0.74041 -0.17528
C   5.64818  0.25108 -2.38189
C   6.55894 -0.65946 -2.94015
H   7.91416 -2.31072 -2.56249
H   7.44189 -2.37488 -0.10741
H   5.81340 -0.75306  0.90011
H   5.15646  1.00267 -3.00725
H   6.77338 -0.62238 -4.01293
C  -3.52034  0.29020 -0.84475
C  -5.28259  2.08000 -2.16465
C  -5.62911  0.72774 -2.04004
C  -4.75215 -0.14990 -1.39094
C  -3.17582  1.66911 -0.95374
C  -4.07307  2.53384 -1.62589
H  -5.95121  2.78135 -2.67310
H  -6.57222  0.35182 -2.44795
H  -5.03430 -1.20149 -1.31453
C  -1.94955  2.34071 -0.40400
H  -3.80643  3.59243 -1.70648
C   0.13550  4.01307  0.54144
C   0.06871  3.66793 -0.81958
C  -0.96817  2.84492 -1.28978
C  -1.86283  2.68407  0.96655
C  -0.83090  3.51557  1.43312
H   0.92234  4.68429  0.90036
H   0.80820  4.06431 -1.52294
H  -1.04251  2.61332 -2.35769
H  -2.63868  2.33935  1.65708
H  -0.80124  3.79927  2.49031
H  -0.90832 -0.74441 -2.60768
H  -2.59982 -0.92286 -3.16188
H  -1.38251 -2.15671 -3.58505
H  -4.11556 -2.78151 -2.13890
H  -3.42531 -3.92323 -0.94168
H  -2.88331 -3.95298 -2.63030
H  -0.52799 -3.79384 -1.95511
H  -0.93480 -3.72383 -0.22473
H   0.08809 -2.45254 -0.94984
H  -2.48321 -3.78117  1.33764
H  -2.83048 -3.19430  2.97747
H  -1.37447 -2.61515  2.12515
H  -2.27494 -0.22298  2.73058
```

```
H  -3.76340 -0.93427  3.41513
H  -3.88346  0.29051  2.12847
H  -5.12654 -2.59303  2.15110
H  -4.80864 -2.97895  0.44809
H  -5.38680 -1.34820  0.91279
H   4.65720  0.83503  2.00643
```

SCF(BP86) = -1434.93757839
H 0K = -1434.292793
H 298K = -1434.249845
G 298K = -1434.373104
Solvent Correction(CH3Cl) = -0.04117649
BP86-D3 Correction = -0.13531985
Lowest frequencies = 7.1499 cm-1, 12.6539 cm-1

*IntII$_{4'}$ (anti)

75

P	2.31105	-0.52148	0.67634
C	1.89209	-0.98317	2.52130
C	3.60212	-1.68457	-0.18301
Au	0.27936	-0.65596	-0.51925
C	-1.82761	-0.56641	-1.32946
C	-2.79148	-0.39305	-0.18101
S	-4.52424	-0.49745	-0.90583
C	-1.39006	-1.79821	-1.84427
C	3.09267	-3.13816	-0.07863
C	5.02937	-1.58053	0.39434
C	3.63384	-1.24833	-1.66569
C	3.10983	-1.37792	3.38242
C	0.88713	-2.15914	2.50644
C	1.19715	0.26180	3.11858
C	-5.49493	0.13280	0.48179
C	-1.72561	-3.12906	-1.21124
C	-0.80678	-1.87395	-3.24026
H	-1.56902	-2.31298	-3.91231
H	0.07171	-2.53934	-3.28333
H	-0.53253	-0.88371	-3.63418
H	-0.87242	-3.82485	-1.27267
H	-2.55479	-3.58885	-1.78144
H	-2.04645	-3.04783	-0.16314
H	-2.68216	-1.16010	0.60121
H	-2.68065	0.60352	0.27232
H	-1.74561	0.30418	-1.99812
C	-7.07458	1.09778	2.59435
C	-6.72947	-0.26305	2.54136
C	-5.94323	-0.75016	1.48567
C	-5.84683	1.49720	0.53105
C	-6.63590	1.97534	1.58895
H	-7.69383	1.47291	3.41524
H	-7.08113	-0.95006	3.31772
H	-5.68637	-1.81251	1.42649
H	-5.51170	2.17017	-0.26432
H	-6.91477	3.03341	1.62287
C	3.03787	1.19430	0.70059
C	4.17115	3.77533	1.02471
C	4.75436	2.67393	1.66575
C	4.18653	1.40423	1.50439
C	2.45562	2.31060	0.03187
C	3.04028	3.58603	0.22199
H	4.59582	4.77695	1.14238
H	5.64234	2.79703	2.29301
H	4.64785	0.56179	2.02284
C	1.27586	2.29259	-0.89747
H	2.59218	4.44090	-0.29432
C	-0.81355	2.69277	-2.77221
C	-0.96702	3.11743	-1.44108
C	0.07056	2.92630	-0.51362
C	1.41255	1.85617	-2.23732
C	0.37800	2.05912	-3.16622
H	-1.60819	2.87489	-3.50272
H	-1.88513	3.62599	-1.12995
H	-0.03589	3.29661	0.51158
H	2.35567	1.40621	-2.56228
H	0.51804	1.75045	-4.20751
H	0.33225	0.58054	2.51003
H	1.88363	1.11659	3.22132
H	0.81894	0.00612	4.12453
H	3.85256	-0.57115	3.47500
H	3.61148	-2.28588	3.01154
H	2.75317	-1.60060	4.40428
H	0.58852	-2.37763	3.54757
H	1.31354	-3.08187	2.08368
H	-0.02506	-1.90814	1.93789
H	3.15386	-3.53115	0.94915
H	3.72700	-3.78321	-0.71270
H	2.05173	-3.23896	-0.43224
H	2.64433	-1.34025	-2.14604
H	4.33957	-1.89902	-2.21277
H	3.98551	-0.20907	-1.77908

```
H   5.67163 -2.29899 -0.14627
H   5.08325 -1.83782  1.46331
H   5.46440 -0.58023  0.24568


SCF(BP86) = -1358.50460683
H 0K = -1357.883625
H 298K = -1357.843728
G 298K = -1357.961072
Solvent Correction(CH3Cl) = -0.04237948
BP86-D3 Correction = -0.12740843
Lowest frequencies = 5.7573 cm-1, 12.9741 cm-1
```

IntIII$_{24'}$ (anti)

```
88

P   3.29594  0.18656  0.27448
C   4.09250  1.51735 -0.90564
C   3.88716  0.27596  2.12053
Au  0.96160  0.58404  0.25125
C  -1.26275  0.94461 -0.02152
C  -1.55792  1.11817 -1.49085
S  -3.40522  1.23829 -1.79120
C  -0.90609  1.96064  0.88376
C   3.63139  1.70603  2.64336
C   5.36570 -0.10677  2.34139
C   2.99666 -0.72613  2.89052
C   5.60917  1.72492 -0.71547
C   3.36991  2.86493 -0.67590
C   3.79672  1.02947 -2.34230
C  -3.76476  2.88303 -1.13697
S  -4.34347 -0.97021  1.45446
C  -0.62106  3.39105  0.48381
C  -1.09295  1.73728  2.37158
H  -1.98135  2.31082  2.69821
H  -0.23902  2.11889  2.95591
H  -1.26290  0.67874  2.62103
H   0.21361  3.80539  1.07351
H  -1.51392  4.00394  0.71025
H  -0.39487  3.51256 -0.58485
C  -5.06067 -2.51405  0.86532
H  -1.07312  1.99485 -1.94573
H  -1.26771  0.21928 -2.05794
H  -1.68081  0.02513  0.41604
C  -4.34475  5.44762 -0.14432
C  -3.74100  5.30238 -1.40588
C  -3.45824  4.02340 -1.90862
C  -4.38664  3.02644  0.12015
C  -4.67550  4.31068  0.61202
H  -4.57318  6.44637  0.24077
H  -3.50462  6.18626 -2.00692
H  -3.01126  3.90236 -2.90075
H  -4.65486  2.13910  0.70220
H  -5.16655  4.42052  1.58429
C   3.80296 -1.47751 -0.39571
C   4.79148 -3.84643 -1.60733
C   5.68925 -2.90571 -1.08481
C   5.19329 -1.73726 -0.49381
C   2.88731 -2.44398 -0.90790
C   3.41554 -3.60995 -1.51486
H   5.15758 -4.76412 -2.07775
H   6.76936 -3.07178 -1.13891
H   5.90988 -1.01015 -0.10866
C   1.39102 -2.38341 -0.85568
H   2.71052 -4.35294 -1.90078
C  -1.42651 -2.71664 -0.82624
C  -0.74296 -2.57186 -2.04623
C   0.65241 -2.41143 -2.06182
C   0.68933 -2.51362  0.36825
C  -0.70620 -2.68377  0.37971
H  -2.51022 -2.87245 -0.81078
H  -1.29292 -2.61340 -2.99242
H   1.18477 -2.33443 -3.01595
H   1.24941 -2.55174  1.30764
H  -1.23201 -2.81194  1.33107
H   2.71793  0.85410 -2.50208
H   4.33947  0.10409 -2.59030
H   4.11563  1.81215 -3.05378
H   6.19587  0.81573 -0.91539
H   5.86017  2.10021  0.28955
H   5.94802  2.48794 -1.43943
H   3.75995  3.59990 -1.40299
H   3.53911  3.27479  0.33180
H   2.28152  2.77537 -0.83426
H   4.32339  2.44266  2.20393
H   3.79223  1.71866  3.73631
H   2.59584  2.03786  2.45259
H   1.92494 -0.47445  2.80964
```

```
H   3.27193 -0.69820  3.96025
H   3.14485 -1.76076  2.53777
H   5.59480  0.01553  3.41544
H   6.06658  0.53496  1.78538
H   5.56425 -1.15873  2.08520
C  -6.17869 -4.96892  0.05496
C  -5.77896 -4.77874  1.38791
C  -5.21898 -3.55968  1.79750
C  -5.46653 -2.69673 -0.47249
C  -6.02051 -3.92458 -0.86946
H  -6.61564 -5.92148 -0.25901
H  -5.90313 -5.58374  2.11943
H  -4.91118 -3.41996  2.83933
H  -5.36803 -1.88067 -1.19639
H  -6.33764 -4.05672 -1.90915
H  -4.26345 -0.34783  0.23703
```

SCF(BP86) = -1600.97726954
H 0K = -1600.258705
H 298K = -1600.210524
G 298K = -1600.350035
Solvent Correction(CH3Cl) = -0.04197091
BP86-D3 Correction = -0.15491520
Lowest frequencies = 3.4136 cm-1, 9.8228 cm-1

TS(III-IV)$_{4'}$ (anti)

```
88

P  -2.99309  0.29247 -0.79215
C  -3.24435  2.16600 -1.27289
C  -3.51106 -0.95811 -2.18245
Au -0.67647 -0.01454 -0.37423
C   1.48329 -0.29215 -0.12871
C   1.67657  0.86165  0.75310
S   3.70044  1.03958  1.58274
C   2.20432 -0.38421 -1.47179
C  -2.72923 -0.59806 -3.46470
C  -5.02061 -1.02128 -2.49451
C  -3.05627 -2.34519 -1.67078
C  -4.55690  2.48901 -2.01567
C  -2.05134  2.59579 -2.15882
C  -3.18181  2.94970  0.05830
C   4.29607  2.62208  0.95693
S   4.10837 -0.79172 -1.19263
C   2.28011  0.94183 -2.24667
C   1.64966 -1.51214 -2.34938
H   2.24832 -1.63281 -3.26753
H   0.61253 -1.26506 -2.63903
H   1.63069 -2.47471 -1.81275
H   1.25337  1.29829 -2.44320
H   2.78911  0.80305 -3.21418
H   2.81420  1.73220 -1.69260
C   4.15617 -2.48256 -0.56613
H   1.64855  1.86379  0.30688
H   1.15767  0.80196  1.71650
H   1.51750 -1.25464  0.41063
C   5.22675  5.11526  0.07693
C   4.16488  5.04604  0.99358
C   3.69525  3.80139  1.44070
C   5.36702  2.68192  0.04211
C   5.82552  3.93510 -0.39347
H   5.59256  6.08814 -0.26463
H   3.70225  5.96323  1.37100
H   2.87698  3.74843  2.16630
H   5.84604  1.76581 -0.31577
H   6.65956  3.98455 -1.10036
C  -4.13395 -0.01295  0.65958
C  -5.99715 -0.19525  2.79902
C  -6.44175  0.14732  1.51514
C  -5.51572  0.23948  0.46860
C  -3.68547 -0.38157  1.96243
C  -4.63801 -0.45367  3.00821
H  -6.70256 -0.26803  3.63253
H  -7.50029  0.34850  1.32419
H  -5.88159  0.52310 -0.51913
C  -2.28371 -0.72970  2.36406
H  -4.28732 -0.74095  4.00465
C   0.20805 -1.53914  3.44440
C  -0.37354 -0.32321  3.84357
C  -1.61209  0.07387  3.31323
C  -1.68285 -1.94481  1.96225
C  -0.44969 -2.34742  2.50191
H   1.15483 -1.86630  3.88633
H   0.12135  0.30321  4.59339
H  -2.07898  1.00556  3.65012
H  -2.20946 -2.59962  1.26132
H  -0.01604 -3.30664  2.20081
H  -2.25881  2.72426  0.62139
H  -4.04535  2.73873  0.70837
H  -3.18122  4.03163 -0.16633
H  -5.45452  2.26431 -1.41990
H  -4.63148  1.96980 -2.98475
H  -4.57943  3.57358 -2.22802
H  -2.13104  3.68059 -2.35518
H  -2.03740  2.08325 -3.13324
H  -1.08619  2.40805 -1.65736
H  -3.10098  0.32764 -3.93365
H  -2.85575 -1.41180 -4.20151
H  -1.64850 -0.48312 -3.27127
H  -1.97030 -2.37685 -1.47485
```

```
H  -3.28764 -3.10474 -2.43956
H  -3.58893 -2.63421 -0.74873
H  -5.17201 -1.73945 -3.32093
H  -5.43517 -0.05601 -2.82465
H  -5.60663 -1.38579 -1.63686
C   4.35804 -5.13863  0.33456
C   4.53286 -4.84392 -1.02752
C   4.43543 -3.51965 -1.48127
C   3.99119 -2.77696  0.80396
C   4.09041 -4.10530  1.24739
H   4.44145 -6.17145  0.68652
H   4.75299 -5.64462 -1.74040
H   4.58363 -3.28315 -2.53929
H   3.80532 -1.97715  1.52652
H   3.97105 -4.33012  2.31205
H   4.10762  0.20817  0.41940
```

SCF(BP86) = -1600.95663792
H 0K = -1600.238612
H 298K = -1600.192471
G 298K = -1600.323635
Solvent Correction(CH3Cl) = -0.03793615
BP86-D3 Correction = -0.15806278
Lowest frequencies = -143.7779 cm-1, 6.9881 cm-1

IntIV$_{4'}$ (anti)

88

```
P  -2.75292 -0.67575  0.87894
C  -2.80292 -2.62254  0.84809
C  -2.93595  0.11621  2.63955
Au -0.65575 -0.06024 -0.02529
C   1.60231  0.94213 -0.20914
C   1.24137  0.02145 -1.19243
S   2.59231 -3.69164 -1.90711
C   2.47754  0.70457  0.99258
C  -1.85928 -0.49831  3.55944
C  -4.33011 -0.05090  3.27928
C  -2.65791  1.62426  2.44311
C  -3.88685 -3.25460  1.74513
C  -1.41582 -3.14805  1.28677
C  -3.03998 -3.01118 -0.62936
C   4.20332 -3.69568 -1.08907
S   4.12880  1.46663  0.37646
C   3.89753  3.25223  0.47905
H   1.63483 -1.00432 -1.18854
H   0.86154  0.39757 -2.15185
H   1.33124  1.99528 -0.37191
C   6.64198 -3.71354  0.31480
C   6.48849 -2.90693 -0.82501
C   5.27016 -2.88315 -1.52238
C   4.35612 -4.51522  0.04648
C   5.57198 -4.51237  0.74835
H   7.59317 -3.72594  0.85529
H   7.31930 -2.28688 -1.17696
H   5.15486 -2.23943 -2.40060
H   3.53656 -5.16727  0.36565
H   5.68636 -5.15589  1.62663
C  -4.19903 -0.09109 -0.14648
C  -6.50669  0.51477 -1.68621
C  -6.63529 -0.21451 -0.49652
C  -5.49161 -0.51343  0.25420
C  -4.06987  0.66594 -1.34834
C  -5.23921  0.94309 -2.09699
H  -7.38695  0.75432 -2.29056
H  -7.61613 -0.55677 -0.15300
H  -5.61247 -1.09628  1.16880
C  -2.80433  1.23213 -1.92207
H  -5.13527  1.52644 -3.01740
C  -0.64684  2.51590 -3.23750
C  -1.24668  1.37979 -3.80748
C  -2.32124  0.74843 -3.15958
C  -2.18644  2.37127 -1.35352
C  -1.11932  3.00927 -2.00877
H   0.16827  3.02715 -3.75957
H  -0.89556  0.99787 -4.77160
H  -2.80741 -0.11692 -3.62222
H  -2.57812  2.78587 -0.41946
H  -0.67721  3.91018 -1.57058
H  -2.28570 -2.56014 -1.29836
H  -4.04068 -2.71899 -0.98433
H  -2.95218 -4.10835 -0.72367
H  -4.90799 -2.96343  1.45585
H  -3.73753 -3.02571  2.81255
H  -3.82705 -4.35288  1.63888
H  -1.41423 -4.24908  1.19331
H  -1.17747 -2.90469  2.33368
H  -0.60767 -2.75462  0.64624
H  -2.08301 -1.54456  3.82354
H  -1.82610  0.07673  4.50225
H  -0.85379 -0.45928  3.10578
H  -1.65049  1.80844  2.03144
H  -2.72350  2.13019  3.42327
H  -3.40187  2.09367  1.77738
H  -4.30342  0.40787  4.28430
H  -4.62159 -1.10443  3.41172
H  -5.11433  0.46726  2.70634
C   3.70302  6.05729  0.58671
C   3.43149  5.38055 -0.61421
```

```
C   3.53003  3.98144 -0.67267
C   4.18711  3.93643  1.67958
C   4.08305  5.33521  1.73021
H   3.63223  7.14874  0.62764
H   3.15496  5.94364 -1.51148
H   3.34601  3.44902 -1.61109
H   4.50451  3.37094  2.56067
H   4.31168  5.86193  2.66213
H   3.04394 -3.39239 -3.15582
C   2.81367 -0.76702  1.26277
C   1.95899  1.41613  2.24549
H   2.68461  1.32322  3.06953
H   1.00978  0.94820  2.56526
H   1.77119  2.48680  2.06574
H   3.48546 -0.84836  2.13122
H   3.30496 -1.25648  0.40591
H   1.88553 -1.32287  1.48869
```

SCF(BP86) = -1600.97012527
H 0K = -1600.252288
H 298K = -1600.203850
G 298K = -1600.345853
Solvent Correction(CH3Cl) = -0.04138690
BP86-D3 Correction = -0.14828263
Lowest frequencies = 4.3131 cm-1, 8.1708 cm-1

*IntIV$_{4'}$ (anti)

```
75

P  -2.26960 -0.83935  0.35248
C  -3.16296 -1.98366 -0.94548
C  -1.94852 -1.66408  2.07823
Au -0.19917 -0.23293 -0.61845
C   2.27146 -0.08654 -0.94383
C   1.45574  0.36728 -1.98069
C   3.00189 -1.39866 -0.85965
C  -1.18911 -2.98825  1.84988
C  -3.22188 -1.92553  2.90942
C  -1.04787 -0.67150  2.84868
C  -4.33696 -2.80886 -0.37952
C  -2.11068 -2.94831 -1.54121
C  -3.66410 -1.03949 -2.06225
S   4.77519 -0.81534 -1.31210
C   5.40801 -0.01165  0.17232
H   1.32540 -0.23087 -2.88972
H   1.25059  1.44251 -2.06786
H   2.51729  0.62221 -0.14017
C  -3.39450  0.62285  0.63548
C  -5.32835  2.68189  0.92373
C  -5.66578  1.36025  1.24481
C  -4.70761  0.35016  1.09520
C  -3.04481  1.97070  0.32676
C  -4.03559  2.97176  0.47286
H  -6.06368  3.48555  1.02791
H  -6.66846  1.10986  1.60407
H  -4.99590 -0.67420  1.33655
C  -1.70680  2.47341 -0.13059
H  -3.76225  4.00521  0.23737
C   0.67347  3.80850 -0.89829
C  -0.39425  3.68974 -1.80490
C  -1.57634  3.03466 -1.42208
C  -0.62036  2.58950  0.76947
C   0.55750  3.25475  0.38886
H   1.58080  4.34934 -1.18572
H  -0.31817  4.12989 -2.80442
H  -2.41883  2.97443 -2.11907
H  -0.72226  2.20330  1.78836
H   1.37261  3.36589  1.11153
H  -2.84646 -0.42254 -2.47586
H  -4.46525 -0.36836 -1.71549
H  -4.06729 -1.65251 -2.88807
H  -5.15288 -2.18285  0.01216
H  -4.01805 -3.51395  0.40470
H  -4.76475 -3.41135 -1.20111
H  -2.59335 -3.54566 -2.33558
H  -1.70542 -3.65203 -0.79801
H  -1.26701 -2.40059 -1.99596
H  -1.82989 -3.76805  1.40753
H  -0.83702 -3.36926  2.82528
H  -0.30473 -2.85281  1.20366
H  -0.09554 -0.48349  2.32315
H  -0.81149 -1.09936  3.83963
H  -1.55450  0.29378  3.01727
H  -2.92114 -2.42028  3.85065
H  -3.93693 -2.59463  2.40598
H  -3.73750 -0.99221  3.18275
C   6.55026  1.26436  2.40331
C   5.92113  2.02687  1.40495
C   5.35245  1.39431  0.28835
C   6.05650 -0.77433  1.16786
C   6.61995 -0.13350  2.28210
H   6.99923  1.76099  3.26912
H   5.88424  3.11780  1.48836
H   4.88544  1.98319 -0.50727
H   6.12814 -1.86024  1.05661
H   7.12525 -0.72745  3.05017
C   2.63905 -2.40876 -1.95521
C   2.94490 -2.01998  0.53817
H   3.59623 -2.90706  0.59272
H   1.90849 -2.33698  0.75505
H   3.25818 -1.30919  1.31930
```

```
H   3.24468 -3.32141 -1.84501
H   2.80930 -2.00977 -2.96905
H   1.57330 -2.68731 -1.86744


SCF(BP86) = -1358.49894062
H 0K = -1357.877748
H 298K = -1357.838321
G 298K = -1357.952691
Solvent Correction(CH3Cl) = -0.04115673
BP86-D3 Correction = -0.13085220
Lowest frequencies = 7.6803 cm-1, 14.9137 cm-1
```

[(Johnphos)Au(HSPh)]$^+$

62

P	1.647560	-0.905010	-0.011051
C	1.491862	-2.170696	1.457714
C	2.527021	-1.589028	-1.594416
Au	-0.521547	-0.257774	-0.601957
C	1.787935	-2.868602	-2.043037
C	4.030842	-1.877885	-1.405373
C	2.362356	-0.491087	-2.670533
C	2.751852	-3.025769	1.704580
C	0.297428	-3.105873	1.152978
C	1.163959	-1.327862	2.710956
S	-2.739723	0.300570	-1.325826
H	-2.622550	1.642697	-1.030408
C	2.652760	0.540699	0.604745
C	4.271479	2.581792	1.730883
C	4.754612	1.268170	1.663144
C	3.948223	0.266010	1.109230
C	2.166271	1.880191	0.660409
C	2.995169	2.873408	1.234889
H	4.884006	3.378608	2.163604
H	5.750141	1.018138	2.041950
H	4.337719	-0.753411	1.076421
C	0.843654	2.383064	0.152726
H	2.620098	3.901074	1.274405
C	-1.496266	3.713567	-0.746143
C	-1.306440	3.461872	0.626295
C	-0.144589	2.809388	1.071322
C	0.640171	2.639891	-1.225533
C	-0.515815	3.302712	-1.669863
H	-2.375580	4.270312	-1.087497
H	-2.049668	3.803738	1.353940
H	0.017705	2.649879	2.142512
H	1.415141	2.357491	-1.944369
H	-0.639316	3.520274	-2.735758
H	0.276558	-0.689736	2.553026
H	2.004769	-0.686065	3.017369
H	0.936394	-2.013278	3.546839
H	3.637015	-2.425408	1.965031
H	2.997294	-3.671034	0.846207
H	2.554770	-3.691973	2.563825
H	0.153030	-3.780795	2.015872
H	0.462486	-3.734953	0.264809
H	-0.638655	-2.540145	1.004424
H	1.961492	-3.715037	-1.359030
H	2.170147	-3.168097	-3.035321
H	0.699923	-2.708074	-2.138238
H	1.300603	-0.274259	-2.880629
H	2.828744	-0.841933	-3.608642
H	2.866366	0.446430	-2.380607
H	4.424709	-2.282656	-2.354977
H	4.233002	-2.627289	-0.624881
H	4.602892	-0.964614	-1.179381
C	-3.905102	-0.187370	-0.012387
C	-5.769846	-0.991926	1.901852
C	-4.597938	-0.325536	2.300431
C	-3.650710	0.074706	1.345511
C	-5.072511	-0.850006	-0.427290
C	-6.004209	-1.255755	0.543645
H	-6.500787	-1.305624	2.653052
H	-4.415086	-0.120446	3.359795
H	-2.729555	0.582119	1.647999
H	-5.255988	-1.041759	-1.488907
H	-6.916453	-1.772436	0.230774

SCF(BP86) = -1163.17418434
H 0K = -1162.668675
H 298K = -1162.635907
G 298K = -1162.735298
Solvent Correction(CH3Cl) = -0.041320
BP86-D3 Correction = -0.10851633
Lowest frequencies = 12.9497 cm-1, 15.1736 cm-1

5. Reactions of **9, 10** and **11** with PhSH

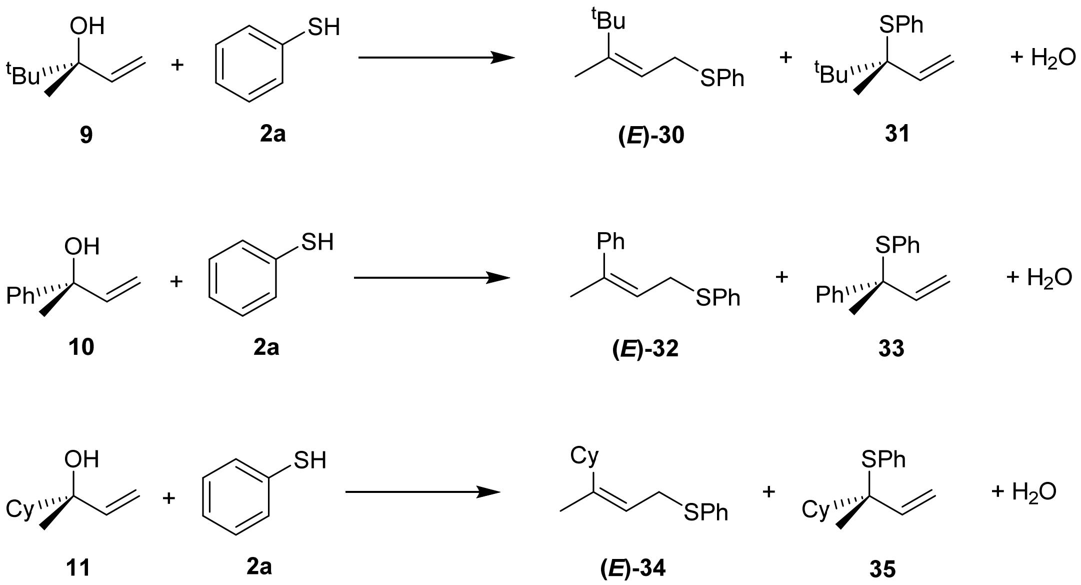

Figure S9. Reactions of **9**, **10** and **11** to form formal S_N2 and S_N2' products.

Table S4. Computed relative energies (kcal/mol) for the reactions of **9**, **10**, and **11** with PhSH. Energies are reported as ΔE (gas-phase SCF energies), ΔH_{298} (zero-point energy corrected energies at 298.15 K), ΔG (free energies at 298.15 K and 1 atm), ΔG_{disp} (including a correction for dispersion effects using Grimme's D3 parameter set) and $\Delta G_{CHCl3+disp}$ including an additional correction for $CHCl_3$ solvent via the PCM approach.

	**ΔE**	**ΔH_{298}**	**ΔG**	**ΔG_{disp}**	**$\Delta G_{CHCl3+disp}$**
			From **9**		
**(*E*)-30**	-4.8	-3.4	-3.9	-3.0	-4.9
**(*Z*)-30**	-0.7	+0.7	+1.0	+0.9	-0.9
**31**	+2.8	+3.8	+5.0	+0.3	-1.1
			From **10**		
**(*E*)-32**	-7.6	-6.3	-6.0	-8.7	-10.1
**(*Z*)-32**	-7.4	-6.1	-5.9	-7.0	-8.4
**33**	+0.9	+1.8	+3.1	-1.6	-2.8
			From **11**		
**(*E*)-34**	-5.1	-3.6	-4.6	-3.8	-5.6
**(*Z*)-34**	-4.7	-3.2	-4.1	-3.8	-5.6
**35**	+1.3	+2.3	+3.7	-1.0	-2.3

From 9
9

25

O	-0.722183	0.615017	-1.433114
C	-0.425258	0.488609	-0.027184
C	-0.339115	1.907179	0.589808
H	-0.055632	1.885642	1.655331
H	0.386931	2.527414	0.042429
H	-1.326980	2.398293	0.527290
C	0.952084	-0.296871	0.031077
C	-1.537862	-0.266367	0.693180
C	0.809558	-1.634733	-0.732592
C	2.066318	0.539020	-0.643483
C	1.360388	-0.599897	1.491001
H	2.984492	-0.067477	-0.734299
H	1.760621	0.856346	-1.653086
H	2.326082	1.437767	-0.058276
H	2.337659	-1.113559	1.500875
H	1.468101	0.316308	2.097195
H	0.636132	-1.263630	1.993103
H	1.761304	-2.193037	-0.690491
H	0.021773	-2.267554	-0.291547
H	0.554542	-1.460014	-1.789089
H	-1.494974	-0.243893	1.790571
C	-2.560085	-0.889783	0.084468
H	-3.351442	-1.386951	0.653875
H	-2.621083	-0.932484	-1.008185
H	-1.566229	1.103756	-1.488432

SCF(BP86) = -389.680346065
H 0K = -389.461263
H 298K = -389.449626
G 298K = -389.496665
Solvent Correction(CH3Cl) = -0.00244022
BP86-D3 Correction = -0.02995967
Lowest frequencies = 57.7975 cm-1, 108.0369 cm-1

(*E*)-30

35

S	-1.008660	-0.792300	-0.288734
C	-0.028960	0.611176	0.460246
C	1.383658	0.141239	0.666926
C	2.436802	0.381417	-0.152898
C	-2.714533	-0.271562	-0.082541
C	-3.706447	-1.260217	-0.277532
C	-5.063186	-0.931540	-0.164713
C	-5.455829	0.379587	0.156569
C	-4.472868	1.360180	0.355320
C	-3.109248	1.045567	0.231579
H	-3.408072	-2.288533	-0.509814
H	-5.818329	-1.709926	-0.318649
H	-6.516582	0.631896	0.251835
H	-4.763184	2.386999	0.603363
H	-2.362784	1.831849	0.374093
H	-0.096459	1.488807	-0.200703
H	-0.504733	0.855555	1.424842
H	1.529685	-0.466878	1.565174
C	3.852468	-0.146134	0.162240
C	2.286193	1.197182	-1.424738
C	4.242080	-1.226980	-0.884578
C	4.871267	1.023162	0.086794
C	3.956513	-0.783935	1.564982
H	2.903996	0.789637	-2.242433
H	1.247177	1.218689	-1.786672
H	2.614149	2.242812	-1.275365
H	5.887733	0.654999	0.311229
H	4.899927	1.484513	-0.914246
H	4.626449	1.811550	0.819849
H	5.257643	-1.607969	-0.676234
H	3.541770	-2.078729	-0.848988
H	4.244024	-0.831022	-1.913974
H	4.995616	-1.106809	1.749454
H	3.679288	-0.069768	2.359371
H	3.313804	-1.675724	1.661685

SCF(BP86) = -555.736680141
H 0K = -555.441540
H 298K = -555.424369
G 298K = -555.488472
Solvent Correction(CH3Cl) = -0.00379926
BP86-D3 Correction = -0.03732698
Lowest frequencies = 12.9665 cm-1, 28.0636 cm-1

(*Z*)-30

35

S	-0.891602	-0.997868	0.457832
C	0.210005	-0.085038	-0.745995
C	1.469972	-0.880339	-0.934037
C	2.738951	-0.638870	-0.510517
C	-2.512594	-0.288107	0.153913
C	-3.624785	-1.020008	0.629369
C	-4.922460	-0.518893	0.464858
C	-5.136452	0.707578	-0.187851
C	-4.034265	1.432157	-0.665230
C	-2.727209	0.947631	-0.491470
H	-3.467179	-1.987095	1.119441
H	-5.773047	-1.098938	0.838775
H	-6.152108	1.092364	-0.323193
H	-4.185043	2.391449	-1.172307
H	-1.882615	1.540980	-0.852756
H	-0.350129	-0.028456	-1.695641
H	0.380662	0.934324	-0.373599
H	1.309984	-1.804784	-1.505892
C	3.193107	0.596521	0.314489
C	3.801122	-1.643718	-0.917936
C	4.685308	0.503913	0.728830
C	3.041345	1.882638	-0.543785
C	2.375954	0.717720	1.628889
H	4.310208	-2.085580	-0.042603
H	3.364045	-2.467136	-1.504649
H	4.592309	-1.176696	-1.533250
H	3.396670	2.763182	0.021284
H	3.639802	1.810246	-1.468526
H	1.997072	2.071500	-0.839412
H	4.956119	1.402365	1.309947
H	4.885011	-0.372745	1.367969
H	5.362930	0.458036	-0.140006
H	2.717234	1.596799	2.204882
H	1.293272	0.823181	1.463919
H	2.522454	-0.176224	2.259131

SCF(BP86) = -555.73003848
H 0K = -555.434828
H 298K = -555.417817
G 298K = -555.480716
Solvent Correction(CH3Cl) = -0.00372069
BP86-D3 Correction = -0.03889275
Lowest frequencies = 18.1250 cm-1, 29.7730 cm-1

31

35

S	-0.032168	-0.821173	-0.976984
C	-1.133996	0.203532	0.245051
C	-0.936197	1.646460	-0.155858
C	1.642399	-0.439104	-0.435708
C	2.337144	-1.347346	0.392368
C	3.670389	-1.096682	0.753203
C	4.318590	0.062559	0.295977
C	3.631946	0.969060	-0.528453
C	2.300823	0.720429	-0.898600
H	1.824764	-2.248722	0.742466
H	4.203052	-1.809541	1.391706
H	5.359375	0.255874	0.576432
H	4.136210	1.870429	-0.893057
H	1.764225	1.415193	-1.549783
C	-2.633423	-0.271966	-0.018847
C	-0.667226	-0.085214	1.678323
H	-1.273943	1.895430	-1.169569
C	-0.370321	2.619454	0.583914
H	-0.260523	3.633016	0.185285
H	0.001648	2.452983	1.599208
H	-1.279835	0.470530	2.409385
H	-0.732753	-1.157806	1.910997
H	0.380959	0.221365	1.817197
C	-3.592106	0.653096	0.774565
C	-3.020244	-0.185532	-1.517784
C	-2.858606	-1.730562	0.448723
H	-4.636028	0.338745	0.599885
H	-3.410800	0.603946	1.861327
H	-3.494745	1.705784	0.461461
H	-3.873100	-2.056597	0.160420
H	-2.134709	-2.422662	-0.013067
H	-2.782836	-1.831519	1.543902
H	-4.078915	-0.475549	-1.635569
H	-2.914384	0.833581	-1.924365
H	-2.418019	-0.864815	-2.143758

SCF(BP86) = -555.724456545
H 0K = -555.429971
H 298K = -555.412956
G 298K = -555.474283
Solvent Correction(CH3Cl) = -0.00303443
BP86-D3 Correction = -0.04622588
Lowest frequencies = 22.3311 cm-1, 49.5870 cm-1

From 10
10

23

C	-1.931813	-0.874825	-0.529896
C	-3.035342	-0.615444	-1.249192
C	-1.187799	0.165040	0.306164
O	-1.649992	1.487788	-0.009011
H	-1.543472	1.585664	-0.976913
H	-3.482085	0.384210	-1.261625
H	-3.534130	-1.396398	-1.830918
H	-1.518127	-1.890912	-0.518604
C	0.339437	0.042086	0.089112
C	-1.517541	-0.024240	1.804808
H	-2.602030	0.091868	1.959898
H	-0.981055	0.732467	2.400034
H	-1.211096	-1.024075	2.150803
C	3.144063	-0.144511	-0.271723
C	2.495433	1.093969	-0.387013
C	1.105841	1.185649	-0.204850
C	1.003596	-1.196627	0.214827
C	2.391775	-1.290126	0.035895
H	4.227271	-0.217768	-0.414328
H	3.071942	1.996970	-0.615769
H	0.601154	2.154002	-0.270282
H	0.438804	-2.102161	0.463966
H	2.886863	-2.261988	0.137022

SCF(BP86) = -463.476951711
H 0K = -463.288465
H 298K = -463.277754
G 298K = -463.324429
Solvent Correction(CH3Cl) = -0.00382334
BP86-D3 Correction = -0.02252316
Lowest frequencies = 48.9435 cm-1, 82.5660 cm-1

(*E*)-32

33

C	0.351457	-1.691910	0.216827
C	-0.953826	-2.322545	0.566810
C	0.947106	-0.626582	0.823849
H	-0.887436	-3.424672	0.533990
H	-1.343055	-2.032123	1.553463
H	0.842096	-2.123694	-0.664491
C	2.270897	-0.138034	0.340957
C	0.321421	0.130942	1.977424
H	1.054368	0.299809	2.785331
H	-0.546432	-0.393312	2.402689
H	-0.037849	1.123220	1.648532
C	4.785735	0.811231	-0.593743
C	3.874653	1.692030	0.009203
C	2.638122	1.222744	0.476053
C	3.212822	-1.014537	-0.252213
C	4.448049	-0.547000	-0.718153
H	5.753986	1.175750	-0.952172
H	4.126023	2.752618	0.118077
H	1.940263	1.928968	0.936154
H	2.982405	-2.082783	-0.318187
H	5.158246	-1.250507	-1.165908
S	-2.309681	-1.982259	-0.706663
C	-2.548489	-0.208576	-0.479154
C	-2.963851	2.556705	-0.147315
C	-3.792639	1.636985	0.516708
C	-3.585763	0.257904	0.355098
C	-1.725716	0.716651	-1.154675
C	-1.932080	2.095143	-0.981824
H	-3.126605	3.632213	-0.020386
H	-4.602437	1.992838	1.162567
H	-4.224478	-0.466691	0.870063
H	-0.931602	0.348369	-1.809770
H	-1.289991	2.809231	-1.508453

SCF(BP86) = -629.537694444
H 0K = -629.273281
H 298K = -629.257084
G 298K = -629.319655
Solvent Correction(CH3Cl) = -0.00448839
BP86-D3 Correction = -0.03546523
Lowest frequencies = 17.7798 cm-1, 25.7603 cm-1

(*Z*)-32

33

C	1.015877	1.849819	0.488038
C	-0.124956	0.901686	0.704260
C	2.310286	1.527886	0.223459
H	0.223246	-0.132475	0.851000
H	-0.734293	1.215686	1.569339
H	0.762135	2.914169	0.578679
C	2.791441	0.128134	0.026235
C	3.353974	2.622915	0.122961
H	4.138590	2.510438	0.893396
H	2.902784	3.620198	0.248000
H	3.868826	2.589396	-0.853998
C	3.740170	-2.517809	-0.370320
C	2.585018	-2.073416	-1.032821
C	2.116529	-0.764984	-0.840018
C	3.962377	-0.331268	0.674744
C	4.426805	-1.640329	0.484926
H	4.106933	-3.538126	-0.523587
H	2.048025	-2.743968	-1.712194
H	1.227578	-0.419980	-1.380003
H	4.504906	0.337388	1.351433
H	5.329254	-1.976123	1.006979
S	-1.250888	0.969723	-0.785938
C	-2.757288	0.185910	-0.201750
C	-5.203666	-1.015888	0.543660
C	-4.043161	-1.240463	1.299221
C	-2.821764	-0.653028	0.930104
C	-3.928420	0.417170	-0.958668
C	-5.137154	-0.187494	-0.589979
H	-6.151430	-1.479465	0.834855
H	-4.079225	-1.886595	2.183089
H	-1.925353	-0.860021	1.521211
H	-3.888332	1.079184	-1.830648
H	-6.035851	0.001691	-1.186865

SCF(BP86) = -629.537381861
H 0K = -629.273004
H 298K = -629.256796
G 298K = -629.319517
Solvent Correction(CH3Cl) = -0.00442160
BP86-D3 Correction = -0.03293397
Lowest frequencies = 15.2644 cm-1, 26.3728 cm-1

33

33

S	0.342074	-0.794976	-0.881663
C	-0.616451	0.367411	0.336215
C	-0.263367	1.769168	-0.093529
C	2.066802	-0.508201	-0.445737
C	2.720325	-1.378935	0.453366
C	4.082408	-1.202170	0.743627
C	4.801619	-0.157242	0.140408
C	4.157054	0.709980	-0.757114
C	2.795654	0.537288	-1.052204
H	2.154326	-2.195544	0.912040
H	4.582544	-1.884681	1.438987
H	5.865059	-0.022884	0.364637
H	4.716688	1.521492	-1.234412
H	2.289144	1.202364	-1.756772
C	-2.097713	0.040308	0.089454
C	-0.229183	0.038253	1.786866
H	-0.584334	2.022446	-1.112638
C	0.420667	2.678682	0.624944
H	0.653934	3.663045	0.207457
H	0.773358	2.483102	1.642038
C	-3.047151	1.072942	-0.057021
C	-4.410762	0.780244	-0.219961
C	-4.851045	-0.551422	-0.240159
C	-3.916708	-1.588710	-0.086793
C	-2.555551	-1.296078	0.080112
H	-2.716773	2.116050	-0.026750
H	-5.129454	1.599358	-0.330422
H	-5.913815	-0.780292	-0.371487
H	-4.247464	-2.632760	-0.097536
H	-1.834684	-2.112453	0.195151
H	-0.768282	0.711266	2.476920
H	-0.506925	-0.998023	2.032787
H	0.853909	0.158020	1.951761

SCF(BP86) = -629.524159434
H 0K = -629.260450
H 298K = -629.244316
G 298K = -629.305107
Solvent Correction(CH3Cl) = -0.00416506
BP86-D3 Correction = -0.03879392
Lowest frequencies = 24.4972 cm-1, 37.0578 cm-1

From 11
11

29

O	1.504597	0.441237	1.411518
C	1.325225	0.401941	-0.016725
C	1.824956	1.773054	-0.517618
H	1.681112	1.870777	-1.606628
H	1.287384	2.591739	-0.014030
H	2.898393	1.875051	-0.294220
C	2.186531	-0.684130	-0.653453
C	-0.183311	0.156178	-0.384643
C	-1.123132	1.262744	0.144467
H	-0.239162	0.156713	-1.494693
C	-2.600189	0.994610	-0.209529
H	-0.999725	1.323864	1.242834
H	-0.827129	2.246969	-0.259060
C	-3.067348	-0.390809	0.271435
H	-3.238761	1.788244	0.220241
H	-2.726331	1.055429	-1.309438
C	-2.143736	-1.497026	-0.269015
H	-3.055079	-0.416524	1.379508
H	-4.113289	-0.574679	-0.035677
C	-0.673078	-1.228517	0.103788
H	-2.456551	-2.486309	0.112734
H	-2.235863	-1.543651	-1.372716
H	-0.583616	-1.270453	1.210025
H	-0.020963	-2.026838	-0.294384
C	3.151133	-1.369306	-0.018811
H	1.986982	-0.878803	-1.717302
H	3.750637	-2.128205	-0.530713
H	3.382022	-1.170757	1.033386
H	1.290857	-0.454903	1.738610

SCF(BP86) = -467.106351444
H 0K = -466.850026
H 298K = -466.837967
G 298K = -466.887013
Solvent Correction(CH3Cl) = -0.00259353
BP86-D3 Correction = -0.03423157
Lowest frequencies = 59.6954 cm-1, 96.4352 cm-1

(*E*)-34

39

S	-1.811013	0.807775	0.177585
C	-0.874902	-0.739545	-0.295435
C	0.546955	-0.361311	-0.591298
C	1.624757	-0.528913	0.215067
H	-0.959777	-1.474976	0.519289
H	-1.368294	-1.146314	-1.194429
C	-3.531406	0.321046	0.012739
C	-3.974098	-1.017375	-0.034606
C	-5.345537	-1.300816	-0.145377
C	-6.289861	-0.264742	-0.194335
C	-5.850010	1.069245	-0.137312
C	-4.484045	1.364301	-0.042602
H	-3.258825	-1.842645	0.022201
H	-5.673222	-2.345417	-0.185275
H	-7.357227	-0.492309	-0.277408
H	-6.574502	1.889701	-0.179496
H	-4.147368	2.406626	-0.020520
H	0.702072	0.122334	-1.565229
C	2.997705	-0.078892	-0.274027
C	1.550523	-1.153415	1.591641
H	2.060157	-0.521296	2.340236
H	0.516697	-1.300656	1.938090
H	2.058404	-2.135339	1.614300
C	4.042569	-1.225781	-0.288742
H	2.873780	0.261997	-1.322599
C	5.396179	-0.751357	-0.852908
H	4.194398	-1.597817	0.743725
H	3.654432	-2.076716	-0.878547
C	5.938533	0.461328	-0.072730
H	6.126318	-1.581178	-0.833196
H	5.268366	-0.472237	-1.917930
C	4.907806	1.605620	-0.033946
H	6.174771	0.149544	0.964506
H	6.887372	0.812612	-0.517823
C	3.552966	1.130276	0.527297
H	5.290742	2.449991	0.568036
H	4.758233	1.996026	-1.060436
H	3.684549	0.841680	1.588860
H	2.816330	1.953976	0.511734

SCF(BP86) = -633.163016803
H 0K = -632.830815
H 298K = -632.813080
G 298K = -632.879911
Solvent Correction(CH3Cl) = -0.00384975
BP86-D3 Correction = -0.04178062
Lowest frequencies = 12.1429 cm-1, 20.8390 cm-1

(*Z*)-34

39

S	1.530377	0.915083	0.703003
C	0.504141	0.821947	-0.856480
C	-0.630931	1.796798	-0.732210
C	-1.917651	1.525275	-0.396663
H	1.168617	1.098977	-1.692197
H	0.164611	-0.215193	-1.001125
C	3.063550	0.100872	0.244519
C	4.174575	0.315151	1.092288
C	5.398466	-0.311273	0.824743
C	5.542464	-1.147074	-0.296382
C	4.442868	-1.355709	-1.141967
C	3.206196	-0.745042	-0.875182
H	4.075881	0.981905	1.956010
H	6.249066	-0.134074	1.491750
H	6.502424	-1.628359	-0.508044
H	4.538862	-2.006779	-2.017786
H	2.358258	-0.938736	-1.538048
H	-0.356411	2.844644	-0.914075
C	-2.427998	0.121983	-0.093959
C	-2.920227	2.659087	-0.328953
H	-3.802011	2.464470	-0.966570
H	-3.302766	2.799663	0.698564
H	-2.471006	3.611323	-0.653396
C	-3.346424	-0.425874	-1.221821
H	-1.557110	-0.559080	-0.030552
C	-3.809470	-1.866016	-0.925386
H	-4.235427	0.227651	-1.321803
H	-2.813576	-0.382084	-2.189327
C	-4.506884	-1.967468	0.444478
H	-4.481777	-2.219590	-1.728608
H	-2.929002	-2.539339	-0.935543
C	-3.609745	-1.416713	1.569277
H	-5.450675	-1.386665	0.415559
H	-4.790128	-3.014951	0.654943
C	-3.156255	0.026002	1.273188
H	-4.139804	-1.454197	2.538564
H	-2.716908	-2.064565	1.675875
H	-4.045316	0.686640	1.265145
H	-2.491882	0.395337	2.075687

SCF(BP86) = -633.162463067
H 0K = -632.830091
H 298K = -632.812488
G 298K = -632.879104
Solvent Correction(CH3Cl) = -0.00383330
BP86-D3 Correction = -0.04255061
Lowest frequencies = 10.8615 cm-1, 18.5658 cm-1

35

39

S	0.577784	-0.882930	-0.881576
C	-0.443375	0.365481	0.184458
C	-0.042449	1.732992	-0.309911
C	2.279184	-0.544453	-0.398326
C	2.892656	-1.321404	0.608360
C	4.239151	-1.107740	0.943586
C	4.983455	-0.120511	0.277020
C	4.379111	0.652401	-0.728071
C	3.032954	0.443822	-1.066928
H	2.307882	-2.095419	1.114795
H	4.707841	-1.717519	1.723371
H	6.035043	0.041820	0.536043
H	4.958111	1.418742	-1.254491
H	2.557778	1.036504	-1.853115
C	-1.930012	0.080342	-0.223370
C	-0.154563	0.130663	1.670443
H	-0.340238	1.938297	-1.348340
C	0.637841	2.673900	0.371302
H	0.890571	3.631966	-0.093512
H	0.967821	2.530291	1.404643
H	-0.714457	0.851480	2.292413
H	-0.441557	-0.888153	1.972080
H	0.917680	0.256863	1.889772
C	-2.899742	1.125379	0.385607
H	-1.969973	0.205643	-1.327332
C	-4.337542	0.929198	-0.135308
H	-2.898635	1.023598	1.488994
H	-2.546379	2.148238	0.164788
C	-4.848768	-0.498404	0.133227
H	-5.010229	1.674601	0.327359
H	-4.356997	1.124578	-1.226194
C	-3.877642	-1.550287	-0.434387
H	-4.947482	-0.648876	1.227028
H	-5.859239	-0.633710	-0.294257
C	-2.443267	-1.344004	0.094099
H	-4.225581	-2.569892	-0.187205
H	-3.869967	-1.482255	-1.540650
H	-2.441535	-1.498993	1.190942
H	-1.766961	-2.105177	-0.332721

SCF(BP86) = -633.152937312
H 0K = -632.821165
H 298K = -632.803730
G 298K = -632.866767
Solvent Correction(CH3Cl) = -0.00306479
BP86-D3 Correction = -0.05047592
Lowest frequencies = 23.0520 cm-1, 47.2347 cm-1

5. Reactions of Substrate **17**

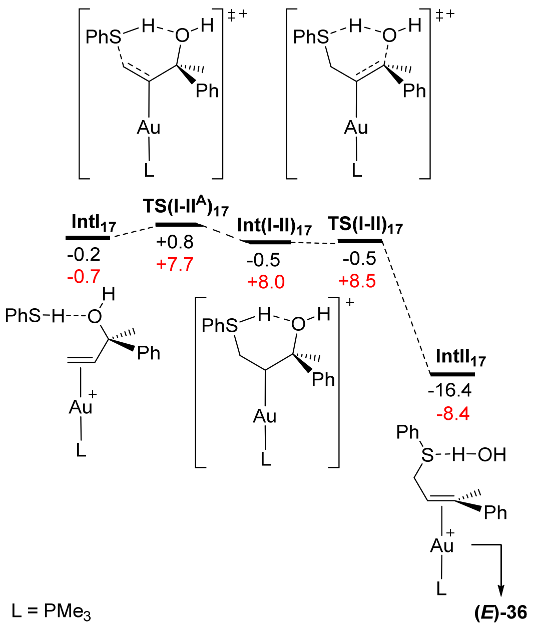

Figure S10. First S_N2' step via *anti* attack of PhSH to give *E*-**36**. Gas phase SCF energies (BP86) are shown in black with free energies at the BP86-D3($CHCl_3$) in red. All energies are in kcal/mol and are quote relative to **5'** and the separated reactants set to zero

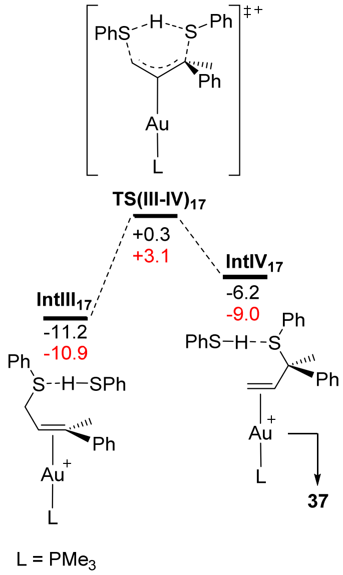

Figure S10. Second S_N2' step via *anti* attack of PhSH to give **37**. Gas phase SCF energies (BP86) are shown in black with free energies at the BP86-D3($CHCl_3$) in red. All energies are in kcal/mol and are quote relative to **5'** and the separated reactants set to zero

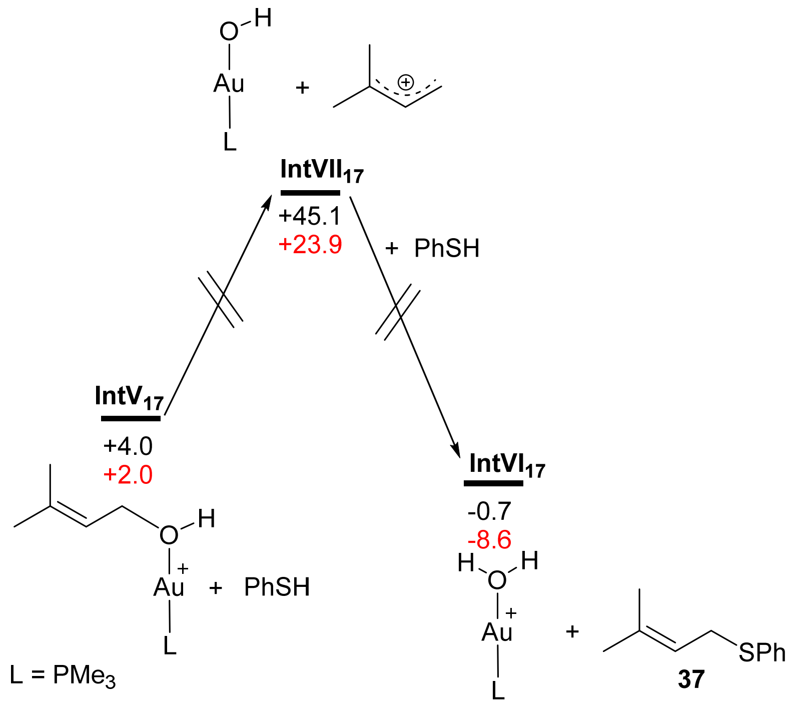

Figure S11. Alternative routes to the formation of **37** via either C–O bond cleavage and formation of an allylic cation or direct SN2 attack at a *O*-bound form of **17**. Gas phase SCF energies (BP86) are shown in black with free energies at the BP86-D3($CHCl_3$) in red. All energies are in kcal/mol and are quoted relative to **5'** and the separated reactants set to zero.

Table S5. Computed relative energies (kcal/mol) for the reactions **17** with PhSH. Energies are reported as ΔE (gas-phase SCF energies), ΔH_{298} (zero-point energy corrected energies at 298.15 K), ΔG (free energies at 298.15 K and 1 atm), ΔG_{disp} (including a correction for dispersion effects using Grimme's D3 parameter set) and $\Delta G_{CHCl3+disp}$ including an additional correction for $CHCl_3$ solvent via the PCM approach.

	**ΔE**	**ΔH_{298}**	**ΔG**	**ΔG_{disp}**	**$\Delta G_{CHCl3+disp}$**
**5'**	0.0	0.0	0.0	0.0	0.0
***IntI$_{17}$ (anti, pro-*E*)**	+4.3	+4.3	+5.4	-1.6	-2.4
***IntI$_{17}$ (anti, pro-*Z*)**	+5.8	+5.7	+6.7	+1.4	+0.1
**IntI$_{17}$ (anti, pro-*E*)**	-0.2	+1.7	+11.6	-2.1	-0.7
**IntI$_{17}$ (anti, pro-*Z*)**	+1.2	+3.2	+14.1	+0.4	+2.4
**IntV$_{17}$ (pro-*E*)**	+4.0	+4.1	+4.6	+1.3	+2.0
**#IntVII$_{17}$ (pro-*E*)**	+20.9	+19.9	+18.5	+18.3	+21.8
**IntVII$_{17}$ (pro-*E*)**	+45.1	+42.3	+31.9	+35.3	+23.9
**IntVI$_{17}$ (pro-*E*)**	-0.7	+0.8	+0.2	-1.6	-8.6
**TS(I-II^A)$_{17}$ (anti, pro-*E*)**	+0.8	+2.2	+14.6	+2.6	+7.7
**TS(I-II)$_{17}$ (anti, pro-*Z*)**	+1.9	+3.4	+17.2	+3.0	+7.8
**Int(I-II)$_{17}$ (anti, pro-*E*)**	-0.5	+0.9	+14.2	+2.3	+8.0
**TS(I-II)$_{17}$ (anti, pro-*E*)**	-0.5	+0.0	+14.6	+2.8	+8.5
**IntII$_{17}$ (anti, pro-*E*)**	-16.4	-13.0	-2.2	-12.1	-8.4
**IntII$_{17}$ (anti, pro-*Z*)**	-11.9	-8.5	+0.8	-10.4	-6.9
***IntII$_{17}$ (anti, pro-*E*)**	-6.6	-5.5	-4.1	-10.2	-10.2
***IntII$_{17}$ (anti, pro-*Z*)**	-3.6	-2.4	-1.2	-8.2	-7.9
**IntIII$_{17}$ (anti, pro-*S*)**	-11.2	-8.2	+0.6	-13.8	-10.9
**TS(III-IV)$_{17}$ (anti, pro-*S*)**	+0.3	+2.2	+15.7	-2.2	+3.1
**IntIV$_{17}$ (anti, pro-*S*)**	-6.2	-3.5	+6.2	-11.6	-9.0
***IntIV$_{17}$ (anti, pro-*S*)**	-2.4	-1.6	+0.4	-8.4	-8.0

*Complex computed in the absence of PhSH nucleophile

#Computed as an ion pair

17

20

C	2.155265	-0.341177	0.626776
C	3.103817	-1.258699	0.372373
C	1.334803	0.350882	-0.442226
O	1.586302	1.766145	-0.419523
H	2.556493	1.866001	-0.399103
H	3.327303	-1.579550	-0.652502
H	3.685290	-1.728255	1.172164
H	1.936926	-0.027614	1.656630
C	-0.158987	0.125043	-0.227481
H	1.615676	-0.079134	-1.430525
C	-2.908577	-0.350321	0.192935
C	-2.369707	0.926825	0.411625
C	-1.000759	1.165444	0.204539
C	-0.704423	-1.155785	-0.445407
C	-2.070197	-1.393284	-0.237227
H	-3.976212	-0.533713	0.353981
H	-3.017514	1.745416	0.743876
H	-0.573685	2.158755	0.364089
H	-0.050335	-1.971521	-0.775495
H	-2.481772	-2.393108	-0.412384

SCF(BP86) = -424.161108345
H 0K = -423.999863
H 298K = -423.990369
G 298K = -424.035497
Solvent Correction(CH3Cl) = -0.00398490
BP86-D3 Correction = -0.01648625
Lowest frequencies = 20.1171 cm-1, 82.9163 cm-1

(E)-36

30

C	0.309555	-1.812563	0.277514
C	-1.018706	-2.268910	0.778544
C	1.004854	-0.786196	0.826487
H	-1.067731	-3.367089	0.886627
H	-1.284300	-1.809283	1.743940
H	0.695573	-2.342016	-0.603114
C	2.307845	-0.255009	0.399898
H	0.563611	-0.282016	1.698675
C	4.819759	0.850491	-0.332255
C	4.116555	1.364845	0.768256
C	2.877261	0.817792	1.127657
C	3.032387	-0.761139	-0.708142
C	4.269510	-0.215215	-1.067591
H	5.788354	1.274309	-0.616759
H	4.533660	2.194496	1.348910
H	2.331270	1.223480	1.987482
H	2.622546	-1.590577	-1.293795
H	4.810905	-0.623036	-1.927983
S	-2.423764	-1.925496	-0.435380
C	-2.553648	-0.130526	-0.302332
C	-2.823817	2.666472	-0.126978
C	-3.645136	1.831226	0.648577
C	-3.511087	0.436442	0.564705
C	-1.734562	0.710119	-1.083847
C	-1.869584	2.104762	-0.990735
H	-2.930541	3.754363	-0.061136
H	-4.393715	2.265323	1.319947
H	-4.146972	-0.222863	1.163975
H	-1.000177	0.264733	-1.760390
H	-1.229385	2.752562	-1.598843

SCF(BP86) = -590.225603601
H 0K = -589.988650
H 298K = -589.973872
G 298K = -590.034045
Solvent Correction(CH3Cl) = -0.00471764
BP86-D3 Correction = -0.02828956
Lowest frequencies = 17.9078 cm-1, 19.5598 cm-1

(Z)-36

30

S	-1.095522	0.967493	-0.802086
C	-0.061284	1.104330	0.748228
C	1.042468	2.092421	0.513630
C	-2.590095	0.154421	-0.226204
C	-3.724760	0.248121	-1.063651
C	-4.919611	-0.389486	-0.705228
C	-5.009068	-1.113709	0.496091
C	-3.885074	-1.201190	1.330964
C	-2.676648	-0.580075	0.974444
H	-3.668225	0.829258	-1.990605
H	-5.790234	-0.307645	-1.364729
H	-5.946657	-1.602829	0.778191
H	-3.939254	-1.765014	2.268559
H	-1.807251	-0.680029	1.630219
H	-0.735495	1.459460	1.545696
H	0.332660	0.109917	1.012459
H	0.756577	3.147203	0.606336
C	2.341813	1.815101	0.235322
C	2.995212	0.507354	0.040536
C	4.352422	0.365777	0.421570
C	5.022970	-0.854879	0.273329
C	4.356320	-1.961578	-0.278944
C	3.019737	-1.830339	-0.690534
C	2.344576	-0.610785	-0.538423
H	4.876804	1.227152	0.851488
H	6.069605	-0.941964	0.583825
H	4.880275	-2.915039	-0.402859
H	2.501061	-2.679687	-1.147854
H	1.320693	-0.509108	-0.914568
H	3.023931	2.675550	0.190443

SCF(BP86) = -590.221484159
H 0K = -589.983933
H 298K = -589.969433
G 298K = -590.028306
Solvent Correction(CH3Cl) = -0.004457122
BP86-D3 Correction = -0.02859682
Lowest frequencies = 16.7045 cm-1, 27.1694 cm-1

37

30

S	-0.288165	-1.718587	-0.944034
C	1.080509	-1.219495	0.284008
C	2.251612	-2.119787	-0.020522
C	-1.647957	-0.654722	-0.413370
C	-1.815879	0.629796	-0.970299
C	-2.902215	1.427768	-0.577630
C	-3.825944	0.950095	0.366346
C	-3.663110	-0.330463	0.920614
C	-2.578112	-1.132515	0.533520
H	-1.091476	0.994712	-1.703670
H	-3.027442	2.424770	-1.013212
H	-4.674545	1.573551	0.667109
H	-4.384178	-0.708653	1.653177
H	-2.446024	-2.134589	0.953910
C	1.430184	0.255695	0.203841
H	0.684641	-1.454315	1.286146
H	2.787136	-1.914111	-0.956700
C	2.660040	-3.120675	0.777764
H	3.522032	-3.743037	0.518816
H	2.148441	-3.350024	1.719623
C	1.288785	1.069253	1.344903
C	1.639993	2.428676	1.307331
C	2.139689	2.993414	0.124509
C	2.280324	2.191601	-1.022073
C	1.924909	0.836468	-0.984081
H	0.901759	0.631124	2.272154
H	1.520415	3.045444	2.204383
H	2.414792	4.052820	0.092396
H	2.665203	2.625870	-1.951051
H	2.012905	0.223177	-1.887710

SCF(BP86) = -590.212291942
H 0K = -589.976037
H 298K = -589.961059
G 298K = -590.021123
Solvent Correction(CH3Cl) = -0.004775303
BP86-D3 Correction = -0.03207694
Lowest frequencies = 15.1620 cm-1, 29.8354 cm-1

***IntI$_{17}$ (anti, pro-*E*)**

34

P	2.339069	0.732656	-0.054819
C	1.931289	2.214435	-1.084726
C	2.766705	1.380427	1.624687
Au	0.584370	-0.815855	-0.025877
C	-1.492816	-1.854335	-0.340688
C	-0.597362	-2.745427	0.228440
C	-2.566277	-1.082749	0.420009
O	-3.777382	-1.582069	-0.143459
H	-4.511310	-1.035495	0.200434
H	-0.016744	-3.433190	-0.397407
H	-0.618421	-2.953737	1.305189
H	-1.598987	-1.813584	-1.434360
C	3.909903	0.041170	-0.746382
C	-2.396566	0.427146	0.240806
H	-2.480635	-1.326943	1.501941
C	-2.106034	3.212608	-0.089728
C	-2.612098	2.426295	-1.140790
C	-2.759614	1.040590	-0.977486
C	-1.893559	1.220654	1.293549
C	-1.750167	2.609144	1.128985
H	-2.015444	4.296690	-0.211684
H	-2.909996	2.897231	-2.082922
H	-3.188505	0.430263	-1.779477
H	-1.650308	0.754577	2.256243
H	-1.393165	3.222956	1.962479
H	1.004405	2.673526	-0.707577
H	4.691752	0.818053	-0.747862
H	1.883115	1.869357	2.061647
H	3.590760	2.108130	1.546193
H	3.735370	-0.307523	-1.775560
H	1.775340	1.900806	-2.128112
H	2.757705	2.942200	-1.037644
H	4.238712	-0.810354	-0.131426
H	3.073744	0.546551	2.274071

SCF(BP86) = -686.219883815
H 0K = -685.945093
H 298K = -685.925361
G 298K = -685.996881
Solvent Correction(CH3Cl) = -0.05196535
BP86-D3 Correction = -0.04247116
Lowest frequencies = 12.0370 cm-1, 21.6263 cm-1

*IntI$_{17}$ (anti, pro-*Z*)

34

C	2.801723	2.286949	-0.744798
C	2.968709	1.112992	1.962020
Au	0.765232	-0.513606	-0.239356
C	-1.128573	-1.866959	0.014548
C	-0.960013	-1.527318	-1.317836
C	-2.110902	-1.207621	0.987706
O	-3.065982	-2.252731	1.191012
H	-3.661323	-1.969513	1.912297
H	-1.531346	-0.700474	-1.757833
H	-0.439558	-2.207176	-2.002684
H	-0.711880	-2.814848	0.383680
C	4.254653	-0.250967	-0.319601
C	-2.720397	0.095643	0.490679
H	-1.546720	-0.998438	1.926266
C	-3.813215	2.510984	-0.466721
C	-4.344339	1.280245	-0.889439
C	-3.802631	0.076222	-0.412809
C	-2.203906	1.332838	0.924547
C	-2.744897	2.537090	0.444999
H	-4.243246	3.448148	-0.833360
H	-5.188855	1.258218	-1.585337
H	-4.227201	-0.886212	-0.716739
H	-1.394213	1.352559	1.665539
H	-2.349827	3.494351	0.799991
P	2.736441	0.683201	0.176296
H	3.745470	2.809285	-0.518282
H	1.949973	2.916864	-0.446834
H	4.308017	-1.192678	0.247579
H	4.208377	-0.483350	-1.394417
H	2.128507	1.736140	2.303627
H	3.913014	1.666889	2.090088
H	2.740336	2.091872	-1.826262
H	5.151238	0.355667	-0.112450
H	2.999919	0.190833	2.561934

SCF(BP86) = -686.217594299
H 0K = -685.942761
H 298K = -685.923099
G 298K = -685.994804
Solvent Correction(CH3Cl) = -0.05285743
BP86-D3 Correction = -0.03978676
Lowest frequencies = 11.2424 cm-1, 14.1264 cm-1

$IntI_{17}$ (anti, pro-*E*)

47

P	3.066819	-1.567464	0.366417
C	4.389152	-0.276043	0.462206
C	2.860653	-2.212691	2.088933
Au	1.083675	-0.724548	-0.552846
C	-0.547057	0.654095	-1.430575
C	-1.013582	-0.651659	-1.485191
S	-4.358424	-0.006529	-1.795239
C	-0.978628	1.658901	-0.368069
C	-4.316389	-0.458672	-0.053469
O	-1.935911	2.483644	-1.060969
H	-2.013378	3.321908	-0.562950
H	-0.882087	-1.258323	-2.388446
H	-1.692683	-1.040523	-0.716796
H	-0.048680	1.090542	-2.307829
C	-4.304881	-1.268453	2.650338
C	-4.148244	0.087089	2.316144
C	-4.159817	0.497331	0.973267
C	-4.470623	-1.821135	0.280558
C	-4.471317	-2.216811	1.627577
H	-4.311237	-1.580834	3.698888
H	-4.037841	0.838319	3.105485
H	-4.067303	1.558337	0.719631
H	-4.600098	-2.567211	-0.510815
H	-4.605523	-3.274805	1.875281
C	3.789132	-2.963449	-0.610900
H	-3.728366	1.196190	-1.643441
C	0.202626	2.454876	0.172573
H	-1.470216	1.112047	0.464162
C	2.393540	3.919903	1.175844
C	1.926073	4.155770	-0.129360
C	0.835965	3.428546	-0.629968
C	0.670900	2.228419	1.483827
C	1.763356	2.957911	1.983144
H	3.234762	4.499492	1.568773
H	2.405009	4.916356	-0.753914
H	0.456646	3.630044	-1.637912
H	0.157632	1.500059	2.123231
H	2.103137	2.795924	3.011349
H	4.019433	0.580461	1.046275
H	4.725089	-3.305525	-0.140000
H	2.498341	-1.404336	2.741802
H	3.825677	-2.587618	2.467004
H	3.995855	-2.623806	-1.637159
H	4.636469	0.067333	-0.553865
H	5.289942	-0.695031	0.939409
H	3.070388	-3.796138	-0.648328
H	2.123331	-3.029834	2.089062

SCF(BP86) = -928.693686455
H 0K = -928.321215
H 298K = -928.293090
G 298K = -928.388035
Solvent Correction(CH3Cl) = -0.05184365
BP86-D3 Correction = -0.06194841
Lowest frequencies = 8.9278 cm-1, 10.1876 cm-1

IntI$_{17}$ (anti, pro-*Z*)

47

C	4.340368	-1.137542	-1.262051
C	3.935877	-2.118460	1.491639
Au	1.086430	-0.717202	-0.010531
C	-0.820960	0.381084	0.576107
C	-0.868881	0.268028	-0.810946
S	-3.738166	1.310318	-1.382761
C	-0.527381	1.696020	1.310613
C	-4.372775	-0.099963	-0.463656
O	-1.827702	2.318907	1.384698
H	-1.743344	3.114354	1.946845
H	-0.510539	1.072450	-1.462848
H	-1.364055	-0.584348	-1.285672
H	-1.375295	-0.359579	1.169804
C	-5.386607	-2.361889	0.870187
C	-5.442291	-2.283068	-0.531848
C	-4.938207	-1.159675	-1.202959
C	-4.316342	-0.170575	0.944336
C	-4.824980	-1.303478	1.601442
H	-5.788878	-3.237350	1.388540
H	-5.887338	-3.098442	-1.111002
H	-4.992886	-1.102223	-2.295338
H	-3.891397	0.658596	1.519084
H	-4.791473	-1.347852	2.695173
C	2.855607	-3.644969	-0.787527
C	0.521722	2.583352	0.652572
H	-0.176381	1.434325	2.330851
C	2.501048	4.179411	-0.572620
C	1.166148	4.255873	-1.003572
C	0.179580	3.465212	-0.392078
C	1.860493	2.526834	1.093928
C	2.846479	3.318087	0.482481
H	3.265512	4.806326	-1.041923
H	0.888929	4.942355	-1.809763
H	-0.866240	3.548550	-0.704896
H	2.123884	1.883561	1.943154
H	3.877996	3.284618	0.848120
H	-3.258063	1.989469	-0.299661
P	3.090931	-1.924445	-0.145315
H	5.263331	-1.739724	-1.278526
H	4.565585	-0.123006	-0.900232
H	2.170007	-4.192938	-0.123417
H	2.416896	-3.602691	-1.796041
H	4.177411	-1.126016	1.901251
H	4.863712	-2.700067	1.366602
H	3.927965	-1.068112	-2.280146
H	3.825312	-4.167188	-0.829272
H	3.266296	-2.641491	2.191249

SCF(BP86) = -928.691354497
H 0K = -928.318529
H 298K = -928.290667
G 298K = -928.384166
Solvent Correction(CH3Cl) = -0.05087661
BP86-D3 Correction = -0.06179849
Lowest frequencies = 6.4290 cm-1, 12.2773 cm-1

IntV$_{17}$ (pro-*E*)

34

```
P   3.32077 -0.08337  0.03621
C   3.96689  0.61386  1.62237
C   4.16039 -1.71573 -0.18501
C   3.96920  1.01106 -1.30589
Au  1.05044 -0.25645  0.00348
C  -1.66301  1.79600 -0.61201
C  -1.18782  2.96794 -0.15208
C  -2.03040  0.65638  0.29215
O  -1.09300 -0.48334 -0.07949
H  -1.45001 -1.30811  0.31424
H  -1.02620  3.14371  0.91795
H  -1.85023  1.64868 -1.68286
C  -3.44292  0.14012  0.15160
H  -1.81961  0.91996  1.34333
H   3.54773  1.61928  1.77892
H   5.06671  0.67480  1.58176
H   3.66413 -0.03482  2.45818
H   3.54936  2.02170 -1.19009
H   3.66755  0.60828 -2.28459
H   5.06893  1.05891 -1.24873
H   3.86119 -2.15390 -1.14915
H   3.85998 -2.39605  0.62612
H   5.25374 -1.57733 -0.16526
C  -6.08429 -0.80698 -0.07253
C  -5.24516 -0.79160 -1.20070
C  -3.93135 -0.31703 -1.09375
C  -4.28744  0.11256  1.28085
C  -5.60541 -0.35402  1.16722
H  -7.11181 -1.17286 -0.16124
H  -5.61819 -1.14593 -2.16646
H  -3.28245 -0.30824 -1.97606
H  -3.91413  0.46743  2.24851
H  -6.25629 -0.36604  2.04662
H  -0.97462  3.80217 -0.82670
```

SCF(BP86) = -686.220467543
H 0K = -685.945490
H 298K = -685.925679
G 298K = -685.998241
Solvent Correction(CH3Cl) = -0.04966730
BP86-D3 Correction = -0.03644421
Lowest frequencies = 10.4876 cm-1, 17.6315 cm-1

IntVII$_{17}$ (pro-*E*)

16

P	1.503762	-0.000059	-0.000004
C	2.302146	-0.838285	-1.456703
C	2.269239	1.694473	-0.003590
C	2.302214	-0.832063	1.460237
Au	-0.765870	-0.002114	0.000005
O	-2.764466	-0.108781	0.000081
H	-3.097163	0.810697	-0.000439
H	1.977590	-1.889847	-1.486148
H	3.401788	-0.791769	-1.388900
H	1.965590	-0.345199	-2.381494
H	1.977845	-1.883549	1.494086
H	1.965531	-0.335153	2.382933
H	3.401848	-0.785616	1.392275
H	1.929337	2.242775	0.888335
H	1.929046	2.239099	-0.897654
H	3.369991	1.631970	-0.003649

SCF(BP86) = -338.074767856
H 0K = -337.950612
H 298K = -337.939568
G 298K = -337.988751
Solvent Correction(CH3Cl) = -0.01252181
BP86-D3 Correction = -0.01355728
Lowest frequencies = 42.5807 cm-1, 91.7053 cm-1

Organic cation fragment for IntVII$_{17}$ (pro-*E*)

18

```
C   2.49797  0.41886  0.00004
C   3.78763 -0.04226  0.00000
C   1.40966 -0.50074 -0.00003
H   4.01156 -1.11524 -0.00006
H   2.31116  1.49741  0.00012
C   0.02620 -0.20749 -0.00005
H   1.68125 -1.56629 -0.00010
C  -2.74955  0.24487  0.00007
C  -1.86266  1.34868  0.00000
C  -0.49080  1.13446 -0.00006
C  -0.89634 -1.31113 -0.00003
C  -2.26755 -1.08279  0.00004
H  -3.82952  0.42522  0.00013
H  -2.26129  2.36679  0.00000
H   0.19181  1.98832 -0.00013
H  -0.50356 -2.33335 -0.00005
H  -2.96914 -1.92123  0.00008
H   4.64027  0.64365  0.00005
```

SCF(BP86) = -348.080138071
H 0K = -347.933245
H 298K = -347.925183
G 298K = -347.965992
Solvent Correction(CH3Cl) = -0.05637435
BP86-D3 Correction = -0.01218360
Lowest frequencies = 83.9377 cm-1, 140.5147 cm-1

#IntVII$_{17}$ (pro-*E*)

34

P	-3.418014	-0.252883	0.468360
C	-3.721168	-1.880252	1.305563
C	-3.916135	1.016169	1.726734
Au	-1.306931	0.002338	-0.348860
C	3.528543	2.623868	0.316143
C	2.609023	3.621237	0.141032
C	3.164367	1.281311	-0.004053
O	0.605240	0.216035	-1.049277
H	0.529915	0.243068	-2.023868
H	1.606293	3.395461	-0.239388
H	2.840658	4.665773	0.371570
H	4.526994	2.866316	0.697216
C	-4.727993	-0.149989	-0.839817
C	3.978626	0.126624	0.106692
H	2.131611	1.100343	-0.388514
C	5.443512	-2.263527	0.256132
C	6.055199	-1.045088	0.640969
C	5.337474	0.141732	0.570005
C	3.375539	-1.120739	-0.279053
C	4.110706	-2.301775	-0.201499
H	7.091011	-1.044403	0.991733
H	5.809903	1.082750	0.865946
H	2.331160	-1.091113	-0.626706
H	3.659506	-3.254393	-0.493130
H	-3.846999	2.018558	1.277477
H	-3.230431	0.965457	2.586279
H	-4.948385	0.835377	2.068285
H	-4.553059	-0.934988	-1.591206
H	-4.670471	0.831124	-1.335262
H	-5.727617	-0.281258	-0.394591
H	-3.526381	-2.695843	0.592633
H	-4.762081	-1.942329	1.662680
H	-3.032566	-1.984748	2.157900
H	6.020623	-3.192329	0.316598

SCF(BP86) = -686.193546456
H 0K = -685.921244
H 298K = -685.900422
G 298K = -685.976058
Solvent Correction(CH3Cl) = -0.04522441
BP86-D3 Correction = -0.03145088
Lowest frequencies = 12.4695 cm-1, 12.9890 cm-1

TS(I-II^{A})$_{17}$ (anti, pro-*E*)

47

P	3.177007	-1.890514	0.348117
C	4.723270	-0.902911	0.087376
C	3.209452	-2.375501	2.137177
Au	1.245020	-0.729861	-0.292091
C	-0.437215	0.505347	-0.955106
C	-1.320251	-0.614040	-0.929661
S	-3.583290	0.087350	-1.707998
C	-0.669497	1.666861	0.011875
C	-4.642576	-0.289541	-0.304929
O	-1.769364	2.429947	-0.568679
H	-1.823565	3.285246	-0.097557
H	-1.255162	-1.377782	-1.710196
H	-1.774543	-0.932541	0.015432
H	-0.103874	0.837018	-1.952032
C	-6.312478	-0.965423	1.841850
C	-5.473504	0.158063	1.930270
C	-4.631293	0.500750	0.862336
C	-5.482560	-1.416670	-0.403527
C	-6.314002	-1.749156	0.676197
H	-6.969096	-1.225314	2.677382
H	-5.476016	0.776700	2.833243
H	-3.980794	1.379082	0.924386
H	-5.496261	-2.019420	-1.317456
H	-6.971608	-2.620428	0.599624
C	3.449351	-3.469458	-0.584488
H	-3.131321	1.311138	-1.263456
C	0.557641	2.543160	0.209576
H	-0.982975	1.256170	0.995066
C	2.840942	4.157478	0.577479
C	2.162161	4.166017	-0.653792
C	1.025090	3.365549	-0.837759
C	1.235075	2.550065	1.445801
C	2.374466	3.350925	1.628418
H	3.722214	4.790415	0.721333
H	2.514832	4.805038	-1.469524
H	0.484061	3.390273	-1.789913
H	0.858256	1.935953	2.272871
H	2.884914	3.360336	2.596895
H	4.659795	0.032018	0.664511
H	4.380230	-3.954252	-0.247898
H	3.128394	-1.472626	2.761339
H	4.819744	-0.652821	-0.980221
H	3.517405	-3.250064	-1.660924
H	4.148874	-2.902022	2.371824
H	5.604232	-1.480249	0.411714
H	2.598833	-4.146725	-0.413141
H	2.354836	-3.034686	2.352992

SCF(BP86) = -928.692034331
H 0K = -928.319017
H 298K = -928.292307
G 298K = -928.383250
Solvent Correction(CH3Cl) = -0.04609117
BP86-D3 Correction = -0.05916102
Lowest frequencies = -81.8427 cm-1, 3.9282 cm-1

Int(I-II)$_{17}$ (anti, pro-*E*)

47

P	3.218643	-1.901679	0.315908
C	4.774251	-0.904211	0.142092
C	3.271481	-2.539202	2.058989
Au	1.268195	-0.715402	-0.235926
C	-0.475260	0.380815	-0.809608
C	-1.617617	-0.598508	-0.778127
S	-3.309307	0.196269	-1.470217
C	-0.649355	1.600373	0.090790
C	-4.574929	-0.276280	-0.275543
O	-1.801358	2.400705	-0.480925
H	-2.003314	3.150403	0.117274
H	-1.474942	-1.468925	-1.432431
H	-1.912565	-0.922392	0.232155
H	-0.250431	0.707327	-1.842701
C	-6.567811	-1.069994	1.515156
C	-5.653571	-0.068223	1.884127
C	-4.649974	0.334697	0.991591
C	-5.488414	-1.277125	-0.657278
C	-6.485032	-1.671362	0.248879
H	-7.350093	-1.377816	2.215285
H	-5.724041	0.406355	2.867580
H	-3.941868	1.120505	1.272456
H	-5.426656	-1.735049	-1.649430
H	-7.200607	-2.446558	-0.040838
C	3.514339	-3.404709	-0.732817
H	-2.829487	1.475776	-0.899247
C	0.546978	2.522506	0.193655
H	-0.955117	1.274485	1.104246
C	2.764707	4.250786	0.408439
C	2.155727	4.036475	-0.840858
C	1.051771	3.179280	-0.949386
C	1.153136	2.754401	1.444824
C	2.261289	3.610075	1.551878
H	3.622650	4.925410	0.490750
H	2.538143	4.545211	-1.731381
H	0.569592	3.030329	-1.921192
H	0.755602	2.259850	2.339438
H	2.721842	3.785846	2.529217
H	4.716057	-0.023421	0.799596
H	4.447125	-3.908940	-0.432148
H	3.188420	-1.693206	2.758359
H	4.869389	-0.559228	-0.898921
H	3.584101	-3.102379	-1.788987
H	4.214444	-3.078624	2.245066
H	5.654934	-1.509571	0.411581
H	2.668058	-4.099618	-0.620327
H	2.421224	-3.218555	2.224217

SCF(BP86) = -928.694185176
H 0K = -928.321164
H 298K = -928.294456
G 298K = -928.384022
Solvent Correction(CH3Cl) = -0.04501049
BP86-D3 Correction = -0.05885883
Lowest frequencies = 12.4946 cm-1, 17.1369 cm-1

TS(I-II)$_{17}$ (anti, pro-*E*)

47

P	3.220561	-1.903055	0.323128
C	4.745926	-0.848352	0.401526
C	3.162056	-2.753092	1.972835
Au	1.272911	-0.715842	-0.236938
C	-0.471949	0.373266	-0.816477
C	-1.620588	-0.603848	-0.787327
S	-3.300715	0.195315	-1.470258
C	-0.647625	1.590850	0.082262
C	-4.563221	-0.283697	-0.274128
O	-1.810328	2.393698	-0.495845
H	-2.040517	3.124187	0.116618
H	-1.474311	-1.474116	-1.441184
H	-1.908766	-0.932336	0.223523
H	-0.247175	0.699535	-1.849652
C	-6.555653	-1.083655	1.516264
C	-5.636553	-0.088351	1.890472
C	-4.634274	0.317739	0.997846
C	-5.481948	-1.278177	-0.660152
C	-6.478240	-1.675366	0.245166
H	-7.337283	-1.393921	2.216060
H	-5.702548	0.378868	2.877772
H	-3.923094	1.099039	1.283719
H	-5.423448	-1.729389	-1.655533
H	-7.197336	-2.445758	-0.048696
C	3.654131	-3.253052	-0.875066
H	-2.793102	1.493907	-0.890711
C	0.536432	2.525972	0.187265
H	-0.961469	1.269338	1.094008
C	2.728730	4.284833	0.411134
C	2.153323	4.031235	-0.846783
C	1.062176	3.158670	-0.960074
C	1.108727	2.797313	1.446533
C	2.204531	3.668073	1.558285
H	3.577182	4.970945	0.496635
H	2.552101	4.521206	-1.740612
H	0.606938	2.978900	-1.939585
H	0.695661	2.320566	2.343781
H	2.639610	3.873336	2.541478
H	4.602782	-0.058317	1.154389
H	4.579515	-3.764417	-0.563768
H	2.994482	-2.004352	2.762183
H	4.909320	-0.373792	-0.578187
H	3.793660	-2.818393	-1.876749
H	4.106813	-3.286524	2.166894
H	5.625073	-1.457605	0.667246
H	2.828981	-3.980374	-0.918511
H	2.326773	-3.469993	1.983476

SCF(BP86) = -928.694165327
H 0K = -928.321714
H 298K = -928.295734
G 298K = -928.383248
Solvent Correction(CH3Cl) = -0.04512896
BP86-D3 Correction = -0.05880550
Lowest frequencies = -77.1164 cm-1, 10.4952 cm-1

TS(I-II)$_{17}$ (anti, pro-*Z*)

47

C	4.383862	-1.021164	-1.349764
C	4.157417	-2.036669	1.404089
Au	1.172019	-0.729626	0.081646
C	-0.746416	0.254742	0.505778
C	-1.027779	0.243732	-0.887282
S	-3.376558	1.127484	-1.248820
C	-0.559804	1.577664	1.266371
C	-4.309234	-0.189051	-0.455492
O	-1.906487	2.105169	1.433242
H	-1.833746	2.966173	1.891586
H	-0.629256	1.028002	-1.540022
H	-1.334885	-0.686496	-1.372943
H	-1.284192	-0.500812	1.101263
C	-5.768576	-2.294924	0.679626
C	-5.704455	-2.156683	-0.717255
C	-4.977591	-1.104896	-1.293181
C	-4.365644	-0.318390	0.947425
C	-5.102079	-1.375418	1.505532
H	-6.344635	-3.112558	1.122970
H	-6.230233	-2.864644	-1.365277
H	-4.941377	-0.990064	-2.381584
H	-3.851699	0.404096	1.588926
H	-5.158487	-1.472763	2.594384
C	3.023778	-3.579912	-0.827832
C	0.374035	2.583924	0.603950
H	-0.151796	1.328161	2.266631
C	2.156950	4.400428	-0.623858
C	0.805569	4.372639	-1.003537
C	-0.081968	3.472573	-0.390576
C	1.728841	2.634649	0.993992
C	2.616326	3.533248	0.381934
H	2.844927	5.109407	-1.094602
H	0.436827	5.060780	-1.770748
H	-1.141266	3.480769	-0.668442
H	2.082486	1.980702	1.800788
H	3.660961	3.574868	0.707031
H	-3.039547	1.812746	-0.103083
P	3.211689	-1.853441	-0.179642
H	5.323043	-1.593844	-1.419127
H	4.597302	-0.003230	-0.990125
H	2.394348	-4.162339	-0.137858
H	2.535210	-3.548873	-1.813624
H	4.381988	-1.040697	1.815158
H	5.099107	-2.579880	1.223045
H	3.919144	-0.954343	-2.345279
H	4.011132	-4.060761	-0.921224
H	3.547269	-2.592427	2.132391

SCF(BP86) = -928.690246737
H 0K = -928.316838
H 298K = -928.290338
G 298K = -928.379133
Solvent Correction(CH3Cl) = -0.04642200
BP86-D3 Correction = -0.06268799
Lowest frequencies = -98.2618 cm-1, 8.7392 cm-1

IntII$_{17}$ (anti, pro-*E*)

47

P	2.748141	-2.441788	0.063024
C	4.499637	-1.976895	-0.317411
C	2.779880	-3.168810	1.765671
Au	1.246210	-0.664670	-0.143230
C	-0.447272	0.734119	-0.722509
C	-1.748654	0.034303	-0.413962
S	-3.107594	1.343791	-0.463654
C	0.254902	1.447040	0.266267
C	-4.566250	0.298659	-0.252481
O	-1.949693	1.063408	2.562305
H	-2.253281	1.493108	3.382498
H	-1.994466	-0.726135	-1.169920
H	-1.754699	-0.409345	0.593066
H	-0.269233	0.969080	-1.781346
C	-6.881076	-1.247347	0.077465
C	-5.993683	-1.074516	1.154001
C	-4.838291	-0.294805	0.998168
C	-5.449644	0.123668	-1.335866
C	-6.611176	-0.645942	-1.162561
H	-7.786416	-1.848470	0.207364
H	-6.208171	-1.537447	2.122556
H	-4.146762	-0.139234	1.833808
H	-5.227399	0.591604	-2.299831
H	-7.303635	-0.776940	-2.000011
C	2.349381	-3.849742	-1.071899
H	-2.477395	1.491515	1.847134
C	1.296029	2.466385	0.072707
H	-0.122023	1.335744	1.298658
C	3.267596	4.474885	-0.152109
C	2.702404	3.907097	-1.310971
C	1.725893	2.912964	-1.203746
C	1.871113	3.049936	1.230363
C	2.848570	4.046370	1.118237
H	4.027523	5.257104	-0.243239
H	3.019979	4.252343	-2.299538
H	1.283528	2.494143	-2.113405
H	1.533631	2.718720	2.218747
H	3.278632	4.493311	2.019537
H	4.825097	-1.182833	0.371632
H	3.081607	-4.661924	-0.934165
H	3.082196	-2.395707	2.488307
H	4.561679	-1.599969	-1.349598
H	2.379694	-3.499928	-2.114988
H	3.493062	-4.008317	1.803224
H	5.155570	-2.855522	-0.205261
H	1.338308	-4.223690	-0.850176
H	1.773184	-3.527589	2.028591

SCF(BP86) = -928.719400637
H 0K = -928.344798
H 298K = -928.316511
G 298K = -928.410083
Solvent Correction(CH3Cl) = -0.04818937
BP86-D3 Correction = -0.05585878
Lowest frequencies = 5.9327 cm-1, 19.7828 cm-1

IntII$_{17}$ (anti, pro-*Z*)

47

C	4.225307	-1.494969	-1.570871
C	4.264352	-2.163767	1.303036
Au	1.306045	-0.642385	0.193601
C	-0.798862	0.246579	0.283120
C	-1.464836	0.179453	-1.063538
S	-3.115169	1.091613	-0.998900
C	0.045077	1.245441	0.809743
C	-4.223461	-0.226361	-0.440178
O	-2.519488	1.998950	2.218962
H	-3.091535	2.630548	2.692506
H	-0.885285	0.641035	-1.875422
H	-1.697669	-0.857869	-1.346392
H	-1.267980	-0.391913	1.044392
C	-6.008953	-2.225849	0.379825
C	-5.760654	-2.027406	-0.988831
C	-4.869695	-1.026229	-1.405155
C	-4.476796	-0.413394	0.934540
C	-5.369350	-1.420359	1.336605
H	-6.708812	-3.004040	0.700209
H	-6.267551	-2.646328	-1.735880
H	-4.680554	-0.854576	-2.469510
H	-3.978741	0.227989	1.671436
H	-5.570106	-1.569373	2.402446
C	2.656888	-3.781839	-0.564609
C	0.641798	2.458285	0.225435
H	0.179484	1.181935	1.898008
C	1.878584	4.838154	-0.685214
C	0.895548	4.206552	-1.467686
C	0.282245	3.029274	-1.023297
C	1.620315	3.124073	1.012118
C	2.237137	4.295188	0.560409
H	2.351231	5.759472	-1.039225
H	0.595804	4.641686	-2.425887
H	-0.502861	2.583789	-1.636628
H	1.886499	2.711046	1.991733
H	2.987060	4.792101	1.183165
H	-2.799068	2.078413	1.280098
P	3.146052	-2.040047	-0.168182
H	5.070071	-2.192769	-1.690265
H	4.608253	-0.483685	-1.365954
H	2.065847	-4.194332	0.267179
H	2.042631	-3.791627	-1.477783
H	4.654974	-1.165306	1.551594
H	5.103484	-2.843116	1.081307
H	3.634271	-1.470271	-2.499016
H	3.556856	-4.399035	-0.719211
H	3.696150	-2.548992	2.163338

SCF(BP86) = -928.712248158
H 0K = -928.337751
H 298K = -928.309319
G 298K = -928.405301
Solvent Correction(CH3Cl) = -0.04858689
BP86-D3 Correction = -0.05778748
Lowest frequencies = 2.7321 cm-1, 7.0480 cm-1

*IntII$_{17}$ (anti, pro-*E*)

44

P	-2.888131	-2.002796	0.423231
C	-4.202983	-1.215783	1.462720
C	-3.772748	-2.759154	-1.016692
Au	-1.201620	-0.514483	-0.213590
C	0.863269	0.449563	-0.591473
C	1.664249	-0.399105	-1.541371
S	3.327535	0.427983	-1.787799
C	-0.099561	1.381305	-1.014947
C	4.259575	-0.230589	-0.388677
H	1.850045	-1.415261	-1.163552
H	1.201187	-0.463029	-2.539511
H	1.284250	0.538695	0.419480
C	5.812203	-1.203232	1.740774
C	5.435479	-2.064237	0.695250
C	4.670738	-1.580309	-0.376400
C	4.639640	0.634761	0.657263
C	5.420568	0.144901	1.716999
H	6.420690	-1.581807	2.567927
H	5.756593	-3.110731	0.702534
H	4.408418	-2.237957	-1.211694
H	4.332908	1.685003	0.629396
H	5.722252	0.819676	2.524404
C	-2.251128	-3.427245	1.420815
C	-0.632943	2.536796	-0.271691
H	-0.363174	1.361562	-2.083752
C	-1.693936	4.809331	1.026894
C	-0.757689	3.990448	1.687270
C	-0.229648	2.864856	1.047666
C	-1.574611	3.370577	-0.925006
C	-2.099056	4.498432	-0.281437
H	-2.098450	5.692418	1.530826
H	-0.432291	4.240325	2.701702
H	0.510039	2.248590	1.568800
H	-1.882578	3.132901	-1.949689
H	-2.817713	5.138122	-0.802313
H	-4.675325	-0.397636	0.898116
H	-3.083860	-4.095559	1.694242
H	-4.240341	-1.965010	-1.618391
H	-3.748564	-0.802160	2.375799
H	-1.768210	-3.048427	2.334364
H	-4.548991	-3.454420	-0.657745
H	-4.964755	-1.964202	1.735511
H	-1.509529	-3.986082	0.830095
H	-3.051737	-3.304557	-1.644387

SCF(BP86) = -852.285955458
H 0K = -851.935504
H 298K = -851.910337
G 298K = -851.997672
Solvent Correction(CH3Cl) = -0.04914679
BP86-D3 Correction = -0.04974030
Lowest frequencies = 10.0600 cm-1, 13.0386 cm-1

***IntII$_{17}$ (anti, pro-*Z*)**

44

C	-4.109950	-1.389343	1.555711
C	-4.040658	-2.352792	-1.233992
Au	-1.181000	-0.606393	-0.215918
C	0.930900	0.318931	-0.339696
C	1.552343	0.343906	1.028683
S	3.181981	1.275738	0.955917
C	0.055555	1.246350	-0.940370
C	4.281658	0.004077	0.295022
H	0.940054	0.856388	1.784803
H	1.771593	-0.673824	1.385224
H	1.420641	-0.372430	-1.040196
C	6.079999	-1.903541	-0.710326
C	5.643196	-1.984937	0.623199
C	4.752733	-1.028289	1.133166
C	4.722821	0.090350	-1.041008
C	5.624979	-0.864381	-1.538071
H	6.784103	-2.644962	-1.100623
H	6.011073	-2.784285	1.274319
H	4.434457	-1.067205	2.180020
H	4.367748	0.907128	-1.676943
H	5.972925	-0.793365	-2.573476
C	-2.428911	-3.704123	0.832899
C	-0.565785	2.485813	-0.439819
H	-0.082161	1.101484	-2.022727
C	-1.840783	4.893840	0.325470
C	-0.774173	4.384778	1.086807
C	-0.141037	3.192575	0.714546
C	-1.626278	3.030242	-1.213384
C	-2.263090	4.215016	-0.830821
H	-2.328622	5.827226	0.622576
H	-0.423122	4.928090	1.969297
H	0.715112	2.846020	1.295899
H	-1.943023	2.511461	-2.125673
H	-3.077889	4.617954	-1.439812
P	-2.972622	-2.034404	0.244836
H	-4.931388	-2.104141	1.726339
H	-4.523985	-0.420740	1.236871
H	-1.797341	-4.175634	0.064733
H	-1.843880	-3.594673	1.758665
H	-4.460397	-1.401691	-1.595262
H	-4.860543	-3.038273	-0.964294
H	-3.546920	-1.245546	2.490422
H	-3.309398	-4.338240	1.026516
H	-3.433502	-2.802246	-2.034434

SCF(BP86) = -852.2811788
H 0K = -851.930427
H 298K = -851.905323
G 298K = -851.992951
Solvent Correction(CH3Cl) = -0.04875727
BP86-D3 Correction = -0.05115393
Lowest frequencies = 7.6783 cm-1, 12.3048 cm-1

IntIII$_{17}$ (anti, pro-*S*)

57

P	-2.575055	-2.141000	-1.781204
C	-2.510949	-3.974219	-1.527762
C	-4.324588	-1.651946	-1.418119
C	-2.355908	-1.883207	-3.601974
Au	-1.018803	-0.982588	-0.479860
C	0.954900	-0.169228	0.398254
C	1.674397	0.661022	-0.629270
S	3.273829	1.267231	0.131704
C	-0.082804	0.326303	1.205036
C	4.331474	-0.183762	-0.065865
H	-2.076542	4.549678	1.898966
S	-2.505563	3.765222	0.873195
C	-1.063894	3.993310	-0.183239
H	1.115617	1.568727	-0.910253
H	1.478803	-1.085241	0.705750
C	-0.572069	-0.229512	2.481003
H	-0.471208	1.326095	0.954441
H	-1.509878	-4.346050	-1.794165
H	-3.267764	-4.469208	-2.157826
H	-2.705714	-4.202932	-0.468928
H	-1.354416	-2.226900	-3.902384
H	-2.448857	-0.811224	-3.833104
H	-3.121605	-2.448347	-4.157919
H	-4.453437	-0.576832	-1.614870
H	-4.545678	-1.848723	-0.358108
H	-5.016079	-2.229033	-2.053496
C	-1.584218	-1.176153	4.941286
C	-0.489826	-1.820232	4.332572
C	0.014581	-1.354355	3.114280
C	-1.669871	0.413372	3.106200
C	-2.170529	-0.057444	4.326832
H	-1.970462	-1.542479	5.897483
H	-0.023149	-2.682384	4.818887
H	0.878371	-1.854143	2.664145
H	-2.117315	1.290461	2.623075
H	-3.014723	0.451430	4.802195
H	1.929984	0.094166	-1.537008
C	1.127288	4.286716	-1.934304
C	1.265001	4.590802	-0.569097
C	0.180919	4.435585	0.310350
C	-1.207751	3.691984	-1.554202
C	-0.111134	3.833778	-2.420277
H	1.973822	4.417371	-2.614703
H	2.221264	4.955186	-0.180767
H	0.303466	4.671675	1.372505
H	-2.182866	3.385378	-1.947440
H	-0.237262	3.616949	-3.486186
C	6.070857	-2.375525	-0.334141
C	5.654140	-1.665049	-1.473370
C	4.796084	-0.563036	-1.342993
C	4.750902	-0.894409	1.077126
C	5.625032	-1.985296	0.938714
H	6.751362	-3.226251	-0.439001
H	6.015495	-1.956176	-2.464917
H	4.499751	0.015380	-2.224377
H	4.399653	-0.582996	2.065894
H	5.956188	-2.530331	1.828479

SCF(BP86) = -1094.75982937
H 0K = -1094.312313
H 298K = -1094.278303
G 298K = -1094.391185
Solvent Correction(CH3Cl) = -0.04791583
BP86-D3 Correction = -0.07179615
Lowest frequencies = 5.3215 cm-1, 8.1185 cm-1

TS(III-IV)$_{17}$ (anti, pro-*S*)

57

P	4.277278	0.207173	-0.369105
C	5.175602	1.702468	-0.998156
C	5.031655	-0.144327	1.287333
C	4.840264	-1.184821	-1.456550
Au	1.950843	0.444033	-0.279064
C	-0.227827	0.582007	-0.050003
C	-0.363930	1.458057	-1.177908
S	-2.621141	2.223471	-1.520640
C	-0.808167	-0.828540	-0.103406
C	-2.798760	3.402704	-0.174961
H	-2.966473	1.053126	-0.812367
S	-2.646936	-0.700617	0.444930
C	-3.364504	-2.205471	-0.243564
H	-0.335642	1.038108	-2.190116
H	-0.317927	1.073640	0.933128
C	-0.079770	-1.856097	0.738503
H	-0.852581	-1.173059	-1.151038
H	4.835738	1.929011	-2.020236
H	6.262767	1.520983	-1.002939
H	4.948995	2.562822	-0.350135
H	4.497745	-1.004549	-2.486948
H	4.399633	-2.126669	-1.095878
H	5.939678	-1.261283	-1.441917
H	4.590685	-1.065361	1.697894
H	4.811565	0.689000	1.972003
H	6.123064	-0.265412	1.193285
C	1.279104	-3.805953	2.271049
C	0.888942	-2.589168	2.859963
C	0.211693	-1.623325	2.102163
C	0.308359	-3.082542	0.159984
C	0.984879	-4.051257	0.920659
H	1.797712	-4.563080	2.867606
H	1.100985	-2.398388	3.916959
H	-0.112121	-0.692069	2.579766
H	0.074785	-3.280523	-0.892409
H	1.271626	-5.001299	0.458447
H	-0.007180	2.488666	-1.090679
C	-4.549080	-4.542068	-1.247824
C	-4.347982	-3.438338	-2.094269
C	-3.760608	-2.265951	-1.595919
C	-3.564997	-3.310925	0.607794
C	-4.160345	-4.476571	0.099858
H	-5.015408	-5.451662	-1.638836
H	-4.660054	-3.484512	-3.142381
H	-3.621517	-1.398680	-2.249350
H	-3.260790	-3.250362	1.656675
H	-4.320981	-5.333579	0.761397
C	-3.044235	5.335179	1.841368
C	-2.561745	5.700251	0.573288
C	-2.437134	4.739539	-0.440636
C	-3.289050	3.027787	1.092075
C	-3.408151	4.003008	2.094857
H	-3.145083	6.089787	2.627045
H	-2.287703	6.739290	0.366178
H	-2.074781	5.028955	-1.432822
H	-3.592250	1.995681	1.290405
H	-3.796295	3.715671	3.077060

SCF(BP86) = -1094.74138322
H 0K = -1094.293811
H 298K = -1094.261642
G 298K = -1094.367133
Solvent Correction(CH3Cl) = -0.04396925
BP86-D3 Correction = -0.07737049
Lowest frequencies = -120.1802 cm-1, 6.1675 cm-1

IntIV$_{17}$ (anti, pro-*S*)

57

P	3.805270	-0.043374	-1.095864
C	4.479224	0.841909	-2.576656
C	4.628227	0.738835	0.367483
C	4.493281	-1.760035	-1.190342
Au	1.468658	0.000891	-0.982576
C	-0.691161	-0.142998	0.034101
C	-0.743859	0.039773	-1.350654
S	-3.928219	3.207325	0.252023
C	-0.843669	-1.468231	0.710831
C	-2.199740	3.388379	0.693343
H	-4.376011	2.897476	1.498899
S	-2.663930	-1.402757	1.277518
C	-3.528208	-1.736894	-0.267153
H	-0.943839	-0.809223	-2.015638
H	-0.699564	0.747064	0.678430
C	0.049922	-1.722325	1.904428
H	-0.776857	-2.299457	-0.009405
H	4.083501	0.377550	-3.492756
H	5.579879	0.785191	-2.581220
H	4.164977	1.896292	-2.547935
H	4.096829	-2.264281	-2.084782
H	4.185694	-2.324216	-0.296812
H	5.593500	-1.724264	-1.244942
H	4.316740	0.213838	1.283122
H	4.320733	1.792995	0.438634
H	5.723615	0.680059	0.260044
C	1.691291	-2.281577	4.134324
C	1.080571	-1.023055	4.005085
C	0.264877	-0.742006	2.897906
C	0.663622	-2.985171	2.045065
C	1.481037	-3.262696	3.151120
H	2.320883	-2.499813	5.002512
H	1.231598	-0.259353	4.774658
H	-0.212269	0.240878	2.817342
H	0.489636	-3.757792	1.287117
H	1.945106	-4.249104	3.249801
H	-0.922984	1.041820	-1.757008
C	-4.968047	-2.276411	-2.616259
C	-4.886026	-0.961384	-2.130832
C	-4.163916	-0.683360	-0.958241
C	-3.619148	-3.061464	-0.748169
C	-4.333715	-3.324644	-1.925862
H	-5.534672	-2.488405	-3.528368
H	-5.387697	-0.146553	-2.662648
H	-4.099089	0.339926	-0.572496
H	-3.149905	-3.876632	-0.187830
H	-4.410550	-4.352069	-2.295540
C	0.545224	3.742482	1.263836
C	0.051738	3.986537	-0.030040
C	-1.310919	3.812800	-0.319050
C	-1.705762	3.138476	1.992102
C	-0.341182	3.321580	2.270312
H	1.601407	3.911495	1.494894
H	0.723176	4.345656	-0.817281
H	-1.690486	4.037800	-1.321685
H	-2.386712	2.824746	2.790026
H	0.022731	3.154367	3.289576

SCF(BP86) = -1094.75178487
H 0K = -1094.304624
H 298K = -1094.270666
G 298K = -1094.382295
Solvent Correction(CH3Cl) = -0.04839041
BP86-D3 Correction = -0.07712229
Lowest frequencies = 6.8348 cm-1, 8.4793 cm-1

***IntIV$_{17}$ (anti, pro-*S*)**

44

P	3.478075	-0.935311	0.292803
C	4.181991	-2.634329	0.508827
C	4.507670	-0.131226	-1.019456
C	3.865871	-0.028603	1.859532
Au	1.194081	-0.955863	-0.221116
C	-0.919060	-0.040706	-0.895433
C	-0.964956	-1.412241	-0.627807
C	-1.355578	1.021976	0.061780
S	-3.097522	1.430558	-0.592647
C	-4.038030	-0.004260	-0.046438
H	-1.374867	-1.774032	0.322920
H	-0.699250	0.292324	-1.918228
C	-0.513940	2.279417	0.079375
H	-1.483615	0.621187	1.079954
H	3.654855	-3.149298	1.326338
H	5.255969	-2.568067	0.747328
H	4.046298	-3.208341	-0.420415
H	3.344342	-0.511524	2.699926
H	3.517185	1.011629	1.772849
H	4.952411	-0.041929	2.043927
H	4.182210	0.911899	-1.150524
H	4.374782	-0.669270	-1.970351
H	5.570705	-0.153203	-0.729035
C	1.042484	4.633221	0.175550
C	0.589806	4.137098	-1.058707
C	-0.183470	2.967371	-1.108944
C	-0.061736	2.788839	1.314683
C	0.714174	3.957518	1.362323
H	1.638605	5.550234	0.212540
H	0.829039	4.668228	-1.985252
H	-0.548289	2.606859	-2.077444
H	-0.330785	2.273237	2.243937
H	1.050266	4.347160	2.328435
H	-0.925592	-2.135560	-1.450873
C	-5.613467	-2.180777	0.770665
C	-5.286698	-2.023314	-0.585847
C	-4.493569	-0.940502	-0.999270
C	-4.379038	-0.154249	1.315570
C	-5.157919	-1.248199	1.719687
H	-6.232912	-3.024912	1.089122
H	-5.650435	-2.742341	-1.326573
H	-4.239770	-0.806579	-2.055305
H	-4.054127	0.595578	2.044177
H	-5.427388	-1.361576	2.774481

SCF(BP86) = -852.279305431
H 0K = -851.929175
H 298K = -851.904052
G 298K = -851.990535
Solvent Correction(CH3Cl) = -0.048577231
BP86-D3 Correction = -0.05401734
Lowest frequencies = 8.1690 cm-1, 16.4009 cm-1
